# An Integrated Genomic Strategy Delineates Candidate Mediator Genes Regulating Grain Size and Weight in Rice

**DOI:** 10.1038/srep23253

**Published:** 2016-03-22

**Authors:** Naveen Malik, Nidhi Dwivedi, Ashok K. Singh, Swarup K. Parida, Pinky Agarwal, Jitendra K. Thakur, Akhilesh K. Tyagi

**Affiliations:** 1 National Institute of Plant Genome Research (NIPGR), Aruna Asaf Ali Marg, New Delhi 110067, India; 2 Division of Genetics, Rice Section, Indian Agricultural Research Institute (IARI), New Delhi 110012, India

## Abstract

The present study deployed a Mediator (*MED*) genes-mediated integrated genomic strategy for understanding the complex genetic architecture of grain size/weight quantitative trait in rice. The targeted multiplex amplicon resequencing of 55 *MED* genes annotated from whole rice genome in 384 accessions discovered 3971 SNPs, which were structurally and functionally annotated in diverse coding and non-coding sequence-components of genes. Association analysis, using the genotyping information of 3971 SNPs in a structured population of 384 accessions (with 50–100 kb linkage disequilibrium decay), detected 10 *MED* gene-derived SNPs significantly associated (46% combined phenotypic variation explained) with grain length, width and weight in rice. Of these, one strong grain weight-associated non-synonymous SNP (G/A)-carrying *OsMED4_2* gene was validated successfully in low- and high-grain weight parental accessions and homozygous individuals of a rice mapping population. The seed-specific expression, including differential up/down-regulation of three grain size/weight-associated *MED* genes (including *OsMED4_2*) in six low and high-grain weight rice accessions was evident. Altogether, combinatorial genomic approach involving haplotype-based association analysis delineated diverse functionally relevant natural SNP-allelic variants in 10 *MED* genes, including three potential novel SNP haplotypes in an *OsMED4_2* gene governing grain size/weight differentiation in rice. These molecular tags have potential to accelerate genomics-assisted crop improvement in rice.

Rice (*Oryza sativa* L.) is a vital staple food and nutritional source for over half of the human population across the globe. With decreasing farmland and increase in global population, the improvement of rice yield and productivity is imperative to sustain world food security in the present scenario of climate change. Enhancing grain yield is thus the primary objective of current rice breeding and genomics research^1^,^2^. Grain size, the major determinant of grain weight and one of the crucial components of grain yield is positively correlated with grain length, grain width, grain thickness and grain filling degree^3^. Therefore, grain size/weight is a known target trait for both domestication and artificial breeding for enhancing productivity in rice^2^,^4^,^5^. Advancements of genome mapping, sequencing, and functional genomics have provided powerful tools to uncover the molecular basis of complex quantitative grain size traits in rice. These efforts led to identify thousands of low-resolution QTLs associated with rice grain size/weight. Of these, a set of selected high-resolution major QTLs has been narrowed-down into six most promising genes (*GS3, GW2, qSW5*/*GW5, GS5, TGW6* and *GIF1*) governing grain size (length, width, weight and filling degree) through map-based cloning and mutant complementation analyses in rice^4^,^6^,^7^,^8^,^9^,^10^,^11^,^12^,^13^,^14^. Detailed molecular characterization and functional validation of these six genes have inferred their negative (*GS3, GW2* and *qSW5*/*GW5*) or positive (*GS5, TGW6* and *GIF1*) regulatory mechanism during plant growth and seed development controlling grain size/weight in rice^1^,^2^. Most of these genes majorly regulate signaling pathways mediated by phytohormones, proteasomal degradation and G-proteins to control cell elongation and proliferation in the seeds of rice. Genetic association analysis of these known cloned/characterized genes with grain morphology of numerous germplasm lines reflects existence of their multiple natural allelic variants in a large rice gene pool with certain degree of pleiotropic/epistasis effects on other undesirable grain yield component traits^1^,^2^. These intricate and complex interactions of grain size genes impose major hindrances for marker-assisted genetic improvement of grain size/weight and yield in rice. Henceforth, a clear understanding of the genetic/molecular basis of grain size/weight variation, by identification of novel functionally relevant genes and alleles governing the target traits, is extremely important for rice genomics-assisted crop improvement program.

In eukaryotes, transcription of genes is regulated by various transcription factors and it is further assisted through different cofactors^15^. A number of studies have established Mediator complex as a crucial cofactor involved in RNA pol II mediated transcription regulation^16^,^17^. The Mediator complex encompassing more than 25 subunits in eukaryotes, acts as an interface between transcriptional activators/repressors and RNA pol II and thus can have both inducing as well as repressing effect on transcription^17^,^18^,^19^. The role of these Mediator subunits in transcription has been established in the formation of pre-initiation complex, transcription initiation and elongation, splicing, gene looping and transcription termination^19^,^20^,^21^,^22^,^23^,^24^,^25^,^26^,^27^,^28^,^29^,^30^. Abundance of intrinsic disorder regions (IDRs) in MED subunits and the modular arrangement consisting of Head, Middle, Tail and Kinase modules makes Mediator a versatile regulator, as it can interact with various factors and attain diverse configurations for regulating multiple processes in eukaryotes^18^,^31^,^32^. Plant Mediator complex composition is no different from animals except for the absence of MED1 and presence of some plant-specific MED subunits^33^,^34^. In plants, specifically in *Arabidopsis*, diverse functions ranging from development to biotic and abiotic stresses have been attributed to different MED subunits. For instance, MED12 and MED13, which are the parts of Kinase module, regulate timing of embryo patterning^35^. Similarly, STRUWELLPETER, identified as MED14, is known to regulate cell proliferation in *Arabidopsis*^36^. MED18 regulates flowering time and identity of floral organs by transcriptional regulation of floral regulators^37^. MED16, MED2 and MED14 are vital for providing freezing tolerance in *Arabidopsis*^38^. A single MED subunit, MED25 regulates multiple growth, development and stress tolerance traits, including plant organ size control^39^, root hair differentiation^40^, lateral root development through auxin signalling^41^, defense against fungal pathogens by regulating jasmonic acid^42^,^43^ and abscissic acid signalling^43^. MED15 and MED16 modulate response to biotic factors by regulating salicylic acid response^44^,^45^. Likewise, MED21 regulates response against necrotrophic fungi^46^ and MED8 controls flowering time and resistance towards necrotrophic fungi^42^. The aforementioned previous studies in *Arabidopsis* and expression profiling of *MED* genes in rice and *Arabidopsis* ascertain their definitive role in diverse useful yield component and stress tolerance traits, besides basal regulation of gene expression^34^,^47^,^48^,^49^,^50^. However, none of the *MED* genes regulating specific agronomic traits, including grain size/yield has been identified and functionally validated so far to be utilized in marker-assisted selection for genetic improvement of rice. In this context, it would be interesting to decipher the possible role of *MED* genes in grain size/weight regulation and seed development in rice.

An integrated approach of SNP marker-based high resolution candidate gene-based association analysis, traditional QTL mapping, differential expression profiling and molecular haplotyping is well documented as an attractive strategy for efficient dissection of complex quantitative yield component traits in multiple crop plants, including rice^1^,^2^,^4^,^11^,^51^,^52^,^53^,^54^,^55^,^56^,^57^. This strategy will also prove useful in rapid identification of natural allelic variants within genes associated with grain size/weight and understanding their mechanism of interaction/regulation for grain size/weight variation in rice cultivars adapted to diverse agroclimatic conditions. All these inputs obtained from the combinatorial approach could essentially expedite marker-assisted breeding for selecting cultivars with large grain size and more yield in rice.

Considering the aforesaid possibilities, genetic association analysis of grain size and weight traits was performed based on precise field phenotyping and genotyping of informative SNPs mined from 55 *MED* subunit genes (distributed across rice genome) in 384 diverse low and high grain weight rice accessions (association panel). This strategy was further integrated with traditional bi-parental mapping population validation, differential expression profiling and gene-based SNP haplotyping/LD (linkage disequilibrium) mapping to delineate functionally relevant natural allelic variants and haplotypes in the potential *MED* gene(s) regulating grain size (grain length, grain width) and 1000-grain weight in rice.

## Results and Discussion

### Discovery, annotation and genotyping of *MED* gene-derived SNPs

The implication of integrated genomic strategy (combining association analysis, QTL mapping, expression profiling and molecular haplotyping) for efficient dissection of complex quantitative traits and rapid identification of potential candidate genes especially regulating grain size/weight traits is well demonstrated in crop plants, including rice^2^,^4^,^55^,^56^,^57^,^58^. In this context, the current study integrated candidate gene-based association mapping with bi-parental mapping population validation, differential gene expression profiling and gene-based haplotyping/LD mapping to scale-down the candidate Mediator (*MED*) genes governing grain size, including grain length, grain width and grain weight in rice. A diverse array of *MED* genes is known to regulate multiple agronomic traits, including yield component and abiotic/biotic stress tolerance traits in crop plants^34^,^49^,^59^. Primarily, to perform candidate gene-based association mapping, the diverse coding and non-coding (introns, URRs and DRRs along with 5′ and 3′ UTRs, respectively) sequence components of 55 *MED* genes annotated from whole rice genome were sequenced and genotyped in low and high grain weight 384 rice accessions (belonging to an association panel) by targeted multiplex-amplicon resequencing to discover potential gene-derived SNP allelic variants.

**Figure 1:**
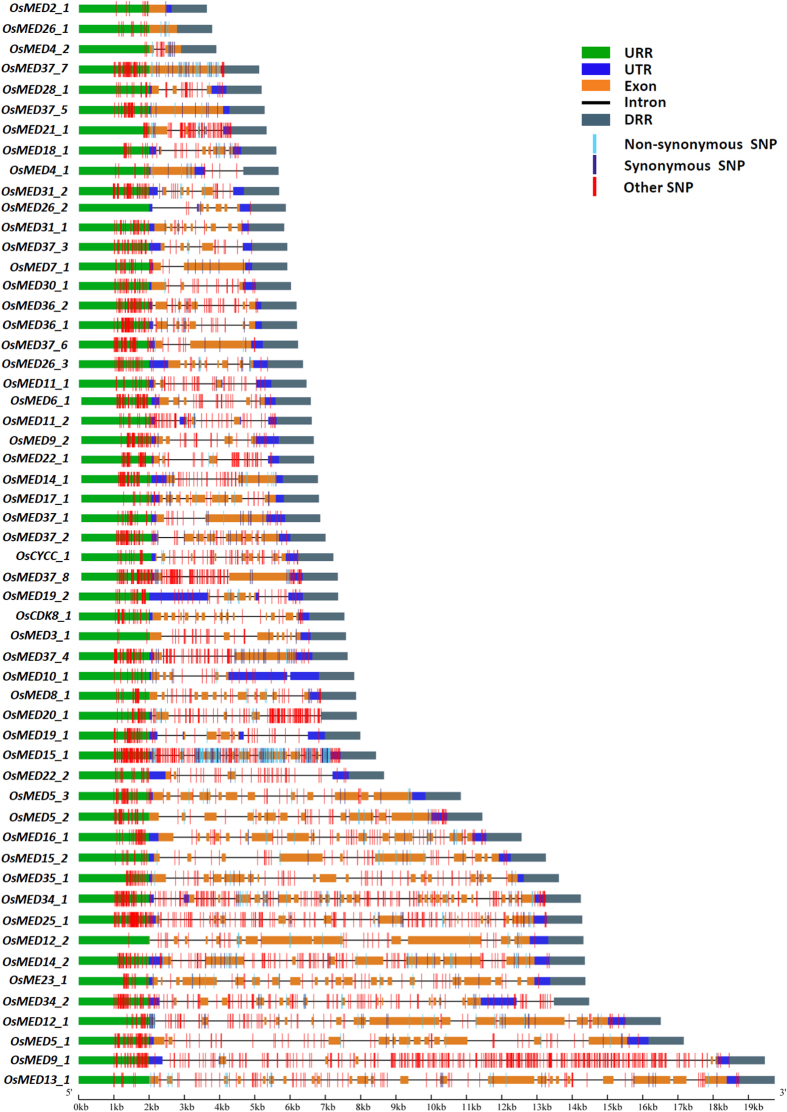
Gene structure of 55 Mediator (*MED*) subunit genes depicting the structural annotation of SNPs in different coding (synonymous and non-synonymous) as well as non-coding regulatory (URRs and DRRs along with 5′ and 3′ UTRs, respectively) and intronic sequence components of genes. URR: upstream regulatory region, DRR: downstream regulatory region, UTR: 5′ or 3′ untranslated region. The sub-genic regions are indicated as per the *MED* gene annotation information available at Rice Genome Annotation Project (RGAP, release 7.0).

The targeted resequencing of coding and non-coding intronic and regulatory sequence components of 55 *MED* genes in 384 diverse low and high grain weight rice accessions (association panel) using the Illumina TruSeq Custom Amplicon strategy mined 3971 high-quality SNPs with an average frequency of 72.2 SNPs/gene (Fig. 1, Tables 1, S1). These SNPs were physically mapped across 12 chromosomes of rice with a highest (14.3%, 568 SNPs) and lowest (1.8%, 73) density on chromosomes 9 and 6, respectively (Figure S1, Table 1). The structural annotation of 3971 SNPs in the *MED* genes revealed the presence of 3306 (83.3%) and 665 (16.7%) SNPs in the non-coding and coding regions of genes, respectively (Fig. 1, Tables 1, S1). Among the non-coding SNPs, 1545 (46.7%) and 1761 (53.3%) SNPs were derived from the regulatory (URRs and DRRs along with 5′ and 3′ UTRs, respectively) and intronic sequence components of genes, respectively. The 665 coding SNPs included 323 (48.6%) non-synonymous (missense and nonsense) and 342 (51.4%) synonymous SNPs in the *MED* genes, respectively (Fig. 1, Tables 1, S1). The informative SNPs (specifically the non-synonymous and regulatory SNPs) discovered from diverse coding and non-coding sequence components of *MED* genes can serve as a useful genomic resource to be utilized for manifold genomics-assisted breeding applications, including genetic association analysis and targeted mapping of potential genes regulating multiple traits of agronomic importance in rice.

### *MED* gene-based association mapping of rice grain size

For candidate gene-based association mapping, the genotyping data of 3971 informative *MED* gene-derived SNPs (with 5% minor allele frequency) exhibiting polymorphism among 384 rice accessions was utilized. The use of these SNPs in determination of population genetic structure and PCA (principal component analysis) differentiated all 384 rice accessions from each other, which clustered into two distinct population groups- POP I and POP II. The determination of LD patterns in a population of 384 accessions using 3971 SNPs (physically mapped on 12 chromosomes) exhibited a broader LD estimate (r^2^: 0.32–0.78) and faster LD decay (r^2^ decreased to half of its maximum value) nearly at 50–100 kb physical distance of rice chromosomes. This estimate is comparable with the chromosomal LD decay documented in previous candidate gene-based and genome-wide association mapping studies of rice^1^,^51^,^52^,^53^,^54^. Therefore, the LD decay documented in the present study using the genotyping information of *MED* gene-derived SNPs mapped on 12 rice chromosomes is adequate enough for efficient trait association mapping to identify potential genic loci governing useful agronomic traits, including grain size/weight in rice.

The normal frequency distribution along with a broader phenotypic variation and higher heritability for grain size, including grain length (6.6 to 11.2 mm, mean ± SD: 8.5 ± 0.71, mean CV: 8% and mean H^2^: 75%), grain width (1.9 to 3.5 mm, 2.8 ± 0.31, 11% and 73%) and 1000-grain weight (15.4 to 39.2 g, 26.5 ± 4.8, 18% and 82%), in 384 rice accessions were observed across two diverse geographical locations/years based on ANOVA (Figure S2, Table S2). The ANOVA outcomes inferred a highly significant difference (P < 0.0001) among rice accessions for grain size/weight trait variation despite significant environmental (years and geographical locations) and block replication effects on these traits (Table S3). A significant interaction between genotypes (G)/accessions and environments (E) for grain size/weight traits was evident. These observations infer complex quantitative genetic inheritance pattern of grain size (grain length, grain width) and grain weight traits in rice and thus require an efficient integrated genomics-assisted breeding strategy (like association/genetic mapping and molecular haplotyping) for genetic dissection of these target traits in rice. Further, consistent phenotypic expression of grain size traits, based on high heritability across diverse geographical locations/years in 384 accessions of an association panel, implicates the robustness of grain size/weight phenotypic data generated in the present study for trait association mapping in rice. Therefore, the mean phenotyping data of accessions, revealing consistent phenotypic expression for grain size/weight traits across geographical locations/years, was utilized for subsequent SNP marker-trait association study.

**Figure 2:**
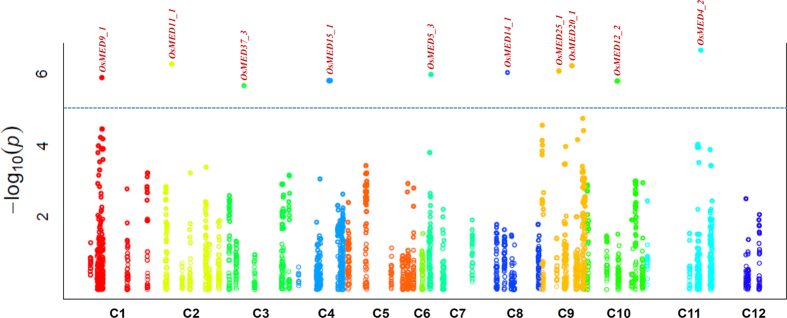
Manhattan plot illustrating the significance of SNP loci-containing *MED* genes for grain weight trait association in rice. X-axis represents the relative density of SNPs mined from *MED* subunit genes distributed over 12 rice chromosomes. Y-axis indicates the-log_10_ (P) value to scan the significant trait-associated SNP loci at a cut-off P ≤ 10^−5^.

The use of CMLM and P3D/EMMAX model-based approaches (at a FDR cut-off ≤0.05) in genetic association analysis identified 10 SNPs in 10 *MED* genes exhibiting significant association (at a P value ≤ 10^−5^) with grain length, grain width and grain weight in rice (Fig. 2, Table 2). These grain size-associated *MED* gene-derived SNPs were mapped on nine chromosomes (excluding chromosomes 5, 6 and 12) of rice. Of these, a maximum of two trait-associated SNPs were represented from rice chromosome 9. Six and four grain size trait-associated genomic SNP loci were derived from coding (six non-synonymous SNPs) and non-coding [URR (three SNPs) and intronic (one SNP)] sequence components of 10 *MED* genes, respectively (Table 2). The estimated minor allele frequency (MAF) for 10 grain size/weight-associated *MED* genes in a constituted association panel varied from 15–26% with an average of 21%. The proportion of phenotypic variation for grain length, grain width and grain weight explained by maximum effect 10 SNP loci in 10 *MED* genes (*OsMED9_1, OsMED11_1, OsMED37_3, OsMED15_1, OsMED5_3, OsMED14_1, OsMED25_1, OsMED20_1, OsMED12_2* and *OsMED4_2*) among 384 rice accessions varied from 15 to 33% R^2^. The percentage of combined PVE (phenotypic variation explained) revealed by all significant 10 *MED* gene-derived SNPs was 46%. Interestingly, seven, four and 10 *MED*-gene based SNPs associated with grain length, grain width and grain weight revealing combined PVE of 43% (varied from 15–33%), 41% (18–33%) and 48% (15–33%) were identified, respectively (Table 2). Five (grain length and grain weight), one (grain width and grain weight) and two (grain length, grain width and grain weight) *MED* gene-derived SNPs exhibited significant association with multiple grain size traits in rice. A strong association of one non-synonymous SNP (G/A) scanned in *OsMED4_2* gene with grain length, grain width and grain weight (33% PVE with P value 0.3 × 10^−8^) followed by one regulatory (URR) SNP (T/A) identified in *OsMED25_1* gene with grain length and grain weight (28% PVE with P value 1.3 × 10^−6^) was evident (Table 2). The added-advantage of CMLM and P3D/EMMAX strategies based on their efficacy towards scanning of non-spurious SNP marker-trait association with maximal statistical power and high prediction accuracy over other association model-based approaches hitherto has been well-documented in crop plants^57^,^60^,^61^. In this perspective, the potential *MED* gene-derived SNP loci associated with grain length, grain width and grain weight scanned in this study deploying CMLM and P3D/EMMAX-based association mapping strategy is relevant and thus can be applied for deciphering the complex gene regulatory networks underlying grain size/weight trait variation in rice. Notably, six (*OsMED15_1, OsMED14_1, OsMED12_2, OsMED25_1, OsMED5_3* and *OsMED4_2*) of the 10 high grain size/weight-associated SNPs-containing *MED* genes identified in our study are known to govern diverse growth and developmental processes in plants^34^,^35^,^36^,^39^,^49^,^59^,^62^,^63^,^64^. Especially, the role of *OsMED15* gene in controlling seed development as well as its significant association potential for high and low grain weight differentiation is well-demonstrated in rice^49^,^59^. Similarly, the involvement of another gene *MED12* in controlling embryo patterning during seed development has been deciphered in *Arabidopsis*^35^. *MED25* of *Arabidopsis* has been found to be involved in the regulation of timing and process of flowering, which though not demonstrated experimentally, may further affect timing and process of seed setting and development^42^. The heterozygote mutant lines of *Atmed14* are dwarf with abnormal architecture including abnormal floral structure suggesting a probable influence on seed setting and maturation^36^. As the *med14* mutant of *Arabidopsis* shows reduced cell numbers in all the aerial organs^36^, there is a possibility that *MED14* can directly or indirectly affects overall seed yield. In rice, MED4 interacts with SAD1 to regulate tiller number which can affect the overall grain yield^64^. The essential role of a *MED5* gene in repressing phenyl propanoid biosynthesis^62^ as well as in regulating proper plant growth/development, including cell wall lignification has been demonstrated in *Arabidopsis*^63^. Therefore, grain size/weight trait-associated 10 SNP loci identified from diverse non-synonymous coding and regulatory sequence components of 10 *MED* genes are assumed to be functionally relevant. Such non-synonymous and regulatory SNPs are known to regulate diverse grain size and weight traits during seed development in crop plants, including rice^2^,^4^,^55^,^56^,^57^,^58^. Henceforth, the trait-associated novel natural SNP allelic variants-containing *MED* genes identified by candidate gene-based association mapping can essentially be utilized for establishing rapid marker-trait linkages and efficient identification/mapping of genes governing grain size/weight trait in rice.

### Validation of grain size-associated *MED* genes in a mapping population

**Figure 3:**
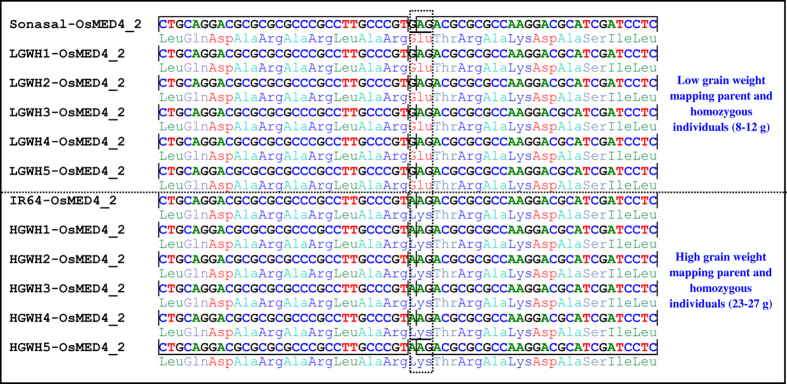
SNP (G to A) depicting the missense non-synonymous amino acid substitution [Glutamic acid (GAG) to Lysine (AAG)] in an *OsMED4*_2 gene differentiated the low (8–12 g) and high (23–27 g) grain weight parental accessions and representative homozygous individuals of a F_4_ mapping population (IR 64 × Sonasal). The *MED* gene sequence region-carrying a non-synonymous SNP allelic variant is highlighted with a dotted box.

To validate 10 *MED* gene-derived SNPs exhibiting significant association with grain length, grain width and grain weight in rice, the SNPs exhibiting parental polymorphism (between IR 64 and Sonasal) were genotyped in 10 of each low and high grain weight homozygous individuals of a F_4_ mapping population (IR 64 × Sonasal). One non-synonymous SNP (G/A)-containing *OsMED4_2* gene showing strong association with grain size, grain width and grain weight (based on trait association analysis), was validated in a mapping population (Fig. 3). All low (8–12 g) and high (23–27 g) grain weight parental accessions and homozygous individuals of a mapping population contained the identical high (A) and low (G) grain size-associated SNP alleles identified from an *OsMED4_2* gene (Fig. 3). Henceforth, a stronger SNP allele effect of *OsMED4_2* gene with high and low grain weight differentiation in rice was apparent. In contrast, SNP alleles mined from nine other *MED* genes revealing association with high and low grain weight differentiation could not correspond to the phenotypes of the low and high grain weight mapping parents and homozygous individuals. However, large-scale validation and genotyping of all 10 grain size/weight-associated *MED* gene-derived SNPs in the numerous bi-parental mapping populations contrasting for grain size/weight are required to ascertain the definitive association potential of these identified functionally relevant molecular tags in grain size/weight trait regulation in rice. Altogether, 10 grain size/weight-associated *MED* genes, including one non-synonymous SNP-containing *OsMED4_2* (validated by both trait association analysis and in bi-parental mapping population) were selected as target candidates for grain weight/size trait regulation by their further validation through differential expression profiling in rice.

### Differential expression profiling of grain size-associated *MED* genes

**Figure 4:**
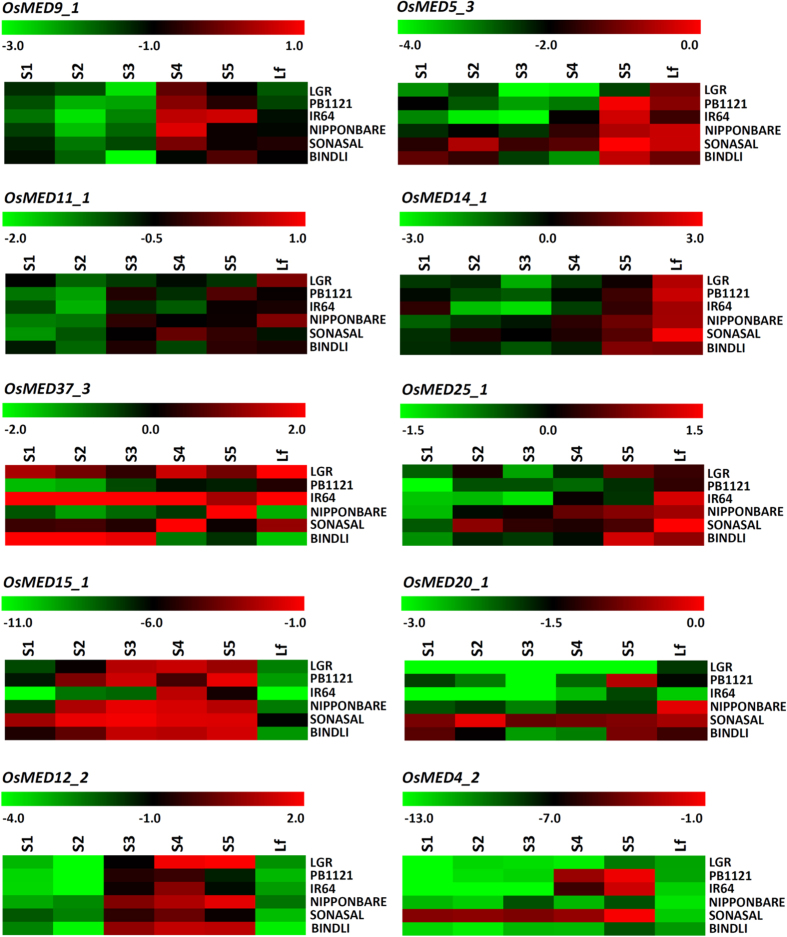
Heat maps depicting the differential expression profiles of 10 grain size/weight-associated *MED* genes in five seed developmental stages (S1–S5) of six contrasting low (Sonasal and Bindli) and high (LGR, Pusa Basmati 1121, Nipponbare and IR 64) grain weight rice accessions as compared to flag leaves. Color scale at the top of each map represents –ΔCt (Ct ubiquitin - Ct gene) values. Green, black and red color shows low, medium and high expression, respectively. Lf- flag leaf and PB1121- Pusa Basmati 1121.

**Figure 5:**
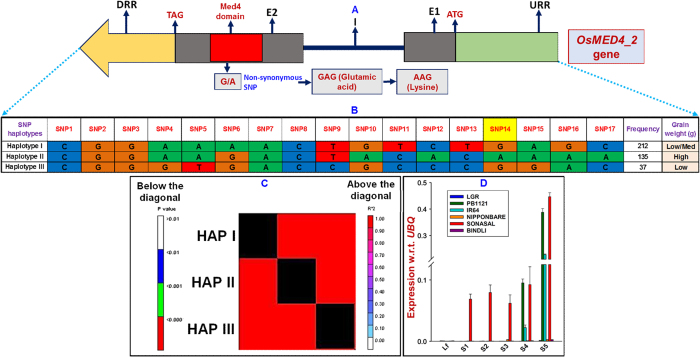
The molecular haplotyping and SNP haplotype-specific association analysis/LD mapping in an *OsMED4*_2 gene (A) validating its strong association potential for grain weight/size differentiation in rice. The genotyping of 17 SNPs, including one missense non-synonymous SNP (G/A, shaded with yellow colour) [encoding for Glutamic acid (GAG) to Lysine (AAG)] among 384 rice accessions (association panel) constituted three haplotypes (B). (C) Three SNP haplotype marker-based genotyping information produced a higher LD estimate and resolution covering the entire gene. (D) The differential expression profiling of *OsMED4*_2 gene in five seed developmental stages (S1–S5) and flag leaves (Lf) of six contrasting low (Sonasal and Bindli) and high (LGR, PusaBasmati 1121, Nipponbare and IR 64) grain weight rice accessions. E1: Exon1, E2: Exon2, I: Intron, URR: upstream regulatory region and DRR: downstream regulatory region.

The grain size-associated ten SNPs-containing *MED* genes (identified by candidate gene-based association analysis), including one validated in bi-parental mapping population, were assayed for differential expression profiling to access the functional regulatory pattern of these genes in controlling grain size/weight of rice. The flag leaves and five seed developmental stages (S1 to S5) of two low (Sonasal and Bindli) and four high/medium (Pusa Basmati 1121, IR 64, Nipponbare and LGR) grain weight rice accessions were utilized for quantitative RT-PCR assay (Fig. 4). Of these, regulatory, intronic and non-synonymous SNPs-containing nine genes (except *OsMED11_1*) were ≥2 fold differentially regulated in at least one of the five seed developmental stages as compared to flag leaf in all the six rice accessions under study (Figure S3). Out of these nine genes, non-synonymous SNPs-containing four genes (*OsMED4_2, OsMED12_2, OsMED15_1* and *OsMED37_3*) exhibited very high expression in seed stages (>7 fold upregulation in at least one of the five seed development stages as compared to flag leaf) of at least two varieties and among these, *OsMED4*_2 showed seed-specific expression (Fig. 4, S3, Table S4). Remarkably, one non-synonymous SNP (G/A)-carrying seed-specific *OsMED4_2* validated by both genetic association analysis and in mapping population revealed almost an inversely correlated differential expression pattern in seed developmental stages of some of the selected low and high grain weight rice accessions (Fig. 4, S3, Table S4). A decreased expression of *OsMED4_2* gene in the initial three seed developmental stages (S1, S2 and S3) of high (Pusa Basmati 1121, IR 64 and LGR) and increased expression in low (Sonasal) grain weight rice accessions than that of flag leaves of respective accessions was observed (Figs 4 and 5D, S3, Table S4). A pronounced higher expression of *OsMED4_2* in S4 and S5 seed developmental stages of both high (IR 64 and Pusa Basmati 1121) and low (Sonasal) grain weight rice accessions was also apparent (Figs 4 and 5D, S3, Table S4). Interestingly, *OsMED4_2* with non-synonymous SNPs validated in high and low grain weight parental accessions of a mapping population (IR 64 × Sonasal), exhibited differential regulation pattern in these accessions during seed development, implicating functional significance of this gene in grain weight regulation of rice. It would be thus interesting to constitute gene-specific haplotypes by targeting/combining other novel coding and non-coding SNP allelic variants mined from this *OsMED4_2* gene and determine trait association potential of the gene haplotypes with grain size/weight variation in naturally occurring rice accessions.

### Molecular haplotyping in a grain size-associated *MED* gene

For molecular haplotyping of a strong grain size-associated *OsMED4_2* gene (validated by association analysis, expression profiling and in mapping population), the cloned PCR amplicon sequencing and Illumina targeted multiplex-gene amplicon resequencing of entire 2 kb URR, exons, 1 kb DRR and intronic region of target gene in 384 rice accessions were performed (Fig. 5A). This discovered 17 SNPs from diverse coding and non-coding (including three non-synonymous, seven intronic and two URR SNPs) sequence components of the gene. The haplotype analysis in *OsMED4_2* gene, by deploying the genotyping data of 17 SNPs among 384 rice accessions, constituted overall three haplotypes (Fig. 5B). The three SNP haplotype-based LD mapping in *OsMED4_2* gene exhibited a higher degree of LD (r^2^ > 0.90 with P < 1.0 × 10^−7^) resolution in this gene (Fig. 5C). The association analysis using *OsMED4_2* gene-derived SNP haplotypes demonstrated its strong association potential (PVE: 44% with P: 1.1 × 10^−10^) for grain size/weight trait variation. Remarkably, two major haplotypes of *OsMED4_2* gene differentiated by a functional non-synonymous coding SNP (G/A) revealed strong association potential for low/medium (haplotypes I and III) and high grain weight (haplotype II) differentiation in rice (Fig. 5B). Nevertheless, novel haplotypes (with diverse allelic recombination) in an *OsMED4_2* gene exhibiting differential trait association potential for rice grain size/weight were identified by SNP-based high-resolution molecular haplotyping. Altogether, a higher association potential of *OsMED4_2* gene with grain size/weight trait variation in rice was ascertained by their combined validation through candidate gene-based association analysis, in mapping population, differential expression profiling and high-resolution molecular haplotyping/LD mapping. The grain size/weight is a complex quantitative trait and controlled by a complex regulatory networks involving a diverse arrays of genes in rice^1^,^2^. A number of known genes underlying QTLs governing grain length, grain width and grain weight have been cloned and characterized so far in rice^4^,^6^,^7^,^8^,^9^,^10^,^11^,^12^,^13^,^14^. In spite of several major efforts, no such potential robust genes/QTLs (validated in multiple genetic backgrounds/environments) have been identified till date to be deployed in marker-assisted breeding for selecting accessions with high grain weight and yield in rice. In the present study, efforts have been made to integrate candidate gene-based association analysis with mapping population validation, differential gene expression profiling and gene-based molecular haplotyping/LD mapping effectively, which enabled to delineate diverse natural SNP allelic variants in 10 *MED* genes, including three novel haplotypes in *OsMED4_2* gene regulating grain weight/size differentiation in rice (Figure S4).

The involvement of *OsMED4* gene in transcriptional regulation by its effective interaction with other protein-coding genes and signalling pathways underlying various aspects of plant development and growth has been deciphered recently^64^. MED4 is a subunit in the Middle module of the complex. Just like yeast and mammalian MED4, *Arabidopsis* MED4 interacts with MED9 and thus appears to be an important component for integrity of Middle module structure^32^. On the basis of very high sequence homology between *Arabidopsis* and rice MED4, it can be postulated that OsMED4 might be interacting with OsMED9. MED4 has two IDRs, one at each terminal, separated by a region which is predicted to be helical in nature. In yeast, a fragment harbouring this helical region and the C-terminal IDR was found to be important in the interaction of MED4 with MED7, MED9, MED10 and MED21^65^. The non-synonymous SNP (G/A) was found to be present in the CDS sequence corresponding to this helical region of OsMED4_2. Interestingly, in one of the earlier study, this region emerged as a signature motif for MED4 suggesting its importance in MED4 functioning^66^. This part of MED4 might thus be important in rice for maintaining the integrity of the Middle module. MED4 is a very disordered protein with a strong tendency to interact with other proteins^32^. In *Arabidopsis*, AtMED4 interacts with more than hundred proteins, including a couple of transcription factors like WOX13 and UNE12 that play important role in seed development and maturation^32^. WOX13 controls medio-lateral patterning of the fruit, which is the basis for seed maturation and dispersal^67^. On the other hand, mutation in UNE12 shows defect in embryo sac functions such as pollen tube guidance or fertilization^68^. There is a possibility that in rice also, MED4 is targeted by orthologs of WOX13 and UNE12 for their function. So any variation in the important residues of MED4 that disrupts its interaction with such transcription factors (WOX13 or UNE12) or other Mediator subunits (MED7, MED9, MED10 or MED21) can exhibit effect on the process of fertilization, seed setting, development and maturation. Such possible transcriptional mechanism of trait regulation due to non-synonymous SNP substitutions in the CDS of genes encoding variable amino acid residues and altered secondary structure of proteins has already been demonstrated in multiple known cloned grain size genes of rice^2^. It will be interesting to expand the SNP analysis in a larger set of diverse rice varieties to the whole genome level to decipher the genetic network significantly associated with rice grain size/weight and then see if *OsMED4_2* is a part of the network. Thus, the grain size/weight-associated functionally relevant molecular tags (alleles and haplotypes) identified in the *MED* genes using a combinatorial genomic approach can be useful for rapid quantitative dissection of complex grain size/weight trait and eventually in marker-assisted breeding to develop improved rice cultivars with high grain weight and yield.

## Methods

### Targeted multiplex-gene amplicon resequencing

The genomic DNA was isolated from the young leaves of 384 low and high grain weight diverse rice accessions using QIAGEN DNeasy96 Plant Kit (QIAGEN, USA) according to the manufacturer’s protocol. For mining and genotyping of gene-based SNPs, a set of 55 *MED* genes structurally and functionally annotated from whole rice genome^34^ were utilized. These selected genes were resequenced using the genomic DNA of 384 rice accessions employing the multiplexed amplicon resequencing method (TruSeq Custom Amplicon v1.5) of Illumina MiSeq next-generation sequencer (Illumina, USA). The CDS (coding sequences)/exons, introns, 2000-bp URRs (upstream regulatory regions) and 1000-bp DRRs (downstream regulatory regions) of 55 *MED* genes were targeted for designing and synthesizing the custom oligo probes using Design Studio software (Illumina, USA). All the probes were pooled into a custom amplicon tube to produce amplicons with an average size of 400 bp per reaction and template library was made using TruSeq Custom Amplicon Assay kit v1.5. The sample-specific indices were added to each library by PCR using common primers from the TruSeq Amplicon Index kit. The normalization of the uniquely tagged pooled amplicon libraries was performed and the generated clusters were sequenced by Illumina MiSeq platform. Illumina Amplicon Viewer was used to visualize the sequenced amplicons and sequence variants. The high-quality gene amplicons sequence reads of each accession were mapped to the pseudomolecules of reference Nipponbare rice genome (MSU, http://rice.plantbiology.msu.edu, Release 7.0) and non-erroneous high-quality SNPs were detected among accessions following methods of Saxena *et al*.^57^ and Kujur *et al*.^69^.

To ascertain the reliability and accuracy of identified SNPs, the genomic DNA of 24 rice accessions (selected from 384 low and high grain weight accessions) were PCR amplified with 55 *MED* gene-specific primers. The amplified PCR products were sequenced by automated 96 capillary ABI 3730xl DNA Analyzer (Applied Biosystems, USA). Subsequently, the high-quality gene sequences were aligned and compared to discover SNPs among accessions as per Saxena *et al*.^70^.

### Association mapping

For phenotyping, 384 diverse rice accessions belonging to an association panel were grown in the field (as per randomised complete block design with two replications) for two consecutive years (2012 and 2013) during crop growing season at two diverse geographical locations (New Delhi-latitude 28°4′ N and longitude 77.1′ E and Tamil Nadu-11° N and 78 °E) of India. The accessions were phenotyped with replications for grain length (mm), grain width (mm) and grain weight (g) by measuring the weight of 1000 mature dried grains (at 10% moisture content) selected from 10–15 representative plants of each accession. The diverse statistical parameters, including frequency distribution, coefficient of variation (CV) and broad-sense heritability (H^2^) of grain size (grain length, width and weight) traits among accessions were estimated using SPSSv17.0 as per Bajaj *et al*.^71^. The determination of population genetic structure, PCA and LD decay among accessions using *MED* gene-derived SNPs was performed following Kujur *et al*.^56^.

For association mapping, the grain length, grain width and 1000-grain weight phenotypic and *MED* gene-based SNP genotyping information (5% MAF) as well as population structure ancestry coefficient (Q matrix), kinship matrix (K) and PCA (P) data of 384 rice accessions were integrated. MAF using the SNP genotyping data was measured using TASSEL v5.0 (http://www.maizegenetics.net/#!tassel/c17q9). Association analysis was performed using CMLM (compressed mixed linear model) and P3D (population parameters previously determined)/EMMAX (efficient mixed model association eXpedited) model-based approach of GAPIT as per Kujur *et al*.^56^ and Kumar *et al*.^61^. To ensure the accuracy of association outcomes, the relative distribution of observed and expected -log_10_(P)-value of each SNP marker-trait association was compared individually with their quantile-quantile plots. According to false discovery rate (FDR cut-off ≤0.05), the adjusted P-value threshold of significance was corrected for multiple comparisons. The potential SNP loci in the diverse coding and non-coding sequence components of *MED* genes revealing significant association with grain length, grain width and grain weight trait at a highest R^2^ (degree of SNP marker-trait association) and lowest FDR adjusted P-values (threshold P ≤ 10^−5^) were selected.

### Validation of associated SNPs in a mapping population

To ascertain the potential of *MED* gene-derived SNPs for grain length, grain width and grain weight association, the trait-associated SNPs were selected to validate in a traditional bi-parental mapping population. For this, 10 of each low (Sonasal with 1000-grain weight: 10 g) and high (IR 64: 25 g) grain weight homozygous individuals derived from a F_4_ mapping population (IR 64 × Sonasal) along with parental accessions were selected for DNA isolation. The grain size/weight-associated SNPs exhibiting polymorphism between the mapping parents were genotyped in the selected 20 homozygous low and high grain weight mapping individuals using MALDI-TOF mass array SNP genotyping assay following Saxena *et al*.^57^,^70^. The correspondence of low and high grain size/weight-associated SNPs with their presence in the low and high grain weight homozygous mapping individuals was determined to validate the grain size/weight trait association potential of *MED* gene-derived SNPs.

### Differential expression profiling

To determine the regulatory pattern of genes associated (validated by association analysis and in mapping population) with grain size/weight, the differential expression profiling of these genes was performed using the quantitative RT-PCR assay. The total RNA was isolated from three biological replicates of flag leaf (considered as control) and five different seed developmental stages (defined as per Agarwal *et al*.^72^ and Sharma *et al*.^73^) of four high/medium (Pusa Basmati 1121, IR 64, Nipponbare and LGR) and two low (Sonasal and Bindli) grain weight rice accessions as previously described^74^. The purified RNA was tested for quality by denaturing agarose gel electrophoresis and NANODROP 2000 Spectrophotometer (Thermo Scientific, NanoDrop products, USA). One μg of high quality total RNA was used for cDNA synthesis using first strand cDNA synthesis kit (Applied Biosystems, USA). The cDNA (1:100 dilution) along with 1X Fast SYBR Green Master Mix (Applied Biosystems) and 200 nM of forward and reverse gene-specific primers (Table S5) in a total reaction volume of 10 μl was amplified in quantitative RT-PCR assay by ViiA™ 7 Real-Time PCR system (Applied Biosystems). The normalization and differential expression were calculated as reported previously^75^.

### Molecular haplotyping

For gene-based SNP haplotyping, the 2 kb URR, exon, intron and 1 kb DRR of grain weight-regulating candidate *MED* gene (validated by association analysis, in mapping population and expression profiling), amplified from 384 rice accessions (association panel) were cloned and sequenced as per Kujur *et al*.^55^ and Saxena *et al*.^70^. The high-quality *MED* gene sequences were aligned among accessions using the CLUSTALW multiple sequence alignment tool of MEGA v6.0^76^ and SNPs in the genes were discovered. The genotyping data of *MED* gene-derived SNPs generated by cloned PCR amplicon sequencing and aforesaid Illumina targeted multiplex-gene amplicon resequencing among accessions, was used to constitute haplotypes within the gene. For gene haplotype-based association analysis, the SNP haplotype genotyping information in the *MED* gene was further correlated with 1000-grain weight phenotyping data of 384 rice accessions using aforementioned genetic association analysis strategy.

## Additional Information

**How to cite this article**: Malik, N. *et al*. An Integrated Genomic Strategy Delineates Candidate Mediator Genes Regulating Grain Size and Weight in Rice. *Sci. Rep.* **6**, 23253; doi: 10.1038/srep23253 (2016).

## Supplementary Material

Supplementary Information

## Figures and Tables

**Table 1:** Genomic distribution of SNPs mined from rice Mediator genes.

Ricechromosomes	Mediator genesannotated	Number (%) ofgenic SNPsdiscovered	Regulatory-SNPs	Intronic-SNPs	**CDS-SNPs**	
					**Non-synonymous**	**Synonymous**
*Os_chr01*	4	484 (12.2)	84	376	9	15
*Os_chr02*	6	328 (8.3)	160	121	17	30
*Os_chr03*	5	286 (7.2)	155	94	5	32
*Os_chr04*	4	504 (12.7)	166	185	92	61
*Os_chr05*	6	370 (9.3)	170	147	19	34
*Os_chr06*	1	73 (1.8)	51	20	0	2
*Os_chr07*	4	263 (6.6)	108	99	26	30
*Os_chr08*	5	327 (8.2)	129	131	36	31
*Os_chr09*	6	568 (14.3)	192	289	39	48
*Os_chr10*	7	324 (8.2)	127	126	48	23
*Os_chr11*	4	332 (8.4)	133	150	24	25
*Os_chr12*	3	112 (2.8)	70	23	8	11
**Total**	**55**	**3971**	**1545 (46.7)**	**1761 (53.3)**	**323 (48.6)**	**342 (51.4)**

Parentheses indicate the proportion of each kind of SNP mined from its respective total *MED* gene-derived SNPs.

**Table 2:** SNPs identified in the *MED* genes associated with grain size traits in rice.

***OsMED*****genes**	MSU locusIDs	**Chromosomes**	AssociatedSNPs	Physicalpositions(bp)	Minor allelefrequency(MAF %)	Functionalsignificanceof SNPs	Encoded aminoacid	**P-value**	PVE(R^**2**^**%)**	Associated grainsize traits
*OsMED9_1*	LOC_Os01g31629	*Oschr01*	(C/G)	17320769	17	Intron	–	1.7 × 10^−5^	21	GWg
*OsMED11_1*	LOC_Os02g09600	*Oschr*02	(G/A)	4940189	15	Non-Synonymous coding	Threonine (ACG)/Methionine (ATG)	1.7 × 10^−6^	25	GL and GWg
*OsMED37_3*	LOC_Os03g16860	*Oschr*03	(A/G)	9369204	16	URR	–	1.1 × 10^−5^	18	GWi and GWg
*OsMED15_1*	LOC_Os04g03860	*Oschr*04	(G/A)	1754571	18	Non-Synonymous coding	Glycine(GGG)/Arginine (AGG)	0.8 × 10^−5^	17	GL and GWg
*OsMED5_3*	LOC_Os07g48350	*Oschr*07	(G/A)	28899486	20	Non-Synonymous coding	Alanine (GCG)/Threonine (ACG)	1.5 × 10^−5^	20	GWg and GWi
*OsMED14_1*	LOC_Os08g24400	*Oschr*08	(G/T)	14736149	18	URR	–	2.1 × 10^−5^	15	GL and GWg
*OsMED25_1*	LOC_Os09g13610	*Oschr*09	(T/A)	7912591	26	URR	–	1.3 × 10^−6^	28	GL and GWg
*OsMED20_1*	LOC_Os09g27140	*Oschr*09	(A/G)	16507513	21	Non-Synonymous coding	Phenylalanine (TTC)/Leucine (CTC)	0.9 × 10^−7^	26	GL, GWi and GWg
*OsMED12_2*	LOC_Os10g40260	*Oschr* 10	(G/C)	21503285	22	Non-Synonymous coding	Valine (GTT)/Leucine (CTT)	1.1 × 10^−5^	24	GL and GWg
*OsMED4_2*	LOC_Os11g05150	*Oschr*11	(C/T)	2260577	24	Non-Synonymous coding	Glutamic acid (GAG)/Lysine(AAG)	0.3 × 10^−8^	33	GL, GWi and GWg

GL: Grain Length, GWi: Grain Width and GWg: 1000-Grain Weight, PVE: Phenotypic Variation Explained and URR: Upstream Regulatory Region.
