# Supplementary material for: An Integrated Genomic Strategy Delineates Candidate Mediator Genes Regulating Grain Size and Weight in Rice
Source: Sci Rep. 2016 Mar 22;6:23253. doi: 10.1038/srep23253 (PMC4802383; doi:10.1038/srep23253)
Supplement: Supplementary Information [file srep23253-s1.pdf]

An Integrated Genomic Strategy Delineates Candidate Mediator Genes Regulating Grain Size and Weight in Rice

Naveen Malik[1*], Nidhi Dwivedi[1*], Ashok K. Singh[2], Swarup K. Parida[1], Pinky Agarwal[1], Jitendra K. Thakur[1] & Akhilesh K. Tyagi[1]

[1]National Institute of Plant Genome Research (NIPGR), Aruna Asaf Ali Marg, New Delhi 110067, India

[2]Division of Genetics, Rice Section, Indian Agricultural Research Institute (IARI), New Delhi 110012, India

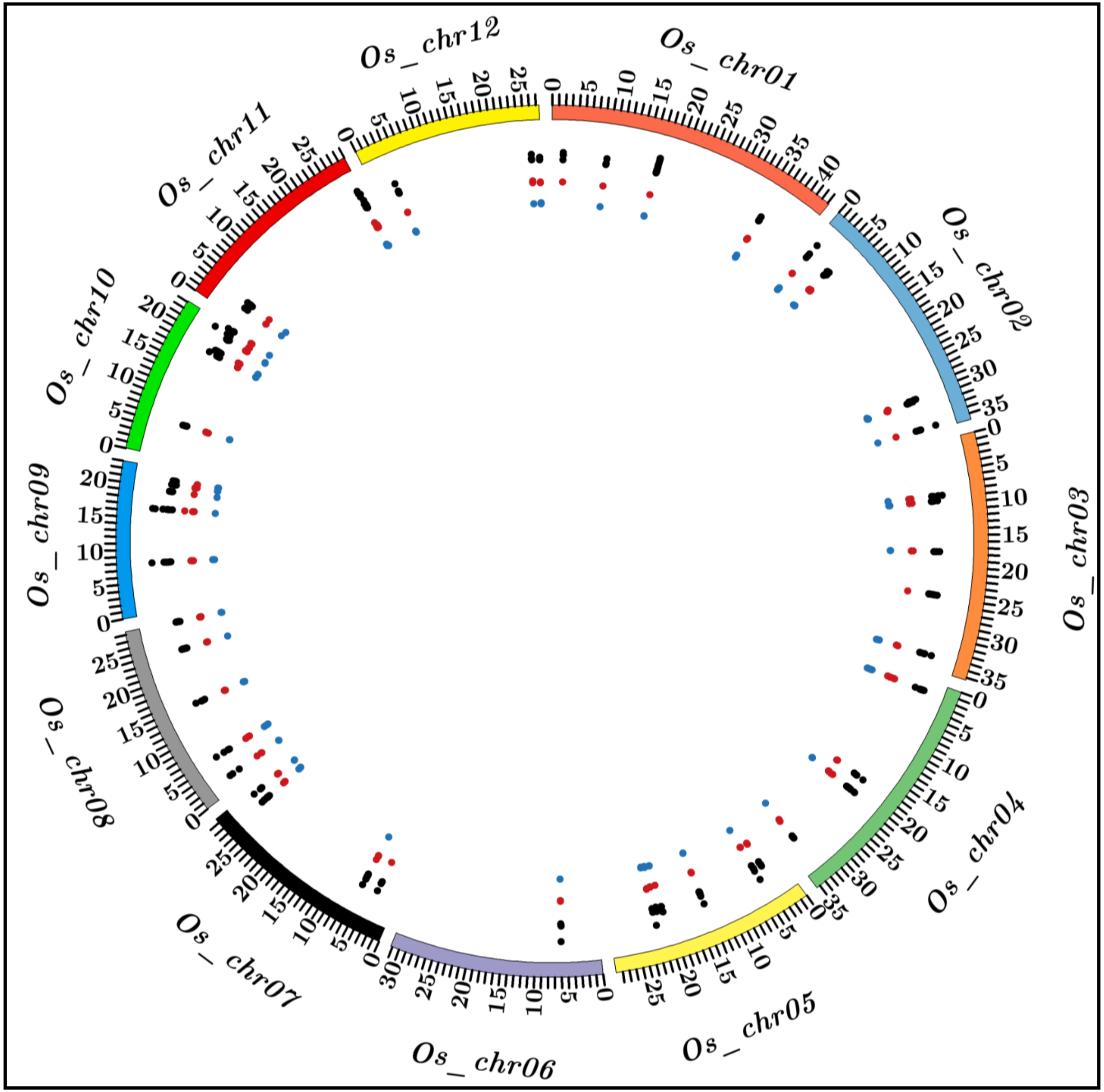

Figure S1. The relative distribution of 3971 *MED* genes-derived SNPs physically mapped on 12 rice chromosomes is illustrated by a Circos circular ideogram. The outermost circle indicates the physical size (Mb) of 12 rice chromosome-pseudomolecules coded with multiple colours. The black (all SNPs), red (non-synonymous and regulatory SNPs) and blue (synonymous SNPs) dots represent different types of coding and non-coding SNPs mined from the 55 *MED* genes.

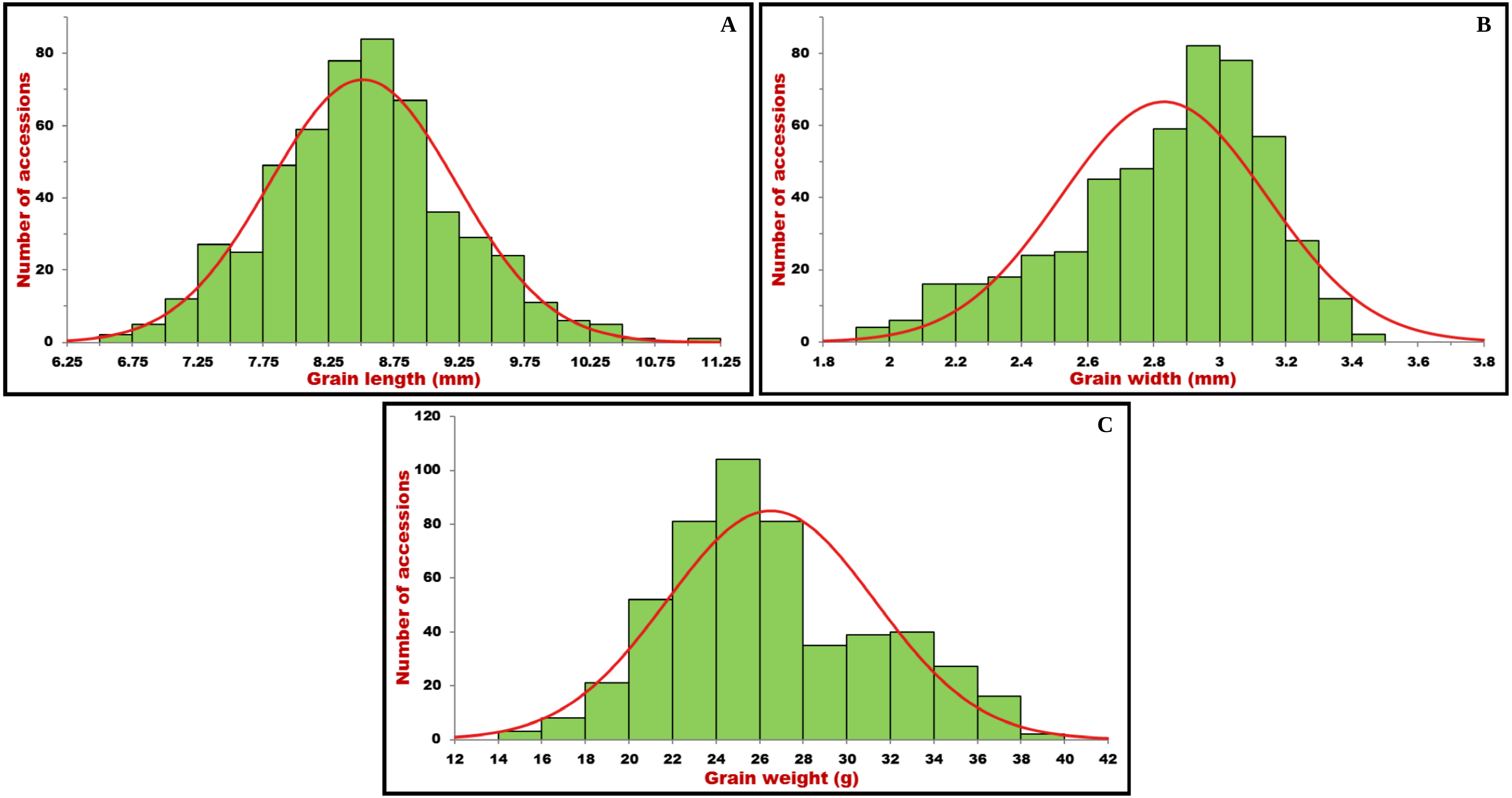

Figure S2. Frequency distribution of grain length (mm) (A), grain width (mm) (B) and 1000-grain weight (g) (C) trait variations among 384 rice accessions (association panel) depicting a normal distribution.

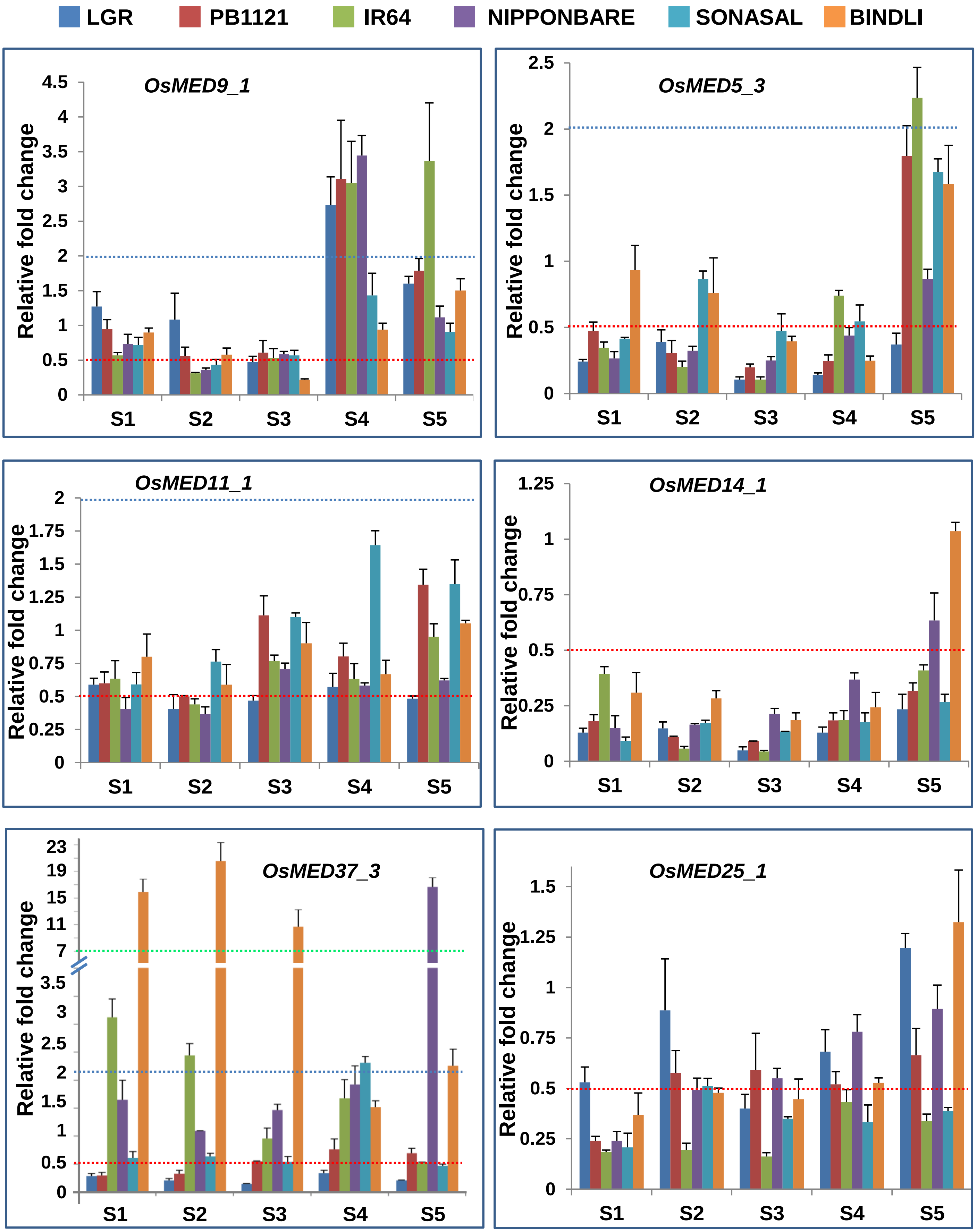

LGR
PB1121
IR64
NIPPONBARE
SONASAL
BINDLI
OsMED9_1
OsMED5_3
OsMED11_1
OsMED14_1
OsMED37_3
OsMED25_1
Relative fold change
S1
S2
S3
S4
S5

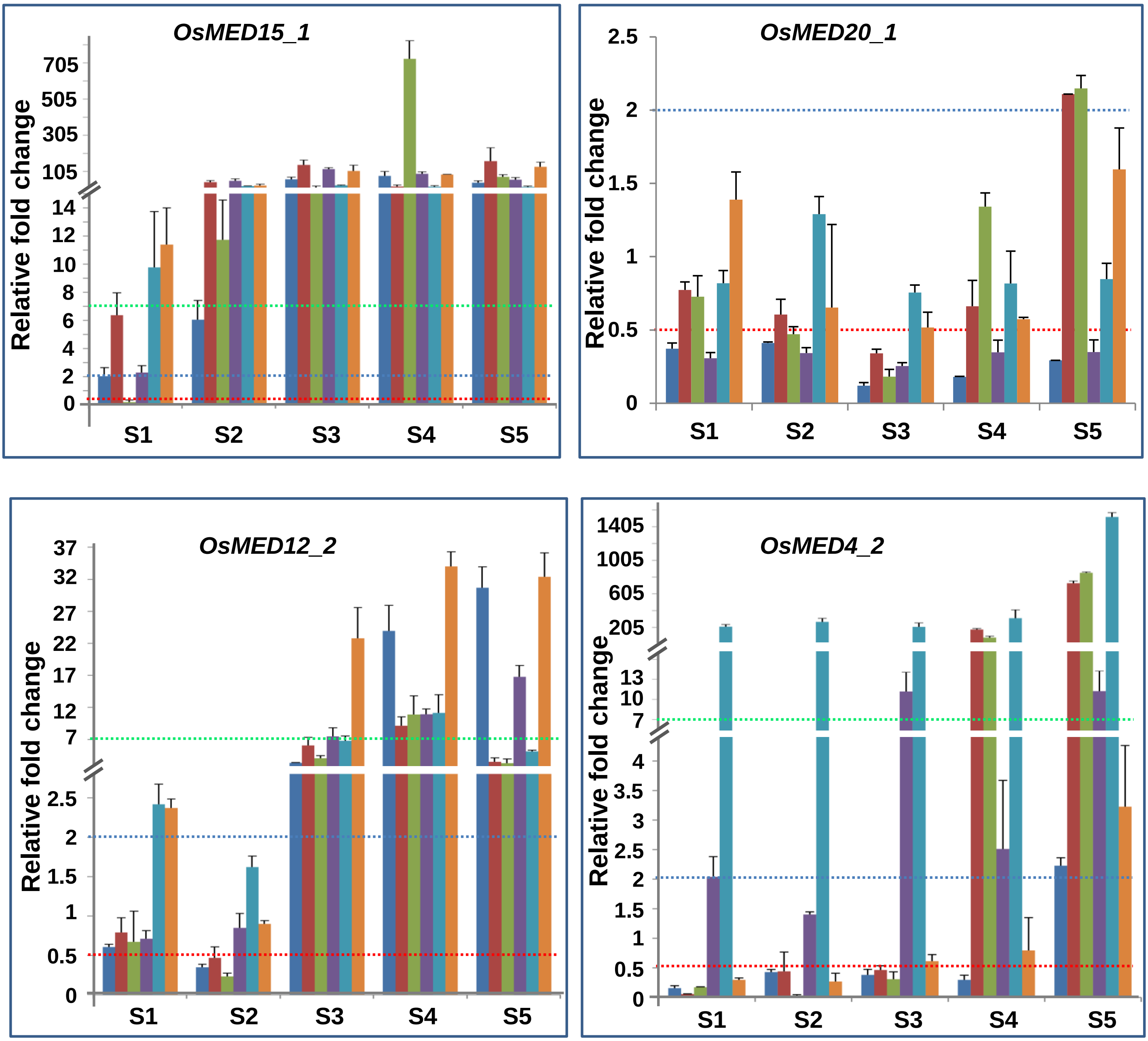

Figure S3: Graphs showing fold changes in seed w.r.t. flag leaf in selected *MED* genes calculated by $2^{-\Delta\Delta Ct}$ method. Graph bars above/below dotted lines represent ≥ 2 fold upregulation (above blue dotted lines), ≥ 2 fold downregulation (below red dotted lines) and ≥ 7 fold upregulation (above green dotted lines). Note the change in scale on y-axis after the break in the graphs. The seed development stages (S1-S5) are depicted on the x-axis. QPCR was performed in six accessions of rice, as mentioned in the legend. The SD values for three biological replicates have been plotted in the bars.

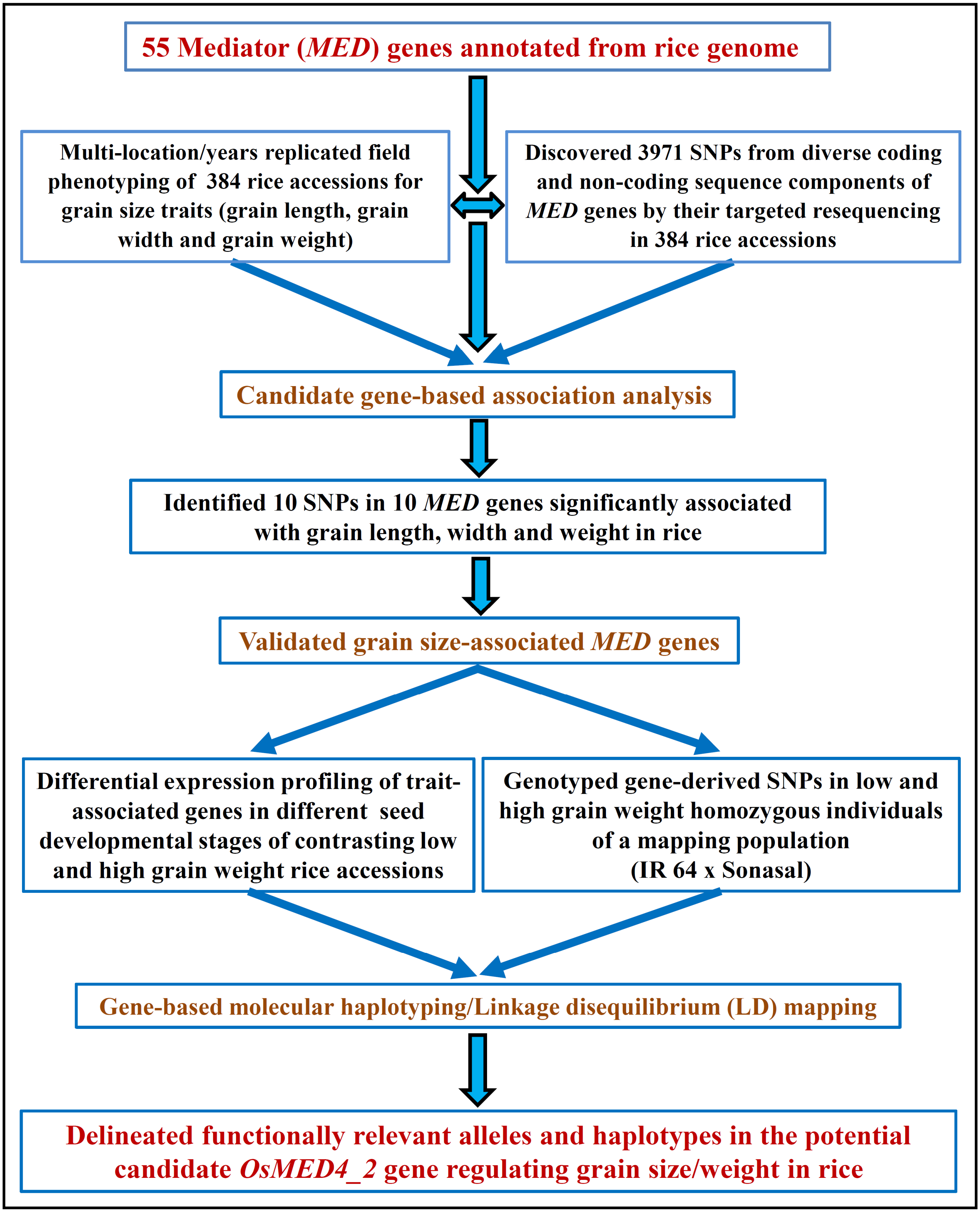

Figure S4. An integrated genomic approach (trait association analysis and mapping population validation, differential expression profiling, molecular haplotyping and SNP haplotype-specific association analysis/LD mapping) to delineate the potential molecular tags in a candidate *MED* gene regulating grain weight/size in rice.

Table S1. Structural and functional annotation of 3971 SNPs mined from the 55 Mediator genes of rice

**Mediator genes**	**MSU locus ID**	**Chromosomes**	**Physical Position (bp)**	**SNPs**	**Structural Annotation**
OsMed2_1	LOC_Os10g40060	Os_chr10	21387939	[A/G]	INTRON
OsMed2_1	LOC_Os10g40060	Os_chr10	21387849	[A/G]	INTRON
OsMed2_1	LOC_Os10g40060	Os_chr10	21387565	[G/A]	INTRON
OsMed2_1	LOC_Os10g40060	Os_chr10	21388461	[C/T]	REGULATORY
OsMed2_1	LOC_Os10g40060	Os_chr10	21388445	[G/T]	REGULATORY
OsMed2_1	LOC_Os10g40060	Os_chr10	21388400	[A/G]	REGULATORY
OsMed2_1	LOC_Os10g40060	Os_chr10	21388371	[A/T]	REGULATORY
OsMed2_1	LOC_Os10g40060	Os_chr10	21388323	[C/A]	REGULATORY
OsMed2_1	LOC_Os10g40070	Os_chr10	21388970	[T/C]	SYNONYMOUS-CODING
OsCdk8_1	LOC_Os10g42950	Os_chr10	23080890	[A/T]	INTRON
OsCdk8_1	LOC_Os10g42950	Os_chr10	23080752	[G/A]	INTRON
OsCdk8_1	LOC_Os10g42950	Os_chr10	23080416	[G/A]	INTRON
OsCdk8_1	LOC_Os10g42950	Os_chr10	23079089	[A/G]	INTRON
OsCdk8_1	LOC_Os10g42950	Os_chr10	23078576	[C/A]	INTRON
OsCdk8_1	LOC_Os10g42950	Os_chr10	23078043	[C/T]	INTRON
OsCdk8_1	LOC_Os10g42950	Os_chr10	23077940	[C/A]	INTRON
OsCdk8_1	LOC_Os10g42950	Os_chr10	23079876	[G/A]	INTRON
OsCdk8_1	LOC_Os10g42950	Os_chr10	23081729	[G/A]	REGULATORY
OsCdk8_1	LOC_Os10g42950	Os_chr10	23077501	[T/C]	REGULATORY
OsCdk8_1	LOC_Os10g42950	Os_chr10	23077448	[C/T]	REGULATORY
OsCdk8_1	LOC_Os10g42950	Os_chr10	23077428	[A/C]	REGULATORY
OsCdk8_1	LOC_Os10g42950	Os_chr10	23077379	[C/T]	REGULATORY
OsCdk8_1	LOC_Os10g42950	Os_chr10	23082694	[G/A]	REGULATORY
OsCdk8_1	LOC_Os10g42950	Os_chr10	23082624	[A/G]	REGULATORY
OsCdk8_1	LOC_Os10g42950	Os_chr10	23082609	[T/A]	REGULATORY
OsCdk8_1	LOC_Os10g42950	Os_chr10	23082601	[G/T]	REGULATORY
OsCdk8_1	LOC_Os10g42950	Os_chr10	23082584	[A/G]	REGULATORY
OsCdk8_1	LOC_Os10g42950	Os_chr10	23082506	[C/T]	REGULATORY
OsCdk8_1	LOC_Os10g42950	Os_chr10	23082491	[T/C]	REGULATORY
OsCdk8_1	LOC_Os10g42950	Os_chr10	23082474	[C/G]	REGULATORY
OsCdk8_1	LOC_Os10g42950	Os_chr10	23082457	[T/C]	REGULATORY
OsCdk8_1	LOC_Os10g42950	Os_chr10	23082426	[C/T]	REGULATORY
OsCdk8_1	LOC_Os10g42950	Os_chr10	23082301	[T/C]	REGULATORY
OsCdk8_1	LOC_Os10g42950	Os_chr10	23082186	[C/T]	REGULATORY
OsCdk8_1	LOC_Os10g42950	Os_chr10	23082161	[A/T]	REGULATORY
OsCdk8_1	LOC_Os10g42950	Os_chr10	23082114	[G/A]	REGULATORY
OsCdk8_1	LOC_Os10g42950	Os_chr10	23082059	[G/A]	REGULATORY
OsCdk8_1	LOC_Os10g42950	Os_chr10	23081982	[G/A]	REGULATORY
OsCdk8_1	LOC_Os10g42950	Os_chr10	23081980	[T/C]	REGULATORY
OsCdk8_1	LOC_Os10g42950	Os_chr10	23081819	[A/G]	REGULATORY
OsCdk8_1	LOC_Os10g42950	Os_chr10	23079675	[C/T]	SYNONYMOUS-CODING
OsCdk8_1	LOC_Os10g42950	Os_chr10	23079512	[G/A]	SYNONYMOUS-CODING
OsCycC_1	LOC_Os09g32680	Os_chr09	19502176	[G/A]	INTRON
OsCycC_1	LOC_Os09g32680	Os_chr09	19501982	[C/T]	INTRON
OsCycC_1	LOC_Os09g32680	Os_chr09	19501753	[T/C]	INTRON
OsCycC_1	LOC_Os09g32680	Os_chr09	19501717	[A/C]	INTRON

**Mediator genes**	**MSU locus ID**	**Chromosomes**	**Physical Position (bp)**	**SNPs**	**Structural Annotation**
OsCycC_1	LOC_Os09g32680	Os_chr09	19501705	[G/A]	INTRON
OsCycC_1	LOC_Os09g32680	Os_chr09	19501621	[A/G]	INTRON
OsCycC_1	LOC_Os09g32680	Os_chr09	19501434	[A/G]	INTRON
OsCycC_1	LOC_Os09g32680	Os_chr09	19501278	[A/C]	INTRON
OsCycC_1	LOC_Os09g32680	Os_chr09	19501260	[C/T]	INTRON
OsCycC_1	LOC_Os09g32680	Os_chr09	19501249	[T/C]	INTRON
OsCycC_1	LOC_Os09g32680	Os_chr09	19501014	[C/A]	INTRON
OsCycC_1	LOC_Os09g32680	Os_chr09	19501006	[A/G]	INTRON
OsCycC_1	LOC_Os09g32680	Os_chr09	19500945	[A/T]	INTRON
OsCycC_1	LOC_Os09g32680	Os_chr09	19500844	[A/T]	INTRON
OsCycC_1	LOC_Os09g32680	Os_chr09	19500826	[A/G]	INTRON
OsCycC_1	LOC_Os09g32680	Os_chr09	19500783	[C/T]	INTRON
OsCycC_1	LOC_Os09g32680	Os_chr09	19500671	[A/T]	INTRON
OsCycC_1	LOC_Os09g32680	Os_chr09	19500312	[G/T]	INTRON
OsCycC_1	LOC_Os09g32680	Os_chr09	19500298	[G/A]	INTRON
OsCycC_1	LOC_Os09g32680	Os_chr09	19500286	[T/C]	INTRON
OsCycC_1	LOC_Os09g32680	Os_chr09	19500283	[T/G]	INTRON
OsCycC_1	LOC_Os09g32680	Os_chr09	19500255	[A/G]	INTRON
OsCycC_1	LOC_Os09g32680	Os_chr09	19500144	[A/T]	INTRON
OsCycC_1	LOC_Os09g32680	Os_chr09	19500018	[A/G]	INTRON
OsCycC_1	LOC_Os09g32680	Os_chr09	19499783	[T/C]	INTRON
OsCycC_1	LOC_Os09g32680	Os_chr09	19499760	[T/C]	INTRON
OsCycC_1	LOC_Os09g32680	Os_chr09	19499607	[G/A]	INTRON
OsCycC_1	LOC_Os09g32680	Os_chr09	19499428	[C/G]	INTRON
OsCycC_1	LOC_Os09g32680	Os_chr09	19499215	[A/T]	INTRON
OsCycC_1	LOC_Os09g32680	Os_chr09	19499172	[A/G]	INTRON
OsCycC_1	LOC_Os09g32680	Os_chr09	19499071	[T/C]	INTRON
OsCycC_1	LOC_Os09g32680	Os_chr09	19498894	[C/T]	INTRON
OsCycC_1	LOC_Os09g32680	Os_chr09	19498845	[G/A]	INTRON
OsCycC_1	LOC_Os09g32680	Os_chr09	19502717	[A/G]	REGULATORY
OsCycC_1	LOC_Os09g32680	Os_chr09	19502704	[G/C]	REGULATORY
OsCycC_1	LOC_Os09g32680	Os_chr09	19502605	[C/T]	REGULATORY
OsCycC_1	LOC_Os09g32680	Os_chr09	19502478	[A/T]	REGULATORY
OsCycC_1	LOC_Os09g32680	Os_chr09	19502434	[A/G]	REGULATORY
OsCycC_1	LOC_Os09g32680	Os_chr09	19498283	[G/A]	REGULATORY
OsCycC_1	LOC_Os09g32680	Os_chr09	19498263	[G/T]	REGULATORY
OsCycC_1	LOC_Os09g32680	Os_chr09	19498261	[C/T]	REGULATORY
OsCycC_1	LOC_Os09g32680	Os_chr09	19498238	[A/C]	REGULATORY
OsCycC_1	LOC_Os09g32680	Os_chr09	19498227	[C/A]	REGULATORY
OsCycC_1	LOC_Os09g32680	Os_chr09	19498051	[T/C]	REGULATORY
OsCycC_1	LOC_Os09g32680	Os_chr09	19497977	[A/T]	REGULATORY
OsCycC_1	LOC_Os09g32680	Os_chr09	19497928	[G/A]	REGULATORY
OsCycC_1	LOC_Os09g32680	Os_chr09	19497818	[A/T]	REGULATORY
OsCycC_1	LOC_Os09g32680	Os_chr09	19497697	[A/G]	REGULATORY
OsCycC_1	LOC_Os09g32680	Os_chr09	19497664	[A/G]	REGULATORY
OsCycC_1	LOC_Os09g32680	Os_chr09	19497598	[G/A]	REGULATORY
OsCycC_1	LOC_Os09g32680	Os_chr09	19502062	[A/T]	SYNONYMOUS-CODING

**Mediator genes**	**MSU locus ID**	**Chromosomes**	**Physical Position (bp)**	**SNPs**	**Structural Annotation**
OsCycC_1	LOC_Os09g32680	Os_chr09	19501136	[A/C]	SYNONYMOUS-CODING
OsMed10_1	LOC_Os09g35920	Os_chr09	20677468	[G/C]	INTRON
OsMed10_1	LOC_Os09g35920	Os_chr09	20677223	[A/G]	INTRON
OsMed10_1	LOC_Os09g35920	Os_chr09	20676655	[C/T]	INTRON
OsMed10_1	LOC_Os09g35920	Os_chr09	20676536	[T/A]	INTRON
OsMed10_1	LOC_Os09g35920	Os_chr09	20676375	[T/C]	INTRON
OsMed10_1	LOC_Os09g35920	Os_chr09	20676302	[C/A]	INTRON
OsMed10_1	LOC_Os09g35920	Os_chr09	20676240	[C/T]	NON-SYNONYMOUS-CODING
OsMed10_1	LOC_Os09g35920	Os_chr09	20675815	[C/A]	REGULATORY
OsMed10_1	LOC_Os09g35920	Os_chr09	20675607	[C/T]	REGULATORY
OsMed10_1	LOC_Os09g35920	Os_chr09	20675399	[C/T]	REGULATORY
OsMed10_1	LOC_Os09g35920	Os_chr09	20675216	[G/T]	REGULATORY
OsMed10_1	LOC_Os09g35920	Os_chr09	20675155	[T/C]	REGULATORY
OsMed10_1	LOC_Os09g35920	Os_chr09	20674697	[A/G]	REGULATORY
OsMed10_1	LOC_Os09g35920	Os_chr09	20674319	[T/C]	REGULATORY
OsMed10_1	LOC_Os09g35920	Os_chr09	20679151	[A/G]	REGULATORY
OsMed10_1	LOC_Os09g35920	Os_chr09	20678967	[C/T]	REGULATORY
OsMed10_1	LOC_Os09g35920	Os_chr09	20678773	[G/A]	REGULATORY
OsMed10_1	LOC_Os09g35920	Os_chr09	20678648	[G/A]	REGULATORY
OsMed10_1	LOC_Os09g35920	Os_chr09	20678640	[G/T]	REGULATORY
OsMed10_1	LOC_Os09g35920	Os_chr09	20678565	[T/C]	REGULATORY
OsMed10_1	LOC_Os09g35920	Os_chr09	20678485	[C/T]	REGULATORY
OsMed10_1	LOC_Os09g35920	Os_chr09	20678383	[A/G]	REGULATORY
OsMed10_1	LOC_Os09g35920	Os_chr09	20678359	[T/C]	REGULATORY
OsMed10_1	LOC_Os09g35920	Os_chr09	20678262	[T/C]	REGULATORY
OsMed10_1	LOC_Os09g35920	Os_chr09	20678057	[T/C]	SYNONYMOUS-CODING
OsMed10_1	LOC_Os09g35920	Os_chr09	20677964	[G/T]	SYNONYMOUS-CODING
OsMed10_1	LOC_Os09g35920	Os_chr09	20677509	[C/T]	SYNONYMOUS-CODING
OsMed11_1	LOC_Os02g09600	Os_chr02	4941639	[A/G]	INTRON
OsMed11_1	LOC_Os02g09600	Os_chr02	4941622	[G/T]	INTRON
OsMed11_1	LOC_Os02g09600	Os_chr02	4941523	[A/G]	INTRON
OsMed11_1	LOC_Os02g09600	Os_chr02	4941457	[C/A]	INTRON
OsMed11_1	LOC_Os02g09600	Os_chr02	4941364	[A/G]	INTRON
OsMed11_1	LOC_Os02g09600	Os_chr02	4941102	[T/C]	INTRON
OsMed11_1	LOC_Os02g09600	Os_chr02	4941078	[T/A]	INTRON
OsMed11_1	LOC_Os02g09600	Os_chr02	4941013	[G/A]	INTRON
OsMed11_1	LOC_Os02g09600	Os_chr02	4940847	[G/A]	INTRON
OsMed11_1	LOC_Os02g09600	Os_chr02	4940787	[C/A]	INTRON
OsMed11_1	LOC_Os02g09600	Os_chr02	4940777	[G/C]	INTRON
OsMed11_1	LOC_Os02g09600	Os_chr02	4940706	[C/T]	INTRON
OsMed11_1	LOC_Os02g09600	Os_chr02	4940694	[G/A]	INTRON
OsMed11_1	LOC_Os02g09600	Os_chr02	4940677	[A/G]	INTRON
OsMed11_1	LOC_Os02g09600	Os_chr02	4940668	[C/T]	INTRON
OsMed11_1	LOC_Os02g09600	Os_chr02	4940266	[G/T]	INTRON
OsMed11_1	LOC_Os02g09600	Os_chr02	4940217	[A/T]	INTRON
OsMed11_1	LOC_Os02g09600	Os_chr02	4939950	[T/C]	INTRON
OsMed11_1	LOC_Os02g09600	Os_chr02	4939929	[A/G]	INTRON

Mediator genes	MSU locus ID	Chromosomes	Physical Position (bp)	SNPs	Structural Annotation
OsMed11_1	LOC_Os02g09600	Os_chr02	4939814	[G/A]	INTRON
OsMed11_1	LOC_Os02g09600	Os_chr02	4939781	[C/G]	INTRON
OsMed11_1	LOC_Os02g09600	Os_chr02	4939741	[T/A]	INTRON
OsMed11_1	LOC_Os02g09600	Os_chr02	4939662	[C/A]	INTRON
OsMed11_1	LOC_Os02g09600	Os_chr02	4939438	[G/A]	INTRON
OsMed11_1	LOC_Os02g09600	Os_chr02	4939348	[C/T]	INTRON
OsMed11_1	LOC_Os02g09600	Os_chr02	4939320	[C/T]	INTRON
OsMed11_1	LOC_Os02g09600	Os_chr02	4940189	[G/A]	NON-SYNONYMOUS-CODING
OsMed11_1	LOC_Os02g09610	Os_chr02	4943009	[G/A]	NON-SYNONYMOUS-CODING
OsMed11_1	LOC_Os02g09610	Os_chr02	4942995	[C/T]	NON-SYNONYMOUS-CODING
OsMed11_1	LOC_Os02g09610	Os_chr02	4942802	[G/A]	NON-SYNONYMOUS-CODING
OsMed11_1	LOC_Os02g09610	Os_chr02	4942779	[C/T]	NON-SYNONYMOUS-CODING
OsMed11_1	LOC_Os02g09600	Os_chr02	4942000	[G/T]	REGULATORY
OsMed11_1	LOC_Os02g09600	Os_chr02	4941959	[G/A]	REGULATORY
OsMed11_1	LOC_Os02g09600	Os_chr02	4939023	[G/A]	REGULATORY
OsMed11_1	LOC_Os02g09600	Os_chr02	4938902	[C/A]	REGULATORY
OsMed11_1	LOC_Os02g09600	Os_chr02	4938781	[A/G]	REGULATORY
OsMed11_1	LOC_Os02g57590	Os_chr02	4942531	[A/G]	REGULATORY
OsMed11_1	LOC_Os02g57590	Os_chr02	4942458	[A/G]	REGULATORY
OsMed11_1	LOC_Os02g57590	Os_chr02	4942433	[A/T]	REGULATORY
OsMed11_1	LOC_Os02g57590	Os_chr02	4942342	[T/C]	REGULATORY
OsMed11_1	LOC_Os02g57590	Os_chr02	4942296	[T/A]	REGULATORY
OsMed11_1	LOC_Os02g57590	Os_chr02	4942157	[A/C]	REGULATORY
OsMed11_1	LOC_Os02g57590	Os_chr02	4942095	[T/C]	REGULATORY
OsMed11_1	LOC_Os02g09600	Os_chr02	4941708	[A/G]	SYNONYMOUS-CODING
OsMed11_1	LOC_Os02g09600	Os_chr02	4940158	[G/A]	SYNONYMOUS-CODING
OsMed11_2	LOC_Os02g49640	Os_chr02	30333694	[C/T]	INTRON
OsMed11_2	LOC_Os02g49640	Os_chr02	30333593	[G/A]	INTRON
OsMed11_2	LOC_Os02g49640	Os_chr02	30333294	[C/A]	INTRON
OsMed11_2	LOC_Os02g49640	Os_chr02	30333183	[G/T]	INTRON
OsMed11_2	LOC_Os02g49640	Os_chr02	30333102	[C/T]	INTRON
OsMed11_2	LOC_Os02g49640	Os_chr02	30333069	[C/A]	INTRON
OsMed11_2	LOC_Os02g49640	Os_chr02	30332737	[T/C]	INTRON
OsMed11_2	LOC_Os02g49640	Os_chr02	30332631	[T/C]	INTRON
OsMed11_2	LOC_Os02g49640	Os_chr02	30332594	[C/T]	INTRON
OsMed11_2	LOC_Os02g49640	Os_chr02	30332470	[C/T]	INTRON
OsMed11_2	LOC_Os02g49640	Os_chr02	30332377	[A/T]	INTRON
OsMed11_2	LOC_Os02g49640	Os_chr02	30332190	[C/T]	INTRON
OsMed11_2	LOC_Os02g49640	Os_chr02	30332028	[T/C]	INTRON
OsMed11_2	LOC_Os02g49640	Os_chr02	30331886	[A/G]	INTRON
OsMed11_2	LOC_Os02g49640	Os_chr02	30331885	[G/T]	INTRON
OsMed11_2	LOC_Os02g49640	Os_chr02	30331573	[G/A]	INTRON
OsMed11_2	LOC_Os02g49640	Os_chr02	30331522	[T/C]	INTRON
OsMed11_2	LOC_Os02g49640	Os_chr02	30331511	[C/T]	INTRON
OsMed11_2	LOC_Os02g49640	Os_chr02	30331163	[G/A]	INTRON
OsMed11_2	LOC_Os02g49640	Os_chr02	30331156	[G/A]	INTRON
OsMed11_2	LOC_Os02g49640	Os_chr02	30331123	[C/T]	INTRON

Mediator genes	MSU locus ID	Chromosomes	Physical Position (bp)	SNPs	Structural Annotation
OsMed11_2	LOC_Os02g49640	Os_chr02	30331119	[G/A]	INTRON
OsMed11_2	LOC_Os02g49640	Os_chr02	30331004	[T/C]	INTRON
OsMed11_2	LOC_Os02g49640	Os_chr02	30330964	[T/C]	INTRON
OsMed11_2	LOC_Os02g49640	Os_chr02	30330914	[C/T]	INTRON
OsMed11_2	LOC_Os02g49640	Os_chr02	30330882	[T/C]	INTRON
OsMed11_2	LOC_Os02g49640	Os_chr02	30330815	[C/T]	INTRON
OsMed11_2	LOC_Os02g49640	Os_chr02	30330771	[C/T]	INTRON
OsMed11_2	LOC_Os02g49640	Os_chr02	30330750	[C/T]	INTRON
OsMed11_2	LOC_Os02g49640	Os_chr02	30330687	[A/G]	INTRON
OsMed11_2	LOC_Os02g49640	Os_chr02	30330658	[T/C]	INTRON
OsMed11_2	LOC_Os02g49640	Os_chr02	30330593	[A/T]	INTRON
OsMed11_2	LOC_Os02g49640	Os_chr02	30330592	[C/A]	INTRON
OsMed11_2	LOC_Os02g49640	Os_chr02	30331703	[G/A]	NON-SYNONYMOUS-CODING
OsMed11_2	LOC_Os02g49640	Os_chr02	30331464	[A/G]	NON-SYNONYMOUS-CODING
OsMed11_2	LOC_Os02g49640	Os_chr02	30331420	[G/A]	REGULATORY
OsMed11_2	LOC_Os02g49640	Os_chr02	30331399	[C/T]	REGULATORY
OsMed11_2	LOC_Os02g49640	Os_chr02	30330562	[T/G]	REGULATORY
OsMed11_2	LOC_Os02g49640	Os_chr02	30330551	[C/T]	REGULATORY
OsMed11_2	LOC_Os02g49640	Os_chr02	30330502	[C/G]	REGULATORY
OsMed11_2	LOC_Os02g49640	Os_chr02	30331650	[A/C]	REGULATORY
OsMed11_2	LOC_Os02g49640	Os_chr02	30333986	[C/A]	REGULATORY
OsMed11_2	LOC_Os02g49640	Os_chr02	30333958	[G/C]	REGULATORY
OsMed11_2	LOC_Os02g49640	Os_chr02	30333908	[G/T]	REGULATORY
OsMed11_2	LOC_Os02g49640	Os_chr02	30333859	[A/G]	REGULATORY
OsMed11_2	LOC_Os02g57590	Os_chr02	30330450	[C/T]	REGULATORY
OsMed11_2	LOC_Os02g57590	Os_chr02	30330424	[C/A]	REGULATORY
OsMed11_2	LOC_Os02g57590	Os_chr02	30330396	[C/G]	REGULATORY
OsMed11_2	LOC_Os02g57590	Os_chr02	30330298	[G/A]	REGULATORY
OsMed11_2	LOC_Os02g57590	Os_chr02	30330292	[G/A]	REGULATORY
OsMed11_2	LOC_Os02g57590	Os_chr02	30330122	[G/A]	REGULATORY
OsMed11_2	LOC_Os02g57590	Os_chr02	30330069	[T/C]	REGULATORY
OsMed11_2	LOC_Os02g57590	Os_chr02	30329851	[A/G]	REGULATORY
OsMed11_2	LOC_Os02g57590	Os_chr02	30329822	[A/G]	REGULATORY
OsMed11_2	LOC_Os02g57590	Os_chr02	30329808	[C/G]	REGULATORY
OsMed11_2	LOC_Os02g57590	Os_chr02	30329666	[G/A]	REGULATORY
OsMed11_2	LOC_Os02g57590	Os_chr02	30329597	[C/T]	REGULATORY
OsMed11_2	LOC_Os02g57590	Os_chr02	30329560	[A/T]	REGULATORY
OsMed11_2	LOC_Os02g49640	Os_chr02	30331651	[T/G]	SYNONYMOUS-CODING
OsMed12_1	LOC_Os07g45400	Os_chr07	27081531	[G/A]	INTRON
OsMed12_1	LOC_Os07g45400	Os_chr07	27081505	[G/A]	INTRON
OsMed12_1	LOC_Os07g45400	Os_chr07	27081490	[T/A]	INTRON
OsMed12_1	LOC_Os07g45400	Os_chr07	27080907	[C/A]	INTRON
OsMed12_1	LOC_Os07g45400	Os_chr07	27078851	[A/T]	INTRON
OsMed12_1	LOC_Os07g45400	Os_chr07	27078030	[C/T]	INTRON
OsMed12_1	LOC_Os07g45400	Os_chr07	27077882	[C/T]	INTRON
OsMed12_1	LOC_Os07g45400	Os_chr07	27077287	[C/T]	INTRON
OsMed12_1	LOC_Os07g45400	Os_chr07	27077271	[A/G]	INTRON

Mediator genes	MSU locus ID	Chromosomes	Physical Position (bp)	SNPs	Structural Annotation
OsMed12_1	LOC_Os07g45400	Os_chr07	27074539	[C/T]	INTRON
OsMed12_1	LOC_Os07g45400	Os_chr07	27074506	[A/G]	INTRON
OsMed12_1	LOC_Os07g45400	Os_chr07	27074144	[C/T]	INTRON
OsMed12_1	LOC_Os07g45400	Os_chr07	27073951	[G/T]	INTRON
OsMed12_1	LOC_Os07g45400	Os_chr07	27073653	[T/A]	INTRON
OsMed12_1	LOC_Os07g45400	Os_chr07	27073214	[A/G]	INTRON
OsMed12_1	LOC_Os07g45400	Os_chr07	27073189	[A/T]	INTRON
OsMed12_1	LOC_Os07g45400	Os_chr07	27073027	[A/G]	INTRON
OsMed12_1	LOC_Os07g45400	Os_chr07	27072548	[T/G]	INTRON
OsMed12_1	LOC_Os07g45400	Os_chr07	27072425	[C/T]	INTRON
OsMed12_1	LOC_Os07g45400	Os_chr07	27072198	[T/A]	INTRON
OsMed12_1	LOC_Os07g45400	Os_chr07	27072168	[G/A]	INTRON
OsMed12_1	LOC_Os07g45400	Os_chr07	27072109	[G/T]	INTRON
OsMed12_1	LOC_Os07g45400	Os_chr07	27072059	[C/T]	INTRON
OsMed12_1	LOC_Os07g45400	Os_chr07	27071898	[A/G]	INTRON
OsMed12_1	LOC_Os07g45400	Os_chr07	27071813	[G/A]	INTRON
OsMed12_1	LOC_Os07g45400	Os_chr07	27071774	[A/T]	INTRON
OsMed12_1	LOC_Os07g45400	Os_chr07	27071702	[A/G]	INTRON
OsMed12_1	LOC_Os07g45400	Os_chr07	27071693	[G/A]	INTRON
OsMed12_1	LOC_Os07g45400	Os_chr07	27071672	[G/A]	INTRON
OsMed12_1	LOC_Os07g45400	Os_chr07	27071559	[T/G]	INTRON
OsMed12_1	LOC_Os07g45400	Os_chr07	27071375	[C/A]	INTRON
OsMed12_1	LOC_Os07g45400	Os_chr07	27071350	[T/C]	INTRON
OsMed12_1	LOC_Os07g45400	Os_chr07	27071299	[G/A]	INTRON
OsMed12_1	LOC_Os07g45400	Os_chr07	27071297	[G/A]	INTRON
OsMed12_1	LOC_Os07g45400	Os_chr07	27071290	[T/C]	INTRON
OsMed12_1	LOC_Os07g45400	Os_chr07	27071227	[G/A]	INTRON
OsMed12_1	LOC_Os07g45400	Os_chr07	27070664	[C/T]	INTRON
OsMed12_1	LOC_Os07g45400	Os_chr07	27070485	[T/G]	INTRON
OsMed12_1	LOC_Os07g45400	Os_chr07	27070419	[A/C]	INTRON
OsMed12_1	LOC_Os07g45400	Os_chr07	27070403	[C/T]	INTRON
OsMed12_1	LOC_Os07g45400	Os_chr07	27069987	[A/G]	INTRON
OsMed12_1	LOC_Os07g45400	Os_chr07	27069878	[A/T]	INTRON
OsMed12_1	LOC_Os07g45400	Os_chr07	27069873	[G/T]	INTRON
OsMed12_1	LOC_Os07g45400	Os_chr07	27069868	[C/T]	INTRON
OsMed12_1	LOC_Os07g45400	Os_chr07	27069821	[G/A]	INTRON
OsMed12_1	LOC_Os07g45400	Os_chr07	27069800	[C/T]	INTRON
OsMed12_1	LOC_Os07g45400	Os_chr07	27069740	[C/T]	INTRON
OsMed12_1	LOC_Os07g45400	Os_chr07	27069608	[A/G]	INTRON
OsMed12_1	LOC_Os07g45400	Os_chr07	27069606	[G/A]	INTRON
OsMed12_1	LOC_Os07g45400	Os_chr07	27069549	[G/A]	INTRON
OsMed12_1	LOC_Os07g45400	Os_chr07	27069461	[G/A]	INTRON
OsMed12_1	LOC_Os07g45400	Os_chr07	27081817	[G/A]	NON-SYNONYMOUS-CODING
OsMed12_1	LOC_Os07g45400	Os_chr07	27079654	[G/A]	NON-SYNONYMOUS-CODING
OsMed12_1	LOC_Os07g45400	Os_chr07	27079049	[G/A]	NON-SYNONYMOUS-CODING
OsMed12_1	LOC_Os07g45400	Os_chr07	27078941	[G/T]	NON-SYNONYMOUS-CODING
OsMed12_1	LOC_Os07g45400	Os_chr07	27078797	[G/A]	NON-SYNONYMOUS-CODING

Mediator genes	MSU locus ID	Chromosomes	Physical Position (bp)	SNPs	Structural Annotation
OsMed12_1	LOC_Os07g45400	Os_chr07	27078269	[G/A]	NON-SYNONYMOUS-CODING
OsMed12_1	LOC_Os07g45400	Os_chr07	27077085	[C/G]	NON-SYNONYMOUS-CODING
OsMed12_1	LOC_Os07g45400	Os_chr07	27076755	[A/G]	NON-SYNONYMOUS-CODING
OsMed12_1	LOC_Os07g45400	Os_chr07	27076375	[G/A]	NON-SYNONYMOUS-CODING
OsMed12_1	LOC_Os07g45400	Os_chr07	27074921	[C/G]	NON-SYNONYMOUS-CODING
OsMed12_1	LOC_Os07g45400	Os_chr07	27069130	[G/A]	NON-SYNONYMOUS-CODING
OsMed12_1	LOC_Os07g45400	Os_chr07	27069117	[A/C]	NON-SYNONYMOUS-CODING
OsMed12_1	LOC_Os07g45400	Os_chr07	27069064	[C/T]	NON-SYNONYMOUS-CODING
OsMed12_1	LOC_Os07g45400	Os_chr07	27082465	[A/C]	REGULATORY
OsMed12_1	LOC_Os07g45400	Os_chr07	27082311	[G/A]	REGULATORY
OsMed12_1	LOC_Os07g45400	Os_chr07	27082136	[T/G]	REGULATORY
OsMed12_1	LOC_Os07g45400	Os_chr07	27082069	[C/T]	REGULATORY
OsMed12_1	LOC_Os07g45400	Os_chr07	27082063	[A/T]	REGULATORY
OsMed12_1	LOC_Os07g45400	Os_chr07	27068947	[G/A]	REGULATORY
OsMed12_1	LOC_Os07g45400	Os_chr07	27068937	[G/A]	REGULATORY
OsMed12_1	LOC_Os07g45400	Os_chr07	27068879	[C/T]	REGULATORY
OsMed12_1	LOC_Os07g45400	Os_chr07	27068868	[A/G]	REGULATORY
OsMed12_1	LOC_Os07g45400	Os_chr07	27068856	[G/A]	REGULATORY
OsMed12_1	LOC_Os07g45400	Os_chr07	27068830	[C/T]	REGULATORY
OsMed12_1	LOC_Os07g45400	Os_chr07	27068806	[T/C]	REGULATORY
OsMed12_1	LOC_Os07g45400	Os_chr07	27068798	[G/A]	REGULATORY
OsMed12_1	LOC_Os07g45400	Os_chr07	27068777	[A/G]	REGULATORY
OsMed12_1	LOC_Os07g45400	Os_chr07	27068771	[C/A]	REGULATORY
OsMed12_1	LOC_Os07g45400	Os_chr07	27068634	[A/T]	REGULATORY
OsMed12_1	LOC_Os07g45400	Os_chr07	27068627	[A/C]	REGULATORY
OsMed12_1	LOC_Os07g45400	Os_chr07	27068532	[A/G]	REGULATORY
OsMed12_1	LOC_Os07g45400	Os_chr07	27068505	[T/C]	REGULATORY
OsMed12_1	LOC_Os07g45400	Os_chr07	27068363	[A/G]	REGULATORY
OsMed12_1	LOC_Os07g45400	Os_chr07	27081727	[T/C]	SYNONYMOUS-CODING
OsMed12_1	LOC_Os07g45400	Os_chr07	27081278	[C/G]	SYNONYMOUS-CODING
OsMed12_1	LOC_Os07g45400	Os_chr07	27079942	[C/T]	SYNONYMOUS-CODING
OsMed12_1	LOC_Os07g45400	Os_chr07	27079768	[G/A]	SYNONYMOUS-CODING
OsMed12_1	LOC_Os07g45400	Os_chr07	27079747	[G/A]	SYNONYMOUS-CODING
OsMed12_1	LOC_Os07g45400	Os_chr07	27079663	[A/G]	SYNONYMOUS-CODING
OsMed12_1	LOC_Os07g45400	Os_chr07	27079093	[T/A]	SYNONYMOUS-CODING
OsMed12_1	LOC_Os07g45400	Os_chr07	27079006	[G/A]	SYNONYMOUS-CODING
OsMed12_1	LOC_Os07g45400	Os_chr07	27075770	[G/A]	SYNONYMOUS-CODING
OsMed12_1	LOC_Os07g45400	Os_chr07	27075737	[G/A]	SYNONYMOUS-CODING
OsMed12_1	LOC_Os07g45400	Os_chr07	27075067	[G/A]	SYNONYMOUS-CODING
OsMed12_1	LOC_Os07g45400	Os_chr07	27075019	[C/T]	SYNONYMOUS-CODING
OsMed12_1	LOC_Os07g45400	Os_chr07	27072672	[C/A]	SYNONYMOUS-CODING
OsMed12_1	LOC_Os07g45400	Os_chr07	27070572	[C/G]	SYNONYMOUS-CODING
OsMed12_1	LOC_Os07g45400	Os_chr07	27070566	[C/G]	SYNONYMOUS-CODING
OsMed12_1	LOC_Os07g45400	Os_chr07	27069119	[T/C]	SYNONYMOUS-CODING
OsMed12_1	LOC_Os07g45400	Os_chr07	27069092	[C/T]	SYNONYMOUS-CODING
OsMed12_1	LOC_Os07g45400	Os_chr07	27069080	[C/T]	SYNONYMOUS-CODING
OsMed12_1	LOC_Os07g45400	Os_chr07	27069068	[T/C]	SYNONYMOUS-CODING

Mediator genes	MSU locus ID	Chromosomes	Physical Position (bp)	SNPs	Structural Annotation
OsMed12_1	LOC_Os07g45400	Os_chr07	27069050	[C/T]	SYNONYMOUS-CODING
OsMed12_1	LOC_Os07g45400	Os_chr07	27069032	[C/T]	SYNONYMOUS-CODING
OsMed12_2	LOC_Os10g40260	Os_chr10	21507688	[C/A]	INTRON
OsMed12_2	LOC_Os10g40260	Os_chr10	21507626	[C/T]	INTRON
OsMed12_2	LOC_Os10g40260	Os_chr10	21507446	[C/T]	INTRON
OsMed12_2	LOC_Os10g40260	Os_chr10	21507268	[C/T]	INTRON
OsMed12_2	LOC_Os10g40260	Os_chr10	21504635	[T/C]	INTRON
OsMed12_2	LOC_Os10g40260	Os_chr10	21504606	[A/G]	INTRON
OsMed12_2	LOC_Os10g40260	Os_chr10	21504354	[G/A]	INTRON
OsMed12_2	LOC_Os10g40260	Os_chr10	21504182	[T/G]	INTRON
OsMed12_2	LOC_Os10g40260	Os_chr10	21504047	[C/T]	INTRON
OsMed12_2	LOC_Os10g40260	Os_chr10	21503929	[T/C]	INTRON
OsMed12_2	LOC_Os10g40260	Os_chr10	21503431	[T/G]	INTRON
OsMed12_2	LOC_Os10g40260	Os_chr10	21503360	[A/T]	INTRON
OsMed12_2	LOC_Os10g40260	Os_chr10	21501011	[C/T]	INTRON
OsMed12_2	LOC_Os10g40260	Os_chr10	21500124	[A/G]	INTRON
OsMed12_2	LOC_Os10g40260	Os_chr10	21500085	[T/C]	INTRON
OsMed12_2	LOC_Os10g40260	Os_chr10	21499972	[C/T]	INTRON
OsMed12_2	LOC_Os10g40260	Os_chr10	21499861	[C/T]	INTRON
OsMed12_2	LOC_Os10g40260	Os_chr10	21499621	[G/T]	INTRON
OsMed12_2	LOC_Os10g40260	Os_chr10	21498946	[T/A]	INTRON
OsMed12_2	LOC_Os10g40260	Os_chr10	21498662	[A/C]	INTRON
OsMed12_2	LOC_Os10g40260	Os_chr10	21498353	[C/T]	INTRON
OsMed12_2	LOC_Os10g40260	Os_chr10	21498320	[C/T]	INTRON
OsMed12_2	LOC_Os10g40260	Os_chr10	21498285	[A/T]	INTRON
OsMed12_2	LOC_Os10g40260	Os_chr10	21498164	[C/T]	INTRON
OsMed12_2	LOC_Os10g40260	Os_chr10	21498089	[G/T]	INTRON
OsMed12_2	LOC_Os10g40260	Os_chr10	21508418	[C/A]	NON-SYNONYMOUS-CODING
OsMed12_2	LOC_Os10g40260	Os_chr10	21508362	[A/G]	NON-SYNONYMOUS-CODING
OsMed12_2	LOC_Os10g40260	Os_chr10	21508303	[C/T]	NON-SYNONYMOUS-CODING
OsMed12_2	LOC_Os10g40260	Os_chr10	21508037	[C/A]	NON-SYNONYMOUS-CODING
OsMed12_2	LOC_Os10g40260	Os_chr10	21506940	[T/A]	NON-SYNONYMOUS-CODING
OsMed12_2	LOC_Os10g40260	Os_chr10	21503285	[C/G]	NON-SYNONYMOUS-CODING
OsMed12_2	LOC_Os10g40260	Os_chr10	21502760	[A/G]	NON-SYNONYMOUS-CODING
OsMed12_2	LOC_Os10g40260	Os_chr10	21501852	[G/C]	NON-SYNONYMOUS-CODING
OsMed12_2	LOC_Os10g40260	Os_chr10	21501824	[T/C]	NON-SYNONYMOUS-CODING
OsMed12_2	LOC_Os10g40260	Os_chr10	21500376	[C/T]	NON-SYNONYMOUS-CODING
OsMed12_2	LOC_Os10g40260	Os_chr10	21500360	[G/A]	NON-SYNONYMOUS-CODING
OsMed12_2	LOC_Os10g40260	Os_chr10	21508792	[T/G]	REGULATORY
OsMed12_2	LOC_Os10g40260	Os_chr10	21508317	[C/T]	SYNONYMOUS-CODING
OsMed12_2	LOC_Os10g40260	Os_chr10	21499834	[T/C]	SYNONYMOUS-CODING
OsMed12_2	LOC_Os10g40260	Os_chr10	21497244	[G/C]	REGULATORY
OsMed13_1	LOC_Os05g37500	Os_chr05	21896932	[G/C]	INTRON
OsMed13_1	LOC_Os05g37500	Os_chr05	21896925	[C/A]	INTRON
OsMed13_1	LOC_Os05g37500	Os_chr05	21896409	[C/G]	INTRON
OsMed13_1	LOC_Os05g37500	Os_chr05	21896374	[T/C]	INTRON
OsMed13_1	LOC_Os05g37500	Os_chr05	21895914	[T/G]	INTRON

Mediator genes	MSU locus ID	Chromosomes	Physical Position (bp)	SNPs	Structural Annotation
OsMed13_1	LOC_Os05g37500	Os_chr05	21895881	[T/C]	INTRON
OsMed13_1	LOC_Os05g37500	Os_chr05	21895810	[G/A]	INTRON
OsMed13_1	LOC_Os05g37500	Os_chr05	21895752	[A/C]	INTRON
OsMed13_1	LOC_Os05g37500	Os_chr05	21895616	[C/A]	INTRON
OsMed13_1	LOC_Os05g37500	Os_chr05	21895449	[C/T]	INTRON
OsMed13_1	LOC_Os05g37500	Os_chr05	21895220	[G/A]	INTRON
OsMed13_1	LOC_Os05g37500	Os_chr05	21894944	[A/G]	INTRON
OsMed13_1	LOC_Os05g37500	Os_chr05	21894752	[A/T]	INTRON
OsMed13_1	LOC_Os05g37500	Os_chr05	21894533	[T/A]	INTRON
OsMed13_1	LOC_Os05g37500	Os_chr05	21894293	[A/G]	INTRON
OsMed13_1	LOC_Os05g37500	Os_chr05	21894285	[A/C]	INTRON
OsMed13_1	LOC_Os05g37500	Os_chr05	21894252	[A/G]	INTRON
OsMed13_1	LOC_Os05g37500	Os_chr05	21894209	[T/G]	INTRON
OsMed13_1	LOC_Os05g37500	Os_chr05	21894190	[T/G]	INTRON
OsMed13_1	LOC_Os05g37500	Os_chr05	21894146	[G/A]	INTRON
OsMed13_1	LOC_Os05g37500	Os_chr05	21894058	[G/A]	INTRON
OsMed13_1	LOC_Os05g37500	Os_chr05	21893870	[G/A]	INTRON
OsMed13_1	LOC_Os05g37500	Os_chr05	21893152	[T/A]	INTRON
OsMed13_1	LOC_Os05g37500	Os_chr05	21893151	[G/A]	INTRON
OsMed13_1	LOC_Os05g37500	Os_chr05	21893048	[A/G]	INTRON
OsMed13_1	LOC_Os05g37500	Os_chr05	21892916	[G/T]	INTRON
OsMed13_1	LOC_Os05g37500	Os_chr05	21892838	[C/T]	INTRON
OsMed13_1	LOC_Os05g37500	Os_chr05	21892800	[G/T]	INTRON
OsMed13_1	LOC_Os05g37500	Os_chr05	21892512	[G/A]	INTRON
OsMed13_1	LOC_Os05g37500	Os_chr05	21892461	[G/A]	INTRON
OsMed13_1	LOC_Os05g37500	Os_chr05	21892341	[T/A]	INTRON
OsMed13_1	LOC_Os05g37500	Os_chr05	21892163	[G/A]	INTRON
OsMed13_1	LOC_Os05g37500	Os_chr05	21892108	[C/G]	INTRON
OsMed13_1	LOC_Os05g37500	Os_chr05	21891873	[C/T]	INTRON
OsMed13_1	LOC_Os05g37500	Os_chr05	21891735	[T/G]	INTRON
OsMed13_1	LOC_Os05g37500	Os_chr05	21891578	[C/T]	INTRON
OsMed13_1	LOC_Os05g37500	Os_chr05	21890828	[A/G]	INTRON
OsMed13_1	LOC_Os05g37500	Os_chr05	21890646	[C/A]	INTRON
OsMed13_1	LOC_Os05g37500	Os_chr05	21890598	[C/A]	INTRON
OsMed13_1	LOC_Os05g37500	Os_chr05	21890560	[C/G]	INTRON
OsMed13_1	LOC_Os05g37500	Os_chr05	21890465	[G/A]	INTRON
OsMed13_1	LOC_Os05g37500	Os_chr05	21889356	[T/A]	INTRON
OsMed13_1	LOC_Os05g37500	Os_chr05	21889068	[A/G]	INTRON
OsMed13_1	LOC_Os05g37500	Os_chr05	21888734	[T/C]	INTRON
OsMed13_1	LOC_Os05g37500	Os_chr05	21888701	[T/A]	INTRON
OsMed13_1	LOC_Os05g37500	Os_chr05	21888640	[G/T]	INTRON
OsMed13_1	LOC_Os05g37500	Os_chr05	21888177	[T/G]	INTRON
OsMed13_1	LOC_Os05g37500	Os_chr05	21887848	[T/C]	INTRON
OsMed13_1	LOC_Os05g37500	Os_chr05	21885882	[C/T]	INTRON
OsMed13_1	LOC_Os05g37500	Os_chr05	21885435	[A/G]	INTRON
OsMed13_1	LOC_Os05g37500	Os_chr05	21885402	[G/T]	INTRON
OsMed13_1	LOC_Os05g37500	Os_chr05	21885380	[G/A]	INTRON

Mediator genes	MSU locus ID	Chromosomes	Physical Position (bp)	SNPs	Structural Annotation
OsMed13_1	LOC_Os05g37500	Os_chr05	21885232	[A/G]	INTRON
OsMed13_1	LOC_Os05g37500	Os_chr05	21885217	[C/T]	INTRON
OsMed13_1	LOC_Os05g37500	Os_chr05	21885203	[C/T]	INTRON
OsMed13_1	LOC_Os05g37500	Os_chr05	21884798	[T/G]	INTRON
OsMed13_1	LOC_Os05g37500	Os_chr05	21883713	[A/G]	INTRON
OsMed13_1	LOC_Os05g37500	Os_chr05	21883529	[T/C]	INTRON
OsMed13_1	LOC_Os05g37500	Os_chr05	21881638	[G/A]	INTRON
OsMed13_1	LOC_Os05g37500	Os_chr05	21881575	[G/T]	INTRON
OsMed13_1	LOC_Os05g37500	Os_chr05	21881528	[T/C]	INTRON
OsMed13_1	LOC_Os05g37500	Os_chr05	21881507	[C/G]	INTRON
OsMed13_1	LOC_Os05g37500	Os_chr05	21881461	[G/A]	INTRON
OsMed13_1	LOC_Os05g37500	Os_chr05	21897176	[G/A]	NON-SYNONYMOUS-CODING
OsMed13_1	LOC_Os05g37500	Os_chr05	21896678	[C/T]	NON-SYNONYMOUS-CODING
OsMed13_1	LOC_Os05g37500	Os_chr05	21896571	[T/G]	NON-SYNONYMOUS-CODING
OsMed13_1	LOC_Os05g37500	Os_chr05	21895022	[T/C]	NON-SYNONYMOUS-CODING
OsMed13_1	LOC_Os05g37500	Os_chr05	21895010	[C/T]	NON-SYNONYMOUS-CODING
OsMed13_1	LOC_Os05g37500	Os_chr05	21893773	[G/A]	NON-SYNONYMOUS-CODING
OsMed13_1	LOC_Os05g37500	Os_chr05	21887545	[C/T]	NON-SYNONYMOUS-CODING
OsMed13_1	LOC_Os05g37500	Os_chr05	21887098	[A/T]	NON-SYNONYMOUS-CODING
OsMed13_1	LOC_Os05g37500	Os_chr05	21881363	[C/T]	NON-SYNONYMOUS-CODING
OsMed13_1	LOC_Os05g37500	Os_chr05	21895050	[C/A]	REGULATORY
OsMed13_1	LOC_Os05g37500	Os_chr05	21880560	[G/A]	REGULATORY
OsMed13_1	LOC_Os05g37500	Os_chr05	21880513	[T/C]	REGULATORY
OsMed13_1	LOC_Os05g37500	Os_chr05	21898227	[T/G]	REGULATORY
OsMed13_1	LOC_Os05g37500	Os_chr05	21898224	[T/G]	REGULATORY
OsMed13_1	LOC_Os05g37500	Os_chr05	21898002	[T/C]	REGULATORY
OsMed13_1	LOC_Os05g37500	Os_chr05	21897974	[T/G]	REGULATORY
OsMed13_1	LOC_Os05g37500	Os_chr05	21897966	[A/G]	REGULATORY
OsMed13_1	LOC_Os05g37500	Os_chr05	21897663	[C/G]	REGULATORY
OsMed13_1	LOC_Os05g37500	Os_chr05	21897615	[G/T]	REGULATORY
OsMed13_1	LOC_Os05g37500	Os_chr05	21897076	[G/A]	SYNONYMOUS-CODING
OsMed13_1	LOC_Os05g37500	Os_chr05	21893752	[G/A]	SYNONYMOUS-CODING
OsMed13_1	LOC_Os05g37500	Os_chr05	21891094	[G/A]	SYNONYMOUS-CODING
OsMed13_1	LOC_Os05g37500	Os_chr05	21890901	[C/T]	SYNONYMOUS-CODING
OsMed13_1	LOC_Os05g37500	Os_chr05	21888951	[T/C]	SYNONYMOUS-CODING
OsMed13_1	LOC_Os05g37500	Os_chr05	21888921	[G/A]	SYNONYMOUS-CODING
OsMed13_1	LOC_Os05g37500	Os_chr05	21888912	[G/A]	SYNONYMOUS-CODING
OsMed13_1	LOC_Os05g37500	Os_chr05	21887459	[A/G]	SYNONYMOUS-CODING
OsMed13_1	LOC_Os05g37500	Os_chr05	21886084	[T/C]	SYNONYMOUS-CODING
OsMed13_1	LOC_Os05g37500	Os_chr05	21885988	[G/A]	SYNONYMOUS-CODING
OsMed13_1	LOC_Os05g37500	Os_chr05	21883499	[A/T]	SYNONYMOUS-CODING
OsMed13_1	LOC_Os05g37500	Os_chr05	21883046	[G/A]	SYNONYMOUS-CODING
OsMed13_1	LOC_Os05g37500	Os_chr05	21882926	[T/C]	SYNONYMOUS-CODING
OsMed13_1	LOC_Os05g37500	Os_chr05	21882857	[A/G]	SYNONYMOUS-CODING
OsMed13_1	LOC_Os05g37500	Os_chr05	21881115	[A/G]	SYNONYMOUS-CODING
OsMed14_1	LOC_Os08g24400	Os_chr08	14738229	[T/C]	INTRON
OsMed14_1	LOC_Os08g24400	Os_chr08	14738212	[G/A]	INTRON

**Mediator genes**	**MSU locus ID**	**Chromosomes**	**Physical Position (bp)**	**SNPs**	**Structural Annotation**
OsMed14_1	LOC_Os08g24400	Os_chr08	14738101	[G/A]	INTRON
OsMed14_1	LOC_Os08g24400	Os_chr08	14738100	[C/T]	INTRON
OsMed14_1	LOC_Os08g24400	Os_chr08	14738015	[C/G]	INTRON
OsMed14_1	LOC_Os08g24400	Os_chr08	14737982	[C/T]	INTRON
OsMed14_1	LOC_Os08g24400	Os_chr08	14737927	[T/A]	INTRON
OsMed14_1	LOC_Os08g24400	Os_chr08	14737908	[A/G]	INTRON
OsMed14_1	LOC_Os08g24400	Os_chr08	14737863	[C/T]	INTRON
OsMed14_1	LOC_Os08g24400	Os_chr08	14737784	[C/T]	INTRON
OsMed14_1	LOC_Os08g24400	Os_chr08	14737682	[C/T]	INTRON
OsMed14_1	LOC_Os08g24400	Os_chr08	14737407	[T/C]	INTRON
OsMed14_1	LOC_Os08g24400	Os_chr08	14737396	[A/T]	INTRON
OsMed14_1	LOC_Os08g24400	Os_chr08	14737255	[T/C]	INTRON
OsMed14_1	LOC_Os08g24400	Os_chr08	14737127	[T/C]	INTRON
OsMed14_1	LOC_Os08g24400	Os_chr08	14737028	[T/G]	INTRON
OsMed14_1	LOC_Os08g24400	Os_chr08	14736901	[T/A]	INTRON
OsMed14_1	LOC_Os08g24400	Os_chr08	14736824	[G/A]	INTRON
OsMed14_1	LOC_Os08g24400	Os_chr08	14736727	[A/G]	INTRON
OsMed14_1	LOC_Os08g24400	Os_chr08	14736662	[C/T]	INTRON
OsMed14_1	LOC_Os08g24400	Os_chr08	14736610	[G/A]	INTRON
OsMed14_1	LOC_Os08g24400	Os_chr08	14736594	[A/T]	INTRON
OsMed14_1	LOC_Os08g24400	Os_chr08	14739304	[T/C]	NON-SYNONYMOUS-CODING
OsMed14_1	LOC_Os08g24400	Os_chr08	14739250	[C/T]	NON-SYNONYMOUS-CODING
OsMed14_1	LOC_Os08g24400	Os_chr08	14739135	[C/G]	NON-SYNONYMOUS-CODING
OsMed14_1	LOC_Os08g24400	Os_chr08	14738558	[A/T]	NON-SYNONYMOUS-CODING
OsMed14_1	LOC_Os08g24400	Os_chr08	14738521	[C/T]	NON-SYNONYMOUS-CODING
OsMed14_1	LOC_Os08g24400	Os_chr08	14736229	[G/A]	REGULATORY
OsMed14_1	LOC_Os08g24400	Os_chr08	14736149	[T/G]	REGULATORY
OsMed14_1	LOC_Os08g24400	Os_chr08	14735959	[C/T]	REGULATORY
OsMed14_1	LOC_Os08g24400	Os_chr08	14735756	[C/A]	REGULATORY
OsMed14_1	LOC_Os08g24400	Os_chr08	14735715	[C/T]	REGULATORY
OsMed14_1	LOC_Os08g24400	Os_chr08	14735474	[C/T]	REGULATORY
OsMed14_1	LOC_Os08g24400	Os_chr08	14735459	[C/G]	REGULATORY
OsMed14_1	LOC_Os08g24400	Os_chr08	14735425	[C/T]	REGULATORY
OsMed14_1	LOC_Os08g24400	Os_chr08	14735419	[G/A]	REGULATORY
OsMed14_1	LOC_Os08g24400	Os_chr08	14735405	[A/T]	REGULATORY
OsMed14_1	LOC_Os08g24400	Os_chr08	14735329	[G/A]	REGULATORY
OsMed14_1	LOC_Os08g24400	Os_chr08	14735328	[C/T]	REGULATORY
OsMed14_1	LOC_Os08g24400	Os_chr08	14735314	[C/T]	REGULATORY
OsMed14_1	LOC_Os08g24400	Os_chr08	14735252	[C/T]	REGULATORY
OsMed14_1	LOC_Os08g24400	Os_chr08	14735241	[G/A]	REGULATORY
OsMed14_1	LOC_Os08g24400	Os_chr08	14735168	[G/A]	REGULATORY
OsMed14_1	LOC_Os08g24400	Os_chr08	14735162	[G/A]	REGULATORY
OsMed14_1	LOC_Os08g24400	Os_chr08	14735157	[A/G]	REGULATORY
OsMed14_1	LOC_Os08g24400	Os_chr08	14735144	[C/T]	REGULATORY
OsMed14_1	LOC_Os08g24400	Os_chr08	14735070	[A/T]	REGULATORY
OsMed14_1	LOC_Os08g24400	Os_chr08	14735038	[C/T]	REGULATORY
OsMed14_1	LOC_Os08g24400	Os_chr08	14735026	[C/T]	REGULATORY

Mediator genes	MSU locus ID	Chromosomes	Physical Position (bp)	SNPs	Structural Annotation
OsMed14_1	LOC_Os08g24400	Os_chr08	14735022	[T/G]	REGULATORY
OsMed14_1	LOC_Os08g24400	Os_chr08	14735013	[C/T]	REGULATORY
OsMed14_1	LOC_Os08g24400	Os_chr08	14734978	[C/G]	REGULATORY
OsMed14_1	LOC_Os08g24400	Os_chr08	14734971	[G/A]	REGULATORY
OsMed14_1	LOC_Os08g24400	Os_chr08	14734951	[T/G]	REGULATORY
OsMed14_1	LOC_Os08g24400	Os_chr08	14734947	[G/A]	REGULATORY
OsMed14_1	LOC_Os08g24400	Os_chr08	14734928	[A/C]	REGULATORY
OsMed14_1	LOC_Os08g24400	Os_chr08	14734881	[C/T]	REGULATORY
OsMed14_1	LOC_Os08g24400	Os_chr08	14739369	[G/T]	SYNONYMOUS-CODING
OsMed14_1	LOC_Os08g24400	Os_chr08	14738883	[T/C]	SYNONYMOUS-CODING
OsMed14_1	LOC_Os08g24400	Os_chr08	14738403	[T/A]	SYNONYMOUS-CODING
OsMed14_1	LOC_Os08g24400	Os_chr08	14738400	[T/C]	SYNONYMOUS-CODING
OsMed14_1	LOC_Os08g24400	Os_chr08	14738325	[C/T]	SYNONYMOUS-CODING
OsMed14_1	LOC_Os08g24400	Os_chr08	14736452	[C/T]	SYNONYMOUS-CODING
OsMed14_1	LOC_Os08g24400	Os_chr08	14736422	[C/T]	SYNONYMOUS-CODING
OsMed14_1	LOC_Os08g24400	Os_chr08	14736335	[C/T]	SYNONYMOUS-CODING
OsMed14_2	LOC_Os09g10960	Os_chr09	6044817	[A/T]	INTRON
OsMed14_2	LOC_Os09g10960	Os_chr09	6043563	[T/C]	INTRON
OsMed14_2	LOC_Os09g10960	Os_chr09	6040991	[C/G]	INTRON
OsMed14_2	LOC_Os09g10960	Os_chr09	6044991	[G/A]	INTRON
OsMed14_2	LOC_Os09g10960	Os_chr09	6047360	[C/T]	INTRON
OsMed14_2	LOC_Os09g10960	Os_chr09	6041201	[A/C]	INTRON
OsMed14_2	LOC_Os09g10960	Os_chr09	6042946	[A/T]	INTRON
OsMed14_2	LOC_Os09g10960	Os_chr09	6047076	[T/C]	INTRON
OsMed14_2	LOC_Os09g10960	Os_chr09	6047614	[G/A]	INTRON
OsMed14_2	LOC_Os09g10960	Os_chr09	6046961	[A/G]	INTRON
OsMed14_2	LOC_Os09g10960	Os_chr09	6041169	[C/T]	INTRON
OsMed14_2	LOC_Os09g10960	Os_chr09	6038799	[A/T]	INTRON
OsMed14_2	LOC_Os09g10960	Os_chr09	6042782	[A/G]	INTRON
OsMed14_2	LOC_Os09g10960	Os_chr09	6045017	[C/A]	INTRON
OsMed14_2	LOC_Os09g10960	Os_chr09	6041488	[T/C]	INTRON
OsMed14_2	LOC_Os09g10960	Os_chr09	6042745	[C/A]	INTRON
OsMed14_2	LOC_Os09g10960	Os_chr09	6046876	[C/T]	INTRON
OsMed14_2	LOC_Os09g10960	Os_chr09	6045567	[T/C]	INTRON
OsMed14_2	LOC_Os09g10960	Os_chr09	6043317	[G/A]	INTRON
OsMed14_2	LOC_Os09g10960	Os_chr09	6044042	[C/T]	INTRON
OsMed14_2	LOC_Os09g10960	Os_chr09	6043711	[C/T]	INTRON
OsMed14_2	LOC_Os09g10960	Os_chr09	6042938	[A/C]	INTRON
OsMed14_2	LOC_Os09g10960	Os_chr09	6043596	[A/G]	INTRON
OsMed14_2	LOC_Os09g10960	Os_chr09	6046824	[G/A]	INTRON
OsMed14_2	LOC_Os09g10960	Os_chr09	6044977	[C/A]	INTRON
OsMed14_2	LOC_Os09g10960	Os_chr09	6043525	[G/A]	INTRON
OsMed14_2	LOC_Os09g10960	Os_chr09	6043190	[T/A]	INTRON
OsMed14_2	LOC_Os09g10960	Os_chr09	6045283	[C/T]	INTRON
OsMed14_2	LOC_Os09g10960	Os_chr09	6041359	[T/C]	INTRON
OsMed14_2	LOC_Os09g10960	Os_chr09	6043951	[G/A]	INTRON
OsMed14_2	LOC_Os09g10960	Os_chr09	6043179	[G/T]	INTRON

**Mediator genes**	**MSU locus ID**	**Chromosomes**	**Physical Position (bp)**	**SNPs**	**Structural Annotation**
OsMed14_2	LOC_Os09g10960	Os_chr09	6038225	[C/G]	INTRON
OsMed14_2	LOC_Os09g10960	Os_chr09	6038194	[G/A]	INTRON
OsMed14_2	LOC_Os09g10960	Os_chr09	6038512	[G/A]	INTRON
OsMed14_2	LOC_Os09g10960	Os_chr09	6042653	[G/A]	INTRON
OsMed14_2	LOC_Os09g10960	Os_chr09	6038842	[A/G]	INTRON
OsMed14_2	LOC_Os09g10960	Os_chr09	6043985	[G/A]	INTRON
OsMed14_2	LOC_Os09g10960	Os_chr09	6043461	[G/A]	INTRON
OsMed14_2	LOC_Os09g10960	Os_chr09	6038761	[A/G]	INTRON
OsMed14_2	LOC_Os09g10960	Os_chr09	6046904	[T/C]	INTRON
OsMed14_2	LOC_Os09g10960	Os_chr09	6045051	[T/C]	INTRON
OsMed14_2	LOC_Os09g10960	Os_chr09	6043914	[A/G]	INTRON
OsMed14_2	LOC_Os09g10960	Os_chr09	6042477	[A/G]	INTRON
OsMed14_2	LOC_Os09g10960	Os_chr09	6041165	[T/G]	INTRON
OsMed14_2	LOC_Os09g10960	Os_chr09	6038159	[A/G]	INTRON
OsMed14_2	LOC_Os09g10960	Os_chr09	6046656	[C/T]	INTRON
OsMed14_2	LOC_Os09g10960	Os_chr09	6043887	[G/A]	INTRON
OsMed14_2	LOC_Os09g10960	Os_chr09	6041274	[A/T]	INTRON
OsMed14_2	LOC_Os09g10960	Os_chr09	6041167	[C/T]	INTRON
OsMed14_2	LOC_Os09g10960	Os_chr09	6044551	[G/A]	INTRON
OsMed14_2	LOC_Os09g10960	Os_chr09	6046892	[C/T]	INTRON
OsMed14_2	LOC_Os09g10960	Os_chr09	6047135	[T/C]	INTRON
OsMed14_2	LOC_Os09g10960	Os_chr09	6044875	[C/A]	INTRON
OsMed14_2	LOC_Os09g10960	Os_chr09	6038425	[C/T]	INTRON
OsMed14_2	LOC_Os09g10960	Os_chr09	6038801	[G/A]	INTRON
OsMed14_2	LOC_Os09g10960	Os_chr09	6041508	[G/A]	INTRON
OsMed14_2	LOC_Os09g10960	Os_chr09	6044350	[T/A]	INTRON
OsMed14_2	LOC_Os09g10960	Os_chr09	6043673	[G/A]	INTRON
OsMed14_2	LOC_Os09g10960	Os_chr09	6046790	[G/C]	INTRON
OsMed14_2	LOC_Os09g10960	Os_chr09	6045321	[C/T]	INTRON
OsMed14_2	LOC_Os09g10960	Os_chr09	6045042	[G/T]	INTRON
OsMed14_2	LOC_Os09g10960	Os_chr09	6044711	[T/A]	INTRON
OsMed14_2	LOC_Os09g10960	Os_chr09	6043321	[G/A]	INTRON
OsMed14_2	LOC_Os09g10960	Os_chr09	6040985	[G/A]	INTRON
OsMed14_2	LOC_Os09g10960	Os_chr09	6043708	[C/G]	INTRON
OsMed14_2	LOC_Os09g10960	Os_chr09	6042847	[T/G]	INTRON
OsMed14_2	LOC_Os09g10960	Os_chr09	6042759	[T/A]	INTRON
OsMed14_2	LOC_Os09g10960	Os_chr09	6041308	[T/C]	INTRON
OsMed14_2	LOC_Os09g10960	Os_chr09	6046657	[A/G]	INTRON
OsMed14_2	LOC_Os09g10960	Os_chr09	6044681	[C/T]	INTRON
OsMed14_2	LOC_Os09g10960	Os_chr09	6038351	[G/A]	INTRON
OsMed14_2	LOC_Os09g10960	Os_chr09	6046003	[G/A]	NON-SYNONYMOUS-CODING
OsMed14_2	LOC_Os09g10960	Os_chr09	6046061	[G/T]	NON-SYNONYMOUS-CODING
OsMed14_2	LOC_Os09g10960	Os_chr09	6037811	[A/C]	NON-SYNONYMOUS-CODING
OsMed14_2	LOC_Os09g10960	Os_chr09	6039629	[G/A]	NON-SYNONYMOUS-CODING
OsMed14_2	LOC_Os09g10960	Os_chr09	6037822	[G/A]	NON-SYNONYMOUS-CODING
OsMed14_2	LOC_Os09g10960	Os_chr09	6040197	[A/T]	NON-SYNONYMOUS-CODING
OsMed14_2	LOC_Os09g10960	Os_chr09	6040796	[G/A]	NON-SYNONYMOUS-CODING

Mediator genes	MSU locus ID	Chromosomes	Physical Position (bp)	SNPs	Structural Annotation
OsMed14_2	LOC_Os09g10960	Os_chr09	6042095	[C/T]	NON-SYNONYMOUS-CODING
OsMed14_2	LOC_Os09g10960	Os_chr09	6039689	[G/A]	NON-SYNONYMOUS-CODING
OsMed14_2	LOC_Os09g10960	Os_chr09	6040890	[G/C]	NON-SYNONYMOUS-CODING
OsMed14_2	LOC_Os09g10960	Os_chr09	6042351	[C/G]	NON-SYNONYMOUS-CODING
OsMed14_2	LOC_Os09g10960	Os_chr09	6046429	[G/A]	NON-SYNONYMOUS-CODING
OsMed14_2	LOC_Os09g10960	Os_chr09	6045778	[A/C]	NON-SYNONYMOUS-CODING
OsMed14_2	LOC_Os09g10960	Os_chr09	6047887	[T/C]	NON-SYNONYMOUS-CODING
OsMed14_2	LOC_Os09g10960	Os_chr09	6040689	[T/C]	NON-SYNONYMOUS-CODING
OsMed14_2	LOC_Os09g10960	Os_chr09	6037542	[A/G]	NON-SYNONYMOUS-CODING
OsMed14_2	LOC_Os09g10960	Os_chr09	6039690	[C/T]	NON-SYNONYMOUS-CODING
OsMed14_2	LOC_Os09g10960	Os_chr09	6045929	[C/T]	NON-SYNONYMOUS-CODING
OsMed14_2	LOC_Os09g10960	Os_chr09	6048075	[A/T]	REGULATORY
OsMed14_2	LOC_Os09g10960	Os_chr09	6047958	[G/T]	REGULATORY
OsMed14_2	LOC_Os09g10960	Os_chr09	6047936	[T/C]	REGULATORY
OsMed14_2	LOC_Os09g10960	Os_chr09	6048168	[G/A]	REGULATORY
OsMed14_2	LOC_Os09g10960	Os_chr09	6047937	[C/T]	REGULATORY
OsMed14_2	LOC_Os09g10960	Os_chr09	6036888	[T/C]	REGULATORY
OsMed14_2	LOC_Os09g10960	Os_chr09	6036944	[C/T]	REGULATORY
OsMed14_2	LOC_Os09g10960	Os_chr09	6048791	[G/A]	REGULATORY
OsMed14_2	LOC_Os09g10960	Os_chr09	6048662	[T/G]	REGULATORY
OsMed14_2	LOC_Os09g10960	Os_chr09	6048989	[A/C]	REGULATORY
OsMed14_2	LOC_Os09g10960	Os_chr09	6049112	[A/G]	REGULATORY
OsMed14_2	LOC_Os09g10960	Os_chr09	6049123	[G/A]	REGULATORY
OsMed14_2	LOC_Os09g10960	Os_chr09	6048681	[T/C]	REGULATORY
OsMed14_2	LOC_Os09g10960	Os_chr09	6048665	[A/G]	REGULATORY
OsMed14_2	LOC_Os09g10960	Os_chr09	6048605	[T/G]	REGULATORY
OsMed14_2	LOC_Os09g10960	Os_chr09	6049094	[C/T]	REGULATORY
OsMed14_2	LOC_Os09g10960	Os_chr09	6049083	[G/A]	REGULATORY
OsMed14_2	LOC_Os09g10960	Os_chr09	6049039	[A/G]	REGULATORY
OsMed14_2	LOC_Os09g10960	Os_chr09	6048915	[A/T]	REGULATORY
OsMed14_2	LOC_Os09g10960	Os_chr09	6048854	[A/G]	REGULATORY
OsMed14_2	LOC_Os09g10960	Os_chr09	6049048	[T/C]	REGULATORY
OsMed14_2	LOC_Os09g10960	Os_chr09	6048668	[A/G]	REGULATORY
OsMed14_2	LOC_Os09g10960	Os_chr09	6049082	[G/A]	REGULATORY
OsMed14_2	LOC_Os09g10960	Os_chr09	6048997	[C/T]	REGULATORY
OsMed14_2	LOC_Os09g10960	Os_chr09	6049155	[C/A]	REGULATORY
OsMed14_2	LOC_Os09g10960	Os_chr09	6048907	[C/A]	REGULATORY
OsMed14_2	LOC_Os09g10960	Os_chr09	6048794	[G/T]	REGULATORY
OsMed14_2	LOC_Os09g10960	Os_chr09	6049002	[C/T]	REGULATORY
OsMed14_2	LOC_Os09g10960	Os_chr09	6048459	[T/C]	REGULATORY
OsMed14_2	LOC_Os09g10960	Os_chr09	6048345	[G/C]	REGULATORY
OsMed14_2	LOC_Os09g10960	Os_chr09	6048811	[C/A]	REGULATORY
OsMed14_2	LOC_Os09g10960	Os_chr09	6049012	[G/A]	REGULATORY
OsMed14_2	LOC_Os09g10960	Os_chr09	6048929	[T/C]	REGULATORY
OsMed14_2	LOC_Os09g10960	Os_chr09	6048813	[C/T]	REGULATORY
OsMed14_2	LOC_Os09g10960	Os_chr09	6048572	[C/T]	REGULATORY
OsMed14_2	LOC_Os09g10960	Os_chr09	6043152	[G/A]	SYNONYMOUS-CODING

Mediator genes	MSU locus ID	Chromosomes	Physical Position (bp)	SNPs	Structural Annotation
OsMed14_2	LOC_Os09g10960	Os_chr09	6040150	[G/A]	SYNONYMOUS-CODING
OsMed14_2	LOC_Os09g10960	Os_chr09	6046488	[C/T]	SYNONYMOUS-CODING
OsMed14_2	LOC_Os09g10960	Os_chr09	6043119	[G/A]	SYNONYMOUS-CODING
OsMed14_2	LOC_Os09g10960	Os_chr09	6045969	[A/G]	SYNONYMOUS-CODING
OsMed14_2	LOC_Os09g10960	Os_chr09	6046119	[C/T]	SYNONYMOUS-CODING
OsMed14_2	LOC_Os09g10960	Os_chr09	6037760	[T/C]	SYNONYMOUS-CODING
OsMed14_2	LOC_Os09g10960	Os_chr09	6046224	[A/T]	SYNONYMOUS-CODING
OsMed14_2	LOC_Os09g10960	Os_chr09	6047703	[G/A]	SYNONYMOUS-CODING
OsMed14_2	LOC_Os09g10960	Os_chr09	6039886	[G/A]	SYNONYMOUS-CODING
OsMed14_2	LOC_Os09g10960	Os_chr09	6040660	[A/T]	SYNONYMOUS-CODING
OsMed14_2	LOC_Os09g10960	Os_chr09	6046389	[G/A]	SYNONYMOUS-CODING
OsMed14_2	LOC_Os09g10960	Os_chr09	6045795	[C/T]	SYNONYMOUS-CODING
OsMed14_2	LOC_Os09g10960	Os_chr09	6039112	[G/A]	SYNONYMOUS-CODING
OsMed14_2	LOC_Os09g10960	Os_chr09	6037445	[G/A]	SYNONYMOUS-CODING
OsMed14_2	LOC_Os09g10960	Os_chr09	6040351	[A/G]	SYNONYMOUS-CODING
OsMed14_2	LOC_Os09g10960	Os_chr09	6040924	[A/G]	SYNONYMOUS-CODING
OsMed14_2	LOC_Os09g10960	Os_chr09	6037883	[C/T]	SYNONYMOUS-CODING
OsMed14_2	LOC_Os09g10960	Os_chr09	6039544	[C/T]	SYNONYMOUS-CODING
OsMed15_1	LOC_Os04g03860	Os_chr04	1754898	[T/C]	INTRON
OsMed15_1	LOC_Os04g03860	Os_chr04	1754748	[A/G]	INTRON
OsMed15_1	LOC_Os04g03860	Os_chr04	1754728	[C/T]	INTRON
OsMed15_1	LOC_Os04g03860	Os_chr04	1754720	[G/T]	INTRON
OsMed15_1	LOC_Os04g03860	Os_chr04	1754681	[T/C]	INTRON
OsMed15_1	LOC_Os04g03860	Os_chr04	1754667	[T/A]	INTRON
OsMed15_1	LOC_Os04g03860	Os_chr04	1754434	[C/T]	INTRON
OsMed15_1	LOC_Os04g03860	Os_chr04	1754431	[G/A]	INTRON
OsMed15_1	LOC_Os04g03860	Os_chr04	1754409	[A/T]	INTRON
OsMed15_1	LOC_Os04g03860	Os_chr04	1754374	[G/A]	INTRON
OsMed15_1	LOC_Os04g03860	Os_chr04	1754357	[T/C]	INTRON
OsMed15_1	LOC_Os04g03860	Os_chr04	1753988	[G/T]	INTRON
OsMed15_1	LOC_Os04g03860	Os_chr04	1753074	[C/T]	INTRON
OsMed15_1	LOC_Os04g03860	Os_chr04	1753069	[C/T]	INTRON
OsMed15_1	LOC_Os04g03860	Os_chr04	1753049	[T/C]	INTRON
OsMed15_1	LOC_Os04g03860	Os_chr04	1753039	[A/T]	INTRON
OsMed15_1	LOC_Os04g03860	Os_chr04	1752987	[A/T]	INTRON
OsMed15_1	LOC_Os04g03860	Os_chr04	1752918	[G/A]	INTRON
OsMed15_1	LOC_Os04g03860	Os_chr04	1752883	[G/C]	INTRON
OsMed15_1	LOC_Os04g03860	Os_chr04	1752563	[T/C]	INTRON
OsMed15_1	LOC_Os04g03860	Os_chr04	1752510	[A/G]	INTRON
OsMed15_1	LOC_Os04g03860	Os_chr04	1752494	[C/T]	INTRON
OsMed15_1	LOC_Os04g03860	Os_chr04	1752480	[C/G]	INTRON
OsMed15_1	LOC_Os04g03860	Os_chr04	1752360	[A/T]	INTRON
OsMed15_1	LOC_Os04g03860	Os_chr04	1752354	[G/C]	INTRON
OsMed15_1	LOC_Os04g03860	Os_chr04	1752343	[G/A]	INTRON
OsMed15_1	LOC_Os04g03860	Os_chr04	1752319	[A/G]	INTRON
OsMed15_1	LOC_Os04g03860	Os_chr04	1752318	[G/A]	INTRON
OsMed15_1	LOC_Os04g03860	Os_chr04	1752289	[G/A]	INTRON

**Mediator genes**	**MSU locus ID**	**Chromosomes**	**Physical Position (bp)**	**SNPs**	**Structural Annotation**
OsMed15_1	LOC_Os04g03860	Os_chr04	1752160	[C/T]	INTRON
OsMed15_1	LOC_Os04g03860	Os_chr04	1752147	[A/C]	INTRON
OsMed15_1	LOC_Os04g03860	Os_chr04	1752113	[A/T]	INTRON
OsMed15_1	LOC_Os04g03860	Os_chr04	1751320	[C/T]	INTRON
OsMed15_1	LOC_Os04g03860	Os_chr04	1751255	[G/T]	INTRON
OsMed15_1	LOC_Os04g03860	Os_chr04	1751191	[A/G]	INTRON
OsMed15_1	LOC_Os04g03860	Os_chr04	1751153	[G/A]	INTRON
OsMed15_1	LOC_Os04g03860	Os_chr04	1751113	[G/A]	INTRON
OsMed15_1	LOC_Os04g03860	Os_chr04	1751031	[A/T]	INTRON
OsMed15_1	LOC_Os04g03860	Os_chr04	1750881	[A/G]	INTRON
OsMed15_1	LOC_Os04g03860	Os_chr04	1750871	[C/T]	INTRON
OsMed15_1	LOC_Os04g03860	Os_chr04	1750812	[C/A]	INTRON
OsMed15_1	LOC_Os04g03860	Os_chr04	1750758	[T/C]	INTRON
OsMed15_1	LOC_Os04g03860	Os_chr04	1750754	[G/A]	INTRON
OsMed15_1	LOC_Os04g03860	Os_chr04	1750739	[T/A]	INTRON
OsMed15_1	LOC_Os04g03860	Os_chr04	1750699	[C/G]	INTRON
OsMed15_1	LOC_Os04g03860	Os_chr04	1750690	[G/A]	INTRON
OsMed15_1	LOC_Os04g03860	Os_chr04	1750682	[T/C]	INTRON
OsMed15_1	LOC_Os04g03860	Os_chr04	1750642	[C/T]	INTRON
OsMed15_1	LOC_Os04g03860	Os_chr04	1750580	[A/C]	INTRON
OsMed15_1	LOC_Os04g03860	Os_chr04	1750554	[T/C]	INTRON
OsMed15_1	LOC_Os04g03860	Os_chr04	1750512	[G/A]	INTRON
OsMed15_1	LOC_Os04g03860	Os_chr04	1750489	[A/G]	INTRON
OsMed15_1	LOC_Os04g03860	Os_chr04	1750447	[C/T]	INTRON
OsMed15_1	LOC_Os04g03860	Os_chr04	1750411	[T/A]	INTRON
OsMed15_1	LOC_Os04g03860	Os_chr04	1750348	[G/A]	INTRON
OsMed15_1	LOC_Os04g03860	Os_chr04	1750339	[G/A]	INTRON
OsMed15_1	LOC_Os04g03860	Os_chr04	1750321	[G/C]	INTRON
OsMed15_1	LOC_Os04g03860	Os_chr04	1750307	[A/C]	INTRON
OsMed15_1	LOC_Os04g03860	Os_chr04	1750303	[T/C]	INTRON
OsMed15_1	LOC_Os04g03860	Os_chr04	1750269	[T/A]	INTRON
OsMed15_1	LOC_Os04g03860	Os_chr04	1750255	[A/G]	INTRON
OsMed15_1	LOC_Os04g03860	Os_chr04	1750251	[C/G]	INTRON
OsMed15_1	LOC_Os04g03860	Os_chr04	1755149	[C/A]	NON-SYNONYMOUS-CODING
OsMed15_1	LOC_Os04g03860	Os_chr04	1755146	[G/T]	NON-SYNONYMOUS-CODING
OsMed15_1	LOC_Os04g03860	Os_chr04	1755144	[A/G]	NON-SYNONYMOUS-CODING
OsMed15_1	LOC_Os04g03860	Os_chr04	1755097	[G/T]	NON-SYNONYMOUS-CODING
OsMed15_1	LOC_Os04g03860	Os_chr04	1755057	[G/A]	NON-SYNONYMOUS-CODING
OsMed15_1	LOC_Os04g03860	Os_chr04	1755048	[C/T]	NON-SYNONYMOUS-CODING
OsMed15_1	LOC_Os04g03860	Os_chr04	1755000	[G/A]	NON-SYNONYMOUS-CODING
OsMed15_1	LOC_Os04g03860	Os_chr04	1754998	[C/T]	NON-SYNONYMOUS-CODING
OsMed15_1	LOC_Os04g03860	Os_chr04	1754979	[C/T]	NON-SYNONYMOUS-CODING
OsMed15_1	LOC_Os04g03860	Os_chr04	1754961	[G/A]	NON-SYNONYMOUS-CODING
OsMed15_1	LOC_Os04g03860	Os_chr04	1754635	[C/G]	NON-SYNONYMOUS-CODING
OsMed15_1	LOC_Os04g03860	Os_chr04	1754602	[T/G]	NON-SYNONYMOUS-CODING
OsMed15_1	LOC_Os04g03860	Os_chr04	1754596	[A/T]	NON-SYNONYMOUS-CODING
OsMed15_1	LOC_Os04g03860	Os_chr04	1754571	[G/A]	NON-SYNONYMOUS-CODING

**Mediator genes**	**MSU locus ID**	**Chromosomes**	**Physical Position (bp)**	**SNPs**	**Structural Annotation**
OsMed15_1	LOC_Os04g03860	Os_chr04	1754523	[A/T]	NON-SYNONYMOUS-CODING
OsMed15_1	LOC_Os04g03860	Os_chr04	1754519	[G/C]	NON-SYNONYMOUS-CODING
OsMed15_1	LOC_Os04g03860	Os_chr04	1754505	[G/A]	NON-SYNONYMOUS-CODING
OsMed15_1	LOC_Os04g03860	Os_chr04	1754254	[C/T]	NON-SYNONYMOUS-CODING
OsMed15_1	LOC_Os04g03860	Os_chr04	1753866	[C/T]	NON-SYNONYMOUS-CODING
OsMed15_1	LOC_Os04g03860	Os_chr04	1753849	[T/C]	NON-SYNONYMOUS-CODING
OsMed15_1	LOC_Os04g03860	Os_chr04	1753779	[C/G]	NON-SYNONYMOUS-CODING
OsMed15_1	LOC_Os04g03860	Os_chr04	1753768	[T/A]	NON-SYNONYMOUS-CODING
OsMed15_1	LOC_Os04g03860	Os_chr04	1753762	[C/A]	NON-SYNONYMOUS-CODING
OsMed15_1	LOC_Os04g03860	Os_chr04	1753737	[G/C]	NON-SYNONYMOUS-CODING
OsMed15_1	LOC_Os04g03860	Os_chr04	1753656	[G/A]	NON-SYNONYMOUS-CODING
OsMed15_1	LOC_Os04g03860	Os_chr04	1753654	[G/C]	NON-SYNONYMOUS-CODING
OsMed15_1	LOC_Os04g03860	Os_chr04	1753650	[G/A]	NON-SYNONYMOUS-CODING
OsMed15_1	LOC_Os04g03860	Os_chr04	1753537	[C/T]	NON-SYNONYMOUS-CODING
OsMed15_1	LOC_Os04g03860	Os_chr04	1753510	[T/G]	NON-SYNONYMOUS-CODING
OsMed15_1	LOC_Os04g03860	Os_chr04	1753470	[C/T]	NON-SYNONYMOUS-CODING
OsMed15_1	LOC_Os04g03860	Os_chr04	1753444	[C/T]	NON-SYNONYMOUS-CODING
OsMed15_1	LOC_Os04g03860	Os_chr04	1753415	[T/G]	NON-SYNONYMOUS-CODING
OsMed15_1	LOC_Os04g03860	Os_chr04	1753380	[G/A]	NON-SYNONYMOUS-CODING
OsMed15_1	LOC_Os04g03860	Os_chr04	1753377	[C/T]	NON-SYNONYMOUS-CODING
OsMed15_1	LOC_Os04g03860	Os_chr04	1753318	[C/T]	NON-SYNONYMOUS-CODING
OsMed15_1	LOC_Os04g03860	Os_chr04	1753299	[A/C]	NON-SYNONYMOUS-CODING
OsMed15_1	LOC_Os04g03860	Os_chr04	1753240	[T/C]	NON-SYNONYMOUS-CODING
OsMed15_1	LOC_Os04g03860	Os_chr04	1753148	[G/T]	NON-SYNONYMOUS-CODING
OsMed15_1	LOC_Os04g03860	Os_chr04	1752774	[G/T]	NON-SYNONYMOUS-CODING
OsMed15_1	LOC_Os04g03860	Os_chr04	1752769	[C/T]	NON-SYNONYMOUS-CODING
OsMed15_1	LOC_Os04g03860	Os_chr04	1752689	[A/G]	NON-SYNONYMOUS-CODING
OsMed15_1	LOC_Os04g03860	Os_chr04	1752584	[G/A]	NON-SYNONYMOUS-CODING
OsMed15_1	LOC_Os04g03860	Os_chr04	1752583	[C/T]	NON-SYNONYMOUS-CODING
OsMed15_1	LOC_Os04g03860	Os_chr04	1752580	[C/T]	NON-SYNONYMOUS-CODING
OsMed15_1	LOC_Os04g03860	Os_chr04	1752574	[G/A]	NON-SYNONYMOUS-CODING
OsMed15_1	LOC_Os04g03860	Os_chr04	1752198	[C/G]	NON-SYNONYMOUS-CODING
OsMed15_1	LOC_Os04g03860	Os_chr04	1752043	[G/C]	NON-SYNONYMOUS-CODING
OsMed15_1	LOC_Os04g03860	Os_chr04	1752025	[C/G]	NON-SYNONYMOUS-CODING
OsMed15_1	LOC_Os04g03860	Os_chr04	1751996	[C/T]	NON-SYNONYMOUS-CODING
OsMed15_1	LOC_Os04g03860	Os_chr04	1751987	[C/A]	NON-SYNONYMOUS-CODING
OsMed15_1	LOC_Os04g03860	Os_chr04	1751950	[C/A]	NON-SYNONYMOUS-CODING
OsMed15_1	LOC_Os04g03860	Os_chr04	1751944	[G/A]	NON-SYNONYMOUS-CODING
OsMed15_1	LOC_Os04g03860	Os_chr04	1751936	[G/A]	NON-SYNONYMOUS-CODING
OsMed15_1	LOC_Os04g03860	Os_chr04	1751884	[A/G]	NON-SYNONYMOUS-CODING
OsMed15_1	LOC_Os04g03860	Os_chr04	1751804	[T/C]	NON-SYNONYMOUS-CODING
OsMed15_1	LOC_Os04g03860	Os_chr04	1751764	[C/A]	NON-SYNONYMOUS-CODING
OsMed15_1	LOC_Os04g03860	Os_chr04	1751748	[G/T]	NON-SYNONYMOUS-CODING
OsMed15_1	LOC_Os04g03860	Os_chr04	1751677	[G/A]	NON-SYNONYMOUS-CODING
OsMed15_1	LOC_Os04g03860	Os_chr04	1751560	[T/C]	NON-SYNONYMOUS-CODING
OsMed15_1	LOC_Os04g03860	Os_chr04	1751539	[G/A]	NON-SYNONYMOUS-CODING
OsMed15_1	LOC_Os04g03860	Os_chr04	1751536	[A/G]	NON-SYNONYMOUS-CODING

Mediator genes	MSU locus ID	Chromosomes	Physical Position (bp)	SNPs	Structural Annotation
OsMed15_1	LOC_Os04g03860	Os_chr04	1751500	[G/T]	NON-SYNONYMOUS-CODING
OsMed15_1	LOC_Os04g03860	Os_chr04	1751483	[A/G]	NON-SYNONYMOUS-CODING
OsMed15_1	LOC_Os04g03860	Os_chr04	1751468	[C/T]	NON-SYNONYMOUS-CODING
OsMed15_1	LOC_Os04g03860	Os_chr04	1751416	[G/A]	NON-SYNONYMOUS-CODING
OsMed15_1	LOC_Os04g03860	Os_chr04	1751383	[T/C]	NON-SYNONYMOUS-CODING
OsMed15_1	LOC_Os04g03860	Os_chr04	1750983	[G/T]	NON-SYNONYMOUS-CODING
OsMed15_1	LOC_Os04g03860	Os_chr04	1750199	[A/G]	NON-SYNONYMOUS-CODING
OsMed15_1	LOC_Os04g03860	Os_chr04	1750179	[T/C]	NON-SYNONYMOUS-CODING
OsMed15_1	LOC_Os04g03860	Os_chr04	1750170	[C/T]	NON-SYNONYMOUS-CODING
OsMed15_1	LOC_Os04g03860	Os_chr04	1750165	[G/A]	NON-SYNONYMOUS-CODING
OsMed15_1	LOC_Os04g03860	Os_chr04	1750112	[G/C]	NON-SYNONYMOUS-CODING
OsMed15_1	LOC_Os04g03860	Os_chr04	1750052	[A/T]	REGULATORY
OsMed15_1	LOC_Os04g03860	Os_chr04	1755128	[G/C]	REGULATORY
OsMed15_1	LOC_Os04g03860	Os_chr04	1755434	[T/C]	REGULATORY
OsMed15_1	LOC_Os04g03860	Os_chr04	1755423	[A/G]	REGULATORY
OsMed15_1	LOC_Os04g03860	Os_chr04	1755415	[C/T]	REGULATORY
OsMed15_1	LOC_Os04g03860	Os_chr04	1755404	[G/A]	REGULATORY
OsMed15_1	LOC_Os04g03860	Os_chr04	1755365	[T/C]	REGULATORY
OsMed15_1	LOC_Os04g03860	Os_chr04	1755341	[G/C]	REGULATORY
OsMed15_1	LOC_Os04g03860	Os_chr04	1755311	[A/T]	REGULATORY
OsMed15_1	LOC_Os04g03860	Os_chr04	1755284	[G/A]	REGULATORY
OsMed15_1	LOC_Os04g03860	Os_chr04	1755200	[T/C]	REGULATORY
OsMed15_1	LOC_Os04g03860	Os_chr04	1755181	[G/A]	REGULATORY
OsMed15_1	LOC_Os04g03860	Os_chr04	1755174	[C/G]	REGULATORY
OsMed15_1	LOC_Os04g03860	Os_chr04	1750027	[C/T]	REGULATORY
OsMed15_1	LOC_Os04g03860	Os_chr04	1749984	[T/C]	REGULATORY
OsMed15_1	LOC_Os04g03860	Os_chr04	1749967	[C/T]	REGULATORY
OsMed15_1	LOC_Os04g03860	Os_chr04	1749964	[G/A]	REGULATORY
OsMed15_1	LOC_Os04g03860	Os_chr04	1749955	[G/A]	REGULATORY
OsMed15_1	LOC_Os04g03860	Os_chr04	1749901	[T/C]	REGULATORY
OsMed15_1	LOC_Os04g03860	Os_chr04	1749838	[C/A]	REGULATORY
OsMed15_1	LOC_Os04g03860	Os_chr04	1749832	[A/G]	REGULATORY
OsMed15_1	LOC_Os04g03860	Os_chr04	1749819	[C/T]	REGULATORY
OsMed15_1	LOC_Os04g03860	Os_chr04	1749799	[G/C]	REGULATORY
OsMed15_1	LOC_Os04g03860	Os_chr04	1749785	[A/T]	REGULATORY
OsMed15_1	LOC_Os04g03860	Os_chr04	1749740	[C/T]	REGULATORY
OsMed15_1	LOC_Os04g03860	Os_chr04	1749720	[G/A]	REGULATORY
OsMed15_1	LOC_Os04g03860	Os_chr04	1749719	[C/T]	REGULATORY
OsMed15_1	LOC_Os04g03860	Os_chr04	1749710	[G/A]	REGULATORY
OsMed15_1	LOC_Os04g03860	Os_chr04	1749701	[T/C]	REGULATORY
OsMed15_1	LOC_Os04g03860	Os_chr04	1749693	[C/A]	REGULATORY
OsMed15_1	LOC_Os04g03860	Os_chr04	1749679	[G/A]	REGULATORY
OsMed15_1	LOC_Os04g03860	Os_chr04	1749668	[G/A]	REGULATORY
OsMed15_1	LOC_Os04g03860	Os_chr04	1749618	[C/A]	REGULATORY
OsMed15_1	LOC_Os04g03860	Os_chr04	1749599	[T/C]	REGULATORY
OsMed15_1	LOC_Os04g03860	Os_chr04	1749568	[C/T]	REGULATORY
OsMed15_1	LOC_Os04g03860	Os_chr04	1749553	[T/C]	REGULATORY

**Mediator genes**	**MSU locus ID**	**Chromosomes**	**Physical Position (bp)**	**SNPs**	**Structural Annotation**
OsMed15_1	LOC_Os04g03860	Os_chr04	1749551	[T/C]	REGULATORY
OsMed15_1	LOC_Os04g03860	Os_chr04	1749540	[C/T]	REGULATORY
OsMed15_1	LOC_Os04g03860	Os_chr04	1749533	[C/T]	REGULATORY
OsMed15_1	LOC_Os04g03860	Os_chr04	1749515	[G/A]	REGULATORY
OsMed15_1	LOC_Os04g03860	Os_chr04	1749506	[C/T]	REGULATORY
OsMed15_1	LOC_Os04g03860	Os_chr04	1749501	[C/T]	REGULATORY
OsMed15_1	LOC_Os04g03860	Os_chr04	1749500	[A/G]	REGULATORY
OsMed15_1	LOC_Os04g03860	Os_chr04	1749486	[G/A]	REGULATORY
OsMed15_1	LOC_Os04g03860	Os_chr04	1749460	[A/T]	REGULATORY
OsMed15_1	LOC_Os04g03860	Os_chr04	1749457	[G/A]	REGULATORY
OsMed15_1	LOC_Os04g03860	Os_chr04	1749451	[T/A]	REGULATORY
OsMed15_1	LOC_Os04g03860	Os_chr04	1749423	[C/T]	REGULATORY
OsMed15_1	LOC_Os04g03860	Os_chr04	1749418	[A/C]	REGULATORY
OsMed15_1	LOC_Os04g03860	Os_chr04	1749414	[C/T]	REGULATORY
OsMed15_1	LOC_Os04g03860	Os_chr04	1749413	[A/G]	REGULATORY
OsMed15_1	LOC_Os04g03860	Os_chr04	1749399	[C/T]	REGULATORY
OsMed15_1	LOC_Os04g03860	Os_chr04	1749389	[A/C]	REGULATORY
OsMed15_1	LOC_Os04g03860	Os_chr04	1749386	[G/T]	REGULATORY
OsMed15_1	LOC_Os04g03860	Os_chr04	1749376	[C/G]	REGULATORY
OsMed15_1	LOC_Os04g03860	Os_chr04	1749356	[T/C]	REGULATORY
OsMed15_1	LOC_Os04g03860	Os_chr04	1749347	[T/A]	REGULATORY
OsMed15_1	LOC_Os04g03860	Os_chr04	1749330	[C/T]	REGULATORY
OsMed15_1	LOC_Os04g03860	Os_chr04	1749323	[G/A]	REGULATORY
OsMed15_1	LOC_Os04g03860	Os_chr04	1749319	[T/C]	REGULATORY
OsMed15_1	LOC_Os04g03860	Os_chr04	1749306	[A/C]	REGULATORY
OsMed15_1	LOC_Os04g03860	Os_chr04	1749277	[G/A]	REGULATORY
OsMed15_1	LOC_Os04g03860	Os_chr04	1749261	[A/T]	REGULATORY
OsMed15_1	LOC_Os04g03860	Os_chr04	1749253	[T/A]	REGULATORY
OsMed15_1	LOC_Os04g03860	Os_chr04	1749242	[A/T]	REGULATORY
OsMed15_1	LOC_Os04g03860	Os_chr04	1749241	[A/T]	REGULATORY
OsMed15_1	LOC_Os04g03860	Os_chr04	1749199	[A/G]	REGULATORY
OsMed15_1	LOC_Os04g03860	Os_chr04	1749151	[A/G]	REGULATORY
OsMed15_1	LOC_Os04g03860	Os_chr04	1749131	[T/C]	REGULATORY
OsMed15_1	LOC_Os04g03860	Os_chr04	1749094	[C/T]	REGULATORY
OsMed15_1	LOC_Os04g03860	Os_chr04	1749077	[G/A]	REGULATORY
OsMed15_1	LOC_Os04g03860	Os_chr04	1749059	[G/A]	REGULATORY
OsMed15_1	LOC_Os04g03860	Os_chr04	1749049	[C/T]	REGULATORY
OsMed15_1	LOC_Os04g03860	Os_chr04	1755089	[A/G]	SYNONYMOUS-CODING
OsMed15_1	LOC_Os04g03860	Os_chr04	1755083	[C/T]	SYNONYMOUS-CODING
OsMed15_1	LOC_Os04g03860	Os_chr04	1755074	[T/C]	SYNONYMOUS-CODING
OsMed15_1	LOC_Os04g03860	Os_chr04	1755014	[C/T]	SYNONYMOUS-CODING
OsMed15_1	LOC_Os04g03860	Os_chr04	1754975	[C/T]	SYNONYMOUS-CODING
OsMed15_1	LOC_Os04g03860	Os_chr04	1754966	[C/A]	SYNONYMOUS-CODING
OsMed15_1	LOC_Os04g03860	Os_chr04	1754963	[C/T]	SYNONYMOUS-CODING
OsMed15_1	LOC_Os04g03860	Os_chr04	1754798	[G/A]	SYNONYMOUS-CODING
OsMed15_1	LOC_Os04g03860	Os_chr04	1754789	[G/A]	SYNONYMOUS-CODING
OsMed15_1	LOC_Os04g03860	Os_chr04	1754597	[T/C]	SYNONYMOUS-CODING

Mediator genes	MSU locus ID	Chromosomes	Physical Position (bp)	SNPs	Structural Annotation
OsMed15_1	LOC_Os04g03860	Os_chr04	1754570	[C/T]	SYNONYMOUS-CODING
OsMed15_1	LOC_Os04g03860	Os_chr04	1754528	[T/A]	SYNONYMOUS-CODING
OsMed15_1	LOC_Os04g03860	Os_chr04	1754483	[A/C]	SYNONYMOUS-CODING
OsMed15_1	LOC_Os04g03860	Os_chr04	1754288	[G/A]	SYNONYMOUS-CODING
OsMed15_1	LOC_Os04g03860	Os_chr04	1754198	[T/G]	SYNONYMOUS-CODING
OsMed15_1	LOC_Os04g03860	Os_chr04	1753865	[T/G]	SYNONYMOUS-CODING
OsMed15_1	LOC_Os04g03860	Os_chr04	1753850	[G/T]	SYNONYMOUS-CODING
OsMed15_1	LOC_Os04g03860	Os_chr04	1753547	[C/A]	SYNONYMOUS-CODING
OsMed15_1	LOC_Os04g03860	Os_chr04	1753499	[C/G]	SYNONYMOUS-CODING
OsMed15_1	LOC_Os04g03860	Os_chr04	1753400	[T/C]	SYNONYMOUS-CODING
OsMed15_1	LOC_Os04g03860	Os_chr04	1753319	[A/G]	SYNONYMOUS-CODING
OsMed15_1	LOC_Os04g03860	Os_chr04	1753298	[T/C]	SYNONYMOUS-CODING
OsMed15_1	LOC_Os04g03860	Os_chr04	1753256	[T/A]	SYNONYMOUS-CODING
OsMed15_1	LOC_Os04g03860	Os_chr04	1753223	[T/C]	SYNONYMOUS-CODING
OsMed15_1	LOC_Os04g03860	Os_chr04	1753187	[A/C]	SYNONYMOUS-CODING
OsMed15_1	LOC_Os04g03860	Os_chr04	1752777	[A/T]	SYNONYMOUS-CODING
OsMed15_1	LOC_Os04g03860	Os_chr04	1752693	[G/A]	SYNONYMOUS-CODING
OsMed15_1	LOC_Os04g03860	Os_chr04	1752455	[A/G]	SYNONYMOUS-CODING
OsMed15_1	LOC_Os04g03860	Os_chr04	1752243	[G/A]	SYNONYMOUS-CODING
OsMed15_1	LOC_Os04g03860	Os_chr04	1752216	[G/C]	SYNONYMOUS-CODING
OsMed15_1	LOC_Os04g03860	Os_chr04	1751997	[G/A]	SYNONYMOUS-CODING
OsMed15_1	LOC_Os04g03860	Os_chr04	1751973	[A/C]	SYNONYMOUS-CODING
OsMed15_1	LOC_Os04g03860	Os_chr04	1751961	[G/A]	SYNONYMOUS-CODING
OsMed15_1	LOC_Os04g03860	Os_chr04	1751868	[G/A]	SYNONYMOUS-CODING
OsMed15_1	LOC_Os04g03860	Os_chr04	1751814	[G/A]	SYNONYMOUS-CODING
OsMed15_1	LOC_Os04g03860	Os_chr04	1751808	[G/A]	SYNONYMOUS-CODING
OsMed15_1	LOC_Os04g03860	Os_chr04	1751790	[A/G]	SYNONYMOUS-CODING
OsMed15_1	LOC_Os04g03860	Os_chr04	1751667	[T/C]	SYNONYMOUS-CODING
OsMed15_1	LOC_Os04g03860	Os_chr04	1751628	[T/A]	SYNONYMOUS-CODING
OsMed15_1	LOC_Os04g03860	Os_chr04	1751454	[C/T]	SYNONYMOUS-CODING
OsMed15_1	LOC_Os04g03860	Os_chr04	1751442	[A/G]	SYNONYMOUS-CODING
OsMed15_1	LOC_Os04g03860	Os_chr04	1751433	[C/T]	SYNONYMOUS-CODING
OsMed15_1	LOC_Os04g03860	Os_chr04	1751409	[A/G]	SYNONYMOUS-CODING
OsMed15_1	LOC_Os04g03860	Os_chr04	1751337	[A/G]	SYNONYMOUS-CODING
OsMed15_1	LOC_Os04g03860	Os_chr04	1750986	[C/T]	SYNONYMOUS-CODING
OsMed15_1	LOC_Os04g03860	Os_chr04	1750968	[T/G]	SYNONYMOUS-CODING
OsMed15_1	LOC_Os04g03860	Os_chr04	1750150	[C/T]	SYNONYMOUS-CODING
OsMed15_1	LOC_Os04g03860	Os_chr04	1750144	[C/T]	SYNONYMOUS-CODING
OsMed15_2	LOC_Os08g45080	Os_chr08	28301886	[G/A]	INTRON
OsMed15_2	LOC_Os08g45080	Os_chr08	28301765	[G/A]	INTRON
OsMed15_2	LOC_Os08g45080	Os_chr08	28301309	[G/A]	INTRON
OsMed15_2	LOC_Os08g45080	Os_chr08	28300846	[C/A]	INTRON
OsMed15_2	LOC_Os08g45080	Os_chr08	28300620	[A/T]	INTRON
OsMed15_2	LOC_Os08g45080	Os_chr08	28299532	[C/A]	INTRON
OsMed15_2	LOC_Os08g45080	Os_chr08	28299339	[T/G]	INTRON
OsMed15_2	LOC_Os08g45080	Os_chr08	28299324	[G/T]	INTRON
OsMed15_2	LOC_Os08g45080	Os_chr08	28299288	[G/A]	INTRON

**Mediator genes**	**MSU locus ID**	**Chromosomes**	**Physical Position (bp)**	**SNPs**	**Structural Annotation**
OsMed15_2	LOC_Os08g45080	Os_chr08	28299215	[C/G]	INTRON
OsMed15_2	LOC_Os08g45080	Os_chr08	28299143	[T/A]	INTRON
OsMed15_2	LOC_Os08g45080	Os_chr08	28297623	[A/G]	INTRON
OsMed15_2	LOC_Os08g45080	Os_chr08	28297413	[A/G]	INTRON
OsMed15_2	LOC_Os08g45080	Os_chr08	28297077	[A/T]	INTRON
OsMed15_2	LOC_Os08g45080	Os_chr08	28296985	[G/A]	INTRON
OsMed15_2	LOC_Os08g45080	Os_chr08	28296692	[G/A]	INTRON
OsMed15_2	LOC_Os08g45080	Os_chr08	28296686	[A/G]	INTRON
OsMed15_2	LOC_Os08g45080	Os_chr08	28296493	[T/G]	INTRON
OsMed15_2	LOC_Os08g45080	Os_chr08	28296401	[A/C]	INTRON
OsMed15_2	LOC_Os08g45080	Os_chr08	28296369	[C/A]	INTRON
OsMed15_2	LOC_Os08g45080	Os_chr08	28296274	[A/C]	INTRON
OsMed15_2	LOC_Os08g45080	Os_chr08	28296258	[T/G]	INTRON
OsMed15_2	LOC_Os08g45080	Os_chr08	28294599	[A/G]	INTRON
OsMed15_2	LOC_Os08g45080	Os_chr08	28294542	[C/T]	INTRON
OsMed15_2	LOC_Os08g45080	Os_chr08	28294446	[T/C]	INTRON
OsMed15_2	LOC_Os08g45080	Os_chr08	28294391	[G/A]	INTRON
OsMed15_2	LOC_Os08g45080	Os_chr08	28294098	[G/T]	INTRON
OsMed15_2	LOC_Os08g45080	Os_chr08	28293425	[G/T]	INTRON
OsMed15_2	LOC_Os08g45080	Os_chr08	28302391	[G/A]	NON-SYNONYMOUS-CODING
OsMed15_2	LOC_Os08g45080	Os_chr08	28298893	[G/T]	NON-SYNONYMOUS-CODING
OsMed15_2	LOC_Os08g45080	Os_chr08	28296090	[A/T]	NON-SYNONYMOUS-CODING
OsMed15_2	LOC_Os08g45080	Os_chr08	28296070	[C/T]	NON-SYNONYMOUS-CODING
OsMed15_2	LOC_Os08g45080	Os_chr08	28295962	[T/C]	NON-SYNONYMOUS-CODING
OsMed15_2	LOC_Os08g45080	Os_chr08	28295611	[C/T]	NON-SYNONYMOUS-CODING
OsMed15_2	LOC_Os08g45080	Os_chr08	28295573	[T/C]	NON-SYNONYMOUS-CODING
OsMed15_2	LOC_Os08g45080	Os_chr08	28295193	[G/C]	NON-SYNONYMOUS-CODING
OsMed15_2	LOC_Os08g45080	Os_chr08	28294801	[G/T]	NON-SYNONYMOUS-CODING
OsMed15_2	LOC_Os08g45080	Os_chr08	28292523	[G/T]	REGULATORY
OsMed15_2	LOC_Os08g45080	Os_chr08	28292455	[G/A]	REGULATORY
OsMed15_2	LOC_Os08g45080	Os_chr08	28303580	[G/A]	REGULATORY
OsMed15_2	LOC_Os08g45080	Os_chr08	28303382	[C/T]	REGULATORY
OsMed15_2	LOC_Os08g45080	Os_chr08	28303346	[C/T]	REGULATORY
OsMed15_2	LOC_Os08g45080	Os_chr08	28303272	[G/C]	REGULATORY
OsMed15_2	LOC_Os08g45080	Os_chr08	28303241	[C/T]	REGULATORY
OsMed15_2	LOC_Os08g45080	Os_chr08	28303222	[G/A]	REGULATORY
OsMed15_2	LOC_Os08g45080	Os_chr08	28303039	[C/T]	REGULATORY
OsMed15_2	LOC_Os08g45080	Os_chr08	28303021	[C/A]	REGULATORY
OsMed15_2	LOC_Os08g45080	Os_chr08	28302838	[C/A]	REGULATORY
OsMed15_2	LOC_Os08g45080	Os_chr08	28302790	[A/G]	REGULATORY
OsMed15_2	LOC_Os08g45080	Os_chr08	28302749	[C/T]	REGULATORY
OsMed15_2	LOC_Os08g45080	Os_chr08	28302685	[T/G]	REGULATORY
OsMed15_2	LOC_Os08g45080	Os_chr08	28298097	[C/T]	SYNONYMOUS-CODING
OsMed15_2	LOC_Os08g45080	Os_chr08	28295397	[C/T]	SYNONYMOUS-CODING
OsMed15_2	LOC_Os08g45080	Os_chr08	28295283	[G/A]	SYNONYMOUS-CODING
OsMed15_2	LOC_Os08g45080	Os_chr08	28293691	[A/G]	SYNONYMOUS-CODING
OsMed16_1	LOC_Os10g35560	Os_chr10	18953375	[A/T]	INTRON

**Mediator genes**	**MSU locus ID**	**Chromosomes**	**Physical Position (bp)**	**SNPs**	**Structural Annotation**
OsMed16_1	LOC_Os10g35560	Os_chr10	18953353	[G/T]	INTRON
OsMed16_1	LOC_Os10g35560	Os_chr10	18952770	[C/T]	INTRON
OsMed16_1	LOC_Os10g35560	Os_chr10	18952667	[A/T]	INTRON
OsMed16_1	LOC_Os10g35560	Os_chr10	18952534	[C/A]	INTRON
OsMed16_1	LOC_Os10g35560	Os_chr10	18952191	[C/T]	INTRON
OsMed16_1	LOC_Os10g35560	Os_chr10	18952078	[C/A]	INTRON
OsMed16_1	LOC_Os10g35560	Os_chr10	18951904	[G/A]	INTRON
OsMed16_1	LOC_Os10g35560	Os_chr10	18951224	[A/T]	INTRON
OsMed16_1	LOC_Os10g35560	Os_chr10	18950953	[G/A]	INTRON
OsMed16_1	LOC_Os10g35560	Os_chr10	18950874	[C/T]	INTRON
OsMed16_1	LOC_Os10g35560	Os_chr10	18950792	[C/G]	INTRON
OsMed16_1	LOC_Os10g35560	Os_chr10	18950740	[G/A]	INTRON
OsMed16_1	LOC_Os10g35560	Os_chr10	18950667	[G/A]	INTRON
OsMed16_1	LOC_Os10g35560	Os_chr10	18950286	[G/C]	INTRON
OsMed16_1	LOC_Os10g35560	Os_chr10	18950209	[T/C]	INTRON
OsMed16_1	LOC_Os10g35560	Os_chr10	18950138	[A/T]	INTRON
OsMed16_1	LOC_Os10g35560	Os_chr10	18950136	[G/T]	INTRON
OsMed16_1	LOC_Os10g35560	Os_chr10	18950059	[G/T]	INTRON
OsMed16_1	LOC_Os10g35560	Os_chr10	18950002	[C/T]	INTRON
OsMed16_1	LOC_Os10g35560	Os_chr10	18949599	[G/C]	INTRON
OsMed16_1	LOC_Os10g35560	Os_chr10	18949541	[C/A]	INTRON
OsMed16_1	LOC_Os10g35560	Os_chr10	18949231	[T/C]	INTRON
OsMed16_1	LOC_Os10g35560	Os_chr10	18948997	[T/C]	INTRON
OsMed16_1	LOC_Os10g35560	Os_chr10	18948162	[G/A]	INTRON
OsMed16_1	LOC_Os10g35560	Os_chr10	18948039	[T/C]	INTRON
OsMed16_1	LOC_Os10g35560	Os_chr10	18947903	[C/T]	INTRON
OsMed16_1	LOC_Os10g35560	Os_chr10	18946985	[C/G]	INTRON
OsMed16_1	LOC_Os10g35560	Os_chr10	18946414	[C/T]	INTRON
OsMed16_1	LOC_Os10g35560	Os_chr10	18946254	[T/A]	INTRON
OsMed16_1	LOC_Os10g35560	Os_chr10	18946240	[A/C]	INTRON
OsMed16_1	LOC_Os10g35560	Os_chr10	18946203	[C/A]	INTRON
OsMed16_1	LOC_Os10g35560	Os_chr10	18946139	[T/G]	INTRON
OsMed16_1	LOC_Os10g35560	Os_chr10	18945730	[T/G]	INTRON
OsMed16_1	LOC_Os10g35560	Os_chr10	18945604	[C/A]	INTRON
OsMed16_1	LOC_Os10g35560	Os_chr10	18953637	[T/G]	INTRON
OsMed16_1	LOC_Os10g35560	Os_chr10	18953601	[G/A]	INTRON
OsMed16_1	LOC_Os10g35560	Os_chr10	18953136	[T/A]	NON-SYNONYMOUS-CODING
OsMed16_1	LOC_Os10g35560	Os_chr10	18953068	[T/C]	NON-SYNONYMOUS-CODING
OsMed16_1	LOC_Os10g35560	Os_chr10	18952302	[G/A]	NON-SYNONYMOUS-CODING
OsMed16_1	LOC_Os10g35560	Os_chr10	18951789	[G/A]	NON-SYNONYMOUS-CODING
OsMed16_1	LOC_Os10g35560	Os_chr10	18951385	[G/A]	NON-SYNONYMOUS-CODING
OsMed16_1	LOC_Os10g35560	Os_chr10	18950508	[A/G]	NON-SYNONYMOUS-CODING
OsMed16_1	LOC_Os10g35560	Os_chr10	18948514	[C/T]	NON-SYNONYMOUS-CODING
OsMed16_1	LOC_Os10g35560	Os_chr10	18947740	[G/A]	NON-SYNONYMOUS-CODING
OsMed16_1	LOC_Os10g35560	Os_chr10	18947704	[A/C]	NON-SYNONYMOUS-CODING
OsMed16_1	LOC_Os10g35560	Os_chr10	18947196	[G/A]	NON-SYNONYMOUS-CODING
OsMed16_1	LOC_Os10g35560	Os_chr10	18953944	[A/G]	REGULATORY

**Mediator genes**	**MSU locus ID**	**Chromosomes**	**Physical Position (bp)**	**SNPs**	**Structural Annotation**
OsMed16_1	LOC_Os10g35560	Os_chr10	18953856	[T/C]	REGULATORY
OsMed16_1	LOC_Os10g35560	Os_chr10	18953844	[A/C]	REGULATORY
OsMed16_1	LOC_Os10g35560	Os_chr10	18953288	[C/T]	SYNONYMOUS-CODING
OsMed16_1	LOC_Os10g35560	Os_chr10	18953057	[A/T]	SYNONYMOUS-CODING
OsMed16_1	LOC_Os10g35560	Os_chr10	18952391	[C/T]	SYNONYMOUS-CODING
OsMed16_1	LOC_Os10g35560	Os_chr10	18952298	[A/C]	SYNONYMOUS-CODING
OsMed16_1	LOC_Os10g35560	Os_chr10	18951650	[T/C]	SYNONYMOUS-CODING
OsMed16_1	LOC_Os10g35560	Os_chr10	18950489	[G/A]	SYNONYMOUS-CODING
OsMed16_1	LOC_Os10g35560	Os_chr10	18949061	[A/G]	SYNONYMOUS-CODING
OsMed16_1	LOC_Os10g35560	Os_chr10	18947200	[A/G]	SYNONYMOUS-CODING
OsMed16_1	LOC_Os10g35560	Os_chr10	18944275	[A/T]	REGULATORY
OsMed16_1	LOC_Os10g35560	Os_chr10	18944253	[G/C]	REGULATORY
OsMed16_1	LOC_Os10g35560	Os_chr10	18944223	[A/T]	REGULATORY
OsMed16_1	LOC_Os10g35560	Os_chr10	18944193	[G/A]	REGULATORY
OsMed16_1	LOC_Os10g35560	Os_chr10	18944176	[A/G]	REGULATORY
OsMed16_1	LOC_Os10g35560	Os_chr10	18944167	[G/A]	REGULATORY
OsMed16_1	LOC_Os10g35560	Os_chr10	18944166	[C/T]	REGULATORY
OsMed16_1	LOC_Os10g35560	Os_chr10	18944159	[G/A]	REGULATORY
OsMed16_1	LOC_Os10g35560	Os_chr10	18944127	[A/G]	REGULATORY
OsMed16_1	LOC_Os10g35560	Os_chr10	18944122	[G/T]	REGULATORY
OsMed16_1	LOC_Os10g35560	Os_chr10	18944099	[T/C]	REGULATORY
OsMed16_1	LOC_Os10g35560	Os_chr10	18944071	[G/A]	REGULATORY
OsMed16_1	LOC_Os10g35560	Os_chr10	18944070	[T/G]	REGULATORY
OsMed16_1	LOC_Os10g35560	Os_chr10	18944021	[G/A]	REGULATORY
OsMed16_1	LOC_Os10g35560	Os_chr10	18944017	[A/T]	REGULATORY
OsMed16_1	LOC_Os10g35560	Os_chr10	18943990	[C/T]	REGULATORY
OsMed16_1	LOC_Os10g35560	Os_chr10	18943935	[C/T]	REGULATORY
OsMed16_1	LOC_Os10g35560	Os_chr10	18943893	[A/G]	REGULATORY
OsMed16_1	LOC_Os10g35560	Os_chr10	18943825	[G/A]	REGULATORY
OsMed16_1	LOC_Os10g35560	Os_chr10	18943630	[G/A]	REGULATORY
OsMed16_1	LOC_Os10g35560	Os_chr10	18943547	[C/A]	REGULATORY
OsMed16_1	LOC_Os10g35560	Os_chr10	18943468	[G/T]	REGULATORY
OsMed17_1	LOC_Os12g44140	Os_chr12	27348405	[C/T]	INTRON
OsMed17_1	LOC_Os12g44140	Os_chr12	27348273	[G/T]	INTRON
OsMed17_1	LOC_Os12g44140	Os_chr12	27348157	[C/T]	INTRON
OsMed17_1	LOC_Os12g44140	Os_chr12	27348147	[T/A]	INTRON
OsMed17_1	LOC_Os12g44140	Os_chr12	27347988	[C/T]	INTRON
OsMed17_1	LOC_Os12g44140	Os_chr12	27346916	[C/T]	INTRON
OsMed17_1	LOC_Os12g44140	Os_chr12	27346805	[A/G]	INTRON
OsMed17_1	LOC_Os12g44140	Os_chr12	27346347	[C/T]	INTRON
OsMed17_1	LOC_Os12g44140	Os_chr12	27346046	[G/A]	INTRON
OsMed17_1	LOC_Os12g44140	Os_chr12	27347597	[C/T]	NON-SYNONYMOUS-CODING
OsMed17_1	LOC_Os12g44140	Os_chr12	27347155	[G/A]	NON-SYNONYMOUS-CODING
OsMed17_1	LOC_Os12g44140	Os_chr12	27346498	[T/C]	NON-SYNONYMOUS-CODING
OsMed17_1	LOC_Os12g44140	Os_chr12	27345321	[C/T]	REGULATORY
OsMed17_1	LOC_Os12g44140	Os_chr12	27345439	[T/C]	REGULATORY
OsMed17_1	LOC_Os12g44140	Os_chr12	27345307	[A/C]	REGULATORY

Mediator genes	MSU locus ID	Chromosomes	Physical Position (bp)	SNPs	Structural Annotation
OsMed17_1	LOC_Os12g44140	Os_chr12	27345222	[A/C]	REGULATORY
OsMed17_1	LOC_Os12g44140	Os_chr12	27345141	[T/G]	REGULATORY
OsMed17_1	LOC_Os12g44140	Os_chr12	27345105	[A/C]	REGULATORY
OsMed17_1	LOC_Os12g44140	Os_chr12	27344905	[G/A]	REGULATORY
OsMed17_1	LOC_Os12g44140	Os_chr12	27344757	[A/C]	REGULATORY
OsMed17_1	LOC_Os12g44140	Os_chr12	27344734	[C/T]	REGULATORY
OsMed17_1	LOC_Os12g44140	Os_chr12	27344710	[C/T]	REGULATORY
OsMed17_1	LOC_Os12g44140	Os_chr12	27344439	[G/A]	REGULATORY
OsMed17_1	LOC_Os12g44140	Os_chr12	27348569	[G/T]	SYNONYMOUS-CODING
OsMed17_1	LOC_Os12g44140	Os_chr12	27348557	[T/C]	SYNONYMOUS-CODING
OsMed17_1	LOC_Os12g44140	Os_chr12	27347328	[C/T]	SYNONYMOUS-CODING
OsMed17_1	LOC_Os12g44140	Os_chr12	27345765	[C/T]	SYNONYMOUS-CODING
OsMed17_1	LOC_Os12g44140	Os_chr12	27345762	[G/A]	SYNONYMOUS-CODING
OsMed17_1	LOC_Os12g44140	Os_chr12	27345666	[C/A]	SYNONYMOUS-CODING
OsMed18_1	LOC_Os02g10050	Os_chr02	5230471	[T/C]	INTRON
OsMed18_1	LOC_Os02g10050	Os_chr02	5230122	[G/A]	INTRON
OsMed18_1	LOC_Os02g10050	Os_chr02	5230060	[C/A]	INTRON
OsMed18_1	LOC_Os02g10050	Os_chr02	5229794	[C/T]	INTRON
OsMed18_1	LOC_Os02g10050	Os_chr02	5229681	[A/G]	INTRON
OsMed18_1	LOC_Os02g10050	Os_chr02	5229638	[T/C]	INTRON
OsMed18_1	LOC_Os02g10050	Os_chr02	5229478	[T/C]	INTRON
OsMed18_1	LOC_Os02g10050	Os_chr02	5229462	[G/A]	INTRON
OsMed18_1	LOC_Os02g10050	Os_chr02	5228954	[G/A]	INTRON
OsMed18_1	LOC_Os02g10050	Os_chr02	5231082	[G/A]	NON-SYNONYMOUS-CODING
OsMed18_1	LOC_Os02g10050	Os_chr02	5230741	[G/A]	NON-SYNONYMOUS-CODING
OsMed18_1	LOC_Os02g10050	Os_chr02	5230709	[G/A]	NON-SYNONYMOUS-CODING
OsMed18_1	LOC_Os02g10050	Os_chr02	5228823	[G/A]	REGULATORY
OsMed18_1	LOC_Os02g10050	Os_chr02	5231256	[A/G]	REGULATORY
OsMed18_1	LOC_Os02g10050	Os_chr02	5231185	[T/C]	REGULATORY
OsMed18_1	LOC_Os02g10050	Os_chr02	5231166	[G/T]	REGULATORY
OsMed18_1	LOC_Os02g57590	Os_chr02	5228677	[C/A]	REGULATORY
OsMed18_1	LOC_Os02g57590	Os_chr02	5228633	[C/A]	REGULATORY
OsMed18_1	LOC_Os02g57590	Os_chr02	5228547	[G/T]	REGULATORY
OsMed18_1	LOC_Os02g57590	Os_chr02	5228208	[G/A]	REGULATORY
OsMed18_1	LOC_Os02g57590	Os_chr02	5228147	[A/G]	REGULATORY
OsMed18_1	LOC_Os02g57590	Os_chr02	5228086	[G/A]	REGULATORY
OsMed18_1	LOC_Os02g57590	Os_chr02	5228046	[C/A]	REGULATORY
OsMed18_1	LOC_Os02g57590	Os_chr02	5228040	[G/A]	REGULATORY
OsMed18_1	LOC_Os02g57590	Os_chr02	5228034	[G/A]	REGULATORY
OsMed18_1	LOC_Os02g57590	Os_chr02	5228016	[G/A]	REGULATORY
OsMed18_1	LOC_Os02g10050	Os_chr02	5231036	[G/A]	SYNONYMOUS-CODING
OsMed18_1	LOC_Os02g10050	Os_chr02	5230869	[C/T]	SYNONYMOUS-CODING
OsMed18_1	LOC_Os02g10050	Os_chr02	5230642	[T/C]	SYNONYMOUS-CODING
OsMed19_1	LOC_Os03g44010	Os_chr03	24729679	[G/A]	INTRON
OsMed19_1	LOC_Os03g44010	Os_chr03	24729295	[A/G]	INTRON
OsMed19_1	LOC_Os03g44010	Os_chr03	24728994	[T/C]	INTRON
OsMed19_1	LOC_Os03g44010	Os_chr03	24728982	[T/G]	INTRON

Mediator genes	MSU locus ID	Chromosomes	Physical Position (bp)	SNPs	Structural Annotation
OsMed19_1	LOC_Os03g44010	Os_chr03	24728915	[G/A]	INTRON
OsMed19_1	LOC_Os03g44010	Os_chr03	24728836	[A/G]	INTRON
OsMed19_1	LOC_Os03g44010	Os_chr03	24728499	[A/G]	INTRON
OsMed19_1	LOC_Os03g44010	Os_chr03	24728417	[C/T]	INTRON
OsMed19_1	LOC_Os03g44010	Os_chr03	24728351	[G/A]	INTRON
OsMed19_1	LOC_Os03g44010	Os_chr03	24728254	[A/T]	INTRON
OsMed19_1	LOC_Os03g44010	Os_chr03	24728234	[A/G]	INTRON
OsMed19_1	LOC_Os03g44010	Os_chr03	24727787	[T/C]	INTRON
OsMed19_1	LOC_Os03g44010	Os_chr03	24727757	[C/T]	INTRON
OsMed19_1	LOC_Os03g44010	Os_chr03	24727432	[C/T]	INTRON
OsMed19_1	LOC_Os03g44010	Os_chr03	24727385	[T/C]	INTRON
OsMed19_1	LOC_Os03g44010	Os_chr03	24726719	[A/G]	INTRON
OsMed19_1	LOC_Os03g44010	Os_chr03	24726214	[T/C]	INTRON
OsMed19_1	LOC_Os03g44010	Os_chr03	24725904	[T/A]	INTRON
OsMed19_1	LOC_Os03g44010	Os_chr03	24727055	[G/A]	NON-SYNONYMOUS-CODING
OsMed19_1	LOC_Os03g44010	Os_chr03	24725593	[C/T]	REGULATORY
OsMed19_1	LOC_Os03g44010	Os_chr03	24725526	[C/T]	REGULATORY
OsMed19_1	LOC_Os03g44010	Os_chr03	24730241	[T/C]	REGULATORY
OsMed19_1	LOC_Os03g44010	Os_chr03	24725357	[A/G]	REGULATORY
OsMed19_1	LOC_Os03g44010	Os_chr03	24725219	[A/G]	REGULATORY
OsMed19_1	LOC_Os03g44010	Os_chr03	24725171	[C/T]	REGULATORY
OsMed19_1	LOC_Os03g44010	Os_chr03	24725145	[T/C]	REGULATORY
OsMed19_1	LOC_Os03g44010	Os_chr03	24725105	[G/A]	REGULATORY
OsMed19_1	LOC_Os03g44010	Os_chr03	24725093	[T/C]	REGULATORY
OsMed19_1	LOC_Os03g44010	Os_chr03	24725078	[C/T]	REGULATORY
OsMed19_1	LOC_Os03g44010	Os_chr03	24725026	[C/G]	REGULATORY
OsMed19_1	LOC_Os03g44010	Os_chr03	24725012	[G/A]	REGULATORY
OsMed19_1	LOC_Os03g44010	Os_chr03	24724979	[C/T]	REGULATORY
OsMed19_1	LOC_Os03g44010	Os_chr03	24724932	[C/T]	REGULATORY
OsMed19_1	LOC_Os03g44010	Os_chr03	24724926	[G/T]	REGULATORY
OsMed19_1	LOC_Os03g44010	Os_chr03	24724899	[C/T]	REGULATORY
OsMed19_1	LOC_Os03g44010	Os_chr03	24724885	[C/T]	REGULATORY
OsMed19_1	LOC_Os03g44010	Os_chr03	24724861	[A/G]	REGULATORY
OsMed19_1	LOC_Os03g44010	Os_chr03	24724847	[T/G]	REGULATORY
OsMed19_1	LOC_Os03g44010	Os_chr03	24724833	[G/A]	REGULATORY
OsMed19_1	LOC_Os03g44010	Os_chr03	24724824	[C/T]	REGULATORY
OsMed19_1	LOC_Os03g44010	Os_chr03	24724823	[G/A]	REGULATORY
OsMed19_1	LOC_Os03g44010	Os_chr03	24724794	[C/T]	REGULATORY
OsMed19_1	LOC_Os03g44010	Os_chr03	24724755	[C/A]	REGULATORY
OsMed19_1	LOC_Os03g44010	Os_chr03	24724729	[G/A]	REGULATORY
OsMed19_1	LOC_Os03g44010	Os_chr03	24724521	[T/C]	REGULATORY
OsMed19_1	LOC_Os03g44010	Os_chr03	24724464	[C/T]	REGULATORY
OsMed19_1	LOC_Os03g44010	Os_chr03	24724434	[G/C]	REGULATORY
OsMed19_1	LOC_Os03g44010	Os_chr03	24724432	[T/C]	REGULATORY
OsMed19_2	LOC_Os12g41962	Os_chr12	25992042	[G/A]	INTRON
OsMed19_2	LOC_Os12g41962	Os_chr12	25992009	[C/T]	INTRON
OsMed19_2	LOC_Os12g41962	Os_chr12	25991894	[T/A]	INTRON

**Mediator genes**	**MSU locus ID**	**Chromosomes**	**Physical Position (bp)**	**SNPs**	**Structural Annotation**
OsMed19_2	LOC_Os12g41962	Os_chr12	25991822	[C/T]	INTRON
OsMed19_2	LOC_Os12g41962	Os_chr12	25991636	[A/G]	INTRON
OsMed19_2	LOC_Os12g41962	Os_chr12	25991444	[A/T]	INTRON
OsMed19_2	LOC_Os12g41962	Os_chr12	25990123	[T/A]	INTRON
OsMed19_2	LOC_Os12g41962	Os_chr12	25990091	[T/C]	INTRON
OsMed19_2	LOC_Os12g41962	Os_chr12	25989859	[G/T]	INTRON
OsMed19_2	LOC_Os12g41962	Os_chr12	25990714	[G/A]	NON-SYNONYMOUS-CODING
OsMed19_2	LOC_Os12g41962	Os_chr12	25990684	[C/T]	NON-SYNONYMOUS-CODING
OsMed19_2	LOC_Os12g41962	Os_chr12	25990395	[C/G]	NON-SYNONYMOUS-CODING
OsMed19_2	LOC_Os12g41962	Os_chr12	25988496	[T/C]	REGULATORY
OsMed19_2	LOC_Os12g41962	Os_chr12	25989587	[G/A]	REGULATORY
OsMed19_2	LOC_Os12g41962	Os_chr12	25989384	[T/C]	REGULATORY
OsMed19_2	LOC_Os12g41962	Os_chr12	25989259	[C/T]	REGULATORY
OsMed19_2	LOC_Os12g41962	Os_chr12	25989093	[A/G]	REGULATORY
OsMed19_2	LOC_Os12g41962	Os_chr12	25988996	[A/G]	REGULATORY
OsMed19_2	LOC_Os12g41962	Os_chr12	25988881	[C/T]	REGULATORY
OsMed19_2	LOC_Os12g41962	Os_chr12	25989818	[A/G]	REGULATORY
OsMed19_2	LOC_Os12g41962	Os_chr12	25992426	[A/T]	REGULATORY
OsMed19_2	LOC_Os12g41962	Os_chr12	25992303	[A/G]	REGULATORY
OsMed19_2	LOC_Os12g41962	Os_chr12	25992302	[T/G]	REGULATORY
OsMed19_2	LOC_Os12g41962	Os_chr12	25992236	[A/G]	REGULATORY
OsMed19_2	LOC_Os12g41962	Os_chr12	25991262	[T/C]	REGULATORY
OsMed19_2	LOC_Os12g41962	Os_chr12	25988051	[G/T]	REGULATORY
OsMed19_2	LOC_Os12g41962	Os_chr12	25988037	[T/A]	REGULATORY
OsMed19_2	LOC_Os12g41962	Os_chr12	25988023	[C/T]	REGULATORY
OsMed19_2	LOC_Os12g41962	Os_chr12	25988005	[C/T]	REGULATORY
OsMed19_2	LOC_Os12g41962	Os_chr12	25987966	[G/A]	REGULATORY
OsMed19_2	LOC_Os12g41962	Os_chr12	25987956	[C/T]	REGULATORY
OsMed19_2	LOC_Os12g41962	Os_chr12	25987950	[A/G]	REGULATORY
OsMed19_2	LOC_Os12g41962	Os_chr12	25987822	[G/A]	REGULATORY
OsMed19_2	LOC_Os12g41962	Os_chr12	25987817	[G/C]	REGULATORY
OsMed19_2	LOC_Os12g41962	Os_chr12	25987786	[C/T]	REGULATORY
OsMed19_2	LOC_Os12g41962	Os_chr12	25987742	[T/C]	REGULATORY
OsMed19_2	LOC_Os12g41962	Os_chr12	25987719	[G/A]	REGULATORY
OsMed19_2	LOC_Os12g41962	Os_chr12	25987684	[A/G]	REGULATORY
OsMed19_2	LOC_Os12g41962	Os_chr12	25987641	[T/C]	REGULATORY
OsMed19_2	LOC_Os12g41962	Os_chr12	25987473	[A/T]	REGULATORY
OsMed19_2	LOC_Os12g41962	Os_chr12	25987322	[G/C]	REGULATORY
OsMed19_2	LOC_Os12g41962	Os_chr12	25987303	[A/C]	REGULATORY
OsMed19_2	LOC_Os12g41962	Os_chr12	25987253	[G/T]	REGULATORY
OsMed19_2	LOC_Os12g41962	Os_chr12	25987216	[T/A]	REGULATORY
OsMed19_2	LOC_Os12g41962	Os_chr12	25990470	[T/C]	SYNONYMOUS-CODING
OsMed20_1	LOC_Os09g27140	Os_chr09	16504333	[G/A]	INTRON
OsMed20_1	LOC_Os09g27140	Os_chr09	16504320	[C/T]	INTRON
OsMed20_1	LOC_Os09g27140	Os_chr09	16504315	[C/A]	INTRON
OsMed20_1	LOC_Os09g27140	Os_chr09	16504300	[C/T]	INTRON
OsMed20_1	LOC_Os09g27140	Os_chr09	16504297	[C/T]	INTRON

Mediator genes	MSU locus ID	Chromosomes	Physical Position (bp)	SNPs	Structural Annotation
OsMed20_1	LOC_Os09g27140	Os_chr09	16504291	[A/T]	INTRON
OsMed20_1	LOC_Os09g27140	Os_chr09	16504289	[G/C]	INTRON
OsMed20_1	LOC_Os09g27140	Os_chr09	16504256	[A/C]	INTRON
OsMed20_1	LOC_Os09g27140	Os_chr09	16504233	[C/A]	INTRON
OsMed20_1	LOC_Os09g27140	Os_chr09	16504193	[G/A]	INTRON
OsMed20_1	LOC_Os09g27140	Os_chr09	16504189	[G/T]	INTRON
OsMed20_1	LOC_Os09g27140	Os_chr09	16504167	[G/A]	INTRON
OsMed20_1	LOC_Os09g27140	Os_chr09	16504165	[A/T]	INTRON
OsMed20_1	LOC_Os09g27140	Os_chr09	16504157	[G/A]	INTRON
OsMed20_1	LOC_Os09g27140	Os_chr09	16504146	[G/A]	INTRON
OsMed20_1	LOC_Os09g27140	Os_chr09	16504145	[G/A]	INTRON
OsMed20_1	LOC_Os09g27140	Os_chr09	16504140	[C/T]	INTRON
OsMed20_1	LOC_Os09g27140	Os_chr09	16504126	[T/A]	INTRON
OsMed20_1	LOC_Os09g27140	Os_chr09	16504120	[G/A]	INTRON
OsMed20_1	LOC_Os09g27140	Os_chr09	16504075	[T/C]	INTRON
OsMed20_1	LOC_Os09g27140	Os_chr09	16504070	[T/C]	INTRON
OsMed20_1	LOC_Os09g27140	Os_chr09	16504065	[G/A]	INTRON
OsMed20_1	LOC_Os09g27140	Os_chr09	16504041	[T/A]	INTRON
OsMed20_1	LOC_Os09g27140	Os_chr09	16504030	[C/A]	INTRON
OsMed20_1	LOC_Os09g27140	Os_chr09	16504002	[C/A]	INTRON
OsMed20_1	LOC_Os09g27140	Os_chr09	16503985	[G/T]	INTRON
OsMed20_1	LOC_Os09g27140	Os_chr09	16503970	[C/T]	INTRON
OsMed20_1	LOC_Os09g27140	Os_chr09	16503876	[C/G]	INTRON
OsMed20_1	LOC_Os09g27140	Os_chr09	16503830	[G/A]	INTRON
OsMed20_1	LOC_Os09g27140	Os_chr09	16503823	[G/A]	INTRON
OsMed20_1	LOC_Os09g27140	Os_chr09	16503742	[G/A]	INTRON
OsMed20_1	LOC_Os09g27140	Os_chr09	16503709	[G/A]	INTRON
OsMed20_1	LOC_Os09g27140	Os_chr09	16503702	[G/A]	INTRON
OsMed20_1	LOC_Os09g27140	Os_chr09	16503676	[C/T]	INTRON
OsMed20_1	LOC_Os09g27140	Os_chr09	16503662	[G/A]	INTRON
OsMed20_1	LOC_Os09g27140	Os_chr09	16503657	[G/A]	INTRON
OsMed20_1	LOC_Os09g27140	Os_chr09	16503640	[A/T]	INTRON
OsMed20_1	LOC_Os09g27140	Os_chr09	16503637	[G/A]	INTRON
OsMed20_1	LOC_Os09g27140	Os_chr09	16503614	[G/A]	INTRON
OsMed20_1	LOC_Os09g27140	Os_chr09	16503613	[G/T]	INTRON
OsMed20_1	LOC_Os09g27140	Os_chr09	16503589	[C/A]	INTRON
OsMed20_1	LOC_Os09g27140	Os_chr09	16503579	[G/A]	INTRON
OsMed20_1	LOC_Os09g27140	Os_chr09	16503571	[G/A]	INTRON
OsMed20_1	LOC_Os09g27140	Os_chr09	16503548	[G/A]	INTRON
OsMed20_1	LOC_Os09g27140	Os_chr09	16503538	[G/A]	INTRON
OsMed20_1	LOC_Os09g27140	Os_chr09	16503531	[G/A]	INTRON
OsMed20_1	LOC_Os09g27140	Os_chr09	16503513	[G/T]	INTRON
OsMed20_1	LOC_Os09g27140	Os_chr09	16503470	[G/A]	INTRON
OsMed20_1	LOC_Os09g27140	Os_chr09	16507690	[C/T]	INTRON
OsMed20_1	LOC_Os09g27140	Os_chr09	16507671	[A/G]	INTRON
OsMed20_1	LOC_Os09g27140	Os_chr09	16507638	[C/T]	INTRON
OsMed20_1	LOC_Os09g27140	Os_chr09	16507093	[A/G]	INTRON

Mediator genes	MSU locus ID	Chromosomes	Physical Position (bp)	SNPs	Structural Annotation
OsMed20_1	LOC_Os09g27140	Os_chr09	16506985	[C/A]	INTRON
OsMed20_1	LOC_Os09g27140	Os_chr09	16506946	[C/T]	INTRON
OsMed20_1	LOC_Os09g27140	Os_chr09	16506784	[G/A]	INTRON
OsMed20_1	LOC_Os09g27140	Os_chr09	16506777	[A/C]	INTRON
OsMed20_1	LOC_Os09g27140	Os_chr09	16506673	[C/G]	INTRON
OsMed20_1	LOC_Os09g27140	Os_chr09	16506660	[T/A]	INTRON
OsMed20_1	LOC_Os09g27140	Os_chr09	16506645	[T/C]	INTRON
OsMed20_1	LOC_Os09g27140	Os_chr09	16506579	[C/A]	INTRON
OsMed20_1	LOC_Os09g27140	Os_chr09	16506430	[A/C]	INTRON
OsMed20_1	LOC_Os09g27140	Os_chr09	16506416	[G/C]	INTRON
OsMed20_1	LOC_Os09g27140	Os_chr09	16506411	[G/T]	INTRON
OsMed20_1	LOC_Os09g27140	Os_chr09	16506276	[C/A]	INTRON
OsMed20_1	LOC_Os09g27140	Os_chr09	16506203	[G/A]	INTRON
OsMed20_1	LOC_Os09g27140	Os_chr09	16505999	[T/C]	INTRON
OsMed20_1	LOC_Os09g27140	Os_chr09	16505876	[C/A]	INTRON
OsMed20_1	LOC_Os09g27140	Os_chr09	16505469	[C/G]	INTRON
OsMed20_1	LOC_Os09g27140	Os_chr09	16505249	[C/A]	INTRON
OsMed20_1	LOC_Os09g27140	Os_chr09	16505136	[C/A]	INTRON
OsMed20_1	LOC_Os09g27140	Os_chr09	16505131	[G/A]	INTRON
OsMed20_1	LOC_Os09g27140	Os_chr09	16505056	[C/A]	INTRON
OsMed20_1	LOC_Os09g27140	Os_chr09	16504986	[G/C]	INTRON
OsMed20_1	LOC_Os09g27140	Os_chr09	16504461	[G/C]	INTRON
OsMed20_1	LOC_Os09g27140	Os_chr09	16504403	[T/C]	INTRON
OsMed20_1	LOC_Os09g27140	Os_chr09	16504389	[G/A]	INTRON
OsMed20_1	LOC_Os09g27140	Os_chr09	16504380	[C/T]	INTRON
OsMed20_1	LOC_Os09g27140	Os_chr09	16504354	[A/T]	INTRON
OsMed20_1	LOC_Os09g27140	Os_chr09	16504353	[G/A]	INTRON
OsMed20_1	LOC_Os09g27140	Os_chr09	16508724	[T/C]	INTRON
OsMed20_1	LOC_Os09g27140	Os_chr09	16507611	[G/T]	NON-SYNONYMOUS-CODING
OsMed20_1	LOC_Os09g27140	Os_chr09	16507513	[A/G]	NON-SYNONYMOUS-CODING
OsMed20_1	LOC_Os09g27140	Os_chr09	16507357	[C/G]	NON-SYNONYMOUS-CODING
OsMed20_1	LOC_Os09g27140	Os_chr09	16504879	[G/C]	NON-SYNONYMOUS-CODING
OsMed20_1	LOC_Os09g27140	Os_chr09	16504876	[A/G]	NON-SYNONYMOUS-CODING
OsMed20_1	LOC_Os09g27150	Os_chr09	16508594	[A/G]	NON-SYNONYMOUS-CODING
OsMed20_1	LOC_Os09g27140	Os_chr09	16507762	[C/A]	REGULATORY
OsMed20_1	LOC_Os09g27140	Os_chr09	16503462	[C/T]	REGULATORY
OsMed20_1	LOC_Os09g27140	Os_chr09	16503403	[C/T]	REGULATORY
OsMed20_1	LOC_Os09g27140	Os_chr09	16503393	[T/C]	REGULATORY
OsMed20_1	LOC_Os09g27140	Os_chr09	16503369	[G/A]	REGULATORY
OsMed20_1	LOC_Os09g27140	Os_chr09	16503353	[C/T]	REGULATORY
OsMed20_1	LOC_Os09g27140	Os_chr09	16503346	[G/A]	REGULATORY
OsMed20_1	LOC_Os09g27140	Os_chr09	16503330	[C/T]	REGULATORY
OsMed20_1	LOC_Os09g27140	Os_chr09	16503301	[G/A]	REGULATORY
OsMed20_1	LOC_Os09g27140	Os_chr09	16503263	[C/T]	REGULATORY
OsMed20_1	LOC_Os09g27140	Os_chr09	16503245	[C/T]	REGULATORY
OsMed20_1	LOC_Os09g27140	Os_chr09	16503138	[G/C]	REGULATORY
OsMed20_1	LOC_Os09g27140	Os_chr09	16503137	[G/A]	REGULATORY

Mediator genes	MSU locus ID	Chromosomes	Physical Position (bp)	SNPs	Structural Annotation
OsMed20_1	LOC_Os09g27140	Os_chr09	16503135	[T/C]	REGULATORY
OsMed20_1	LOC_Os09g27140	Os_chr09	16503131	[G/A]	REGULATORY
OsMed20_1	LOC_Os09g27140	Os_chr09	16503038	[C/T]	REGULATORY
OsMed20_1	LOC_Os09g27140	Os_chr09	16503018	[G/A]	REGULATORY
OsMed20_1	LOC_Os09g27140	Os_chr09	16503010	[C/T]	REGULATORY
OsMed20_1	LOC_Os09g27140	Os_chr09	16503008	[G/A]	REGULATORY
OsMed20_1	LOC_Os09g27140	Os_chr09	16503002	[G/A]	REGULATORY
OsMed20_1	LOC_Os09g27140	Os_chr09	16502988	[C/T]	REGULATORY
OsMed20_1	LOC_Os09g27140	Os_chr09	16502940	[G/A]	REGULATORY
OsMed20_1	LOC_Os09g27140	Os_chr09	16502937	[G/A]	REGULATORY
OsMed20_1	LOC_Os09g27140	Os_chr09	16508426	[G/T]	REGULATORY
OsMed20_1	LOC_Os09g27140	Os_chr09	16508294	[G/A]	REGULATORY
OsMed20_1	LOC_Os09g27140	Os_chr09	16508286	[G/A]	REGULATORY
OsMed20_1	LOC_Os09g27140	Os_chr09	16508275	[G/A]	REGULATORY
OsMed20_1	LOC_Os09g27140	Os_chr09	16508228	[C/T]	REGULATORY
OsMed20_1	LOC_Os09g27140	Os_chr09	16508140	[G/A]	REGULATORY
OsMed20_1	LOC_Os09g27140	Os_chr09	16508131	[A/G]	REGULATORY
OsMed20_1	LOC_Os09g27140	Os_chr09	16508123	[G/A]	REGULATORY
OsMed20_1	LOC_Os09g27140	Os_chr09	16508113	[G/A]	REGULATORY
OsMed20_1	LOC_Os09g27140	Os_chr09	16508112	[C/A]	REGULATORY
OsMed20_1	LOC_Os09g27140	Os_chr09	16508103	[C/T]	REGULATORY
OsMed20_1	LOC_Os09g27140	Os_chr09	16508102	[G/A]	REGULATORY
OsMed20_1	LOC_Os09g27140	Os_chr09	16508096	[C/T]	REGULATORY
OsMed20_1	LOC_Os09g27140	Os_chr09	16508094	[G/A]	REGULATORY
OsMed20_1	LOC_Os09g27140	Os_chr09	16508066	[C/A]	REGULATORY
OsMed20_1	LOC_Os09g27140	Os_chr09	16508042	[C/T]	REGULATORY
OsMed20_1	LOC_Os09g27140	Os_chr09	16508040	[G/T]	REGULATORY
OsMed20_1	LOC_Os09g27140	Os_chr09	16508037	[A/G]	REGULATORY
OsMed20_1	LOC_Os09g27140	Os_chr09	16508033	[G/A]	REGULATORY
OsMed20_1	LOC_Os09g27140	Os_chr09	16508001	[T/C]	REGULATORY
OsMed20_1	LOC_Os09g27140	Os_chr09	16507997	[C/A]	REGULATORY
OsMed20_1	LOC_Os09g27140	Os_chr09	16507960	[G/A]	REGULATORY
OsMed20_1	LOC_Os09g27140	Os_chr09	16507934	[T/C]	REGULATORY
OsMed20_1	LOC_Os09g27140	Os_chr09	16507475	[G/A]	SYNONYMOUS-CODING
OsMed20_1	LOC_Os09g27140	Os_chr09	16505641	[G/A]	SYNONYMOUS-CODING
OsMed20_1	LOC_Os09g27140	Os_chr09	16504776	[A/G]	SYNONYMOUS-CODING
OsMed21_1	LOC_Os08g04150	Os_chr08	2014025	[G/A]	INTRON
OsMed21_1	LOC_Os08g04150	Os_chr08	2013980	[G/C]	INTRON
OsMed21_1	LOC_Os08g04150	Os_chr08	2013954	[A/G]	INTRON
OsMed21_1	LOC_Os08g04150	Os_chr08	2013932	[G/A]	INTRON
OsMed21_1	LOC_Os08g04150	Os_chr08	2013683	[T/G]	INTRON
OsMed21_1	LOC_Os08g04150	Os_chr08	2013663	[C/T]	INTRON
OsMed21_1	LOC_Os08g04150	Os_chr08	2013662	[C/A]	INTRON
OsMed21_1	LOC_Os08g04150	Os_chr08	2013655	[C/T]	INTRON
OsMed21_1	LOC_Os08g04150	Os_chr08	2013644	[C/G]	INTRON
OsMed21_1	LOC_Os08g04150	Os_chr08	2013634	[A/G]	INTRON
OsMed21_1	LOC_Os08g04150	Os_chr08	2013625	[G/C]	INTRON

**Mediator genes**	**MSU locus ID**	**Chromosomes**	**Physical Position (bp)**	**SNPs**	**Structural Annotation**
OsMed21_1	LOC_Os08g04150	Os_chr08	2013605	[G/A]	INTRON
OsMed21_1	LOC_Os08g04150	Os_chr08	2013560	[A/T]	INTRON
OsMed21_1	LOC_Os08g04150	Os_chr08	2013550	[A/C]	INTRON
OsMed21_1	LOC_Os08g04150	Os_chr08	2013542	[T/A]	INTRON
OsMed21_1	LOC_Os08g04150	Os_chr08	2013459	[C/T]	INTRON
OsMed21_1	LOC_Os08g04150	Os_chr08	2013438	[G/T]	INTRON
OsMed21_1	LOC_Os08g04150	Os_chr08	2013418	[C/T]	INTRON
OsMed21_1	LOC_Os08g04150	Os_chr08	2013412	[C/T]	INTRON
OsMed21_1	LOC_Os08g04150	Os_chr08	2013395	[T/C]	INTRON
OsMed21_1	LOC_Os08g04150	Os_chr08	2013391	[T/C]	INTRON
OsMed21_1	LOC_Os08g04150	Os_chr08	2013367	[G/C]	INTRON
OsMed21_1	LOC_Os08g04150	Os_chr08	2013331	[G/A]	INTRON
OsMed21_1	LOC_Os08g04150	Os_chr08	2013321	[A/C]	INTRON
OsMed21_1	LOC_Os08g04150	Os_chr08	2013319	[A/G]	INTRON
OsMed21_1	LOC_Os08g04150	Os_chr08	2013309	[A/G]	INTRON
OsMed21_1	LOC_Os08g04150	Os_chr08	2013307	[A/G]	INTRON
OsMed21_1	LOC_Os08g04150	Os_chr08	2013277	[A/T]	INTRON
OsMed21_1	LOC_Os08g04150	Os_chr08	2013276	[A/G]	INTRON
OsMed21_1	LOC_Os08g04150	Os_chr08	2013257	[A/G]	INTRON
OsMed21_1	LOC_Os08g04150	Os_chr08	2013084	[T/A]	INTRON
OsMed21_1	LOC_Os08g04150	Os_chr08	2013076	[T/C]	INTRON
OsMed21_1	LOC_Os08g04150	Os_chr08	2013055	[G/A]	INTRON
OsMed21_1	LOC_Os08g04150	Os_chr08	2013049	[C/A]	INTRON
OsMed21_1	LOC_Os08g04150	Os_chr08	2012890	[G/A]	INTRON
OsMed21_1	LOC_Os08g04150	Os_chr08	2012862	[T/G]	INTRON
OsMed21_1	LOC_Os08g04150	Os_chr08	2012816	[T/C]	INTRON
OsMed21_1	LOC_Os08g04150	Os_chr08	2012746	[A/G]	INTRON
OsMed21_1	LOC_Os08g04150	Os_chr08	2012742	[G/A]	INTRON
OsMed21_1	LOC_Os08g04150	Os_chr08	2012734	[A/G]	INTRON
OsMed21_1	LOC_Os08g04150	Os_chr08	2012707	[T/C]	INTRON
OsMed21_1	LOC_Os08g04150	Os_chr08	2012672	[G/C]	INTRON
OsMed21_1	LOC_Os08g04150	Os_chr08	2012627	[G/T]	INTRON
OsMed21_1	LOC_Os08g04150	Os_chr08	2012606	[G/A]	INTRON
OsMed21_1	LOC_Os08g04150	Os_chr08	2012579	[C/T]	INTRON
OsMed21_1	LOC_Os08g04150	Os_chr08	2012537	[A/G]	INTRON
OsMed21_1	LOC_Os08g04150	Os_chr08	2012520	[G/C]	INTRON
OsMed21_1	LOC_Os08g04150	Os_chr08	2012507	[C/T]	INTRON
OsMed21_1	LOC_Os08g04150	Os_chr08	2012500	[T/G]	INTRON
OsMed21_1	LOC_Os08g04150	Os_chr08	2012498	[T/C]	INTRON
OsMed21_1	LOC_Os08g04150	Os_chr08	2014496	[G/A]	NON-SYNONYMOUS-CODING
OsMed21_1	LOC_Os08g04150	Os_chr08	2014446	[C/T]	NON-SYNONYMOUS-CODING
OsMed21_1	LOC_Os08g04150	Os_chr08	2014394	[C/G]	NON-SYNONYMOUS-CODING
OsMed21_1	LOC_Os08g04150	Os_chr08	2013169	[A/T]	NON-SYNONYMOUS-CODING
OsMed21_1	LOC_Os08g04150	Os_chr08	2012458	[A/G]	REGULATORY
OsMed21_1	LOC_Os08g04150	Os_chr08	2012406	[T/C]	REGULATORY
OsMed21_1	LOC_Os08g04150	Os_chr08	2012405	[C/T]	REGULATORY
OsMed21_1	LOC_Os08g04150	Os_chr08	2012362	[T/A]	REGULATORY

**Mediator genes**	**MSU locus ID**	**Chromosomes**	**Physical Position (bp)**	**SNPs**	**Structural Annotation**
OsMed21_1	LOC_Os08g04150	Os_chr08	2012357	[C/T]	REGULATORY
OsMed21_1	LOC_Os08g04150	Os_chr08	2012347	[C/A]	REGULATORY
OsMed21_1	LOC_Os08g04150	Os_chr08	2012316	[A/G]	REGULATORY
OsMed21_1	LOC_Os08g04150	Os_chr08	2012275	[T/C]	REGULATORY
OsMed21_1	LOC_Os08g04150	Os_chr08	2012271	[A/T]	REGULATORY
OsMed21_1	LOC_Os08g04150	Os_chr08	2012267	[T/A]	REGULATORY
OsMed21_1	LOC_Os08g04150	Os_chr08	2012247	[G/A]	REGULATORY
OsMed21_1	LOC_Os08g04150	Os_chr08	2012245	[T/G]	REGULATORY
OsMed21_1	LOC_Os08g04150	Os_chr08	2014722	[G/C]	REGULATORY
OsMed21_1	LOC_Os08g04150	Os_chr08	2014715	[A/G]	REGULATORY
OsMed21_1	LOC_Os08g04150	Os_chr08	2014706	[T/A]	REGULATORY
OsMed21_1	LOC_Os08g04150	Os_chr08	2014670	[T/C]	REGULATORY
OsMed21_1	LOC_Os08g04150	Os_chr08	2014645	[C/T]	REGULATORY
OsMed21_1	LOC_Os08g04150	Os_chr08	2014641	[C/A]	REGULATORY
OsMed21_1	LOC_Os08g04150	Os_chr08	2014617	[C/T]	REGULATORY
OsMed21_1	LOC_Os08g04150	Os_chr08	2014574	[G/T]	REGULATORY
OsMed21_1	LOC_Os08g04150	Os_chr08	2014435	[T/C]	SYNONYMOUS-CODING
OsMed21_1	LOC_Os08g04150	Os_chr08	2014417	[T/C]	SYNONYMOUS-CODING
OsMed21_1	LOC_Os08g04150	Os_chr08	2013140	[T/A]	SYNONYMOUS-CODING
OsMed21_1	LOC_Os08g04150	Os_chr08	2012996	[C/A]	SYNONYMOUS-CODING
OsMed22_1	LOC_Os01g04110	Os_chr01	1790276	[G/A]	INTRON
OsMed22_1	LOC_Os01g04110	Os_chr01	1790115	[A/G]	INTRON
OsMed22_1	LOC_Os01g04110	Os_chr01	1790027	[G/T]	INTRON
OsMed22_1	LOC_Os01g04110	Os_chr01	1790023	[C/T]	INTRON
OsMed22_1	LOC_Os01g04110	Os_chr01	1789309	[C/G]	INTRON
OsMed22_1	LOC_Os01g04110	Os_chr01	1789272	[C/T]	INTRON
OsMed22_1	LOC_Os01g04110	Os_chr01	1789071	[C/T]	INTRON
OsMed22_1	LOC_Os01g04110	Os_chr01	1789037	[C/T]	INTRON
OsMed22_1	LOC_Os01g04110	Os_chr01	1788204	[C/T]	INTRON
OsMed22_1	LOC_Os01g04110	Os_chr01	1788199	[G/A]	INTRON
OsMed22_1	LOC_Os01g04110	Os_chr01	1788149	[C/A]	INTRON
OsMed22_1	LOC_Os01g04110	Os_chr01	1788142	[C/T]	INTRON
OsMed22_1	LOC_Os01g04110	Os_chr01	1788132	[T/A]	INTRON
OsMed22_1	LOC_Os01g04110	Os_chr01	1788094	[C/T]	INTRON
OsMed22_1	LOC_Os01g04110	Os_chr01	1788080	[C/T]	INTRON
OsMed22_1	LOC_Os01g04110	Os_chr01	1788054	[G/A]	INTRON
OsMed22_1	LOC_Os01g04110	Os_chr01	1788041	[G/A]	INTRON
OsMed22_1	LOC_Os01g04110	Os_chr01	1788019	[A/G]	INTRON
OsMed22_1	LOC_Os01g04110	Os_chr01	1788005	[A/C]	INTRON
OsMed22_1	LOC_Os01g04110	Os_chr01	1787851	[C/T]	INTRON
OsMed22_1	LOC_Os01g04110	Os_chr01	1787814	[G/A]	INTRON
OsMed22_1	LOC_Os01g04110	Os_chr01	1787776	[C/T]	INTRON
OsMed22_1	LOC_Os01g04110	Os_chr01	1787761	[C/T]	INTRON
OsMed22_1	LOC_Os01g04110	Os_chr01	1787760	[C/T]	INTRON
OsMed22_1	LOC_Os01g04110	Os_chr01	1787704	[C/A]	INTRON
OsMed22_1	LOC_Os01g04110	Os_chr01	1787627	[G/A]	INTRON
OsMed22_1	LOC_Os01g04110	Os_chr01	1787599	[G/A]	INTRON

**Mediator genes**	**MSU locus ID**	**Chromosomes**	**Physical Position (bp)**	**SNPs**	**Structural Annotation**
OsMed22_1	LOC_Os01g04110	Os_chr01	1787527	[C/T]	INTRON
OsMed22_1	LOC_Os01g04110	Os_chr01	1787516	[T/C]	INTRON
OsMed22_1	LOC_Os01g04110	Os_chr01	1787481	[C/A]	INTRON
OsMed22_1	LOC_Os01g04110	Os_chr01	1787466	[G/A]	INTRON
OsMed22_1	LOC_Os01g04110	Os_chr01	1787376	[A/T]	INTRON
OsMed22_1	LOC_Os01g04110	Os_chr01	1790166	[G/A]	NON-SYNONYMOUS-CODING
OsMed22_1	LOC_Os01g04110	Os_chr01	1788862	[G/A]	NON-SYNONYMOUS-CODING
OsMed22_1	LOC_Os01g04110	Os_chr01	1788740	[C/G]	NON-SYNONYMOUS-CODING
OsMed22_1	LOC_Os01g04110	Os_chr01	1790456	[C/T]	REGULATORY
OsMed22_1	LOC_Os01g04110	Os_chr01	1788630	[T/C]	REGULATORY
OsMed22_1	LOC_Os01g04110	Os_chr01	1787106	[G/C]	REGULATORY
OsMed22_1	LOC_Os01g04110	Os_chr01	1787072	[A/T]	REGULATORY
OsMed22_1	LOC_Os01g04110	Os_chr01	1791335	[G/A]	REGULATORY
OsMed22_1	LOC_Os01g04110	Os_chr01	1791289	[T/C]	REGULATORY
OsMed22_1	LOC_Os01g04110	Os_chr01	1791277	[C/T]	REGULATORY
OsMed22_1	LOC_Os01g04110	Os_chr01	1791274	[A/G]	REGULATORY
OsMed22_1	LOC_Os01g04110	Os_chr01	1791251	[A/G]	REGULATORY
OsMed22_1	LOC_Os01g04110	Os_chr01	1791220	[C/A]	REGULATORY
OsMed22_1	LOC_Os01g04110	Os_chr01	1791191	[T/C]	REGULATORY
OsMed22_1	LOC_Os01g04110	Os_chr01	1791180	[C/T]	REGULATORY
OsMed22_1	LOC_Os01g04110	Os_chr01	1791179	[G/A]	REGULATORY
OsMed22_1	LOC_Os01g04110	Os_chr01	1791155	[T/A]	REGULATORY
OsMed22_1	LOC_Os01g04110	Os_chr01	1791149	[A/C]	REGULATORY
OsMed22_1	LOC_Os01g04110	Os_chr01	1791148	[C/T]	REGULATORY
OsMed22_1	LOC_Os01g04110	Os_chr01	1791083	[C/T]	REGULATORY
OsMed22_1	LOC_Os01g04110	Os_chr01	1790860	[G/A]	REGULATORY
OsMed22_1	LOC_Os01g04110	Os_chr01	1790830	[C/T]	REGULATORY
OsMed22_1	LOC_Os01g04110	Os_chr01	1790820	[G/A]	REGULATORY
OsMed22_1	LOC_Os01g04110	Os_chr01	1790795	[G/A]	REGULATORY
OsMed22_1	LOC_Os01g04110	Os_chr01	1790785	[C/T]	REGULATORY
OsMed22_1	LOC_Os01g04110	Os_chr01	1790769	[C/T]	REGULATORY
OsMed22_1	LOC_Os01g04110	Os_chr01	1790757	[A/G]	REGULATORY
OsMed22_1	LOC_Os01g04110	Os_chr01	1790736	[C/T]	REGULATORY
OsMed22_1	LOC_Os01g04110	Os_chr01	1790725	[G/A]	REGULATORY
OsMed22_1	LOC_Os01g04110	Os_chr01	1790684	[A/C]	REGULATORY
OsMed22_1	LOC_Os01g04110	Os_chr01	1790675	[T/C]	REGULATORY
OsMed22_1	LOC_Os01g04110	Os_chr01	1790669	[G/T]	REGULATORY
OsMed22_2	LOC_Os08g37480	Os_chr08	23741679	[T/C]	INTRON
OsMed22_2	LOC_Os08g37480	Os_chr08	23741417	[A/G]	INTRON
OsMed22_2	LOC_Os08g37480	Os_chr08	23741214	[T/G]	INTRON
OsMed22_2	LOC_Os08g37480	Os_chr08	23741153	[C/A]	INTRON
OsMed22_2	LOC_Os08g37480	Os_chr08	23741046	[G/A]	INTRON
OsMed22_2	LOC_Os08g37480	Os_chr08	23741042	[C/A]	INTRON
OsMed22_2	LOC_Os08g37480	Os_chr08	23740960	[C/T]	INTRON
OsMed22_2	LOC_Os08g37480	Os_chr08	23740916	[A/T]	INTRON
OsMed22_2	LOC_Os08g37480	Os_chr08	23740836	[C/T]	INTRON
OsMed22_2	LOC_Os08g37480	Os_chr08	23740812	[G/A]	INTRON

Mediator genes	MSU locus ID	Chromosomes	Physical Position (bp)	SNPs	Structural Annotation
OsMed22_2	LOC_Os08g37480	Os_chr08	23740694	[T/C]	INTRON
OsMed22_2	LOC_Os08g37480	Os_chr08	23740657	[A/G]	INTRON
OsMed22_2	LOC_Os08g37480	Os_chr08	23740571	[C/G]	INTRON
OsMed22_2	LOC_Os08g37480	Os_chr08	23740452	[C/T]	INTRON
OsMed22_2	LOC_Os08g37480	Os_chr08	23740429	[G/A]	INTRON
OsMed22_2	LOC_Os08g37480	Os_chr08	23740423	[C/T]	INTRON
OsMed22_2	LOC_Os08g37480	Os_chr08	23740347	[C/T]	INTRON
OsMed22_2	LOC_Os08g37480	Os_chr08	23740184	[T/C]	INTRON
OsMed22_2	LOC_Os08g37480	Os_chr08	23739495	[A/T]	INTRON
OsMed22_2	LOC_Os08g37480	Os_chr08	23739438	[G/A]	INTRON
OsMed22_2	LOC_Os08g37480	Os_chr08	23743703	[A/G]	INTRON
OsMed22_2	LOC_Os08g37480	Os_chr08	23743507	[A/T]	INTRON
OsMed22_2	LOC_Os08g37480	Os_chr08	23743460	[A/G]	INTRON
OsMed22_2	LOC_Os08g37480	Os_chr08	23743134	[T/C]	INTRON
OsMed22_2	LOC_Os08g37480	Os_chr08	23742981	[C/T]	INTRON
OsMed22_2	LOC_Os08g37480	Os_chr08	23742848	[A/G]	INTRON
OsMed22_2	LOC_Os08g37480	Os_chr08	23742772	[A/T]	INTRON
OsMed22_2	LOC_Os08g37480	Os_chr08	23742446	[T/A]	INTRON
OsMed22_2	LOC_Os08g37480	Os_chr08	23742416	[C/A]	INTRON
OsMed22_2	LOC_Os08g37480	Os_chr08	23742352	[G/A]	INTRON
OsMed22_2	LOC_Os08g37480	Os_chr08	23742293	[A/T]	INTRON
OsMed22_2	LOC_Os08g37480	Os_chr08	23741877	[G/A]	NON-SYNONYMOUS-CODING
OsMed22_2	LOC_Os08g37480	Os_chr08	23738984	[T/A]	REGULATORY
OsMed22_2	LOC_Os08g37480	Os_chr08	23738787	[G/A]	REGULATORY
OsMed22_2	LOC_Os08g37480	Os_chr08	23738735	[C/T]	REGULATORY
OsMed22_2	LOC_Os08g37480	Os_chr08	23745275	[C/T]	REGULATORY
OsMed22_2	LOC_Os08g37480	Os_chr08	23745252	[G/A]	REGULATORY
OsMed22_2	LOC_Os08g37480	Os_chr08	23745208	[G/A]	REGULATORY
OsMed22_2	LOC_Os08g37480	Os_chr08	23745138	[A/G]	REGULATORY
OsMed22_2	LOC_Os08g37480	Os_chr08	23745092	[A/G]	REGULATORY
OsMed22_2	LOC_Os08g37480	Os_chr08	23745088	[C/T]	REGULATORY
OsMed22_2	LOC_Os08g37480	Os_chr08	23745061	[A/G]	REGULATORY
OsMed22_2	LOC_Os08g37480	Os_chr08	23744807	[A/G]	REGULATORY
OsMed22_2	LOC_Os08g37480	Os_chr08	23744768	[A/G]	REGULATORY
OsMed22_2	LOC_Os08g37480	Os_chr08	23744730	[A/G]	REGULATORY
OsMed22_2	LOC_Os08g37480	Os_chr08	23744530	[C/T]	REGULATORY
OsMed22_2	LOC_Os08g37480	Os_chr08	23744529	[G/A]	REGULATORY
OsMed22_2	LOC_Os08g37480	Os_chr08	23744481	[A/G]	REGULATORY
OsMed22_2	LOC_Os08g37480	Os_chr08	23744471	[G/A]	REGULATORY
OsMed22_2	LOC_Os08g37480	Os_chr08	23744380	[G/A]	REGULATORY
OsMed22_2	LOC_Os08g37480	Os_chr08	23744354	[A/G]	REGULATORY
OsMed22_2	LOC_Os08g37480	Os_chr08	23744336	[C/T]	REGULATORY
OsMed22_2	LOC_Os08g37480	Os_chr08	23742002	[C/G]	SYNONYMOUS-CODING
OsMed23_1	LOC_Os02g49992	Os_chr02	30547092	[G/C]	INTRON
OsMed23_1	LOC_Os02g49992	Os_chr02	30545344	[T/G]	INTRON
OsMed23_1	LOC_Os02g49992	Os_chr02	30545004	[G/A]	INTRON
OsMed23_1	LOC_Os02g49992	Os_chr02	30544980	[T/G]	INTRON

**Mediator genes**	**MSU locus ID**	**Chromosomes**	**Physical Position (bp)**	**SNPs**	**Structural Annotation**
OsMed23_1	LOC_Os02g49992	Os_chr02	30544313	[T/G]	INTRON
OsMed23_1	LOC_Os02g49992	Os_chr02	30543992	[T/A]	INTRON
OsMed23_1	LOC_Os02g49992	Os_chr02	30543341	[C/T]	INTRON
OsMed23_1	LOC_Os02g49992	Os_chr02	30543062	[A/G]	INTRON
OsMed23_1	LOC_Os02g49992	Os_chr02	30542513	[G/T]	INTRON
OsMed23_1	LOC_Os02g49992	Os_chr02	30542484	[T/A]	INTRON
OsMed23_1	LOC_Os02g49992	Os_chr02	30542184	[C/A]	INTRON
OsMed23_1	LOC_Os02g49992	Os_chr02	30541851	[T/C]	INTRON
OsMed23_1	LOC_Os02g49992	Os_chr02	30541831	[G/A]	INTRON
OsMed23_1	LOC_Os02g49992	Os_chr02	30541659	[G/A]	INTRON
OsMed23_1	LOC_Os02g49992	Os_chr02	30541561	[G/C]	INTRON
OsMed23_1	LOC_Os02g49992	Os_chr02	30541448	[G/T]	INTRON
OsMed23_1	LOC_Os02g49992	Os_chr02	30540512	[C/G]	INTRON
OsMed23_1	LOC_Os02g49992	Os_chr02	30539950	[C/T]	INTRON
OsMed23_1	LOC_Os02g49992	Os_chr02	30539608	[A/G]	INTRON
OsMed23_1	LOC_Os02g49992	Os_chr02	30539542	[G/T]	INTRON
OsMed23_1	LOC_Os02g49992	Os_chr02	30539511	[A/G]	INTRON
OsMed23_1	LOC_Os02g49992	Os_chr02	30539356	[A/G]	INTRON
OsMed23_1	LOC_Os02g49992	Os_chr02	30539267	[C/T]	INTRON
OsMed23_1	LOC_Os02g49992	Os_chr02	30539151	[T/C]	INTRON
OsMed23_1	LOC_Os02g49992	Os_chr02	30537661	[A/G]	INTRON
OsMed23_1	LOC_Os02g49992	Os_chr02	30537558	[A/G]	INTRON
OsMed23_1	LOC_Os02g49992	Os_chr02	30546483	[G/C]	NON-SYNONYMOUS-CODING
OsMed23_1	LOC_Os02g49992	Os_chr02	30546104	[A/T]	NON-SYNONYMOUS-CODING
OsMed23_1	LOC_Os02g49992	Os_chr02	30545858	[A/G]	NON-SYNONYMOUS-CODING
OsMed23_1	LOC_Os02g49992	Os_chr02	30544711	[A/C]	NON-SYNONYMOUS-CODING
OsMed23_1	LOC_Os02g49992	Os_chr02	30544011	[T/C]	NON-SYNONYMOUS-CODING
OsMed23_1	LOC_Os02g49992	Os_chr02	30541135	[A/T]	NON-SYNONYMOUS-CODING
OsMed23_1	LOC_Os02g49992	Os_chr02	30540197	[C/T]	NON-SYNONYMOUS-CODING
OsMed23_1	LOC_Os02g49992	Os_chr02	30536625	[G/T]	REGULATORY
OsMed23_1	LOC_Os02g57590	Os_chr02	30548430	[G/A]	REGULATORY
OsMed23_1	LOC_Os02g57590	Os_chr02	30548412	[G/T]	REGULATORY
OsMed23_1	LOC_Os02g57590	Os_chr02	30548396	[G/A]	REGULATORY
OsMed23_1	LOC_Os02g57590	Os_chr02	30548370	[C/T]	REGULATORY
OsMed23_1	LOC_Os02g57590	Os_chr02	30548352	[G/A]	REGULATORY
OsMed23_1	LOC_Os02g57590	Os_chr02	30548348	[A/G]	REGULATORY
OsMed23_1	LOC_Os02g57590	Os_chr02	30548340	[T/C]	REGULATORY
OsMed23_1	LOC_Os02g57590	Os_chr02	30548312	[T/C]	REGULATORY
OsMed23_1	LOC_Os02g57590	Os_chr02	30548299	[T/C]	REGULATORY
OsMed23_1	LOC_Os02g57590	Os_chr02	30548297	[C/T]	REGULATORY
OsMed23_1	LOC_Os02g57590	Os_chr02	30548184	[A/G]	REGULATORY
OsMed23_1	LOC_Os02g57590	Os_chr02	30548121	[T/C]	REGULATORY
OsMed23_1	LOC_Os02g57590	Os_chr02	30548087	[G/A]	REGULATORY
OsMed23_1	LOC_Os02g57590	Os_chr02	30547774	[T/G]	REGULATORY
OsMed23_1	LOC_Os02g57590	Os_chr02	30547740	[C/T]	REGULATORY
OsMed23_1	LOC_Os02g49992	Os_chr02	30547585	[C/T]	SYNONYMOUS-CODING
OsMed23_1	LOC_Os02g49992	Os_chr02	30546466	[A/G]	SYNONYMOUS-CODING

**Mediator genes**	**MSU locus ID**	**Chromosomes**	**Physical Position (bp)**	**SNPs**	**Structural Annotation**
OsMed23_1	LOC_Os02g49992	Os_chr02	30546265	[C/T]	SYNONYMOUS-CODING
OsMed23_1	LOC_Os02g49992	Os_chr02	30545190	[G/A]	SYNONYMOUS-CODING
OsMed23_1	LOC_Os02g49992	Os_chr02	30538983	[A/G]	SYNONYMOUS-CODING
OsMed23_1	LOC_Os02g49992	Os_chr02	30538896	[G/A]	SYNONYMOUS-CODING
OsMed23_1	LOC_Os02g49992	Os_chr02	30538322	[A/G]	SYNONYMOUS-CODING
OsMed25_1	LOC_Os09g13610	Os_chr09	7923217	[G/A]	INTRON
OsMed25_1	LOC_Os09g13610	Os_chr09	7923215	[T/A]	INTRON
OsMed25_1	LOC_Os09g13610	Os_chr09	7923178	[T/G]	INTRON
OsMed25_1	LOC_Os09g13610	Os_chr09	7922944	[G/A]	INTRON
OsMed25_1	LOC_Os09g13610	Os_chr09	7922812	[T/C]	INTRON
OsMed25_1	LOC_Os09g13610	Os_chr09	7922477	[G/A]	INTRON
OsMed25_1	LOC_Os09g13610	Os_chr09	7922406	[T/A]	INTRON
OsMed25_1	LOC_Os09g13610	Os_chr09	7922360	[C/T]	INTRON
OsMed25_1	LOC_Os09g13610	Os_chr09	7922272	[C/T]	INTRON
OsMed25_1	LOC_Os09g13610	Os_chr09	7922171	[G/C]	INTRON
OsMed25_1	LOC_Os09g13610	Os_chr09	7921990	[C/T]	INTRON
OsMed25_1	LOC_Os09g13610	Os_chr09	7921277	[C/T]	INTRON
OsMed25_1	LOC_Os09g13610	Os_chr09	7921248	[G/A]	INTRON
OsMed25_1	LOC_Os09g13610	Os_chr09	7921131	[A/G]	INTRON
OsMed25_1	LOC_Os09g13610	Os_chr09	7921113	[A/G]	INTRON
OsMed25_1	LOC_Os09g13610	Os_chr09	7921065	[C/T]	INTRON
OsMed25_1	LOC_Os09g13610	Os_chr09	7920926	[A/T]	INTRON
OsMed25_1	LOC_Os09g13610	Os_chr09	7920881	[C/A]	INTRON
OsMed25_1	LOC_Os09g13610	Os_chr09	7920878	[C/G]	INTRON
OsMed25_1	LOC_Os09g13610	Os_chr09	7920859	[C/T]	INTRON
OsMed25_1	LOC_Os09g13610	Os_chr09	7920726	[G/T]	INTRON
OsMed25_1	LOC_Os09g13610	Os_chr09	7920719	[T/G]	INTRON
OsMed25_1	LOC_Os09g13610	Os_chr09	7920702	[A/G]	INTRON
OsMed25_1	LOC_Os09g13610	Os_chr09	7920661	[G/A]	INTRON
OsMed25_1	LOC_Os09g13610	Os_chr09	7920585	[T/C]	INTRON
OsMed25_1	LOC_Os09g13610	Os_chr09	7920569	[C/T]	INTRON
OsMed25_1	LOC_Os09g13610	Os_chr09	7920474	[A/C]	INTRON
OsMed25_1	LOC_Os09g13610	Os_chr09	7920459	[T/A]	INTRON
OsMed25_1	LOC_Os09g13610	Os_chr09	7919388	[C/T]	INTRON
OsMed25_1	LOC_Os09g13610	Os_chr09	7919131	[A/G]	INTRON
OsMed25_1	LOC_Os09g13610	Os_chr09	7919087	[G/A]	INTRON
OsMed25_1	LOC_Os09g13610	Os_chr09	7918909	[A/G]	INTRON
OsMed25_1	LOC_Os09g13610	Os_chr09	7918673	[C/G]	INTRON
OsMed25_1	LOC_Os09g13610	Os_chr09	7918634	[G/T]	INTRON
OsMed25_1	LOC_Os09g13610	Os_chr09	7918628	[C/A]	INTRON
OsMed25_1	LOC_Os09g13610	Os_chr09	7918406	[T/A]	INTRON
OsMed25_1	LOC_Os09g13610	Os_chr09	7918378	[G/A]	INTRON
OsMed25_1	LOC_Os09g13610	Os_chr09	7918371	[T/A]	INTRON
OsMed25_1	LOC_Os09g13610	Os_chr09	7918367	[A/C]	INTRON
OsMed25_1	LOC_Os09g13610	Os_chr09	7918349	[G/T]	INTRON
OsMed25_1	LOC_Os09g13610	Os_chr09	7918335	[C/T]	INTRON
OsMed25_1	LOC_Os09g13610	Os_chr09	7918290	[A/G]	INTRON

Mediator genes	MSU locus ID	Chromosomes	Physical Position (bp)	SNPs	Structural Annotation
OsMed25_1	LOC_Os09g13610	Os_chr09	7917814	[T/C]	INTRON
OsMed25_1	LOC_Os09g13610	Os_chr09	7917791	[A/T]	INTRON
OsMed25_1	LOC_Os09g13610	Os_chr09	7917663	[A/G]	INTRON
OsMed25_1	LOC_Os09g13610	Os_chr09	7917524	[T/G]	INTRON
OsMed25_1	LOC_Os09g13610	Os_chr09	7917498	[C/T]	INTRON
OsMed25_1	LOC_Os09g13610	Os_chr09	7917223	[T/G]	INTRON
OsMed25_1	LOC_Os09g13610	Os_chr09	7917152	[C/T]	INTRON
OsMed25_1	LOC_Os09g13610	Os_chr09	7917067	[A/G]	INTRON
OsMed25_1	LOC_Os09g13610	Os_chr09	7916656	[G/A]	INTRON
OsMed25_1	LOC_Os09g13610	Os_chr09	7916610	[A/G]	INTRON
OsMed25_1	LOC_Os09g13610	Os_chr09	7916531	[A/G]	INTRON
OsMed25_1	LOC_Os09g13610	Os_chr09	7916316	[G/A]	INTRON
OsMed25_1	LOC_Os09g13610	Os_chr09	7916302	[T/C]	INTRON
OsMed25_1	LOC_Os09g13610	Os_chr09	7916298	[C/T]	INTRON
OsMed25_1	LOC_Os09g13610	Os_chr09	7916249	[A/C]	INTRON
OsMed25_1	LOC_Os09g13610	Os_chr09	7916231	[G/A]	INTRON
OsMed25_1	LOC_Os09g13610	Os_chr09	7916220	[C/T]	INTRON
OsMed25_1	LOC_Os09g13610	Os_chr09	7916115	[G/A]	INTRON
OsMed25_1	LOC_Os09g13610	Os_chr09	7916106	[C/T]	INTRON
OsMed25_1	LOC_Os09g13610	Os_chr09	7916060	[T/A]	INTRON
OsMed25_1	LOC_Os09g13610	Os_chr09	7916010	[C/T]	INTRON
OsMed25_1	LOC_Os09g13610	Os_chr09	7915828	[A/G]	INTRON
OsMed25_1	LOC_Os09g13610	Os_chr09	7915797	[A/G]	INTRON
OsMed25_1	LOC_Os09g13610	Os_chr09	7915774	[C/G]	INTRON
OsMed25_1	LOC_Os09g13610	Os_chr09	7915742	[A/C]	INTRON
OsMed25_1	LOC_Os09g13610	Os_chr09	7915700	[T/C]	INTRON
OsMed25_1	LOC_Os09g13610	Os_chr09	7915541	[G/T]	INTRON
OsMed25_1	LOC_Os09g13610	Os_chr09	7915391	[G/T]	INTRON
OsMed25_1	LOC_Os09g13610	Os_chr09	7915260	[G/A]	INTRON
OsMed25_1	LOC_Os09g13610	Os_chr09	7915064	[C/T]	INTRON
OsMed25_1	LOC_Os09g13610	Os_chr09	7915020	[T/A]	INTRON
OsMed25_1	LOC_Os09g13610	Os_chr09	7915019	[C/A]	INTRON
OsMed25_1	LOC_Os09g13610	Os_chr09	7914904	[A/C]	INTRON
OsMed25_1	LOC_Os09g13610	Os_chr09	7914831	[C/T]	INTRON
OsMed25_1	LOC_Os09g13610	Os_chr09	7914638	[T/G]	INTRON
OsMed25_1	LOC_Os09g13610	Os_chr09	7914545	[C/T]	INTRON
OsMed25_1	LOC_Os09g13610	Os_chr09	7914308	[T/C]	INTRON
OsMed25_1	LOC_Os09g13610	Os_chr09	7914279	[A/T]	INTRON
OsMed25_1	LOC_Os09g13610	Os_chr09	7914266	[C/T]	INTRON
OsMed25_1	LOC_Os09g13610	Os_chr09	7914212	[G/A]	INTRON
OsMed25_1	LOC_Os09g13610	Os_chr09	7914211	[C/T]	INTRON
OsMed25_1	LOC_Os09g13610	Os_chr09	7914158	[G/T]	INTRON
OsMed25_1	LOC_Os09g13610	Os_chr09	7914101	[G/T]	INTRON
OsMed25_1	LOC_Os09g13610	Os_chr09	7913989	[T/A]	INTRON
OsMed25_1	LOC_Os09g13610	Os_chr09	7913857	[G/C]	INTRON
OsMed25_1	LOC_Os09g13610	Os_chr09	7913806	[G/A]	INTRON
OsMed25_1	LOC_Os09g13610	Os_chr09	7913770	[G/T]	INTRON

**Mediator genes**	**MSU locus ID**	**Chromosomes**	**Physical Position (bp)**	**SNPs**	**Structural Annotation**
OsMed25_1	LOC_Os09g13610	Os_chr09	7913630	[C/T]	INTRON
OsMed25_1	LOC_Os09g13610	Os_chr09	7913435	[T/C]	INTRON
OsMed25_1	LOC_Os09g13610	Os_chr09	7913418	[C/G]	INTRON
OsMed25_1	LOC_Os09g13610	Os_chr09	7923934	[G/A]	NON-SYNONYMOUS-CODING
OsMed25_1	LOC_Os09g13610	Os_chr09	7923676	[G/T]	NON-SYNONYMOUS-CODING
OsMed25_1	LOC_Os09g13610	Os_chr09	7923546	[T/C]	NON-SYNONYMOUS-CODING
OsMed25_1	LOC_Os09g13610	Os_chr09	7921525	[C/T]	NON-SYNONYMOUS-CODING
OsMed25_1	LOC_Os09g13610	Os_chr09	7921458	[G/A]	NON-SYNONYMOUS-CODING
OsMed25_1	LOC_Os09g13610	Os_chr09	7921455	[C/G]	NON-SYNONYMOUS-CODING
OsMed25_1	LOC_Os09g13610	Os_chr09	7920107	[A/G]	NON-SYNONYMOUS-CODING
OsMed25_1	LOC_Os09g13610	Os_chr09	7919512	[C/T]	NON-SYNONYMOUS-CODING
OsMed25_1	LOC_Os09g13610	Os_chr09	7916913	[G/A]	NON-SYNONYMOUS-CODING
OsMed25_1	LOC_Os09g13610	Os_chr09	7913212	[G/A]	REGULATORY
OsMed25_1	LOC_Os09g13610	Os_chr09	7913177	[A/G]	REGULATORY
OsMed25_1	LOC_Os09g13610	Os_chr09	7913150	[G/A]	REGULATORY
OsMed25_1	LOC_Os09g13610	Os_chr09	7913124	[G/A]	REGULATORY
OsMed25_1	LOC_Os09g13610	Os_chr09	7913123	[T/A]	REGULATORY
OsMed25_1	LOC_Os09g13610	Os_chr09	7924374	[T/C]	REGULATORY
OsMed25_1	LOC_Os09g13610	Os_chr09	7924368	[C/A]	REGULATORY
OsMed25_1	LOC_Os09g13610	Os_chr09	7924358	[T/A]	REGULATORY
OsMed25_1	LOC_Os09g13610	Os_chr09	7924033	[A/G]	REGULATORY
OsMed25_1	LOC_Os09g13610	Os_chr09	7922655	[C/A]	REGULATORY
OsMed25_1	LOC_Os09g13610	Os_chr09	7921796	[A/G]	REGULATORY
OsMed25_1	LOC_Os09g13610	Os_chr09	7921699	[C/T]	REGULATORY
OsMed25_1	LOC_Os09g13610	Os_chr09	7921654	[T/C]	REGULATORY
OsMed25_1	LOC_Os09g13610	Os_chr09	7913063	[T/C]	REGULATORY
OsMed25_1	LOC_Os09g13610	Os_chr09	7913013	[G/A]	REGULATORY
OsMed25_1	LOC_Os09g13610	Os_chr09	7913008	[C/T]	REGULATORY
OsMed25_1	LOC_Os09g13610	Os_chr09	7912991	[C/T]	REGULATORY
OsMed25_1	LOC_Os09g13610	Os_chr09	7912984	[C/T]	REGULATORY
OsMed25_1	LOC_Os09g13610	Os_chr09	7912981	[C/T]	REGULATORY
OsMed25_1	LOC_Os09g13610	Os_chr09	7912929	[A/C]	REGULATORY
OsMed25_1	LOC_Os09g13610	Os_chr09	7912902	[G/T]	REGULATORY
OsMed25_1	LOC_Os09g13610	Os_chr09	7912874	[C/T]	REGULATORY
OsMed25_1	LOC_Os09g13610	Os_chr09	7912856	[T/C]	REGULATORY
OsMed25_1	LOC_Os09g13610	Os_chr09	7912851	[C/T]	REGULATORY
OsMed25_1	LOC_Os09g13610	Os_chr09	7912841	[C/T]	REGULATORY
OsMed25_1	LOC_Os09g13610	Os_chr09	7912791	[C/T]	REGULATORY
OsMed25_1	LOC_Os09g13610	Os_chr09	7912788	[G/A]	REGULATORY
OsMed25_1	LOC_Os09g13610	Os_chr09	7912769	[C/T]	REGULATORY
OsMed25_1	LOC_Os09g13610	Os_chr09	7912761	[C/T]	REGULATORY
OsMed25_1	LOC_Os09g13610	Os_chr09	7912760	[G/A]	REGULATORY
OsMed25_1	LOC_Os09g13610	Os_chr09	7912757	[C/T]	REGULATORY
OsMed25_1	LOC_Os09g13610	Os_chr09	7912748	[C/T]	REGULATORY
OsMed25_1	LOC_Os09g13610	Os_chr09	7912739	[A/C]	REGULATORY
OsMed25_1	LOC_Os09g13610	Os_chr09	7912738	[T/G]	REGULATORY
OsMed25_1	LOC_Os09g13610	Os_chr09	7912733	[A/C]	REGULATORY

Mediator genes	MSU locus ID	Chromosomes	Physical Position (bp)	SNPs	Structural Annotation
OsMed25_1	LOC_Os09g13610	Os_chr09	7912725	[A/T]	REGULATORY
OsMed25_1	LOC_Os09g13610	Os_chr09	7912722	[C/T]	REGULATORY
OsMed25_1	LOC_Os09g13610	Os_chr09	7912721	[C/T]	REGULATORY
OsMed25_1	LOC_Os09g13610	Os_chr09	7912713	[T/C]	REGULATORY
OsMed25_1	LOC_Os09g13610	Os_chr09	7912700	[C/T]	REGULATORY
OsMed25_1	LOC_Os09g13610	Os_chr09	7912682	[G/A]	REGULATORY
OsMed25_1	LOC_Os09g13610	Os_chr09	7912669	[C/T]	REGULATORY
OsMed25_1	LOC_Os09g13610	Os_chr09	7912657	[C/T]	REGULATORY
OsMed25_1	LOC_Os09g13610	Os_chr09	7912655	[A/G]	REGULATORY
OsMed25_1	LOC_Os09g13610	Os_chr09	7912643	[G/A]	REGULATORY
OsMed25_1	LOC_Os09g13610	Os_chr09	7912631	[C/T]	REGULATORY
OsMed25_1	LOC_Os09g13610	Os_chr09	7912619	[A/G]	REGULATORY
OsMed25_1	LOC_Os09g13610	Os_chr09	7912615	[G/A]	REGULATORY
OsMed25_1	LOC_Os09g13610	Os_chr09	7912603	[T/C]	REGULATORY
OsMed25_1	LOC_Os09g13610	Os_chr09	7912602	[G/A]	REGULATORY
OsMed25_1	LOC_Os09g13610	Os_chr09	7912591	[T/A]	REGULATORY
OsMed25_1	LOC_Os09g13610	Os_chr09	7912576	[C/T]	REGULATORY
OsMed25_1	LOC_Os09g13610	Os_chr09	7912572	[G/A]	REGULATORY
OsMed25_1	LOC_Os09g13610	Os_chr09	7912570	[C/T]	REGULATORY
OsMed25_1	LOC_Os09g13610	Os_chr09	7912552	[G/A]	REGULATORY
OsMed25_1	LOC_Os09g13610	Os_chr09	7912547	[C/T]	REGULATORY
OsMed25_1	LOC_Os09g13610	Os_chr09	7912524	[C/T]	REGULATORY
OsMed25_1	LOC_Os09g13610	Os_chr09	7912492	[A/G]	REGULATORY
OsMed25_1	LOC_Os09g13610	Os_chr09	7912463	[A/C]	REGULATORY
OsMed25_1	LOC_Os09g13610	Os_chr09	7912411	[G/A]	REGULATORY
OsMed25_1	LOC_Os09g13610	Os_chr09	7912393	[C/G]	REGULATORY
OsMed25_1	LOC_Os09g13610	Os_chr09	7912386	[A/G]	REGULATORY
OsMed25_1	LOC_Os09g13610	Os_chr09	7912361	[G/A]	REGULATORY
OsMed25_1	LOC_Os09g13610	Os_chr09	7912336	[C/T]	REGULATORY
OsMed25_1	LOC_Os09g13610	Os_chr09	7912331	[T/C]	REGULATORY
OsMed25_1	LOC_Os09g13610	Os_chr09	7912320	[T/C]	REGULATORY
OsMed25_1	LOC_Os09g13610	Os_chr09	7912239	[A/G]	REGULATORY
OsMed25_1	LOC_Os09g13610	Os_chr09	7912225	[G/A]	REGULATORY
OsMed25_1	LOC_Os09g13610	Os_chr09	7912194	[G/A]	REGULATORY
OsMed25_1	LOC_Os09g13610	Os_chr09	7912157	[A/C]	REGULATORY
OsMed25_1	LOC_Os09g13610	Os_chr09	7912120	[A/T]	REGULATORY
OsMed25_1	LOC_Os09g13610	Os_chr09	7912118	[C/T]	REGULATORY
OsMed25_1	LOC_Os09g13610	Os_chr09	7912099	[T/A]	REGULATORY
OsMed25_1	LOC_Os09g13610	Os_chr09	7923892	[G/T]	SYNONYMOUS-CODING
OsMed25_1	LOC_Os09g13610	Os_chr09	7923751	[A/G]	SYNONYMOUS-CODING
OsMed25_1	LOC_Os09g13610	Os_chr09	7923745	[G/A]	SYNONYMOUS-CODING
OsMed25_1	LOC_Os09g13610	Os_chr09	7923577	[A/G]	SYNONYMOUS-CODING
OsMed25_1	LOC_Os09g13610	Os_chr09	7923448	[G/A]	SYNONYMOUS-CODING
OsMed25_1	LOC_Os09g13610	Os_chr09	7922740	[T/C]	SYNONYMOUS-CODING
OsMed25_1	LOC_Os09g13610	Os_chr09	7921633	[G/A]	SYNONYMOUS-CODING
OsMed25_1	LOC_Os09g13610	Os_chr09	7920298	[G/A]	SYNONYMOUS-CODING
OsMed25_1	LOC_Os09g13610	Os_chr09	7920295	[A/G]	SYNONYMOUS-CODING

**Mediator genes**	**MSU locus ID**	**Chromosomes**	**Physical Position (bp)**	**SNPs**	**Structural Annotation**
OsMed25_1	LOC_Os09g13610	Os_chr09	7920256	[A/G]	SYNONYMOUS-CODING
OsMed25_1	LOC_Os09g13610	Os_chr09	7920025	[G/A]	SYNONYMOUS-CODING
OsMed25_1	LOC_Os09g13610	Os_chr09	7919638	[G/A]	SYNONYMOUS-CODING
OsMed25_1	LOC_Os09g13610	Os_chr09	7919611	[G/A]	SYNONYMOUS-CODING
OsMed25_1	LOC_Os09g13610	Os_chr09	7918109	[A/T]	SYNONYMOUS-CODING
OsMed25_1	LOC_Os09g13610	Os_chr09	7917108	[T/G]	SYNONYMOUS-CODING
OsMed25_1	LOC_Os09g13610	Os_chr09	7915192	[A/G]	SYNONYMOUS-CODING
OsMed25_1	LOC_Os09g13610	Os_chr09	7914973	[C/G]	SYNONYMOUS-CODING
OsMed25_1	LOC_Os09g13610	Os_chr09	7913350	[C/T]	SYNONYMOUS-CODING
OsMed26_1	LOC_Os10g10260	Os_chr10	5662135	[C/T]	NON-SYNONYMOUS-CODING
OsMed26_1	LOC_Os10g10260	Os_chr10	5662125	[C/T]	NON-SYNONYMOUS-CODING
OsMed26_1	LOC_Os10g10260	Os_chr10	5662120	[T/C]	NON-SYNONYMOUS-CODING
OsMed26_1	LOC_Os10g10260	Os_chr10	5661892	[C/T]	NON-SYNONYMOUS-CODING
OsMed26_1	LOC_Os10g10260	Os_chr10	5661850	[T/C]	NON-SYNONYMOUS-CODING
OsMed26_1	LOC_Os10g10260	Os_chr10	5661768	[T/C]	NON-SYNONYMOUS-CODING
OsMed26_1	LOC_Os10g10270	Os_chr10	5663189	[C/G]	NON-SYNONYMOUS-CODING
OsMed26_1	LOC_Os10g10270	Os_chr10	5663079	[G/T]	NON-SYNONYMOUS-CODING
OsMed26_1	LOC_Os10g10270	Os_chr10	5662959	[G/T]	NON-SYNONYMOUS-CODING
OsMed26_1	LOC_Os10g10270	Os_chr10	5663078	[G/T]	SYNONYMOUS-CODING
OsMed26_1	LOC_Os10g10270	Os_chr10	5662553	[C/T]	REGULATORY
OsMed26_1	LOC_Os10g10270	Os_chr10	5662492	[G/C]	REGULATORY
OsMed26_1	LOC_Os10g10270	Os_chr10	5662396	[C/A]	REGULATORY
OsMed26_2	LOC_Os11g06650	Os_chr11	3231731	[G/A]	INTRON
OsMed26_2	LOC_Os11g06650	Os_chr11	3231694	[C/T]	INTRON
OsMed26_2	LOC_Os11g06650	Os_chr11	3231500	[A/G]	INTRON
OsMed26_2	LOC_Os11g06650	Os_chr11	3231131	[G/A]	INTRON
OsMed26_2	LOC_Os11g06650	Os_chr11	3230878	[G/T]	INTRON
OsMed26_2	LOC_Os11g06650	Os_chr11	3230876	[A/T]	INTRON
OsMed26_2	LOC_Os11g06650	Os_chr11	3229809	[T/C]	REGULATORY
OsMed26_2	LOC_Os11g06650	Os_chr11	3229638	[A/G]	REGULATORY
OsMed26_2	LOC_Os11g06650	Os_chr11	3233424	[A/C]	REGULATORY
OsMed26_2	LOC_Os11g06650	Os_chr11	3233369	[G/A]	REGULATORY
OsMed26_2	LOC_Os11g06650	Os_chr11	3233266	[A/G]	REGULATORY
OsMed26_2	LOC_Os11g06650	Os_chr11	3233254	[C/T]	REGULATORY
OsMed26_2	LOC_Os11g06650	Os_chr11	3233234	[C/T]	REGULATORY
OsMed26_2	LOC_Os11g06650	Os_chr11	3233170	[G/A]	REGULATORY
OsMed26_2	LOC_Os11g06650	Os_chr11	3233121	[C/T]	REGULATORY
OsMed26_2	LOC_Os11g06650	Os_chr11	3232968	[C/T]	REGULATORY
OsMed26_2	LOC_Os11g06650	Os_chr11	3232888	[T/C]	REGULATORY
OsMed26_2	LOC_Os11g06650	Os_chr11	3232879	[C/T]	REGULATORY
OsMed26_2	LOC_Os11g06650	Os_chr11	3232858	[T/C]	REGULATORY
OsMed26_2	LOC_Os11g06650	Os_chr11	3232850	[G/C]	REGULATORY
OsMed26_2	LOC_Os11g06650	Os_chr11	3232802	[T/C]	REGULATORY
OsMed26_2	LOC_Os11g06650	Os_chr11	3232773	[G/T]	REGULATORY
OsMed26_2	LOC_Os11g06650	Os_chr11	3232771	[T/C]	REGULATORY
OsMed26_2	LOC_Os11g06650	Os_chr11	3232763	[G/A]	REGULATORY
OsMed26_2	LOC_Os11g06650	Os_chr11	3232748	[A/C]	REGULATORY

**Mediator genes**	**MSU locus ID**	**Chromosomes**	**Physical Position (bp)**	**SNPs**	**Structural Annotation**
OsMed26_2	LOC_Os11g06650	Os_chr11	3232677	[T/G]	REGULATORY
OsMed26_2	LOC_Os11g06650	Os_chr11	3232596	[T/G]	REGULATORY
OsMed26_2	LOC_Os11g06650	Os_chr11	3232588	[T/G]	REGULATORY
OsMed26_2	LOC_Os11g06650	Os_chr11	3232474	[T/G]	REGULATORY
OsMed26_2	LOC_Os11g06650	Os_chr11	3231995	[G/A]	SYNONYMOUS-CODING
OsMed26_2	LOC_Os11g06650	Os_chr11	3231620	[G/A]	SYNONYMOUS-CODING
OsMed26_3	LOC_Os12g06850	Os_chr12	3325594	[A/T]	INTRON
OsMed26_3	LOC_Os12g06850	Os_chr12	3326944	[T/C]	INTRON
OsMed26_3	LOC_Os12g06850	Os_chr12	3326926	[G/A]	INTRON
OsMed26_3	LOC_Os12g06850	Os_chr12	3326481	[T/C]	INTRON
OsMed26_3	LOC_Os12g06850	Os_chr12	3325886	[G/A]	INTRON
OsMed26_3	LOC_Os12g06850	Os_chr12	3325247	[C/A]	NON-SYNONYMOUS-CODING
OsMed26_3	LOC_Os12g06850	Os_chr12	3326666	[C/T]	NON-SYNONYMOUS-CODING
OsMed26_3	LOC_Os12g06850	Os_chr12	3328046	[T/A]	REGULATORY
OsMed26_3	LOC_Os12g06850	Os_chr12	3328001	[A/G]	REGULATORY
OsMed26_3	LOC_Os12g06850	Os_chr12	3327691	[T/C]	REGULATORY
OsMed26_3	LOC_Os12g06850	Os_chr12	3324940	[T/C]	REGULATORY
OsMed26_3	LOC_Os12g06850	Os_chr12	3324751	[C/G]	REGULATORY
OsMed26_3	LOC_Os12g06850	Os_chr12	3329085	[C/T]	REGULATORY
OsMed26_3	LOC_Os12g06850	Os_chr12	3329033	[A/G]	REGULATORY
OsMed26_3	LOC_Os12g06850	Os_chr12	3328995	[G/A]	REGULATORY
OsMed26_3	LOC_Os12g06850	Os_chr12	3328989	[C/T]	REGULATORY
OsMed26_3	LOC_Os12g06850	Os_chr12	3328983	[T/G]	REGULATORY
OsMed26_3	LOC_Os12g06850	Os_chr12	3328962	[G/A]	REGULATORY
OsMed26_3	LOC_Os12g06850	Os_chr12	3328945	[G/A]	REGULATORY
OsMed26_3	LOC_Os12g06850	Os_chr12	3328919	[C/A]	REGULATORY
OsMed26_3	LOC_Os12g06850	Os_chr12	3328896	[G/A]	REGULATORY
OsMed26_3	LOC_Os12g06850	Os_chr12	3328865	[C/T]	REGULATORY
OsMed26_3	LOC_Os12g06850	Os_chr12	3328845	[T/G]	REGULATORY
OsMed26_3	LOC_Os12g06850	Os_chr12	3328773	[T/C]	REGULATORY
OsMed26_3	LOC_Os12g06850	Os_chr12	3328694	[G/A]	REGULATORY
OsMed26_3	LOC_Os12g06850	Os_chr12	3328653	[G/A]	REGULATORY
OsMed26_3	LOC_Os12g06850	Os_chr12	3328602	[T/G]	REGULATORY
OsMed26_3	LOC_Os12g06850	Os_chr12	3328560	[G/A]	REGULATORY
OsMed26_3	LOC_Os12g06850	Os_chr12	3328496	[C/T]	REGULATORY
OsMed26_3	LOC_Os12g06850	Os_chr12	3328469	[C/T]	REGULATORY
OsMed26_3	LOC_Os12g06850	Os_chr12	3328443	[T/C]	REGULATORY
OsMed26_3	LOC_Os12g06850	Os_chr12	3328422	[A/G]	REGULATORY
OsMed26_3	LOC_Os12g06850	Os_chr12	3328382	[G/A]	REGULATORY
OsMed26_3	LOC_Os12g06850	Os_chr12	3328322	[A/G]	REGULATORY
OsMed26_3	LOC_Os12g06850	Os_chr12	3325488	[G/A]	SYNONYMOUS-CODING
OsMed26_3	LOC_Os12g06850	Os_chr12	3325473	[T/C]	SYNONYMOUS-CODING
OsMed26_3	LOC_Os12g06850	Os_chr12	3325260	[C/T]	SYNONYMOUS-CODING
OsMed26_3	LOC_Os12g06850	Os_chr12	3326713	[T/C]	SYNONYMOUS-CODING
OsMed26_4	LOC_Os10g10260	Os_chr10	5662135	[C/T]	NON-SYNONYMOUS-CODING
OsMed26_4	LOC_Os10g10260	Os_chr10	5662125	[C/T]	NON-SYNONYMOUS-CODING
OsMed26_4	LOC_Os10g10260	Os_chr10	5662120	[T/C]	NON-SYNONYMOUS-CODING

**Mediator genes**	**MSU locus ID**	**Chromosomes**	**Physical Position (bp)**	**SNPs**	**Structural Annotation**
OsMed26_4	LOC_Os10g10260	Os_chr10	5661892	[C/T]	NON-SYNONYMOUS-CODING
OsMed26_4	LOC_Os10g10260	Os_chr10	5661850	[T/C]	NON-SYNONYMOUS-CODING
OsMed26_4	LOC_Os10g10260	Os_chr10	5661768	[T/C]	NON-SYNONYMOUS-CODING
OsMed26_4	LOC_Os10g10260	Os_chr10	5661508	[C/T]	NON-SYNONYMOUS-CODING
OsMed26_4	LOC_Os10g10260	Os_chr10	5661501	[C/T]	NON-SYNONYMOUS-CODING
OsMed26_4	LOC_Os10g10260	Os_chr10	5661466	[C/T]	NON-SYNONYMOUS-CODING
OsMed26_4	LOC_Os10g10270	Os_chr10	5663189	[C/G]	NON-SYNONYMOUS-CODING
OsMed26_4	LOC_Os10g10270	Os_chr10	5663079	[G/T]	NON-SYNONYMOUS-CODING
OsMed26_4	LOC_Os10g10270	Os_chr10	5662959	[G/T]	NON-SYNONYMOUS-CODING
OsMed26_4	LOC_Os10g10260	Os_chr10	5661611	[C/G]	SYNONYMOUS-CODING
OsMed26_4	LOC_Os10g10270	Os_chr10	5663078	[G/T]	SYNONYMOUS-CODING
OsMed26_4	LOC_Os10g10270	Os_chr10	5662553	[C/T]	REGULATORY
OsMed26_4	LOC_Os10g10270	Os_chr10	5662492	[G/C]	REGULATORY
OsMed26_4	LOC_Os10g10270	Os_chr10	5662396	[C/A]	REGULATORY
OsMed28_1	LOC_Os05g06290	Os_chr05	3215847	[T/A]	INTRON
OsMed28_1	LOC_Os05g06290	Os_chr05	3215833	[G/A]	INTRON
OsMed28_1	LOC_Os05g06290	Os_chr05	3215829	[C/A]	INTRON
OsMed28_1	LOC_Os05g06290	Os_chr05	3215396	[T/C]	INTRON
OsMed28_1	LOC_Os05g06290	Os_chr05	3215358	[C/T]	INTRON
OsMed28_1	LOC_Os05g06290	Os_chr05	3215299	[C/A]	INTRON
OsMed28_1	LOC_Os05g06290	Os_chr05	3215289	[C/T]	INTRON
OsMed28_1	LOC_Os05g06290	Os_chr05	3215272	[C/T]	INTRON
OsMed28_1	LOC_Os05g06290	Os_chr05	3215269	[C/A]	INTRON
OsMed28_1	LOC_Os05g06290	Os_chr05	3215236	[G/A]	INTRON
OsMed28_1	LOC_Os05g06290	Os_chr05	3215173	[T/C]	INTRON
OsMed28_1	LOC_Os05g06290	Os_chr05	3215138	[C/T]	INTRON
OsMed28_1	LOC_Os05g06290	Os_chr05	3215096	[C/A]	INTRON
OsMed28_1	LOC_Os05g06290	Os_chr05	3214953	[A/G]	INTRON
OsMed28_1	LOC_Os05g06290	Os_chr05	3214818	[T/A]	INTRON
OsMed28_1	LOC_Os05g06290	Os_chr05	3214614	[T/A]	NON-SYNONYMOUS-CODING
OsMed28_1	LOC_Os05g06290	Os_chr05	3216297	[G/T]	REGULATORY
OsMed28_1	LOC_Os05g06290	Os_chr05	3214365	[C/T]	REGULATORY
OsMed28_1	LOC_Os05g06290	Os_chr05	3214307	[T/C]	REGULATORY
OsMed28_1	LOC_Os05g06290	Os_chr05	3214233	[A/C]	REGULATORY
OsMed28_1	LOC_Os05g06290	Os_chr05	3217243	[A/C]	REGULATORY
OsMed28_1	LOC_Os05g06290	Os_chr05	3217186	[G/A]	REGULATORY
OsMed28_1	LOC_Os05g06290	Os_chr05	3217140	[C/T]	REGULATORY
OsMed28_1	LOC_Os05g06290	Os_chr05	3216952	[G/A]	REGULATORY
OsMed28_1	LOC_Os05g06290	Os_chr05	3216804	[A/T]	REGULATORY
OsMed28_1	LOC_Os05g06290	Os_chr05	3216756	[G/A]	REGULATORY
OsMed28_1	LOC_Os05g06290	Os_chr05	3216552	[A/G]	REGULATORY
OsMed28_1	LOC_Os05g06290	Os_chr05	3216546	[G/A]	REGULATORY
OsMed28_1	LOC_Os05g06290	Os_chr05	3216409	[C/T]	REGULATORY
OsMed28_1	LOC_Os05g06290	Os_chr05	3216397	[C/T]	REGULATORY
OsMed28_1	LOC_Os05g06290	Os_chr05	3216385	[T/C]	REGULATORY
OsMed3_1	LOC_Os01g15850	Os_chr01	8927985	[G/A]	INTRON
OsMed3_1	LOC_Os01g15850	Os_chr01	8926787	[C/T]	INTRON

**Mediator genes**	**MSU locus ID**	**Chromosomes**	**Physical Position (bp)**	**SNPs**	**Structural Annotation**
OsMed3_1	LOC_Os01g15850	Os_chr01	8926776	[A/T]	INTRON
OsMed3_1	LOC_Os01g15850	Os_chr01	8926761	[C/T]	INTRON
OsMed3_1	LOC_Os01g15850	Os_chr01	8926486	[G/A]	INTRON
OsMed3_1	LOC_Os01g15850	Os_chr01	8925941	[G/A]	INTRON
OsMed3_1	LOC_Os01g15850	Os_chr01	8925742	[G/A]	INTRON
OsMed3_1	LOC_Os01g15850	Os_chr01	8925741	[T/C]	INTRON
OsMed3_1	LOC_Os01g15850	Os_chr01	8925712	[A/T]	INTRON
OsMed3_1	LOC_Os01g15850	Os_chr01	8925706	[A/G]	INTRON
OsMed3_1	LOC_Os01g15850	Os_chr01	8925528	[A/G]	INTRON
OsMed3_1	LOC_Os01g15850	Os_chr01	8925390	[G/A]	INTRON
OsMed3_1	LOC_Os01g15850	Os_chr01	8925385	[G/C]	INTRON
OsMed3_1	LOC_Os01g15850	Os_chr01	8925310	[G/A]	INTRON
OsMed3_1	LOC_Os01g15850	Os_chr01	8925293	[T/C]	INTRON
OsMed3_1	LOC_Os01g15850	Os_chr01	8925104	[G/A]	INTRON
OsMed3_1	LOC_Os01g15850	Os_chr01	8925077	[G/A]	INTRON
OsMed3_1	LOC_Os01g15850	Os_chr01	8925076	[C/T]	INTRON
OsMed3_1	LOC_Os01g15850	Os_chr01	8924909	[C/T]	INTRON
OsMed3_1	LOC_Os01g15850	Os_chr01	8924819	[T/G]	INTRON
OsMed3_1	LOC_Os01g15850	Os_chr01	8924736	[G/A]	INTRON
OsMed3_1	LOC_Os01g15840	Os_chr01	8924005	[T/A]	NON-SYNONYMOUS-CODING
OsMed3_1	LOC_Os01g15840	Os_chr01	8923173	[C/T]	NON-SYNONYMOUS-CODING
OsMed3_1	LOC_Os01g15850	Os_chr01	8928601	[G/T]	REGULATORY
OsMed3_1	LOC_Os01g15840	Os_chr01	8923212	[C/T]	SYNONYMOUS-CODING
OsMed3_1	LOC_Os01g15850	Os_chr01	8928241	[G/A]	SYNONYMOUS-CODING
OsMed3_1	LOC_Os01g15850	Os_chr01	8927468	[A/G]	SYNONYMOUS-CODING
OsMed30_1	LOC_Os04g31910	Os_chr04	18944391	[A/T]	INTRON
OsMed30_1	LOC_Os04g31910	Os_chr04	18944034	[G/A]	INTRON
OsMed30_1	LOC_Os04g31910	Os_chr04	18943696	[A/T]	INTRON
OsMed30_1	LOC_Os04g31910	Os_chr04	18943649	[C/G]	INTRON
OsMed30_1	LOC_Os04g31910	Os_chr04	18943576	[G/A]	INTRON
OsMed30_1	LOC_Os04g31910	Os_chr04	18943535	[T/A]	INTRON
OsMed30_1	LOC_Os04g31910	Os_chr04	18943298	[G/T]	INTRON
OsMed30_1	LOC_Os04g31910	Os_chr04	18943019	[C/T]	INTRON
OsMed30_1	LOC_Os04g31910	Os_chr04	18942972	[C/T]	INTRON
OsMed30_1	LOC_Os04g31910	Os_chr04	18942728	[C/T]	INTRON
OsMed30_1	LOC_Os04g31910	Os_chr04	18942716	[C/A]	INTRON
OsMed30_1	LOC_Os04g31910	Os_chr04	18942572	[C/G]	INTRON
OsMed30_1	LOC_Os04g31910	Os_chr04	18942561	[T/C]	INTRON
OsMed30_1	LOC_Os04g31910	Os_chr04	18942414	[C/A]	INTRON
OsMed30_1	LOC_Os04g31910	Os_chr04	18944491	[T/C]	NON-SYNONYMOUS-CODING
OsMed30_1	LOC_Os04g31910	Os_chr04	18942234	[C/A]	REGULATORY
OsMed30_1	LOC_Os04g31910	Os_chr04	18942227	[T/A]	REGULATORY
OsMed30_1	LOC_Os04g31910	Os_chr04	18941985	[A/T]	REGULATORY
OsMed30_1	LOC_Os04g31910	Os_chr04	18941936	[A/G]	REGULATORY
OsMed30_1	LOC_Os04g31910	Os_chr04	18945913	[T/C]	REGULATORY
OsMed30_1	LOC_Os04g31910	Os_chr04	18945894	[G/A]	REGULATORY
OsMed30_1	LOC_Os04g31910	Os_chr04	18945822	[A/T]	REGULATORY

Mediator genes	MSU locus ID	Chromosomes	Physical Position (bp)	SNPs	Structural Annotation
OsMed30_1	LOC_Os04g31910	Os_chr04	18945772	[C/T]	REGULATORY
OsMed30_1	LOC_Os04g31910	Os_chr04	18945752	[G/A]	REGULATORY
OsMed30_1	LOC_Os04g31910	Os_chr04	18945711	[T/C]	REGULATORY
OsMed30_1	LOC_Os04g31910	Os_chr04	18945671	[A/C]	REGULATORY
OsMed30_1	LOC_Os04g31910	Os_chr04	18945637	[C/T]	REGULATORY
OsMed30_1	LOC_Os04g31910	Os_chr04	18945592	[G/A]	REGULATORY
OsMed30_1	LOC_Os04g31910	Os_chr04	18945585	[C/A]	REGULATORY
OsMed30_1	LOC_Os04g31910	Os_chr04	18945563	[G/A]	REGULATORY
OsMed30_1	LOC_Os04g31910	Os_chr04	18945528	[C/T]	REGULATORY
OsMed30_1	LOC_Os04g31910	Os_chr04	18945440	[G/A]	REGULATORY
OsMed30_1	LOC_Os04g31910	Os_chr04	18945436	[A/T]	REGULATORY
OsMed30_1	LOC_Os04g31910	Os_chr04	18945359	[C/T]	REGULATORY
OsMed30_1	LOC_Os04g31910	Os_chr04	18945350	[A/G]	REGULATORY
OsMed30_1	LOC_Os04g31910	Os_chr04	18945324	[G/A]	REGULATORY
OsMed30_1	LOC_Os04g31910	Os_chr04	18945264	[A/G]	REGULATORY
OsMed30_1	LOC_Os04g31910	Os_chr04	18945216	[T/C]	REGULATORY
OsMed30_1	LOC_Os04g31910	Os_chr04	18945213	[C/T]	REGULATORY
OsMed30_1	LOC_Os04g31910	Os_chr04	18945126	[C/T]	REGULATORY
OsMed30_1	LOC_Os04g31910	Os_chr04	18945089	[G/C]	REGULATORY
OsMed30_1	LOC_Os04g31910	Os_chr04	18945027	[C/G]	REGULATORY
OsMed31_1	LOC_Os07g07020	Os_chr07	3461341	[G/A]	INTRON
OsMed31_1	LOC_Os07g07020	Os_chr07	3460271	[G/T]	INTRON
OsMed31_1	LOC_Os07g07020	Os_chr07	3459601	[C/G]	INTRON
OsMed31_1	LOC_Os07g07020	Os_chr07	3459430	[G/T]	INTRON
OsMed31_1	LOC_Os07g07020	Os_chr07	3460793	[T/G]	INTRON
OsMed31_1	LOC_Os07g07020	Os_chr07	3460718	[C/T]	INTRON
OsMed31_1	LOC_Os07g07020	Os_chr07	3460697	[G/C]	INTRON
OsMed31_1	LOC_Os07g07020	Os_chr07	3460999	[A/G]	INTRON
OsMed31_1	LOC_Os07g07020	Os_chr07	3460968	[C/T]	INTRON
OsMed31_1	LOC_Os07g07020	Os_chr07	3459943	[C/T]	NON-SYNONYMOUS-CODING
OsMed31_1	LOC_Os07g07020	Os_chr07	3458909	[C/G]	REGULATORY
OsMed31_1	LOC_Os07g07020	Os_chr07	3461747	[C/T]	REGULATORY
OsMed31_1	LOC_Os07g07020	Os_chr07	3458743	[G/A]	REGULATORY
OsMed31_1	LOC_Os07g07020	Os_chr07	3458727	[T/C]	REGULATORY
OsMed31_1	LOC_Os07g07020	Os_chr07	3458716	[A/G]	REGULATORY
OsMed31_1	LOC_Os07g07020	Os_chr07	3458634	[G/A]	REGULATORY
OsMed31_1	LOC_Os07g07020	Os_chr07	3458618	[C/T]	REGULATORY
OsMed31_1	LOC_Os07g07020	Os_chr07	3458600	[T/A]	REGULATORY
OsMed31_1	LOC_Os07g07020	Os_chr07	3458564	[G/T]	REGULATORY
OsMed31_1	LOC_Os07g07020	Os_chr07	3458531	[G/A]	REGULATORY
OsMed31_1	LOC_Os07g07020	Os_chr07	3458470	[C/T]	REGULATORY
OsMed31_1	LOC_Os07g07020	Os_chr07	3458433	[C/G]	REGULATORY
OsMed31_1	LOC_Os07g07020	Os_chr07	3458399	[C/A]	REGULATORY
OsMed31_1	LOC_Os07g07020	Os_chr07	3458360	[G/T]	REGULATORY
OsMed31_1	LOC_Os07g07020	Os_chr07	3458348	[T/C]	REGULATORY
OsMed31_1	LOC_Os07g07020	Os_chr07	3458245	[G/T]	REGULATORY
OsMed31_1	LOC_Os07g07020	Os_chr07	3458237	[A/G]	REGULATORY

**Mediator genes**	**MSU locus ID**	**Chromosomes**	**Physical Position (bp)**	**SNPs**	**Structural Annotation**
OsMed31_1	LOC_Os07g07020	Os_chr07	3458215	[A/G]	REGULATORY
OsMed31_1	LOC_Os07g07020	Os_chr07	3458154	[G/A]	REGULATORY
OsMed31_1	LOC_Os07g07020	Os_chr07	3458135	[A/G]	REGULATORY
OsMed31_1	LOC_Os07g07020	Os_chr07	3458078	[G/A]	REGULATORY
OsMed31_1	LOC_Os07g07020	Os_chr07	3458075	[G/A]	REGULATORY
OsMed31_1	LOC_Os07g07020	Os_chr07	3457973	[G/A]	REGULATORY
OsMed31_1	LOC_Os07g07020	Os_chr07	3457969	[T/C]	REGULATORY
OsMed31_1	LOC_Os07g07020	Os_chr07	3457952	[C/T]	REGULATORY
OsMed31_1	LOC_Os07g07020	Os_chr07	3457941	[C/A]	REGULATORY
OsMed31_1	LOC_Os07g07020	Os_chr07	3457849	[G/A]	REGULATORY
OsMed31_1	LOC_Os07g07020	Os_chr07	3457841	[C/T]	REGULATORY
OsMed31_2	LOC_Os10g41450	Os_chr10	22209544	[C/T]	INTRON
OsMed31_2	LOC_Os10g41450	Os_chr10	22209200	[A/G]	INTRON
OsMed31_2	LOC_Os10g41450	Os_chr10	22209143	[T/A]	INTRON
OsMed31_2	LOC_Os10g41450	Os_chr10	22209092	[A/T]	INTRON
OsMed31_2	LOC_Os10g41450	Os_chr10	22209026	[C/G]	INTRON
OsMed31_2	LOC_Os10g41450	Os_chr10	22208618	[G/T]	INTRON
OsMed31_2	LOC_Os10g41450	Os_chr10	22207961	[T/A]	INTRON
OsMed31_2	LOC_Os10g41450	Os_chr10	22207950	[C/T]	INTRON
OsMed31_2	LOC_Os10g41450	Os_chr10	22207943	[G/A]	INTRON
OsMed31_2	LOC_Os10g41450	Os_chr10	22207942	[T/C]	INTRON
OsMed31_2	LOC_Os10g41450	Os_chr10	22207892	[T/A]	INTRON
OsMed31_2	LOC_Os10g41450	Os_chr10	22207737	[C/T]	INTRON
OsMed31_2	LOC_Os10g41450	Os_chr10	22207542	[A/G]	INTRON
OsMed31_2	LOC_Os10g41450	Os_chr10	22208947	[T/C]	NON-SYNONYMOUS-CODING
OsMed31_2	LOC_Os10g41450	Os_chr10	22208938	[T/A]	NON-SYNONYMOUS-CODING
OsMed31_2	LOC_Os10g41450	Os_chr10	22208266	[G/A]	NON-SYNONYMOUS-CODING
OsMed31_2	LOC_Os10g41450	Os_chr10	22209721	[C/T]	REGULATORY
OsMed31_2	LOC_Os10g41450	Os_chr10	22210829	[G/A]	REGULATORY
OsMed31_2	LOC_Os10g41450	Os_chr10	22210819	[T/C]	REGULATORY
OsMed31_2	LOC_Os10g41450	Os_chr10	22210818	[A/G]	REGULATORY
OsMed31_2	LOC_Os10g41450	Os_chr10	22210813	[G/A]	REGULATORY
OsMed31_2	LOC_Os10g41450	Os_chr10	22210744	[G/A]	REGULATORY
OsMed31_2	LOC_Os10g41450	Os_chr10	22210737	[G/T]	REGULATORY
OsMed31_2	LOC_Os10g41450	Os_chr10	22210733	[C/A]	REGULATORY
OsMed31_2	LOC_Os10g41450	Os_chr10	22210722	[G/A]	REGULATORY
OsMed31_2	LOC_Os10g41450	Os_chr10	22210714	[A/G]	REGULATORY
OsMed31_2	LOC_Os10g41450	Os_chr10	22210708	[C/T]	REGULATORY
OsMed31_2	LOC_Os10g41450	Os_chr10	22210706	[C/T]	REGULATORY
OsMed31_2	LOC_Os10g41450	Os_chr10	22210551	[A/G]	REGULATORY
OsMed31_2	LOC_Os10g41450	Os_chr10	22210528	[G/A]	REGULATORY
OsMed31_2	LOC_Os10g41450	Os_chr10	22210478	[A/G]	REGULATORY
OsMed31_2	LOC_Os10g41450	Os_chr10	22210463	[G/T]	REGULATORY
OsMed31_2	LOC_Os10g41450	Os_chr10	22210456	[A/G]	REGULATORY
OsMed31_2	LOC_Os10g41450	Os_chr10	22210452	[C/G]	REGULATORY
OsMed31_2	LOC_Os10g41450	Os_chr10	22210450	[C/T]	REGULATORY
OsMed31_2	LOC_Os10g41450	Os_chr10	22210436	[A/C]	REGULATORY

Mediator genes	MSU locus ID	Chromosomes	Physical Position (bp)	SNPs	Structural Annotation
OsMed31_2	LOC_Os10g41450	Os_chr10	22210434	[C/T]	REGULATORY
OsMed31_2	LOC_Os10g41450	Os_chr10	22210424	[G/A]	REGULATORY
OsMed31_2	LOC_Os10g41450	Os_chr10	22210404	[T/C]	REGULATORY
OsMed31_2	LOC_Os10g41450	Os_chr10	22210398	[T/C]	REGULATORY
OsMed31_2	LOC_Os10g41450	Os_chr10	22210394	[G/A]	REGULATORY
OsMed31_2	LOC_Os10g41450	Os_chr10	22210375	[C/T]	REGULATORY
OsMed31_2	LOC_Os10g41450	Os_chr10	22210369	[A/G]	REGULATORY
OsMed31_2	LOC_Os10g41450	Os_chr10	22210368	[C/T]	REGULATORY
OsMed31_2	LOC_Os10g41450	Os_chr10	22210355	[A/G]	REGULATORY
OsMed31_2	LOC_Os10g41450	Os_chr10	22210302	[C/T]	REGULATORY
OsMed31_2	LOC_Os10g41450	Os_chr10	22210297	[C/T]	REGULATORY
OsMed31_2	LOC_Os10g41450	Os_chr10	22210296	[G/A]	REGULATORY
OsMed31_2	LOC_Os10g41450	Os_chr10	22210183	[C/T]	REGULATORY
OsMed31_2	LOC_Os10g41450	Os_chr10	22210178	[T/C]	REGULATORY
OsMed31_2	LOC_Os10g41450	Os_chr10	22210159	[C/A]	REGULATORY
OsMed31_2	LOC_Os10g41450	Os_chr10	22210119	[C/T]	REGULATORY
OsMed31_2	LOC_Os10g41450	Os_chr10	22210095	[G/T]	REGULATORY
OsMed31_2	LOC_Os10g41450	Os_chr10	22210044	[G/A]	REGULATORY
OsMed31_2	LOC_Os10g41450	Os_chr10	22210014	[C/T]	REGULATORY
OsMed31_2	LOC_Os10g41450	Os_chr10	22209967	[G/A]	REGULATORY
OsMed31_2	LOC_Os10g41450	Os_chr10	22209957	[T/C]	REGULATORY
OsMed31_2	LOC_Os10g41450	Os_chr10	22209936	[C/T]	REGULATORY
OsMed31_2	LOC_Os10g41450	Os_chr10	22209909	[C/T]	REGULATORY
OsMed31_2	LOC_Os10g41450	Os_chr10	22209846	[C/T]	REGULATORY
OsMed34_1	LOC_Os04g35420	Os_chr04	21386178	[C/A]	INTRON
OsMed34_1	LOC_Os04g35420	Os_chr04	21386083	[A/C]	INTRON
OsMed34_1	LOC_Os04g35420	Os_chr04	21386044	[T/C]	INTRON
OsMed34_1	LOC_Os04g35420	Os_chr04	21385957	[C/A]	INTRON
OsMed34_1	LOC_Os04g35420	Os_chr04	21385927	[G/A]	INTRON
OsMed34_1	LOC_Os04g35420	Os_chr04	21385878	[C/A]	INTRON
OsMed34_1	LOC_Os04g35420	Os_chr04	21385732	[G/A]	INTRON
OsMed34_1	LOC_Os04g35420	Os_chr04	21385604	[T/C]	INTRON
OsMed34_1	LOC_Os04g35420	Os_chr04	21385489	[G/A]	INTRON
OsMed34_1	LOC_Os04g35420	Os_chr04	21385484	[C/A]	INTRON
OsMed34_1	LOC_Os04g35420	Os_chr04	21385454	[A/G]	INTRON
OsMed34_1	LOC_Os04g35420	Os_chr04	21385392	[C/G]	INTRON
OsMed34_1	LOC_Os04g35420	Os_chr04	21385014	[A/G]	INTRON
OsMed34_1	LOC_Os04g35420	Os_chr04	21385005	[A/G]	INTRON
OsMed34_1	LOC_Os04g35420	Os_chr04	21385004	[G/T]	INTRON
OsMed34_1	LOC_Os04g35420	Os_chr04	21384954	[G/A]	INTRON
OsMed34_1	LOC_Os04g35420	Os_chr04	21384886	[G/T]	INTRON
OsMed34_1	LOC_Os04g35420	Os_chr04	21384882	[G/A]	INTRON
OsMed34_1	LOC_Os04g35420	Os_chr04	21384880	[A/T]	INTRON
OsMed34_1	LOC_Os04g35420	Os_chr04	21384859	[A/T]	INTRON
OsMed34_1	LOC_Os04g35420	Os_chr04	21384831	[A/G]	INTRON
OsMed34_1	LOC_Os04g35420	Os_chr04	21384810	[G/A]	INTRON
OsMed34_1	LOC_Os04g35420	Os_chr04	21384784	[T/C]	INTRON

**Mediator genes**	**MSU locus ID**	**Chromosomes**	**Physical Position (bp)**	**SNPs**	**Structural Annotation**
OsMed34_1	LOC_Os04g35420	Os_chr04	21384725	[G/A]	INTRON
OsMed34_1	LOC_Os04g35420	Os_chr04	21384708	[T/G]	INTRON
OsMed34_1	LOC_Os04g35420	Os_chr04	21384682	[T/C]	INTRON
OsMed34_1	LOC_Os04g35420	Os_chr04	21384599	[G/A]	INTRON
OsMed34_1	LOC_Os04g35420	Os_chr04	21384586	[G/A]	INTRON
OsMed34_1	LOC_Os04g35420	Os_chr04	21384545	[C/T]	INTRON
OsMed34_1	LOC_Os04g35420	Os_chr04	21384527	[A/G]	INTRON
OsMed34_1	LOC_Os04g35420	Os_chr04	21384525	[C/T]	INTRON
OsMed34_1	LOC_Os04g35420	Os_chr04	21384514	[C/T]	INTRON
OsMed34_1	LOC_Os04g35420	Os_chr04	21384473	[G/A]	INTRON
OsMed34_1	LOC_Os04g35420	Os_chr04	21384281	[C/G]	INTRON
OsMed34_1	LOC_Os04g35420	Os_chr04	21384234	[A/G]	INTRON
OsMed34_1	LOC_Os04g35420	Os_chr04	21384205	[C/T]	INTRON
OsMed34_1	LOC_Os04g35420	Os_chr04	21384182	[C/T]	INTRON
OsMed34_1	LOC_Os04g35420	Os_chr04	21384106	[G/A]	INTRON
OsMed34_1	LOC_Os04g35420	Os_chr04	21384083	[G/A]	INTRON
OsMed34_1	LOC_Os04g35420	Os_chr04	21383928	[T/C]	INTRON
OsMed34_1	LOC_Os04g35420	Os_chr04	21383476	[G/A]	INTRON
OsMed34_1	LOC_Os04g35420	Os_chr04	21383112	[A/T]	INTRON
OsMed34_1	LOC_Os04g35420	Os_chr04	21382975	[C/A]	INTRON
OsMed34_1	LOC_Os04g35420	Os_chr04	21382920	[G/A]	INTRON
OsMed34_1	LOC_Os04g35420	Os_chr04	21382901	[C/T]	INTRON
OsMed34_1	LOC_Os04g35420	Os_chr04	21382828	[G/T]	INTRON
OsMed34_1	LOC_Os04g35420	Os_chr04	21382035	[G/A]	INTRON
OsMed34_1	LOC_Os04g35420	Os_chr04	21381948	[C/A]	INTRON
OsMed34_1	LOC_Os04g35420	Os_chr04	21381916	[G/A]	INTRON
OsMed34_1	LOC_Os04g35420	Os_chr04	21381863	[C/T]	INTRON
OsMed34_1	LOC_Os04g35420	Os_chr04	21381743	[G/A]	INTRON
OsMed34_1	LOC_Os04g35420	Os_chr04	21381653	[G/T]	INTRON
OsMed34_1	LOC_Os04g35420	Os_chr04	21381609	[C/T]	INTRON
OsMed34_1	LOC_Os04g35420	Os_chr04	21381581	[G/A]	INTRON
OsMed34_1	LOC_Os04g35420	Os_chr04	21381523	[G/T]	INTRON
OsMed34_1	LOC_Os04g35420	Os_chr04	21381086	[C/A]	INTRON
OsMed34_1	LOC_Os04g35420	Os_chr04	21381081	[C/T]	INTRON
OsMed34_1	LOC_Os04g35420	Os_chr04	21381080	[C/T]	INTRON
OsMed34_1	LOC_Os04g35420	Os_chr04	21381058	[A/G]	INTRON
OsMed34_1	LOC_Os04g35420	Os_chr04	21381009	[G/T]	INTRON
OsMed34_1	LOC_Os04g35420	Os_chr04	21381000	[G/T]	INTRON
OsMed34_1	LOC_Os04g35420	Os_chr04	21380528	[C/T]	INTRON
OsMed34_1	LOC_Os04g35420	Os_chr04	21380364	[T/A]	INTRON
OsMed34_1	LOC_Os04g35420	Os_chr04	21380223	[G/A]	INTRON
OsMed34_1	LOC_Os04g35420	Os_chr04	21380165	[T/C]	INTRON
OsMed34_1	LOC_Os04g35420	Os_chr04	21380144	[A/G]	INTRON
OsMed34_1	LOC_Os04g35420	Os_chr04	21380131	[G/A]	INTRON
OsMed34_1	LOC_Os04g35420	Os_chr04	21380126	[C/G]	INTRON
OsMed34_1	LOC_Os04g35420	Os_chr04	21380073	[G/A]	INTRON
OsMed34_1	LOC_Os04g35420	Os_chr04	21379851	[T/G]	INTRON

Mediator genes	MSU locus ID	Chromosomes	Physical Position (bp)	SNPs	Structural Annotation
OsMed34_1	LOC_Os04g35420	Os_chr04	21379848	[T/C]	INTRON
OsMed34_1	LOC_Os04g35420	Os_chr04	21379697	[T/C]	INTRON
OsMed34_1	LOC_Os04g35420	Os_chr04	21379692	[T/C]	INTRON
OsMed34_1	LOC_Os04g35420	Os_chr04	21379642	[A/G]	INTRON
OsMed34_1	LOC_Os04g35420	Os_chr04	21379487	[G/T]	INTRON
OsMed34_1	LOC_Os04g35420	Os_chr04	21379450	[A/T]	INTRON
OsMed34_1	LOC_Os04g35420	Os_chr04	21379310	[C/T]	INTRON
OsMed34_1	LOC_Os04g35420	Os_chr04	21379255	[G/A]	INTRON
OsMed34_1	LOC_Os04g35420	Os_chr04	21379234	[G/A]	INTRON
OsMed34_1	LOC_Os04g35420	Os_chr04	21378953	[G/C]	INTRON
OsMed34_1	LOC_Os04g35420	Os_chr04	21378938	[C/T]	INTRON
OsMed34_1	LOC_Os04g35420	Os_chr04	21378836	[C/T]	INTRON
OsMed34_1	LOC_Os04g35420	Os_chr04	21378817	[T/A]	INTRON
OsMed34_1	LOC_Os04g35420	Os_chr04	21378752	[C/A]	INTRON
OsMed34_1	LOC_Os04g35420	Os_chr04	21378577	[A/G]	INTRON
OsMed34_1	LOC_Os04g35420	Os_chr04	21378436	[G/A]	INTRON
OsMed34_1	LOC_Os04g35420	Os_chr04	21378287	[A/G]	INTRON
OsMed34_1	LOC_Os04g35420	Os_chr04	21378238	[A/T]	INTRON
OsMed34_1	LOC_Os04g35420	Os_chr04	21378230	[C/T]	INTRON
OsMed34_1	LOC_Os04g35420	Os_chr04	21378068	[A/T]	INTRON
OsMed34_1	LOC_Os04g35420	Os_chr04	21377838	[C/A]	INTRON
OsMed34_1	LOC_Os04g35420	Os_chr04	21377562	[C/T]	INTRON
OsMed34_1	LOC_Os04g35420	Os_chr04	21377548	[C/T]	INTRON
OsMed34_1	LOC_Os04g35420	Os_chr04	21377043	[G/T]	INTRON
OsMed34_1	LOC_Os04g35420	Os_chr04	21377023	[A/G]	INTRON
OsMed34_1	LOC_Os04g35420	Os_chr04	21376663	[C/T]	INTRON
OsMed34_1	LOC_Os04g35420	Os_chr04	21376567	[T/C]	INTRON
OsMed34_1	LOC_Os04g35420	Os_chr04	21376555	[G/A]	INTRON
OsMed34_1	LOC_Os04g35420	Os_chr04	21376522	[G/T]	INTRON
OsMed34_1	LOC_Os04g35420	Os_chr04	21376490	[C/T]	INTRON
OsMed34_1	LOC_Os04g35420	Os_chr04	21376477	[A/G]	INTRON
OsMed34_1	LOC_Os04g35420	Os_chr04	21376419	[C/T]	INTRON
OsMed34_1	LOC_Os04g35420	Os_chr04	21376330	[C/G]	INTRON
OsMed34_1	LOC_Os04g35420	Os_chr04	21375868	[G/A]	INTRON
OsMed34_1	LOC_Os04g35420	Os_chr04	21375846	[G/T]	INTRON
OsMed34_1	LOC_Os04g35420	Os_chr04	21383778	[C/T]	NON-SYNONYMOUS-CODING
OsMed34_1	LOC_Os04g35420	Os_chr04	21383769	[T/A]	NON-SYNONYMOUS-CODING
OsMed34_1	LOC_Os04g35420	Os_chr04	21383688	[T/C]	NON-SYNONYMOUS-CODING
OsMed34_1	LOC_Os04g35420	Os_chr04	21383600	[T/A]	NON-SYNONYMOUS-CODING
OsMed34_1	LOC_Os04g35420	Os_chr04	21383555	[G/T]	NON-SYNONYMOUS-CODING
OsMed34_1	LOC_Os04g35420	Os_chr04	21383259	[C/T]	NON-SYNONYMOUS-CODING
OsMed34_1	LOC_Os04g35420	Os_chr04	21383101	[C/G]	NON-SYNONYMOUS-CODING
OsMed34_1	LOC_Os04g35420	Os_chr04	21383085	[G/A]	NON-SYNONYMOUS-CODING
OsMed34_1	LOC_Os04g35420	Os_chr04	21382999	[C/T]	NON-SYNONYMOUS-CODING
OsMed34_1	LOC_Os04g35420	Os_chr04	21382442	[C/T]	NON-SYNONYMOUS-CODING
OsMed34_1	LOC_Os04g35420	Os_chr04	21381267	[G/A]	NON-SYNONYMOUS-CODING
OsMed34_1	LOC_Os04g35420	Os_chr04	21380904	[A/C]	NON-SYNONYMOUS-CODING

Mediator genes	MSU locus ID	Chromosomes	Physical Position (bp)	SNPs	Structural Annotation
OsMed34_1	LOC_Os04g35420	Os_chr04	21378714	[T/G]	NON-SYNONYMOUS-CODING
OsMed34_1	LOC_Os04g35420	Os_chr04	21378336	[A/T]	NON-SYNONYMOUS-CODING
OsMed34_1	LOC_Os04g35420	Os_chr04	21378161	[G/T]	NON-SYNONYMOUS-CODING
OsMed34_1	LOC_Os04g35420	Os_chr04	21377894	[C/T]	NON-SYNONYMOUS-CODING
OsMed34_1	LOC_Os04g35420	Os_chr04	21375929	[T/C]	NON-SYNONYMOUS-CODING
OsMed34_1	LOC_Os04g35420	Os_chr04	21375467	[T/C]	NON-SYNONYMOUS-CODING
OsMed34_1	LOC_Os04g35420	Os_chr04	21375452	[C/T]	NON-SYNONYMOUS-CODING
OsMed34_1	LOC_Os04g35420	Os_chr04	21385334	[C/T]	REGULATORY
OsMed34_1	LOC_Os04g35420	Os_chr04	21386319	[G/A]	REGULATORY
OsMed34_1	LOC_Os04g35420	Os_chr04	21385297	[G/A]	REGULATORY
OsMed34_1	LOC_Os04g35420	Os_chr04	21385268	[C/A]	REGULATORY
OsMed34_1	LOC_Os04g35420	Os_chr04	21375387	[T/G]	REGULATORY
OsMed34_1	LOC_Os04g35420	Os_chr04	21375299	[G/A]	REGULATORY
OsMed34_1	LOC_Os04g35420	Os_chr04	21375242	[C/A]	REGULATORY
OsMed34_1	LOC_Os04g35420	Os_chr04	21375224	[T/C]	REGULATORY
OsMed34_1	LOC_Os04g35420	Os_chr04	21375157	[C/T]	REGULATORY
OsMed34_1	LOC_Os04g35420	Os_chr04	21375152	[C/A]	REGULATORY
OsMed34_1	LOC_Os04g35420	Os_chr04	21375130	[A/C]	REGULATORY
OsMed34_1	LOC_Os04g35420	Os_chr04	21375114	[G/A]	REGULATORY
OsMed34_1	LOC_Os04g35420	Os_chr04	21375103	[C/T]	REGULATORY
OsMed34_1	LOC_Os04g35430	Os_chr04	21387334	[C/T]	REGULATORY
OsMed34_1	LOC_Os04g35430	Os_chr04	21387302	[T/C]	REGULATORY
OsMed34_1	LOC_Os04g35430	Os_chr04	21387287	[A/G]	REGULATORY
OsMed34_1	LOC_Os04g35430	Os_chr04	21387285	[C/A]	REGULATORY
OsMed34_1	LOC_Os04g35420	Os_chr04	21387264	[A/C]	REGULATORY
OsMed34_1	LOC_Os04g35420	Os_chr04	21387258	[T/C]	REGULATORY
OsMed34_1	LOC_Os04g35420	Os_chr04	21387247	[A/G]	REGULATORY
OsMed34_1	LOC_Os04g35420	Os_chr04	21387219	[A/G]	REGULATORY
OsMed34_1	LOC_Os04g35420	Os_chr04	21387197	[A/C]	REGULATORY
OsMed34_1	LOC_Os04g35420	Os_chr04	21387165	[G/T]	REGULATORY
OsMed34_1	LOC_Os04g35420	Os_chr04	21387147	[C/T]	REGULATORY
OsMed34_1	LOC_Os04g35420	Os_chr04	21387130	[G/A]	REGULATORY
OsMed34_1	LOC_Os04g35420	Os_chr04	21387127	[G/A]	REGULATORY
OsMed34_1	LOC_Os04g35420	Os_chr04	21387108	[A/T]	REGULATORY
OsMed34_1	LOC_Os04g35420	Os_chr04	21387101	[C/A]	REGULATORY
OsMed34_1	LOC_Os04g35420	Os_chr04	21387088	[G/A]	REGULATORY
OsMed34_1	LOC_Os04g35420	Os_chr04	21387083	[G/A]	REGULATORY
OsMed34_1	LOC_Os04g35420	Os_chr04	21387082	[C/T]	REGULATORY
OsMed34_1	LOC_Os04g35420	Os_chr04	21387081	[G/A]	REGULATORY
OsMed34_1	LOC_Os04g35420	Os_chr04	21387077	[A/C]	REGULATORY
OsMed34_1	LOC_Os04g35420	Os_chr04	21387071	[G/A]	REGULATORY
OsMed34_1	LOC_Os04g35420	Os_chr04	21387062	[T/C]	REGULATORY
OsMed34_1	LOC_Os04g35420	Os_chr04	21387026	[A/T]	REGULATORY
OsMed34_1	LOC_Os04g35420	Os_chr04	21387015	[G/A]	REGULATORY
OsMed34_1	LOC_Os04g35420	Os_chr04	21387007	[G/A]	REGULATORY
OsMed34_1	LOC_Os04g35420	Os_chr04	21387006	[C/T]	REGULATORY
OsMed34_1	LOC_Os04g35420	Os_chr04	21386995	[C/T]	REGULATORY

**Mediator genes**	**MSU locus ID**	**Chromosomes**	**Physical Position (bp)**	**SNPs**	**Structural Annotation**
OsMed34_1	LOC_Os04g35420	Os_chr04	21386991	[C/T]	REGULATORY
OsMed34_1	LOC_Os04g35420	Os_chr04	21386949	[G/T]	REGULATORY
OsMed34_1	LOC_Os04g35420	Os_chr04	21386947	[G/A]	REGULATORY
OsMed34_1	LOC_Os04g35420	Os_chr04	21386932	[G/A]	REGULATORY
OsMed34_1	LOC_Os04g35420	Os_chr04	21386915	[A/T]	REGULATORY
OsMed34_1	LOC_Os04g35420	Os_chr04	21386862	[A/G]	REGULATORY
OsMed34_1	LOC_Os04g35420	Os_chr04	21386859	[G/T]	REGULATORY
OsMed34_1	LOC_Os04g35420	Os_chr04	21386839	[T/A]	REGULATORY
OsMed34_1	LOC_Os04g35420	Os_chr04	21386810	[C/A]	REGULATORY
OsMed34_1	LOC_Os04g35420	Os_chr04	21386805	[C/T]	REGULATORY
OsMed34_1	LOC_Os04g35420	Os_chr04	21386801	[C/T]	REGULATORY
OsMed34_1	LOC_Os04g35420	Os_chr04	21386791	[C/T]	REGULATORY
OsMed34_1	LOC_Os04g35420	Os_chr04	21386788	[C/T]	REGULATORY
OsMed34_1	LOC_Os04g35420	Os_chr04	21386783	[C/T]	REGULATORY
OsMed34_1	LOC_Os04g35420	Os_chr04	21386757	[T/C]	REGULATORY
OsMed34_1	LOC_Os04g35420	Os_chr04	21386725	[C/A]	REGULATORY
OsMed34_1	LOC_Os04g35420	Os_chr04	21386579	[T/G]	REGULATORY
OsMed34_1	LOC_Os04g35420	Os_chr04	21386578	[G/A]	REGULATORY
OsMed34_1	LOC_Os04g35420	Os_chr04	21386474	[C/T]	REGULATORY
OsMed34_1	LOC_Os04g35420	Os_chr04	21383869	[C/A]	SYNONYMOUS-CODING
OsMed34_1	LOC_Os04g35420	Os_chr04	21383818	[G/A]	SYNONYMOUS-CODING
OsMed34_1	LOC_Os04g35420	Os_chr04	21383000	[A/G]	SYNONYMOUS-CODING
OsMed34_1	LOC_Os04g35420	Os_chr04	21382672	[G/A]	SYNONYMOUS-CODING
OsMed34_1	LOC_Os04g35420	Os_chr04	21381471	[A/T]	SYNONYMOUS-CODING
OsMed34_1	LOC_Os04g35420	Os_chr04	21381299	[C/T]	SYNONYMOUS-CODING
OsMed34_1	LOC_Os04g35420	Os_chr04	21380908	[T/C]	SYNONYMOUS-CODING
OsMed34_1	LOC_Os04g35420	Os_chr04	21380480	[G/A]	SYNONYMOUS-CODING
OsMed34_1	LOC_Os04g35420	Os_chr04	21378680	[C/T]	SYNONYMOUS-CODING
OsMed34_1	LOC_Os04g35420	Os_chr04	21376119	[G/C]	SYNONYMOUS-CODING
OsMed34_1	LOC_Os04g35420	Os_chr04	21376062	[T/C]	SYNONYMOUS-CODING
OsMed34_1	LOC_Os04g35420	Os_chr04	21375759	[A/G]	SYNONYMOUS-CODING
OsMed34_2	LOC_Os11g48090	Os_chr11	28477271	[A/T]	INTRON
OsMed34_2	LOC_Os11g48090	Os_chr11	28477176	[T/C]	INTRON
OsMed34_2	LOC_Os11g48090	Os_chr11	28477036	[T/G]	INTRON
OsMed34_2	LOC_Os11g48090	Os_chr11	28477009	[G/A]	INTRON
OsMed34_2	LOC_Os11g48090	Os_chr11	28476605	[T/G]	INTRON
OsMed34_2	LOC_Os11g48090	Os_chr11	28476581	[T/A]	INTRON
OsMed34_2	LOC_Os11g48090	Os_chr11	28476435	[C/A]	INTRON
OsMed34_2	LOC_Os11g48090	Os_chr11	28476304	[A/G]	INTRON
OsMed34_2	LOC_Os11g48090	Os_chr11	28476291	[G/A]	INTRON
OsMed34_2	LOC_Os11g48090	Os_chr11	28476267	[C/T]	INTRON
OsMed34_2	LOC_Os11g48090	Os_chr11	28476246	[C/A]	INTRON
OsMed34_2	LOC_Os11g48090	Os_chr11	28475766	[T/C]	INTRON
OsMed34_2	LOC_Os11g48090	Os_chr11	28475445	[G/A]	INTRON
OsMed34_2	LOC_Os11g48090	Os_chr11	28475428	[A/T]	INTRON
OsMed34_2	LOC_Os11g48090	Os_chr11	28475427	[G/C]	INTRON
OsMed34_2	LOC_Os11g48090	Os_chr11	28475424	[C/T]	INTRON

**Mediator genes**	**MSU locus ID**	**Chromosomes**	**Physical Position (bp)**	**SNPs**	**Structural Annotation**
OsMed34_2	LOC_Os11g48090	Os_chr11	28475319	[T/A]	INTRON
OsMed34_2	LOC_Os11g48090	Os_chr11	28475153	[A/G]	INTRON
OsMed34_2	LOC_Os11g48090	Os_chr11	28475080	[G/C]	INTRON
OsMed34_2	LOC_Os11g48090	Os_chr11	28475071	[G/A]	INTRON
OsMed34_2	LOC_Os11g48090	Os_chr11	28474913	[A/G]	INTRON
OsMed34_2	LOC_Os11g48090	Os_chr11	28474766	[G/C]	INTRON
OsMed34_2	LOC_Os11g48090	Os_chr11	28474740	[G/A]	INTRON
OsMed34_2	LOC_Os11g48090	Os_chr11	28474736	[C/A]	INTRON
OsMed34_2	LOC_Os11g48090	Os_chr11	28474720	[G/A]	INTRON
OsMed34_2	LOC_Os11g48090	Os_chr11	28474298	[G/A]	INTRON
OsMed34_2	LOC_Os11g48090	Os_chr11	28474271	[C/A]	INTRON
OsMed34_2	LOC_Os11g48090	Os_chr11	28474249	[C/G]	INTRON
OsMed34_2	LOC_Os11g48090	Os_chr11	28474091	[T/C]	INTRON
OsMed34_2	LOC_Os11g48090	Os_chr11	28473678	[G/A]	INTRON
OsMed34_2	LOC_Os11g48090	Os_chr11	28473623	[G/A]	INTRON
OsMed34_2	LOC_Os11g48090	Os_chr11	28473619	[C/T]	INTRON
OsMed34_2	LOC_Os11g48090	Os_chr11	28473602	[G/A]	INTRON
OsMed34_2	LOC_Os11g48090	Os_chr11	28473543	[G/A]	INTRON
OsMed34_2	LOC_Os11g48090	Os_chr11	28473489	[C/A]	INTRON
OsMed34_2	LOC_Os11g48090	Os_chr11	28473018	[A/G]	INTRON
OsMed34_2	LOC_Os11g48090	Os_chr11	28472640	[T/C]	INTRON
OsMed34_2	LOC_Os11g48090	Os_chr11	28472614	[A/G]	INTRON
OsMed34_2	LOC_Os11g48090	Os_chr11	28472590	[T/C]	INTRON
OsMed34_2	LOC_Os11g48090	Os_chr11	28472469	[T/C]	INTRON
OsMed34_2	LOC_Os11g48090	Os_chr11	28472349	[T/C]	INTRON
OsMed34_2	LOC_Os11g48090	Os_chr11	28472348	[T/A]	INTRON
OsMed34_2	LOC_Os11g48090	Os_chr11	28472029	[A/G]	INTRON
OsMed34_2	LOC_Os11g48090	Os_chr11	28471972	[A/T]	INTRON
OsMed34_2	LOC_Os11g48090	Os_chr11	28471776	[G/A]	INTRON
OsMed34_2	LOC_Os11g48090	Os_chr11	28471735	[C/T]	INTRON
OsMed34_2	LOC_Os11g48090	Os_chr11	28471684	[C/T]	INTRON
OsMed34_2	LOC_Os11g48090	Os_chr11	28471658	[C/T]	INTRON
OsMed34_2	LOC_Os11g48090	Os_chr11	28471620	[C/G]	INTRON
OsMed34_2	LOC_Os11g48090	Os_chr11	28471573	[C/T]	INTRON
OsMed34_2	LOC_Os11g48090	Os_chr11	28471465	[G/A]	INTRON
OsMed34_2	LOC_Os11g48090	Os_chr11	28471398	[G/A]	INTRON
OsMed34_2	LOC_Os11g48090	Os_chr11	28471253	[C/T]	INTRON
OsMed34_2	LOC_Os11g48090	Os_chr11	28471237	[C/T]	INTRON
OsMed34_2	LOC_Os11g48090	Os_chr11	28471122	[T/G]	INTRON
OsMed34_2	LOC_Os11g48090	Os_chr11	28470818	[A/G]	INTRON
OsMed34_2	LOC_Os11g48090	Os_chr11	28470675	[G/A]	INTRON
OsMed34_2	LOC_Os11g48090	Os_chr11	28470619	[C/T]	INTRON
OsMed34_2	LOC_Os11g48090	Os_chr11	28470604	[C/A]	INTRON
OsMed34_2	LOC_Os11g48090	Os_chr11	28470275	[T/A]	INTRON
OsMed34_2	LOC_Os11g48090	Os_chr11	28470076	[T/A]	INTRON
OsMed34_2	LOC_Os11g48090	Os_chr11	28470058	[A/G]	INTRON
OsMed34_2	LOC_Os11g48090	Os_chr11	28470046	[G/A]	INTRON

Mediator genes	MSU locus ID	Chromosomes	Physical Position (bp)	SNPs	Structural Annotation
OsMed34_2	LOC_Os11g48090	Os_chr11	28469664	[C/A]	INTRON
OsMed34_2	LOC_Os11g48090	Os_chr11	28469288	[G/C]	INTRON
OsMed34_2	LOC_Os11g48090	Os_chr11	28468721	[C/A]	INTRON
OsMed34_2	LOC_Os11g48090	Os_chr11	28468314	[T/C]	INTRON
OsMed34_2	LOC_Os11g48090	Os_chr11	28468074	[T/C]	INTRON
OsMed34_2	LOC_Os11g48090	Os_chr11	28467910	[G/C]	INTRON
OsMed34_2	LOC_Os11g48090	Os_chr11	28467682	[T/C]	INTRON
OsMed34_2	LOC_Os11g48090	Os_chr11	28467512	[C/A]	INTRON
OsMed34_2	LOC_Os11g48090	Os_chr11	28467320	[A/G]	INTRON
OsMed34_2	LOC_Os11g48090	Os_chr11	28467313	[T/C]	INTRON
OsMed34_2	LOC_Os11g48090	Os_chr11	28467294	[G/T]	INTRON
OsMed34_2	LOC_Os11g48090	Os_chr11	28467108	[G/A]	INTRON
OsMed34_2	LOC_Os11g48090	Os_chr11	28467104	[C/T]	INTRON
OsMed34_2	LOC_Os11g48090	Os_chr11	28467101	[G/T]	INTRON
OsMed34_2	LOC_Os11g48090	Os_chr11	28467004	[A/T]	INTRON
OsMed34_2	LOC_Os11g48090	Os_chr11	28466990	[G/C]	INTRON
OsMed34_2	LOC_Os11g48090	Os_chr11	28466596	[T/A]	INTRON
OsMed34_2	LOC_Os11g48090	Os_chr11	28466589	[T/C]	INTRON
OsMed34_2	LOC_Os11g48090	Os_chr11	28468481	[C/A]	NON-SYNONYMOUS-CODING
OsMed34_2	LOC_Os11g48090	Os_chr11	28474560	[C/G]	NON-SYNONYMOUS-CODING
OsMed34_2	LOC_Os11g48090	Os_chr11	28473794	[C/T]	NON-SYNONYMOUS-CODING
OsMed34_2	LOC_Os11g48090	Os_chr11	28473257	[G/A]	NON-SYNONYMOUS-CODING
OsMed34_2	LOC_Os11g48090	Os_chr11	28472227	[C/T]	NON-SYNONYMOUS-CODING
OsMed34_2	LOC_Os11g48090	Os_chr11	28472217	[T/A]	NON-SYNONYMOUS-CODING
OsMed34_2	LOC_Os11g48090	Os_chr11	28467148	[T/G]	NON-SYNONYMOUS-CODING
OsMed34_2	LOC_Os11g48090	Os_chr11	28466536	[A/C]	NON-SYNONYMOUS-CODING
OsMed34_2	LOC_Os11g48090	Os_chr11	28466495	[C/T]	NON-SYNONYMOUS-CODING
OsMed34_2	LOC_Os11g48090	Os_chr11	28466452	[C/T]	NON-SYNONYMOUS-CODING
OsMed34_2	LOC_Os11g48090	Os_chr11	28477658	[G/T]	REGULATORY
OsMed34_2	LOC_Os11g48090	Os_chr11	28477548	[C/T]	REGULATORY
OsMed34_2	LOC_Os11g48090	Os_chr11	28477524	[G/C]	REGULATORY
OsMed34_2	LOC_Os11g48090	Os_chr11	28477466	[G/A]	REGULATORY
OsMed34_2	LOC_Os11g48090	Os_chr11	28477410	[G/A]	REGULATORY
OsMed34_2	LOC_Os11g48090	Os_chr11	28477684	[T/C]	REGULATORY
OsMed34_2	LOC_Os11g48090	Os_chr11	28466388	[G/C]	REGULATORY
OsMed34_2	LOC_Os11g48090	Os_chr11	28466303	[G/A]	REGULATORY
OsMed34_2	LOC_Os11g48090	Os_chr11	28478684	[C/T]	REGULATORY
OsMed34_2	LOC_Os11g48090	Os_chr11	28478651	[C/T]	REGULATORY
OsMed34_2	LOC_Os11g48090	Os_chr11	28478611	[C/T]	REGULATORY
OsMed34_2	LOC_Os11g48090	Os_chr11	28478573	[C/T]	REGULATORY
OsMed34_2	LOC_Os11g48090	Os_chr11	28478570	[G/T]	REGULATORY
OsMed34_2	LOC_Os11g48090	Os_chr11	28478544	[G/T]	REGULATORY
OsMed34_2	LOC_Os11g48090	Os_chr11	28478510	[C/T]	REGULATORY
OsMed34_2	LOC_Os11g48090	Os_chr11	28478505	[T/G]	REGULATORY
OsMed34_2	LOC_Os11g48090	Os_chr11	28478500	[G/A]	REGULATORY
OsMed34_2	LOC_Os11g48090	Os_chr11	28478495	[G/A]	REGULATORY
OsMed34_2	LOC_Os11g48090	Os_chr11	28478488	[G/A]	REGULATORY

Mediator genes	MSU locus ID	Chromosomes	Physical Position (bp)	SNPs	Structural Annotation
OsMed34_2	LOC_Os11g48090	Os_chr11	28478482	[C/T]	REGULATORY
OsMed34_2	LOC_Os11g48090	Os_chr11	28478470	[G/A]	REGULATORY
OsMed34_2	LOC_Os11g48090	Os_chr11	28478445	[G/T]	REGULATORY
OsMed34_2	LOC_Os11g48090	Os_chr11	28478435	[G/A]	REGULATORY
OsMed34_2	LOC_Os11g48090	Os_chr11	28478422	[C/T]	REGULATORY
OsMed34_2	LOC_Os11g48090	Os_chr11	28478407	[A/G]	REGULATORY
OsMed34_2	LOC_Os11g48090	Os_chr11	28478404	[G/A]	REGULATORY
OsMed34_2	LOC_Os11g48090	Os_chr11	28478383	[A/C]	REGULATORY
OsMed34_2	LOC_Os11g48090	Os_chr11	28478380	[T/C]	REGULATORY
OsMed34_2	LOC_Os11g48090	Os_chr11	28478373	[T/A]	REGULATORY
OsMed34_2	LOC_Os11g48090	Os_chr11	28478355	[A/C]	REGULATORY
OsMed34_2	LOC_Os11g48090	Os_chr11	28478328	[C/T]	REGULATORY
OsMed34_2	LOC_Os11g48090	Os_chr11	28478316	[G/A]	REGULATORY
OsMed34_2	LOC_Os11g48090	Os_chr11	28478296	[G/A]	REGULATORY
OsMed34_2	LOC_Os11g48090	Os_chr11	28478278	[C/A]	REGULATORY
OsMed34_2	LOC_Os11g48090	Os_chr11	28478277	[A/C]	REGULATORY
OsMed34_2	LOC_Os11g48090	Os_chr11	28478223	[C/T]	REGULATORY
OsMed34_2	LOC_Os11g48090	Os_chr11	28478096	[G/A]	REGULATORY
OsMed34_2	LOC_Os11g48090	Os_chr11	28478072	[C/T]	REGULATORY
OsMed34_2	LOC_Os11g48090	Os_chr11	28478028	[G/T]	REGULATORY
OsMed34_2	LOC_Os11g48090	Os_chr11	28478025	[G/T]	REGULATORY
OsMed34_2	LOC_Os11g48090	Os_chr11	28477942	[C/T]	REGULATORY
OsMed34_2	LOC_Os11g48090	Os_chr11	28477936	[G/A]	REGULATORY
OsMed34_2	LOC_Os11g48090	Os_chr11	28477926	[G/A]	REGULATORY
OsMed34_2	LOC_Os11g48090	Os_chr11	28477899	[G/T]	REGULATORY
OsMed34_2	LOC_Os11g48090	Os_chr11	28477897	[G/A]	REGULATORY
OsMed34_2	LOC_Os11g48090	Os_chr11	28477895	[G/A]	REGULATORY
OsMed34_2	LOC_Os11g48090	Os_chr11	28477864	[C/T]	REGULATORY
OsMed34_2	LOC_Os11g48090	Os_chr11	28477855	[C/T]	REGULATORY
OsMed34_2	LOC_Os11g48090	Os_chr11	28477710	[A/G]	REGULATORY
OsMed34_2	LOC_Os11g48090	Os_chr11	28468468	[G/A]	SYNONYMOUS-CODING
OsMed34_2	LOC_Os11g48090	Os_chr11	28474564	[A/C]	SYNONYMOUS-CODING
OsMed34_2	LOC_Os11g48090	Os_chr11	28474510	[A/T]	SYNONYMOUS-CODING
OsMed34_2	LOC_Os11g48090	Os_chr11	28473292	[A/G]	SYNONYMOUS-CODING
OsMed34_2	LOC_Os11g48090	Os_chr11	28473256	[C/T]	SYNONYMOUS-CODING
OsMed34_2	LOC_Os11g48090	Os_chr11	28469429	[A/C]	SYNONYMOUS-CODING
OsMed34_2	LOC_Os11g48090	Os_chr11	28468420	[G/A]	SYNONYMOUS-CODING
OsMed34_2	LOC_Os11g48090	Os_chr11	28466496	[A/G]	SYNONYMOUS-CODING
OsMed34_2	LOC_Os11g48090	Os_chr11	28466493	[G/A]	SYNONYMOUS-CODING
OsMed35_1	LOC_Os10g34380	Os_chr10	18259575	[C/T]	INTRON
OsMed35_1	LOC_Os10g34380	Os_chr10	18259346	[A/G]	INTRON
OsMed35_1	LOC_Os10g34380	Os_chr10	18259220	[G/A]	INTRON
OsMed35_1	LOC_Os10g34380	Os_chr10	18259045	[G/A]	INTRON
OsMed35_1	LOC_Os10g34380	Os_chr10	18259010	[A/G]	INTRON
OsMed35_1	LOC_Os10g34380	Os_chr10	18258960	[T/C]	INTRON
OsMed35_1	LOC_Os10g34380	Os_chr10	18258937	[G/A]	INTRON
OsMed35_1	LOC_Os10g34380	Os_chr10	18258588	[A/G]	INTRON

**Mediator genes**	**MSU locus ID**	**Chromosomes**	**Physical Position (bp)**	**SNPs**	**Structural Annotation**
OsMed35_1	LOC_Os10g34380	Os_chr10	18257034	[A/G]	INTRON
OsMed35_1	LOC_Os10g34380	Os_chr10	18256652	[T/A]	INTRON
OsMed35_1	LOC_Os10g34380	Os_chr10	18256549	[G/A]	INTRON
OsMed35_1	LOC_Os10g34380	Os_chr10	18256498	[C/G]	INTRON
OsMed35_1	LOC_Os10g34380	Os_chr10	18256496	[C/T]	INTRON
OsMed35_1	LOC_Os10g34380	Os_chr10	18256369	[C/T]	INTRON
OsMed35_1	LOC_Os10g34380	Os_chr10	18256314	[T/C]	INTRON
OsMed35_1	LOC_Os10g34380	Os_chr10	18256196	[G/A]	INTRON
OsMed35_1	LOC_Os10g34380	Os_chr10	18254291	[A/C]	INTRON
OsMed35_1	LOC_Os10g34380	Os_chr10	18254183	[C/T]	INTRON
OsMed35_1	LOC_Os10g34380	Os_chr10	18254151	[A/G]	INTRON
OsMed35_1	LOC_Os10g34380	Os_chr10	18254113	[G/A]	INTRON
OsMed35_1	LOC_Os10g34380	Os_chr10	18253983	[A/T]	INTRON
OsMed35_1	LOC_Os10g34380	Os_chr10	18253836	[T/G]	INTRON
OsMed35_1	LOC_Os10g34380	Os_chr10	18253733	[G/A]	INTRON
OsMed35_1	LOC_Os10g34380	Os_chr10	18253729	[G/A]	INTRON
OsMed35_1	LOC_Os10g34380	Os_chr10	18253405	[C/A]	INTRON
OsMed35_1	LOC_Os10g34380	Os_chr10	18253180	[G/A]	INTRON
OsMed35_1	LOC_Os10g34380	Os_chr10	18252980	[A/G]	INTRON
OsMed35_1	LOC_Os10g34380	Os_chr10	18252956	[C/A]	INTRON
OsMed35_1	LOC_Os10g34380	Os_chr10	18252485	[G/A]	INTRON
OsMed35_1	LOC_Os10g34380	Os_chr10	18252453	[G/A]	INTRON
OsMed35_1	LOC_Os10g34380	Os_chr10	18252113	[G/A]	INTRON
OsMed35_1	LOC_Os10g34380	Os_chr10	18251966	[T/C]	INTRON
OsMed35_1	LOC_Os10g34380	Os_chr10	18251719	[C/T]	INTRON
OsMed35_1	LOC_Os10g34380	Os_chr10	18251697	[C/T]	INTRON
OsMed35_1	LOC_Os10g34380	Os_chr10	18251513	[G/T]	INTRON
OsMed35_1	LOC_Os10g34380	Os_chr10	18251319	[C/T]	INTRON
OsMed35_1	LOC_Os10g34380	Os_chr10	18251202	[T/C]	INTRON
OsMed35_1	LOC_Os10g34380	Os_chr10	18250994	[T/A]	INTRON
OsMed35_1	LOC_Os10g34380	Os_chr10	18250982	[G/C]	INTRON
OsMed35_1	LOC_Os10g34380	Os_chr10	18250162	[G/A]	INTRON
OsMed35_1	LOC_Os10g34380	Os_chr10	18260166	[A/G]	NON-SYNONYMOUS-CODING
OsMed35_1	LOC_Os10g34380	Os_chr10	18258021	[C/T]	NON-SYNONYMOUS-CODING
OsMed35_1	LOC_Os10g34380	Os_chr10	18249910	[T/C]	NON-SYNONYMOUS-CODING
OsMed35_1	LOC_Os10g34380	Os_chr10	18260267	[G/A]	REGULATORY
OsMed35_1	LOC_Os10g34380	Os_chr10	18258510	[G/T]	SYNONYMOUS-CODING
OsMed35_1	LOC_Os10g34380	Os_chr10	18258154	[G/A]	SYNONYMOUS-CODING
OsMed35_1	LOC_Os10g34380	Os_chr10	18257917	[C/A]	SYNONYMOUS-CODING
OsMed35_1	LOC_Os10g34380	Os_chr10	18257758	[C/A]	SYNONYMOUS-CODING
OsMed35_1	LOC_Os10g34380	Os_chr10	18257440	[T/C]	SYNONYMOUS-CODING
OsMed35_1	LOC_Os10g34380	Os_chr10	18250895	[T/C]	SYNONYMOUS-CODING
OsMed35_1	LOC_Os10g34380	Os_chr10	18250008	[G/A]	SYNONYMOUS-CODING
OsMed35_1	LOC_Os10g34380	Os_chr10	18260979	[T/C]	REGULATORY
OsMed35_1	LOC_Os10g34380	Os_chr10	18260963	[C/T]	REGULATORY
OsMed35_1	LOC_Os10g34380	Os_chr10	18260957	[C/T]	REGULATORY
OsMed35_1	LOC_Os10g34380	Os_chr10	18260944	[A/T]	REGULATORY

Mediator genes	MSU locus ID	Chromosomes	Physical Position (bp)	SNPs	Structural Annotation
OsMed35_1	LOC_Os10g34380	Os_chr10	18260918	[C/T]	REGULATORY
OsMed35_1	LOC_Os10g34380	Os_chr10	18260856	[T/G]	REGULATORY
OsMed35_1	LOC_Os10g34380	Os_chr10	18260846	[A/G]	REGULATORY
OsMed35_1	LOC_Os10g34380	Os_chr10	18260845	[C/T]	REGULATORY
OsMed35_1	LOC_Os10g34380	Os_chr10	18260838	[A/G]	REGULATORY
OsMed35_1	LOC_Os10g34380	Os_chr10	18260794	[G/A]	REGULATORY
OsMed35_1	LOC_Os10g34380	Os_chr10	18260785	[C/T]	REGULATORY
OsMed35_1	LOC_Os10g34380	Os_chr10	18260741	[A/G]	REGULATORY
OsMed35_1	LOC_Os10g34380	Os_chr10	18260725	[G/T]	REGULATORY
OsMed35_1	LOC_Os10g34380	Os_chr10	18260701	[C/T]	REGULATORY
OsMed35_1	LOC_Os10g34380	Os_chr10	18260654	[G/T]	REGULATORY
OsMed35_1	LOC_Os10g34380	Os_chr10	18260627	[G/A]	REGULATORY
OsMed35_1	LOC_Os10g34380	Os_chr10	18260586	[C/T]	REGULATORY
OsMed35_1	LOC_Os10g34380	Os_chr10	18260535	[C/T]	REGULATORY
OsMed35_1	LOC_Os10g34380	Os_chr10	18260506	[G/A]	REGULATORY
OsMed35_1	LOC_Os10g34380	Os_chr10	18260454	[C/T]	REGULATORY
OsMed35_1	LOC_Os10g34380	Os_chr10	18260380	[C/T]	REGULATORY
OsMed36_1	LOC_Os02g57590	Os_chr02	35266835	[G/A]	INTRON
OsMed36_1	LOC_Os02g57590	Os_chr02	35266697	[T/C]	INTRON
OsMed36_1	LOC_Os02g57590	Os_chr02	35266659	[A/T]	INTRON
OsMed36_1	LOC_Os02g57590	Os_chr02	35266428	[T/C]	INTRON
OsMed36_1	LOC_Os02g57590	Os_chr02	35266419	[G/A]	INTRON
OsMed36_1	LOC_Os02g57590	Os_chr02	35265926	[T/C]	INTRON
OsMed36_1	LOC_Os02g57590	Os_chr02	35265920	[C/T]	INTRON
OsMed36_1	LOC_Os02g57590	Os_chr02	35267671	[G/A]	INTRON
OsMed36_1	LOC_Os02g57590	Os_chr02	35267619	[T/A]	INTRON
OsMed36_1	LOC_Os02g57590	Os_chr02	35267531	[A/G]	INTRON
OsMed36_1	LOC_Os02g57590	Os_chr02	35267487	[T/G]	INTRON
OsMed36_1	LOC_Os02g57590	Os_chr02	35267212	[C/T]	INTRON
OsMed36_1	LOC_Os02g57590	Os_chr02	35265782	[G/T]	REGULATORY
OsMed36_1	LOC_Os02g57590	Os_chr02	35267880	[G/A]	REGULATORY
OsMed36_1	LOC_Os02g57590	Os_chr02	35268423	[A/G]	REGULATORY
OsMed36_1	LOC_Os02g57590	Os_chr02	35265693	[C/T]	REGULATORY
OsMed36_1	LOC_Os02g57590	Os_chr02	35265626	[G/A]	REGULATORY
OsMed36_1	LOC_Os02g57590	Os_chr02	35265594	[C/A]	REGULATORY
OsMed36_1	LOC_Os02g57590	Os_chr02	35265593	[G/A]	REGULATORY
OsMed36_1	LOC_Os02g57590	Os_chr02	35265581	[T/C]	REGULATORY
OsMed36_1	LOC_Os02g57590	Os_chr02	35265573	[C/G]	REGULATORY
OsMed36_1	LOC_Os02g57590	Os_chr02	35265539	[T/C]	REGULATORY
OsMed36_1	LOC_Os02g57590	Os_chr02	35265495	[G/T]	REGULATORY
OsMed36_1	LOC_Os02g57590	Os_chr02	35265446	[T/C]	REGULATORY
OsMed36_1	LOC_Os02g57590	Os_chr02	35265427	[C/T]	REGULATORY
OsMed36_1	LOC_Os02g57590	Os_chr02	35265339	[G/A]	REGULATORY
OsMed36_1	LOC_Os02g57590	Os_chr02	35265336	[A/T]	REGULATORY
OsMed36_1	LOC_Os02g57590	Os_chr02	35265270	[C/T]	REGULATORY
OsMed36_1	LOC_Os02g57590	Os_chr02	35265269	[A/C]	REGULATORY
OsMed36_1	LOC_Os02g57590	Os_chr02	35265268	[C/T]	REGULATORY

**Mediator genes**	**MSU locus ID**	**Chromosomes**	**Physical Position (bp)**	**SNPs**	**Structural Annotation**
OsMed36_1	LOC_Os02g57590	Os_chr02	35265258	[C/T]	REGULATORY
OsMed36_1	LOC_Os02g57590	Os_chr02	35265247	[G/A]	REGULATORY
OsMed36_1	LOC_Os02g57590	Os_chr02	35265227	[G/A]	REGULATORY
OsMed36_1	LOC_Os02g57590	Os_chr02	35265206	[T/A]	REGULATORY
OsMed36_1	LOC_Os02g57590	Os_chr02	35265204	[C/T]	REGULATORY
OsMed36_1	LOC_Os02g57590	Os_chr02	35265199	[G/A]	REGULATORY
OsMed36_1	LOC_Os02g57590	Os_chr02	35265187	[A/G]	REGULATORY
OsMed36_1	LOC_Os02g57590	Os_chr02	35265162	[G/A]	REGULATORY
OsMed36_1	LOC_Os02g57590	Os_chr02	35265158	[T/A]	REGULATORY
OsMed36_1	LOC_Os02g57590	Os_chr02	35265145	[C/G]	REGULATORY
OsMed36_1	LOC_Os02g57590	Os_chr02	35265143	[C/T]	REGULATORY
OsMed36_1	LOC_Os02g57590	Os_chr02	35265131	[G/T]	REGULATORY
OsMed36_1	LOC_Os02g57590	Os_chr02	35265128	[G/A]	REGULATORY
OsMed36_1	LOC_Os02g57590	Os_chr02	35265122	[G/A]	REGULATORY
OsMed36_1	LOC_Os02g57590	Os_chr02	35265120	[G/A]	REGULATORY
OsMed36_1	LOC_Os02g57590	Os_chr02	35265115	[C/T]	REGULATORY
OsMed36_1	LOC_Os02g57590	Os_chr02	35265110	[T/C]	REGULATORY
OsMed36_1	LOC_Os02g57590	Os_chr02	35265107	[C/G]	REGULATORY
OsMed36_1	LOC_Os02g57590	Os_chr02	35265094	[G/A]	REGULATORY
OsMed36_1	LOC_Os02g57590	Os_chr02	35265074	[G/C]	REGULATORY
OsMed36_1	LOC_Os02g57590	Os_chr02	35265063	[G/A]	REGULATORY
OsMed36_1	LOC_Os02g57590	Os_chr02	35265057	[C/T]	REGULATORY
OsMed36_1	LOC_Os02g57590	Os_chr02	35265046	[G/A]	REGULATORY
OsMed36_1	LOC_Os02g57590	Os_chr02	35265039	[T/C]	REGULATORY
OsMed36_1	LOC_Os02g57590	Os_chr02	35265036	[G/A]	REGULATORY
OsMed36_1	LOC_Os02g57590	Os_chr02	35265030	[C/T]	REGULATORY
OsMed36_1	LOC_Os02g57590	Os_chr02	35265005	[C/T]	REGULATORY
OsMed36_1	LOC_Os02g57590	Os_chr02	35265004	[G/A]	REGULATORY
OsMed36_1	LOC_Os02g57590	Os_chr02	35264981	[G/A]	REGULATORY
OsMed36_1	LOC_Os02g57590	Os_chr02	35264978	[A/G]	REGULATORY
OsMed36_1	LOC_Os02g57590	Os_chr02	35264967	[A/C]	REGULATORY
OsMed36_1	LOC_Os02g57590	Os_chr02	35264959	[T/A]	REGULATORY
OsMed36_1	LOC_Os02g57590	Os_chr02	35264955	[A/G]	REGULATORY
OsMed36_1	LOC_Os02g57590	Os_chr02	35264950	[C/T]	REGULATORY
OsMed36_1	LOC_Os02g57590	Os_chr02	35264939	[C/T]	REGULATORY
OsMed36_1	LOC_Os02g57590	Os_chr02	35264835	[C/A]	REGULATORY
OsMed36_1	LOC_Os02g57590	Os_chr02	35264821	[A/G]	REGULATORY
OsMed36_1	LOC_Os02g57590	Os_chr02	35264721	[C/T]	REGULATORY
OsMed36_1	LOC_Os02g57590	Os_chr02	35266731	[C/T]	SYNONYMOUS-CODING
OsMed36_1	LOC_Os02g57590	Os_chr02	35266723	[C/T]	SYNONYMOUS-CODING
OsMed36_1	LOC_Os02g57590	Os_chr02	35266541	[C/T]	SYNONYMOUS-CODING
OsMed36_1	LOC_Os02g57590	Os_chr02	35266234	[C/T]	SYNONYMOUS-CODING
OsMed36_1	LOC_Os02g57590	Os_chr02	35266069	[T/C]	SYNONYMOUS-CODING
OsMed36_2	LOC_Os05g08360	Os_chr05	4561760	[T/A]	INTRON
OsMed36_2	LOC_Os05g08360	Os_chr05	4561676	[T/G]	INTRON
OsMed36_2	LOC_Os05g08360	Os_chr05	4561665	[A/C]	INTRON
OsMed36_2	LOC_Os05g08360	Os_chr05	4561648	[T/C]	INTRON

Mediator genes	MSU locus ID	Chromosomes	Physical Position (bp)	SNPs	Structural Annotation
OsMed36_2	LOC_Os05g08360	Os_chr05	4561344	[A/C]	INTRON
OsMed36_2	LOC_Os05g08360	Os_chr05	4561321	[G/T]	INTRON
OsMed36_2	LOC_Os05g08360	Os_chr05	4561247	[G/A]	INTRON
OsMed36_2	LOC_Os05g08360	Os_chr05	4561195	[A/G]	INTRON
OsMed36_2	LOC_Os05g08360	Os_chr05	4560994	[T/G]	INTRON
OsMed36_2	LOC_Os05g08360	Os_chr05	4560953	[G/T]	INTRON
OsMed36_2	LOC_Os05g08360	Os_chr05	4560939	[G/T]	INTRON
OsMed36_2	LOC_Os05g08360	Os_chr05	4560901	[T/C]	INTRON
OsMed36_2	LOC_Os05g08360	Os_chr05	4560852	[G/T]	INTRON
OsMed36_2	LOC_Os05g08360	Os_chr05	4560793	[G/A]	INTRON
OsMed36_2	LOC_Os05g08360	Os_chr05	4560393	[T/C]	INTRON
OsMed36_2	LOC_Os05g08360	Os_chr05	4560373	[T/A]	INTRON
OsMed36_2	LOC_Os05g08360	Os_chr05	4560344	[T/A]	INTRON
OsMed36_2	LOC_Os05g08360	Os_chr05	4560235	[T/G]	INTRON
OsMed36_2	LOC_Os05g08360	Os_chr05	4559964	[T/C]	INTRON
OsMed36_2	LOC_Os05g08360	Os_chr05	4559887	[A/G]	INTRON
OsMed36_2	LOC_Os05g08360	Os_chr05	4559868	[A/G]	INTRON
OsMed36_2	LOC_Os05g08360	Os_chr05	4559728	[A/C]	INTRON
OsMed36_2	LOC_Os05g08360	Os_chr05	4559287	[T/C]	REGULATORY
OsMed36_2	LOC_Os05g08360	Os_chr05	4559260	[G/C]	REGULATORY
OsMed36_2	LOC_Os05g08360	Os_chr05	4561992	[A/T]	REGULATORY
OsMed36_2	LOC_Os05g08360	Os_chr05	4562303	[A/G]	REGULATORY
OsMed36_2	LOC_Os05g08360	Os_chr05	4562263	[C/T]	REGULATORY
OsMed36_2	LOC_Os05g08360	Os_chr05	4559199	[C/T]	REGULATORY
OsMed36_2	LOC_Os05g08360	Os_chr05	4559088	[T/C]	REGULATORY
OsMed36_2	LOC_Os05g08360	Os_chr05	4558995	[G/A]	REGULATORY
OsMed36_2	LOC_Os05g08360	Os_chr05	4558971	[A/T]	REGULATORY
OsMed36_2	LOC_Os05g08360	Os_chr05	4558943	[G/A]	REGULATORY
OsMed36_2	LOC_Os05g08360	Os_chr05	4558927	[T/C]	REGULATORY
OsMed36_2	LOC_Os05g08360	Os_chr05	4558886	[T/C]	REGULATORY
OsMed36_2	LOC_Os05g08360	Os_chr05	4558885	[A/G]	REGULATORY
OsMed36_2	LOC_Os05g08360	Os_chr05	4558856	[G/A]	REGULATORY
OsMed36_2	LOC_Os05g08360	Os_chr05	4558837	[G/A]	REGULATORY
OsMed36_2	LOC_Os05g08360	Os_chr05	4558817	[C/A]	REGULATORY
OsMed36_2	LOC_Os05g08360	Os_chr05	4558810	[C/T]	REGULATORY
OsMed36_2	LOC_Os05g08360	Os_chr05	4558796	[C/T]	REGULATORY
OsMed36_2	LOC_Os05g08360	Os_chr05	4558788	[G/C]	REGULATORY
OsMed36_2	LOC_Os05g08360	Os_chr05	4558762	[C/T]	REGULATORY
OsMed36_2	LOC_Os05g08360	Os_chr05	4558739	[T/C]	REGULATORY
OsMed36_2	LOC_Os05g08360	Os_chr05	4558720	[T/C]	REGULATORY
OsMed36_2	LOC_Os05g08360	Os_chr05	4558712	[C/T]	REGULATORY
OsMed36_2	LOC_Os05g08360	Os_chr05	4558698	[G/A]	REGULATORY
OsMed36_2	LOC_Os05g08360	Os_chr05	4558693	[T/C]	REGULATORY
OsMed36_2	LOC_Os05g08360	Os_chr05	4558636	[C/T]	REGULATORY
OsMed36_2	LOC_Os05g08360	Os_chr05	4558627	[G/A]	REGULATORY
OsMed36_2	LOC_Os05g08360	Os_chr05	4558621	[G/A]	REGULATORY
OsMed36_2	LOC_Os05g08360	Os_chr05	4558608	[G/A]	REGULATORY

**Mediator genes**	**MSU locus ID**	**Chromosomes**	**Physical Position (bp)**	**SNPs**	**Structural Annotation**
OsMed36_2	LOC_Os05g08360	Os_chr05	4558538	[G/A]	REGULATORY
OsMed36_2	LOC_Os05g08360	Os_chr05	4558532	[G/A]	REGULATORY
OsMed36_2	LOC_Os05g08360	Os_chr05	4558451	[G/A]	REGULATORY
OsMed36_2	LOC_Os05g08360	Os_chr05	4558414	[G/A]	REGULATORY
OsMed36_2	LOC_Os05g08360	Os_chr05	4558389	[T/C]	REGULATORY
OsMed36_2	LOC_Os05g08360	Os_chr05	4558380	[G/A]	REGULATORY
OsMed36_2	LOC_Os05g08360	Os_chr05	4558378	[T/C]	REGULATORY
OsMed36_2	LOC_Os05g08360	Os_chr05	4558355	[C/T]	REGULATORY
OsMed36_2	LOC_Os05g08360	Os_chr05	4558354	[T/C]	REGULATORY
OsMed36_2	LOC_Os05g08360	Os_chr05	4558352	[C/T]	REGULATORY
OsMed36_2	LOC_Os05g08360	Os_chr05	4558298	[C/T]	REGULATORY
OsMed36_2	LOC_Os05g08360	Os_chr05	4560528	[G/A]	SYNONYMOUS-CODING
OsMed36_2	LOC_Os05g08360	Os_chr05	4560312	[T/G]	SYNONYMOUS-CODING
OsMed36_2	LOC_Os05g08360	Os_chr05	4560270	[G/A]	SYNONYMOUS-CODING
OsMed36_2	LOC_Os05g08360	Os_chr05	4560074	[C/T]	SYNONYMOUS-CODING
OsMed37_1	LOC_Os01g62280	Os_chr01	36038114	[C/T]	INTRON
OsMed37_1	LOC_Os01g62280	Os_chr01	36038091	[G/A]	INTRON
OsMed37_1	LOC_Os01g62280	Os_chr01	36038072	[C/A]	INTRON
OsMed37_1	LOC_Os01g62280	Os_chr01	36038066	[G/A]	INTRON
OsMed37_1	LOC_Os01g62280	Os_chr01	36038048	[C/T]	INTRON
OsMed37_1	LOC_Os01g62280	Os_chr01	36038004	[G/A]	INTRON
OsMed37_1	LOC_Os01g62290	Os_chr01	36039867	[T/C]	INTRON
OsMed37_1	LOC_Os01g62290	Os_chr01	36039569	[A/G]	INTRON
OsMed37_1	LOC_Os01g62290	Os_chr01	36039405	[G/A]	INTRON
OsMed37_1	LOC_Os01g62290	Os_chr01	36039279	[G/A]	INTRON
OsMed37_1	LOC_Os01g62290	Os_chr01	36039247	[G/T]	INTRON
OsMed37_1	LOC_Os01g62290	Os_chr01	36039153	[A/C]	INTRON
OsMed37_1	LOC_Os01g62290	Os_chr01	36039103	[G/T]	INTRON
OsMed37_1	LOC_Os01g62280	Os_chr01	36037985	[G/T]	NON-SYNONYMOUS-CODING
OsMed37_1	LOC_Os01g62280	Os_chr01	36037862	[G/A]	NON-SYNONYMOUS-CODING
OsMed37_1	LOC_Os01g62280	Os_chr01	36037826	[C/T]	NON-SYNONYMOUS-CODING
OsMed37_1	LOC_Os01g62290	Os_chr01	36040723	[A/T]	NON-SYNONYMOUS-CODING
OsMed37_1	LOC_Os01g62290	Os_chr01	36038769	[T/C]	REGULATORY
OsMed37_1	LOC_Os01g62290	Os_chr01	36038663	[T/A]	REGULATORY
OsMed37_1	LOC_Os01g62290	Os_chr01	36042352	[G/A]	REGULATORY
OsMed37_1	LOC_Os01g62290	Os_chr01	36042255	[C/G]	REGULATORY
OsMed37_1	LOC_Os01g62290	Os_chr01	36042211	[C/A]	REGULATORY
OsMed37_1	LOC_Os01g62290	Os_chr01	36042047	[G/T]	REGULATORY
OsMed37_1	LOC_Os01g62290	Os_chr01	36041974	[G/C]	REGULATORY
OsMed37_1	LOC_Os01g62290	Os_chr01	36038534	[C/A]	REGULATORY
OsMed37_1	LOC_Os01g62290	Os_chr01	36038518	[T/G]	REGULATORY
OsMed37_1	LOC_Os01g62290	Os_chr01	36038266	[C/T]	REGULATORY
OsMed37_1	LOC_Os01g62290	Os_chr01	36038199	[T/G]	REGULATORY
OsMed37_1	LOC_Os01g62290	Os_chr01	36037714	[C/T]	REGULATORY
OsMed37_1	LOC_Os01g62280	Os_chr01	36037926	[C/A]	SYNONYMOUS-CODING
OsMed37_1	LOC_Os01g62290	Os_chr01	36041837	[C/T]	SYNONYMOUS-CODING
OsMed37_1	LOC_Os01g62290	Os_chr01	36041504	[C/T]	SYNONYMOUS-CODING

**Mediator genes**	**MSU locus ID**	**Chromosomes**	**Physical Position (bp)**	**SNPs**	**Structural Annotation**
OsMed37_1	LOC_Os01g62290	Os_chr01	36041477	[C/T]	SYNONYMOUS-CODING
OsMed37_1	LOC_Os01g62290	Os_chr01	36041429	[C/T]	SYNONYMOUS-CODING
OsMed37_1	LOC_Os01g62290	Os_chr01	36041276	[A/G]	SYNONYMOUS-CODING
OsMed37_1	LOC_Os01g62290	Os_chr01	36041204	[T/C]	SYNONYMOUS-CODING
OsMed37_1	LOC_Os01g62290	Os_chr01	36040286	[C/T]	SYNONYMOUS-CODING
OsMed37_1	LOC_Os01g62290	Os_chr01	36040241	[C/T]	SYNONYMOUS-CODING
OsMed37_2	LOC_Os02g02410	Os_chr02	842015	[G/T]	INTRON
OsMed37_2	LOC_Os02g02410	Os_chr02	841964	[T/A]	INTRON
OsMed37_2	LOC_Os02g02410	Os_chr02	841923	[A/G]	INTRON
OsMed37_2	LOC_Os02g02410	Os_chr02	841464	[C/T]	INTRON
OsMed37_2	LOC_Os02g02410	Os_chr02	841147	[G/T]	INTRON
OsMed37_2	LOC_Os02g02410	Os_chr02	840824	[A/G]	INTRON
OsMed37_2	LOC_Os02g02410	Os_chr02	840821	[T/C]	INTRON
OsMed37_2	LOC_Os02g02410	Os_chr02	840690	[G/A]	INTRON
OsMed37_2	LOC_Os02g02410	Os_chr02	840624	[G/A]	INTRON
OsMed37_2	LOC_Os02g02410	Os_chr02	840074	[G/A]	INTRON
OsMed37_2	LOC_Os02g02410	Os_chr02	840073	[C/T]	INTRON
OsMed37_2	LOC_Os02g02410	Os_chr02	839987	[G/A]	INTRON
OsMed37_2	LOC_Os02g02410	Os_chr02	839986	[C/T]	INTRON
OsMed37_2	LOC_Os02g02410	Os_chr02	839802	[C/G]	INTRON
OsMed37_2	LOC_Os02g02410	Os_chr02	839625	[G/T]	INTRON
OsMed37_2	LOC_Os02g02410	Os_chr02	842651	[T/C]	REGULATORY
OsMed37_2	LOC_Os02g02410	Os_chr02	842605	[G/T]	REGULATORY
OsMed37_2	LOC_Os02g02410	Os_chr02	842568	[T/A]	REGULATORY
OsMed37_2	LOC_Os02g02410	Os_chr02	838958	[T/C]	REGULATORY
OsMed37_2	LOC_Os02g02410	Os_chr02	838819	[G/C]	REGULATORY
OsMed37_2	LOC_Os02g57590	Os_chr02	843665	[G/A]	REGULATORY
OsMed37_2	LOC_Os02g57590	Os_chr02	843647	[G/A]	REGULATORY
OsMed37_2	LOC_Os02g57590	Os_chr02	843617	[G/A]	REGULATORY
OsMed37_2	LOC_Os02g57590	Os_chr02	843604	[G/T]	REGULATORY
OsMed37_2	LOC_Os02g57590	Os_chr02	843533	[C/T]	REGULATORY
OsMed37_2	LOC_Os02g57590	Os_chr02	843526	[C/T]	REGULATORY
OsMed37_2	LOC_Os02g57590	Os_chr02	843517	[C/T]	REGULATORY
OsMed37_2	LOC_Os02g57590	Os_chr02	843495	[A/G]	REGULATORY
OsMed37_2	LOC_Os02g57590	Os_chr02	843489	[C/A]	REGULATORY
OsMed37_2	LOC_Os02g57590	Os_chr02	843483	[G/T]	REGULATORY
OsMed37_2	LOC_Os02g57590	Os_chr02	843430	[C/T]	REGULATORY
OsMed37_2	LOC_Os02g57590	Os_chr02	843428	[G/A]	REGULATORY
OsMed37_2	LOC_Os02g57590	Os_chr02	843422	[G/A]	REGULATORY
OsMed37_2	LOC_Os02g57590	Os_chr02	843400	[G/A]	REGULATORY
OsMed37_2	LOC_Os02g57590	Os_chr02	843397	[C/T]	REGULATORY
OsMed37_2	LOC_Os02g57590	Os_chr02	843387	[T/G]	REGULATORY
OsMed37_2	LOC_Os02g57590	Os_chr02	843379	[C/A]	REGULATORY
OsMed37_2	LOC_Os02g57590	Os_chr02	843365	[G/C]	REGULATORY
OsMed37_2	LOC_Os02g57590	Os_chr02	843348	[C/T]	REGULATORY
OsMed37_2	LOC_Os02g57590	Os_chr02	843347	[G/A]	REGULATORY
OsMed37_2	LOC_Os02g57590	Os_chr02	843339	[G/A]	REGULATORY

**Mediator genes**	**MSU locus ID**	**Chromosomes**	**Physical Position (bp)**	**SNPs**	**Structural Annotation**
OsMed37_2	LOC_Os02g57590	Os_chr02	843330	[G/A]	REGULATORY
OsMed37_2	LOC_Os02g57590	Os_chr02	843289	[G/A]	REGULATORY
OsMed37_2	LOC_Os02g57590	Os_chr02	843248	[G/A]	REGULATORY
OsMed37_2	LOC_Os02g57590	Os_chr02	843238	[C/T]	REGULATORY
OsMed37_2	LOC_Os02g57590	Os_chr02	843170	[T/A]	REGULATORY
OsMed37_2	LOC_Os02g57590	Os_chr02	843159	[A/T]	REGULATORY
OsMed37_2	LOC_Os02g57590	Os_chr02	843148	[T/A]	REGULATORY
OsMed37_2	LOC_Os02g57590	Os_chr02	843106	[G/A]	REGULATORY
OsMed37_2	LOC_Os02g57590	Os_chr02	843052	[G/T]	REGULATORY
OsMed37_2	LOC_Os02g57590	Os_chr02	843021	[G/A]	REGULATORY
OsMed37_2	LOC_Os02g57590	Os_chr02	843016	[A/T]	REGULATORY
OsMed37_2	LOC_Os02g57590	Os_chr02	843005	[T/C]	REGULATORY
OsMed37_2	LOC_Os02g57590	Os_chr02	842919	[G/A]	REGULATORY
OsMed37_2	LOC_Os02g02410	Os_chr02	842490	[G/A]	SYNONYMOUS-CODING
OsMed37_2	LOC_Os02g02410	Os_chr02	841731	[C/T]	SYNONYMOUS-CODING
OsMed37_2	LOC_Os02g02410	Os_chr02	841334	[C/T]	SYNONYMOUS-CODING
OsMed37_2	LOC_Os02g02410	Os_chr02	840991	[G/A]	SYNONYMOUS-CODING
OsMed37_2	LOC_Os02g02410	Os_chr02	840895	[C/A]	SYNONYMOUS-CODING
OsMed37_2	LOC_Os02g02410	Os_chr02	840472	[A/T]	SYNONYMOUS-CODING
OsMed37_2	LOC_Os02g02410	Os_chr02	840421	[G/A]	SYNONYMOUS-CODING
OsMed37_2	LOC_Os02g02410	Os_chr02	840250	[G/A]	SYNONYMOUS-CODING
OsMed37_2	LOC_Os02g02410	Os_chr02	840103	[A/G]	SYNONYMOUS-CODING
OsMed37_2	LOC_Os02g02410	Os_chr02	839661	[G/T]	SYNONYMOUS-CODING
OsMed37_2	LOC_Os02g02410	Os_chr02	839465	[C/T]	SYNONYMOUS-CODING
OsMed37_2	LOC_Os02g02410	Os_chr02	839096	[G/A]	SYNONYMOUS-CODING
OsMed37_3	LOC_Os03g16860	Os_chr03	9369665	[A/G]	INTRON
OsMed37_3	LOC_Os03g16860	Os_chr03	9369489	[A/T]	INTRON
OsMed37_3	LOC_Os03g16860	Os_chr03	9369204	[A/G]	REGULATORY
OsMed37_3	LOC_Os03g16860	Os_chr03	9369147	[C/G]	REGULATORY
OsMed37_3	LOC_Os03g16860	Os_chr03	9369146	[C/A]	REGULATORY
OsMed37_3	LOC_Os03g16860	Os_chr03	9369145	[T/G]	REGULATORY
OsMed37_3	LOC_Os03g16860	Os_chr03	9369123	[T/C]	REGULATORY
OsMed37_3	LOC_Os03g16860	Os_chr03	9369122	[A/C]	REGULATORY
OsMed37_3	LOC_Os03g16860	Os_chr03	9371937	[T/A]	REGULATORY
OsMed37_3	LOC_Os03g16860	Os_chr03	9368882	[C/T]	REGULATORY
OsMed37_3	LOC_Os03g16860	Os_chr03	9368706	[T/C]	REGULATORY
OsMed37_3	LOC_Os03g16860	Os_chr03	9368648	[A/G]	REGULATORY
OsMed37_3	LOC_Os03g16860	Os_chr03	9368619	[C/T]	REGULATORY
OsMed37_3	LOC_Os03g16860	Os_chr03	9368599	[C/T]	REGULATORY
OsMed37_3	LOC_Os03g16860	Os_chr03	9368595	[A/G]	REGULATORY
OsMed37_3	LOC_Os03g16860	Os_chr03	9368535	[T/C]	REGULATORY
OsMed37_3	LOC_Os03g16860	Os_chr03	9368425	[A/T]	REGULATORY
OsMed37_3	LOC_Os03g16860	Os_chr03	9368399	[A/G]	REGULATORY
OsMed37_3	LOC_Os03g16860	Os_chr03	9368381	[C/T]	REGULATORY
OsMed37_3	LOC_Os03g16860	Os_chr03	9368337	[G/A]	REGULATORY
OsMed37_3	LOC_Os03g16860	Os_chr03	9368305	[T/C]	REGULATORY
OsMed37_3	LOC_Os03g16860	Os_chr03	9368293	[G/A]	REGULATORY

Mediator genes	MSU locus ID	Chromosomes	Physical Position (bp)	SNPs	Structural Annotation
OsMed37_3	LOC_Os03g16860	Os_chr03	9368163	[C/A]	REGULATORY
OsMed37_3	LOC_Os03g16860	Os_chr03	9368146	[T/C]	REGULATORY
OsMed37_3	LOC_Os03g16860	Os_chr03	9371773	[C/T]	SYNONYMOUS-CODING
OsMed37_3	LOC_Os03g16860	Os_chr03	9371083	[T/G]	SYNONYMOUS-CODING
OsMed37_3	LOC_Os03g16860	Os_chr03	9370828	[G/A]	SYNONYMOUS-CODING
OsMed37_3	LOC_Os03g16860	Os_chr03	9370615	[A/G]	SYNONYMOUS-CODING
OsMed37_3	LOC_Os03g16860	Os_chr03	9370408	[C/G]	SYNONYMOUS-CODING
OsMed37_3	LOC_Os03g16860	Os_chr03	9370204	[A/C]	SYNONYMOUS-CODING
OsMed37_4	LOC_Os03g60620	Os_chr03	34441521	[A/G]	INTRON
OsMed37_4	LOC_Os03g60620	Os_chr03	34441505	[A/G]	INTRON
OsMed37_4	LOC_Os03g60620	Os_chr03	34441453	[G/C]	INTRON
OsMed37_4	LOC_Os03g60620	Os_chr03	34441451	[G/A]	INTRON
OsMed37_4	LOC_Os03g60620	Os_chr03	34441422	[G/A]	INTRON
OsMed37_4	LOC_Os03g60620	Os_chr03	34441421	[C/T]	INTRON
OsMed37_4	LOC_Os03g60620	Os_chr03	34441417	[C/T]	INTRON
OsMed37_4	LOC_Os03g60620	Os_chr03	34441268	[G/A]	INTRON
OsMed37_4	LOC_Os03g60620	Os_chr03	34441138	[G/T]	INTRON
OsMed37_4	LOC_Os03g60620	Os_chr03	34441112	[A/G]	INTRON
OsMed37_4	LOC_Os03g60620	Os_chr03	34441070	[G/T]	INTRON
OsMed37_4	LOC_Os03g60620	Os_chr03	34441043	[A/G]	INTRON
OsMed37_4	LOC_Os03g60620	Os_chr03	34440881	[C/T]	INTRON
OsMed37_4	LOC_Os03g60620	Os_chr03	34440873	[T/C]	INTRON
OsMed37_4	LOC_Os03g60620	Os_chr03	34440869	[G/A]	INTRON
OsMed37_4	LOC_Os03g60620	Os_chr03	34440855	[G/A]	INTRON
OsMed37_4	LOC_Os03g60620	Os_chr03	34440778	[G/C]	INTRON
OsMed37_4	LOC_Os03g60620	Os_chr03	34440765	[A/C]	INTRON
OsMed37_4	LOC_Os03g60620	Os_chr03	34440652	[G/T]	INTRON
OsMed37_4	LOC_Os03g60620	Os_chr03	34440613	[C/A]	INTRON
OsMed37_4	LOC_Os03g60620	Os_chr03	34440548	[G/T]	INTRON
OsMed37_4	LOC_Os03g60620	Os_chr03	34440544	[A/T]	INTRON
OsMed37_4	LOC_Os03g60620	Os_chr03	34440523	[G/T]	INTRON
OsMed37_4	LOC_Os03g60620	Os_chr03	34440422	[T/C]	INTRON
OsMed37_4	LOC_Os03g60620	Os_chr03	34440392	[G/A]	INTRON
OsMed37_4	LOC_Os03g60620	Os_chr03	34440313	[A/T]	INTRON
OsMed37_4	LOC_Os03g60620	Os_chr03	34440162	[T/A]	INTRON
OsMed37_4	LOC_Os03g60620	Os_chr03	34440124	[C/T]	INTRON
OsMed37_4	LOC_Os03g60620	Os_chr03	34439900	[C/A]	INTRON
OsMed37_4	LOC_Os03g60620	Os_chr03	34439894	[T/C]	INTRON
OsMed37_4	LOC_Os03g60620	Os_chr03	34439814	[T/C]	INTRON
OsMed37_4	LOC_Os03g60620	Os_chr03	34439805	[G/C]	INTRON
OsMed37_4	LOC_Os03g60620	Os_chr03	34439784	[G/A]	INTRON
OsMed37_4	LOC_Os03g60620	Os_chr03	34439764	[C/T]	INTRON
OsMed37_4	LOC_Os03g60620	Os_chr03	34439710	[A/G]	INTRON
OsMed37_4	LOC_Os03g60620	Os_chr03	34439696	[G/A]	INTRON
OsMed37_4	LOC_Os03g60620	Os_chr03	34439688	[C/T]	INTRON
OsMed37_4	LOC_Os03g60620	Os_chr03	34439678	[T/G]	INTRON
OsMed37_4	LOC_Os03g60620	Os_chr03	34439673	[A/G]	INTRON

**Mediator genes**	**MSU locus ID**	**Chromosomes**	**Physical Position (bp)**	**SNPs**	**Structural Annotation**
OsMed37_4	LOC_Os03g60620	Os_chr03	34439593	[T/C]	INTRON
OsMed37_4	LOC_Os03g60620	Os_chr03	34439590	[T/C]	INTRON
OsMed37_4	LOC_Os03g60620	Os_chr03	34443099	[C/G]	NON-SYNONYMOUS-CODING
OsMed37_4	LOC_Os03g60620	Os_chr03	34439280	[T/C]	REGULATORY
OsMed37_4	LOC_Os03g60620	Os_chr03	34439263	[T/G]	REGULATORY
OsMed37_4	LOC_Os03g60620	Os_chr03	34443712	[G/A]	REGULATORY
OsMed37_4	LOC_Os03g60620	Os_chr03	34443585	[T/A]	REGULATORY
OsMed37_4	LOC_Os03g60620	Os_chr03	34443521	[G/T]	REGULATORY
OsMed37_4	LOC_Os03g60620	Os_chr03	34443360	[G/A]	REGULATORY
OsMed37_4	LOC_Os03g60620	Os_chr03	34439026	[T/A]	REGULATORY
OsMed37_4	LOC_Os03g60620	Os_chr03	34438949	[T/C]	REGULATORY
OsMed37_4	LOC_Os03g60620	Os_chr03	34438932	[C/A]	REGULATORY
OsMed37_4	LOC_Os03g60620	Os_chr03	34438899	[T/A]	REGULATORY
OsMed37_4	LOC_Os03g60620	Os_chr03	34438819	[A/G]	REGULATORY
OsMed37_4	LOC_Os03g60620	Os_chr03	34438779	[C/T]	REGULATORY
OsMed37_4	LOC_Os03g60620	Os_chr03	34438758	[C/T]	REGULATORY
OsMed37_4	LOC_Os03g60620	Os_chr03	34438754	[G/T]	REGULATORY
OsMed37_4	LOC_Os03g60620	Os_chr03	34438744	[C/T]	REGULATORY
OsMed37_4	LOC_Os03g60620	Os_chr03	34438740	[A/G]	REGULATORY
OsMed37_4	LOC_Os03g60620	Os_chr03	34438681	[T/C]	REGULATORY
OsMed37_4	LOC_Os03g60620	Os_chr03	34438670	[T/A]	REGULATORY
OsMed37_4	LOC_Os03g60620	Os_chr03	34438642	[A/T]	REGULATORY
OsMed37_4	LOC_Os03g60620	Os_chr03	34438639	[G/T]	REGULATORY
OsMed37_4	LOC_Os03g60620	Os_chr03	34438606	[A/C]	REGULATORY
OsMed37_4	LOC_Os03g60620	Os_chr03	34438587	[T/C]	REGULATORY
OsMed37_4	LOC_Os03g60620	Os_chr03	34438574	[C/T]	REGULATORY
OsMed37_4	LOC_Os03g60620	Os_chr03	34438570	[A/G]	REGULATORY
OsMed37_4	LOC_Os03g60620	Os_chr03	34438552	[C/A]	REGULATORY
OsMed37_4	LOC_Os03g60620	Os_chr03	34438465	[A/G]	REGULATORY
OsMed37_4	LOC_Os03g60620	Os_chr03	34438445	[C/A]	REGULATORY
OsMed37_4	LOC_Os03g60620	Os_chr03	34438383	[C/T]	REGULATORY
OsMed37_4	LOC_Os03g60620	Os_chr03	34438366	[A/G]	REGULATORY
OsMed37_4	LOC_Os03g60620	Os_chr03	34438276	[C/T]	REGULATORY
OsMed37_4	LOC_Os03g60620	Os_chr03	34438263	[T/C]	REGULATORY
OsMed37_4	LOC_Os03g60620	Os_chr03	34438254	[C/T]	REGULATORY
OsMed37_4	LOC_Os03g60620	Os_chr03	34438242	[G/A]	REGULATORY
OsMed37_4	LOC_Os03g60620	Os_chr03	34438238	[C/T]	REGULATORY
OsMed37_4	LOC_Os03g60620	Os_chr03	34438224	[A/G]	REGULATORY
OsMed37_4	LOC_Os03g60620	Os_chr03	34438214	[G/A]	REGULATORY
OsMed37_4	LOC_Os03g60620	Os_chr03	34438211	[C/T]	REGULATORY
OsMed37_4	LOC_Os03g60620	Os_chr03	34438202	[T/C]	REGULATORY
OsMed37_4	LOC_Os03g60620	Os_chr03	34443318	[T/C]	SYNONYMOUS-CODING
OsMed37_4	LOC_Os03g60620	Os_chr03	34443273	[C/T]	SYNONYMOUS-CODING
OsMed37_4	LOC_Os03g60620	Os_chr03	34443222	[T/C]	SYNONYMOUS-CODING
OsMed37_4	LOC_Os03g60620	Os_chr03	34443165	[C/T]	SYNONYMOUS-CODING
OsMed37_4	LOC_Os03g60620	Os_chr03	34443123	[G/T]	SYNONYMOUS-CODING
OsMed37_4	LOC_Os03g60620	Os_chr03	34442886	[A/C]	SYNONYMOUS-CODING

**Mediator genes**	**MSU locus ID**	**Chromosomes**	**Physical Position (bp)**	**SNPs**	**Structural Annotation**
OsMed37_4	LOC_Os03g60620	Os_chr03	34442847	[T/C]	SYNONYMOUS-CODING
OsMed37_4	LOC_Os03g60620	Os_chr03	34442469	[T/C]	SYNONYMOUS-CODING
OsMed37_4	LOC_Os03g60620	Os_chr03	34442460	[T/C]	SYNONYMOUS-CODING
OsMed37_4	LOC_Os03g60620	Os_chr03	34442436	[T/C]	SYNONYMOUS-CODING
OsMed37_4	LOC_Os03g60620	Os_chr03	34442292	[C/T]	SYNONYMOUS-CODING
OsMed37_4	LOC_Os03g60620	Os_chr03	34442166	[C/T]	SYNONYMOUS-CODING
OsMed37_4	LOC_Os03g60620	Os_chr03	34442037	[C/A]	SYNONYMOUS-CODING
OsMed37_4	LOC_Os03g60620	Os_chr03	34441881	[A/C]	SYNONYMOUS-CODING
OsMed37_4	LOC_Os03g60620	Os_chr03	34441842	[A/T]	SYNONYMOUS-CODING
OsMed37_4	LOC_Os03g60620	Os_chr03	34441806	[G/A]	SYNONYMOUS-CODING
OsMed37_4	LOC_Os03g60620	Os_chr03	34441650	[C/T]	SYNONYMOUS-CODING
OsMed37_4	LOC_Os03g60620	Os_chr03	34441617	[T/C]	SYNONYMOUS-CODING
OsMed37_4	LOC_Os03g60620	Os_chr03	34439451	[C/G]	SYNONYMOUS-CODING
OsMed37_4	LOC_Os03g60620	Os_chr03	34439391	[G/C]	SYNONYMOUS-CODING
OsMed37_5	LOC_Os05g35400	Os_chr05	20973353	[C/G]	NON-SYNONYMOUS-CODING
OsMed37_5	LOC_Os05g35400	Os_chr05	20972744	[C/T]	NON-SYNONYMOUS-CODING
OsMed37_5	LOC_Os05g35400	Os_chr05	20971988	[G/A]	REGULATORY
OsMed37_5	LOC_Os05g35400	Os_chr05	20974460	[G/T]	REGULATORY
OsMed37_5	LOC_Os05g35400	Os_chr05	20974347	[A/G]	REGULATORY
OsMed37_5	LOC_Os05g35400	Os_chr05	20974262	[C/T]	REGULATORY
OsMed37_5	LOC_Os05g35400	Os_chr05	20974185	[C/A]	REGULATORY
OsMed37_5	LOC_Os05g35400	Os_chr05	20974174	[A/T]	REGULATORY
OsMed37_5	LOC_Os05g35400	Os_chr05	20974149	[C/T]	REGULATORY
OsMed37_5	LOC_Os05g35400	Os_chr05	20974143	[T/C]	REGULATORY
OsMed37_5	LOC_Os05g35400	Os_chr05	20974090	[C/T]	REGULATORY
OsMed37_5	LOC_Os05g35400	Os_chr05	20974083	[A/G]	REGULATORY
OsMed37_5	LOC_Os05g35400	Os_chr05	20974076	[G/A]	REGULATORY
OsMed37_5	LOC_Os05g35400	Os_chr05	20974054	[T/C]	REGULATORY
OsMed37_5	LOC_Os05g35400	Os_chr05	20974028	[T/C]	REGULATORY
OsMed37_5	LOC_Os05g35400	Os_chr05	20974007	[T/C]	REGULATORY
OsMed37_5	LOC_Os05g35400	Os_chr05	20973991	[C/T]	REGULATORY
OsMed37_5	LOC_Os05g35400	Os_chr05	20973977	[G/T]	REGULATORY
OsMed37_5	LOC_Os05g35400	Os_chr05	20973963	[C/T]	REGULATORY
OsMed37_5	LOC_Os05g35400	Os_chr05	20973958	[G/C]	REGULATORY
OsMed37_5	LOC_Os05g35400	Os_chr05	20973944	[C/A]	REGULATORY
OsMed37_5	LOC_Os05g35400	Os_chr05	20973903	[A/G]	REGULATORY
OsMed37_5	LOC_Os05g35400	Os_chr05	20973717	[C/A]	REGULATORY
OsMed37_5	LOC_Os05g35400	Os_chr05	20973692	[T/G]	REGULATORY
OsMed37_5	LOC_Os05g35400	Os_chr05	20973597	[C/A]	REGULATORY
OsMed37_5	LOC_Os05g35400	Os_chr05	20973565	[G/A]	REGULATORY
OsMed37_5	LOC_Os05g35400	Os_chr05	20973535	[C/T]	REGULATORY
OsMed37_5	LOC_Os05g35400	Os_chr05	20973426	[C/A]	SYNONYMOUS-CODING
OsMed37_5	LOC_Os05g35400	Os_chr05	20973285	[G/A]	SYNONYMOUS-CODING
OsMed37_5	LOC_Os05g35400	Os_chr05	20972535	[G/A]	SYNONYMOUS-CODING
OsMed37_5	LOC_Os05g35400	Os_chr05	20972169	[G/T]	SYNONYMOUS-CODING
OsMed37_5	LOC_Os05g35400	Os_chr05	20971851	[C/G]	SYNONYMOUS-CODING
OsMed37_5	LOC_Os05g35400	Os_chr05	20971803	[G/C]	SYNONYMOUS-CODING

**Mediator genes**	**MSU locus ID**	**Chromosomes**	**Physical Position (bp)**	**SNPs**	**Structural Annotation**
OsMed37_5	LOC_Os05g35400	Os_chr05	20971536	[A/G]	SYNONYMOUS-CODING
OsMed37_6	LOC_Os05g38530	Os_chr05	22539630	[G/T]	INTRON
OsMed37_6	LOC_Os05g38530	Os_chr05	22539484	[C/T]	INTRON
OsMed37_6	LOC_Os05g38530	Os_chr05	22539174	[A/T]	INTRON
OsMed37_6	LOC_Os05g38530	Os_chr05	22539141	[C/T]	INTRON
OsMed37_6	LOC_Os05g38530	Os_chr05	22539046	[T/C]	INTRON
OsMed37_6	LOC_Os05g38530	Os_chr05	22538986	[C/T]	INTRON
OsMed37_6	LOC_Os05g38530	Os_chr05	22539863	[C/A]	NON-SYNONYMOUS-CODING
OsMed37_6	LOC_Os05g38530	Os_chr05	22537346	[C/T]	NON-SYNONYMOUS-CODING
OsMed37_6	LOC_Os05g38530	Os_chr05	22539980	[T/A]	REGULATORY
OsMed37_6	LOC_Os05g38530	Os_chr05	22539883	[C/T]	REGULATORY
OsMed37_6	LOC_Os05g38530	Os_chr05	22539878	[C/T]	REGULATORY
OsMed37_6	LOC_Os05g38530	Os_chr05	22537029	[T/C]	REGULATORY
OsMed37_6	LOC_Os05g38530	Os_chr05	22537016	[C/T]	REGULATORY
OsMed37_6	LOC_Os05g38530	Os_chr05	22541021	[C/A]	REGULATORY
OsMed37_6	LOC_Os05g38530	Os_chr05	22541002	[G/A]	REGULATORY
OsMed37_6	LOC_Os05g38530	Os_chr05	22540980	[G/A]	REGULATORY
OsMed37_6	LOC_Os05g38530	Os_chr05	22540976	[G/T]	REGULATORY
OsMed37_6	LOC_Os05g38530	Os_chr05	22540966	[T/C]	REGULATORY
OsMed37_6	LOC_Os05g38530	Os_chr05	22540955	[C/T]	REGULATORY
OsMed37_6	LOC_Os05g38530	Os_chr05	22540937	[C/T]	REGULATORY
OsMed37_6	LOC_Os05g38530	Os_chr05	22540932	[G/A]	REGULATORY
OsMed37_6	LOC_Os05g38530	Os_chr05	22540910	[A/G]	REGULATORY
OsMed37_6	LOC_Os05g38530	Os_chr05	22540873	[C/T]	REGULATORY
OsMed37_6	LOC_Os05g38530	Os_chr05	22540818	[T/G]	REGULATORY
OsMed37_6	LOC_Os05g38530	Os_chr05	22540802	[T/C]	REGULATORY
OsMed37_6	LOC_Os05g38530	Os_chr05	22540779	[G/A]	REGULATORY
OsMed37_6	LOC_Os05g38530	Os_chr05	22540765	[G/C]	REGULATORY
OsMed37_6	LOC_Os05g38530	Os_chr05	22540726	[A/G]	REGULATORY
OsMed37_6	LOC_Os05g38530	Os_chr05	22540724	[G/A]	REGULATORY
OsMed37_6	LOC_Os05g38530	Os_chr05	22540698	[T/A]	REGULATORY
OsMed37_6	LOC_Os05g38530	Os_chr05	22540691	[C/T]	REGULATORY
OsMed37_6	LOC_Os05g38530	Os_chr05	22540659	[A/C]	REGULATORY
OsMed37_6	LOC_Os05g38530	Os_chr05	22540567	[G/T]	REGULATORY
OsMed37_6	LOC_Os05g38530	Os_chr05	22540565	[C/A]	REGULATORY
OsMed37_6	LOC_Os05g38530	Os_chr05	22540559	[T/C]	REGULATORY
OsMed37_6	LOC_Os05g38530	Os_chr05	22540555	[T/C]	REGULATORY
OsMed37_6	LOC_Os05g38530	Os_chr05	22540533	[G/A]	REGULATORY
OsMed37_6	LOC_Os05g38530	Os_chr05	22540502	[G/T]	REGULATORY
OsMed37_6	LOC_Os05g38530	Os_chr05	22540501	[G/A]	REGULATORY
OsMed37_6	LOC_Os05g38530	Os_chr05	22540482	[T/C]	REGULATORY
OsMed37_6	LOC_Os05g38530	Os_chr05	22540471	[C/A]	REGULATORY
OsMed37_6	LOC_Os05g38530	Os_chr05	22540470	[T/C]	REGULATORY
OsMed37_6	LOC_Os05g38530	Os_chr05	22540460	[C/T]	REGULATORY
OsMed37_6	LOC_Os05g38530	Os_chr05	22540445	[G/A]	REGULATORY
OsMed37_6	LOC_Os05g38530	Os_chr05	22540432	[A/G]	REGULATORY
OsMed37_6	LOC_Os05g38530	Os_chr05	22540418	[C/T]	REGULATORY

**Mediator genes**	**MSU locus ID**	**Chromosomes**	**Physical Position (bp)**	**SNPs**	**Structural Annotation**
OsMed37_6	LOC_Os05g38530	Os_chr05	22540403	[C/T]	REGULATORY
OsMed37_6	LOC_Os05g38530	Os_chr05	22540368	[A/G]	REGULATORY
OsMed37_6	LOC_Os05g38530	Os_chr05	22540070	[G/T]	REGULATORY
OsMed37_6	LOC_Os05g38530	Os_chr05	22540043	[T/G]	REGULATORY
OsMed37_6	LOC_Os05g38530	Os_chr05	22538434	[G/A]	SYNONYMOUS-CODING
OsMed37_6	LOC_Os05g38530	Os_chr05	22538008	[C/T]	SYNONYMOUS-CODING
OsMed37_6	LOC_Os05g38530	Os_chr05	22537276	[G/A]	SYNONYMOUS-CODING
OsMed37_7	LOC_Os08g09770	Os_chr08	5646727	[G/A]	NON-SYNONYMOUS-CODING
OsMed37_7	LOC_Os08g09770	Os_chr08	5646705	[G/A]	NON-SYNONYMOUS-CODING
OsMed37_7	LOC_Os08g09770	Os_chr08	5646329	[G/T]	NON-SYNONYMOUS-CODING
OsMed37_7	LOC_Os08g09770	Os_chr08	5645904	[G/C]	NON-SYNONYMOUS-CODING
OsMed37_7	LOC_Os08g09770	Os_chr08	5645824	[C/T]	NON-SYNONYMOUS-CODING
OsMed37_7	LOC_Os08g09770	Os_chr08	5645795	[C/G]	NON-SYNONYMOUS-CODING
OsMed37_7	LOC_Os08g09770	Os_chr08	5645701	[G/C]	NON-SYNONYMOUS-CODING
OsMed37_7	LOC_Os08g09770	Os_chr08	5645470	[C/T]	NON-SYNONYMOUS-CODING
OsMed37_7	LOC_Os08g09770	Os_chr08	5645443	[C/T]	NON-SYNONYMOUS-CODING
OsMed37_7	LOC_Os08g09770	Os_chr08	5645430	[G/A]	NON-SYNONYMOUS-CODING
OsMed37_7	LOC_Os08g09770	Os_chr08	5645394	[A/G]	NON-SYNONYMOUS-CODING
OsMed37_7	LOC_Os08g09770	Os_chr08	5645093	[C/G]	NON-SYNONYMOUS-CODING
OsMed37_7	LOC_Os08g09770	Os_chr08	5645072	[C/A]	NON-SYNONYMOUS-CODING
OsMed37_7	LOC_Os08g09770	Os_chr08	5645052	[C/T]	NON-SYNONYMOUS-CODING
OsMed37_7	LOC_Os08g09770	Os_chr08	5644959	[G/T]	NON-SYNONYMOUS-CODING
OsMed37_7	LOC_Os08g09770	Os_chr08	5644877	[C/G]	NON-SYNONYMOUS-CODING
OsMed37_7	LOC_Os08g09770	Os_chr08	5644825	[C/T]	NON-SYNONYMOUS-CODING
OsMed37_7	LOC_Os08g09770	Os_chr08	5644719	[T/C]	REGULATORY
OsMed37_7	LOC_Os08g09770	Os_chr08	5644717	[T/A]	REGULATORY
OsMed37_7	LOC_Os08g09770	Os_chr08	5644712	[C/A]	REGULATORY
OsMed37_7	LOC_Os08g09770	Os_chr08	5644681	[G/A]	REGULATORY
OsMed37_7	LOC_Os08g09770	Os_chr08	5647794	[T/A]	REGULATORY
OsMed37_7	LOC_Os08g09770	Os_chr08	5647772	[T/C]	REGULATORY
OsMed37_7	LOC_Os08g09770	Os_chr08	5647750	[A/C]	REGULATORY
OsMed37_7	LOC_Os08g09770	Os_chr08	5647663	[T/C]	REGULATORY
OsMed37_7	LOC_Os08g09770	Os_chr08	5647642	[C/T]	REGULATORY
OsMed37_7	LOC_Os08g09770	Os_chr08	5647637	[C/A]	REGULATORY
OsMed37_7	LOC_Os08g09770	Os_chr08	5647606	[T/A]	REGULATORY
OsMed37_7	LOC_Os08g09770	Os_chr08	5647537	[C/A]	REGULATORY
OsMed37_7	LOC_Os08g09770	Os_chr08	5647515	[C/T]	REGULATORY
OsMed37_7	LOC_Os08g09770	Os_chr08	5647514	[C/T]	REGULATORY
OsMed37_7	LOC_Os08g09770	Os_chr08	5647458	[T/C]	REGULATORY
OsMed37_7	LOC_Os08g09770	Os_chr08	5647457	[G/A]	REGULATORY
OsMed37_7	LOC_Os08g09770	Os_chr08	5647452	[T/C]	REGULATORY
OsMed37_7	LOC_Os08g09770	Os_chr08	5647450	[C/T]	REGULATORY
OsMed37_7	LOC_Os08g09770	Os_chr08	5647413	[G/A]	REGULATORY
OsMed37_7	LOC_Os08g09770	Os_chr08	5647374	[C/T]	REGULATORY
OsMed37_7	LOC_Os08g09770	Os_chr08	5647367	[T/G]	REGULATORY
OsMed37_7	LOC_Os08g09770	Os_chr08	5647358	[C/T]	REGULATORY
OsMed37_7	LOC_Os08g09770	Os_chr08	5647355	[C/T]	REGULATORY

**Mediator genes**	**MSU locus ID**	**Chromosomes**	**Physical Position (bp)**	**SNPs**	**Structural Annotation**
OsMed37_7	LOC_Os08g09770	Os_chr08	5647343	[T/A]	REGULATORY
OsMed37_7	LOC_Os08g09770	Os_chr08	5647325	[C/T]	REGULATORY
OsMed37_7	LOC_Os08g09770	Os_chr08	5647302	[C/A]	REGULATORY
OsMed37_7	LOC_Os08g09770	Os_chr08	5647253	[C/T]	REGULATORY
OsMed37_7	LOC_Os08g09770	Os_chr08	5647229	[G/A]	REGULATORY
OsMed37_7	LOC_Os08g09770	Os_chr08	5647219	[A/G]	REGULATORY
OsMed37_7	LOC_Os08g09770	Os_chr08	5647215	[T/A]	REGULATORY
OsMed37_7	LOC_Os08g09770	Os_chr08	5647213	[G/A]	REGULATORY
OsMed37_7	LOC_Os08g09770	Os_chr08	5647207	[T/C]	REGULATORY
OsMed37_7	LOC_Os08g09770	Os_chr08	5647200	[G/A]	REGULATORY
OsMed37_7	LOC_Os08g09770	Os_chr08	5647196	[G/A]	REGULATORY
OsMed37_7	LOC_Os08g09770	Os_chr08	5647195	[T/A]	REGULATORY
OsMed37_7	LOC_Os08g09770	Os_chr08	5647175	[G/A]	REGULATORY
OsMed37_7	LOC_Os08g09770	Os_chr08	5647157	[C/T]	REGULATORY
OsMed37_7	LOC_Os08g09770	Os_chr08	5647131	[A/G]	REGULATORY
OsMed37_7	LOC_Os08g09770	Os_chr08	5647129	[T/C]	REGULATORY
OsMed37_7	LOC_Os08g09770	Os_chr08	5647077	[T/C]	REGULATORY
OsMed37_7	LOC_Os08g09770	Os_chr08	5647055	[G/T]	REGULATORY
OsMed37_7	LOC_Os08g09770	Os_chr08	5646993	[A/G]	REGULATORY
OsMed37_7	LOC_Os08g09770	Os_chr08	5646918	[G/A]	REGULATORY
OsMed37_7	LOC_Os08g09770	Os_chr08	5646906	[A/C]	REGULATORY
OsMed37_7	LOC_Os08g09770	Os_chr08	5646884	[G/A]	REGULATORY
OsMed37_7	LOC_Os08g09770	Os_chr08	5646581	[G/A]	SYNONYMOUS-CODING
OsMed37_7	LOC_Os08g09770	Os_chr08	5646518	[A/G]	SYNONYMOUS-CODING
OsMed37_7	LOC_Os08g09770	Os_chr08	5646251	[G/C]	SYNONYMOUS-CODING
OsMed37_7	LOC_Os08g09770	Os_chr08	5646128	[G/A]	SYNONYMOUS-CODING
OsMed37_7	LOC_Os08g09770	Os_chr08	5645900	[G/A]	SYNONYMOUS-CODING
OsMed37_7	LOC_Os08g09770	Os_chr08	5645879	[G/A]	SYNONYMOUS-CODING
OsMed37_7	LOC_Os08g09770	Os_chr08	5645783	[G/A]	SYNONYMOUS-CODING
OsMed37_7	LOC_Os08g09770	Os_chr08	5645771	[C/T]	SYNONYMOUS-CODING
OsMed37_7	LOC_Os08g09770	Os_chr08	5645702	[G/A]	SYNONYMOUS-CODING
OsMed37_7	LOC_Os08g09770	Os_chr08	5645552	[G/T]	SYNONYMOUS-CODING
OsMed37_7	LOC_Os08g09770	Os_chr08	5645366	[C/T]	SYNONYMOUS-CODING
OsMed37_7	LOC_Os08g09770	Os_chr08	5645060	[G/C]	SYNONYMOUS-CODING
OsMed37_7	LOC_Os08g09770	Os_chr08	5645012	[G/C]	SYNONYMOUS-CODING
OsMed37_7	LOC_Os08g09770	Os_chr08	5644891	[G/A]	SYNONYMOUS-CODING
OsMed37_8	LOC_Os11g47760	Os_chr11	28283202	[C/G]	INTRON
OsMed37_8	LOC_Os11g47760	Os_chr11	28283196	[A/T]	INTRON
OsMed37_8	LOC_Os11g47760	Os_chr11	28283106	[A/G]	INTRON
OsMed37_8	LOC_Os11g47760	Os_chr11	28283090	[G/A]	INTRON
OsMed37_8	LOC_Os11g47760	Os_chr11	28282999	[C/T]	INTRON
OsMed37_8	LOC_Os11g47760	Os_chr11	28282981	[T/G]	INTRON
OsMed37_8	LOC_Os11g47760	Os_chr11	28282979	[G/T]	INTRON
OsMed37_8	LOC_Os11g47760	Os_chr11	28282939	[C/T]	INTRON
OsMed37_8	LOC_Os11g47760	Os_chr11	28282922	[C/A]	INTRON
OsMed37_8	LOC_Os11g47760	Os_chr11	28282831	[C/T]	INTRON
OsMed37_8	LOC_Os11g47760	Os_chr11	28282785	[T/G]	INTRON

Mediator genes	MSU locus ID	Chromosomes	Physical Position (bp)	SNPs	Structural Annotation
OsMed37_8	LOC_Os11g47760	Os_chr11	28282783	[C/G]	INTRON
OsMed37_8	LOC_Os11g47760	Os_chr11	28282748	[T/C]	INTRON
OsMed37_8	LOC_Os11g47760	Os_chr11	28282710	[T/A]	INTRON
OsMed37_8	LOC_Os11g47760	Os_chr11	28282640	[C/A]	INTRON
OsMed37_8	LOC_Os11g47760	Os_chr11	28282602	[G/C]	INTRON
OsMed37_8	LOC_Os11g47760	Os_chr11	28282601	[T/G]	INTRON
OsMed37_8	LOC_Os11g47760	Os_chr11	28282531	[C/T]	INTRON
OsMed37_8	LOC_Os11g47760	Os_chr11	28282492	[A/T]	INTRON
OsMed37_8	LOC_Os11g47760	Os_chr11	28282471	[T/A]	INTRON
OsMed37_8	LOC_Os11g47760	Os_chr11	28282436	[T/C]	INTRON
OsMed37_8	LOC_Os11g47760	Os_chr11	28282399	[T/C]	INTRON
OsMed37_8	LOC_Os11g47760	Os_chr11	28282325	[T/A]	INTRON
OsMed37_8	LOC_Os11g47760	Os_chr11	28282323	[T/G]	INTRON
OsMed37_8	LOC_Os11g47760	Os_chr11	28282256	[T/A]	INTRON
OsMed37_8	LOC_Os11g47760	Os_chr11	28282217	[A/G]	INTRON
OsMed37_8	LOC_Os11g47760	Os_chr11	28282197	[C/A]	INTRON
OsMed37_8	LOC_Os11g47760	Os_chr11	28282195	[C/G]	INTRON
OsMed37_8	LOC_Os11g47760	Os_chr11	28282154	[A/G]	INTRON
OsMed37_8	LOC_Os11g47760	Os_chr11	28282135	[C/T]	INTRON
OsMed37_8	LOC_Os11g47760	Os_chr11	28282129	[T/C]	INTRON
OsMed37_8	LOC_Os11g47760	Os_chr11	28282126	[A/C]	INTRON
OsMed37_8	LOC_Os11g47760	Os_chr11	28281900	[A/T]	INTRON
OsMed37_8	LOC_Os11g47760	Os_chr11	28281839	[G/A]	INTRON
OsMed37_8	LOC_Os11g47760	Os_chr11	28281838	[A/G]	INTRON
OsMed37_8	LOC_Os11g47760	Os_chr11	28281822	[A/G]	INTRON
OsMed37_8	LOC_Os11g47760	Os_chr11	28281781	[A/G]	INTRON
OsMed37_8	LOC_Os11g47760	Os_chr11	28281768	[T/A]	INTRON
OsMed37_8	LOC_Os11g47760	Os_chr11	28281753	[T/A]	INTRON
OsMed37_8	LOC_Os11g47760	Os_chr11	28281735	[T/C]	INTRON
OsMed37_8	LOC_Os11g47760	Os_chr11	28281711	[C/T]	INTRON
OsMed37_8	LOC_Os11g47760	Os_chr11	28281691	[A/G]	INTRON
OsMed37_8	LOC_Os11g47760	Os_chr11	28281669	[T/A]	INTRON
OsMed37_8	LOC_Os11g47760	Os_chr11	28281661	[C/A]	INTRON
OsMed37_8	LOC_Os11g47760	Os_chr11	28281632	[T/G]	INTRON
OsMed37_8	LOC_Os11g47760	Os_chr11	28281627	[A/G]	INTRON
OsMed37_8	LOC_Os11g47760	Os_chr11	28281611	[C/T]	INTRON
OsMed37_8	LOC_Os11g47760	Os_chr11	28281594	[A/G]	INTRON
OsMed37_8	LOC_Os11g47760	Os_chr11	28281584	[A/T]	INTRON
OsMed37_8	LOC_Os11g47760	Os_chr11	28281571	[C/A]	INTRON
OsMed37_8	LOC_Os11g47760	Os_chr11	28281565	[T/G]	INTRON
OsMed37_8	LOC_Os11g47760	Os_chr11	28281527	[A/C]	INTRON
OsMed37_8	LOC_Os11g47760	Os_chr11	28281457	[G/A]	INTRON
OsMed37_8	LOC_Os11g47760	Os_chr11	28281456	[G/T]	INTRON
OsMed37_8	LOC_Os11g47760	Os_chr11	28281389	[G/A]	INTRON
OsMed37_8	LOC_Os11g47760	Os_chr11	28281337	[G/T]	INTRON
OsMed37_8	LOC_Os11g47750	Os_chr11	28280328	[C/T]	NON-SYNONYMOUS-CODING
OsMed37_8	LOC_Os11g47750	Os_chr11	28280248	[C/T]	NON-SYNONYMOUS-CODING

Mediator genes	MSU locus ID	Chromosomes	Physical Position (bp)	SNPs	Structural Annotation
OsMed37_8	LOC_Os11g47750	Os_chr11	28280245	[C/T]	NON-SYNONYMOUS-CODING
OsMed37_8	LOC_Os11g47750	Os_chr11	28280227	[T/A]	NON-SYNONYMOUS-CODING
OsMed37_8	LOC_Os11g47750	Os_chr11	28280211	[G/T]	NON-SYNONYMOUS-CODING
OsMed37_8	LOC_Os11g47750	Os_chr11	28280191	[C/T]	NON-SYNONYMOUS-CODING
OsMed37_8	LOC_Os11g47750	Os_chr11	28280154	[G/C]	NON-SYNONYMOUS-CODING
OsMed37_8	LOC_Os11g47750	Os_chr11	28280149	[G/T]	NON-SYNONYMOUS-CODING
OsMed37_8	LOC_Os11g47750	Os_chr11	28280118	[G/A]	NON-SYNONYMOUS-CODING
OsMed37_8	LOC_Os11g47750	Os_chr11	28280103	[T/C]	NON-SYNONYMOUS-CODING
OsMed37_8	LOC_Os11g47760	Os_chr11	28283876	[C/T]	NON-SYNONYMOUS-CODING
OsMed37_8	LOC_Os11g47760	Os_chr11	28281055	[C/T]	REGULATORY
OsMed37_8	LOC_Os11g47760	Os_chr11	28281031	[C/T]	REGULATORY
OsMed37_8	LOC_Os11g47760	Os_chr11	28281029	[G/T]	REGULATORY
OsMed37_8	LOC_Os11g47760	Os_chr11	28281028	[A/C]	REGULATORY
OsMed37_8	LOC_Os11g47750	Os_chr11	28280053	[C/A]	REGULATORY
OsMed37_8	LOC_Os11g47760	Os_chr11	28284929	[G/A]	REGULATORY
OsMed37_8	LOC_Os11g47760	Os_chr11	28284951	[G/A]	REGULATORY
OsMed37_8	LOC_Os11g47760	Os_chr11	28284912	[C/T]	REGULATORY
OsMed37_8	LOC_Os11g47760	Os_chr11	28285294	[G/C]	REGULATORY
OsMed37_8	LOC_Os11g47760	Os_chr11	28285292	[T/C]	REGULATORY
OsMed37_8	LOC_Os11g47760	Os_chr11	28285280	[A/T]	REGULATORY
OsMed37_8	LOC_Os11g47760	Os_chr11	28285255	[C/T]	REGULATORY
OsMed37_8	LOC_Os11g47760	Os_chr11	28285245	[G/T]	REGULATORY
OsMed37_8	LOC_Os11g47760	Os_chr11	28285235	[G/T]	REGULATORY
OsMed37_8	LOC_Os11g47760	Os_chr11	28285229	[T/A]	REGULATORY
OsMed37_8	LOC_Os11g47760	Os_chr11	28285199	[C/G]	REGULATORY
OsMed37_8	LOC_Os11g47760	Os_chr11	28285096	[C/T]	REGULATORY
OsMed37_8	LOC_Os11g47760	Os_chr11	28285065	[A/G]	REGULATORY
OsMed37_8	LOC_Os11g47760	Os_chr11	28285063	[C/T]	REGULATORY
OsMed37_8	LOC_Os11g47760	Os_chr11	28285031	[T/G]	REGULATORY
OsMed37_8	LOC_Os11g47760	Os_chr11	28284998	[C/T]	REGULATORY
OsMed37_8	LOC_Os11g47760	Os_chr11	28281021	[A/T]	REGULATORY
OsMed37_8	LOC_Os11g47760	Os_chr11	28280961	[G/A]	REGULATORY
OsMed37_8	LOC_Os11g47760	Os_chr11	28280959	[C/T]	REGULATORY
OsMed37_8	LOC_Os11g47760	Os_chr11	28280931	[G/C]	REGULATORY
OsMed37_8	LOC_Os11g47760	Os_chr11	28280927	[C/G]	REGULATORY
OsMed37_8	LOC_Os11g47760	Os_chr11	28280903	[G/T]	REGULATORY
OsMed37_8	LOC_Os11g47760	Os_chr11	28280902	[C/G]	REGULATORY
OsMed37_8	LOC_Os11g47760	Os_chr11	28280869	[T/C]	REGULATORY
OsMed37_8	LOC_Os11g47760	Os_chr11	28280840	[C/T]	REGULATORY
OsMed37_8	LOC_Os11g47760	Os_chr11	28280821	[G/A]	REGULATORY
OsMed37_8	LOC_Os11g47760	Os_chr11	28280811	[G/T]	REGULATORY
OsMed37_8	LOC_Os11g47760	Os_chr11	28280767	[C/T]	REGULATORY
OsMed37_8	LOC_Os11g47760	Os_chr11	28280749	[T/C]	REGULATORY
OsMed37_8	LOC_Os11g47760	Os_chr11	28280737	[G/A]	REGULATORY
OsMed37_8	LOC_Os11g47760	Os_chr11	28280710	[C/G]	REGULATORY
OsMed37_8	LOC_Os11g47760	Os_chr11	28280707	[G/A]	REGULATORY
OsMed37_8	LOC_Os11g47760	Os_chr11	28280682	[T/C]	REGULATORY

**Mediator genes**	**MSU locus ID**	**Chromosomes**	**Physical Position (bp)**	**SNPs**	**Structural Annotation**
OsMed37_8	LOC_Os11g47760	Os_chr11	28280681	[T/C]	REGULATORY
OsMed37_8	LOC_Os11g47760	Os_chr11	28280675	[A/G]	REGULATORY
OsMed37_8	LOC_Os11g47760	Os_chr11	28280671	[A/G]	REGULATORY
OsMed37_8	LOC_Os11g47760	Os_chr11	28280667	[C/T]	REGULATORY
OsMed37_8	LOC_Os11g47760	Os_chr11	28280662	[C/A]	REGULATORY
OsMed37_8	LOC_Os11g47760	Os_chr11	28280661	[T/A]	REGULATORY
OsMed37_8	LOC_Os11g47760	Os_chr11	28280658	[A/G]	REGULATORY
OsMed37_8	LOC_Os11g47760	Os_chr11	28280655	[C/T]	REGULATORY
OsMed37_8	LOC_Os11g47760	Os_chr11	28280650	[C/T]	REGULATORY
OsMed37_8	LOC_Os11g47760	Os_chr11	28280644	[A/G]	REGULATORY
OsMed37_8	LOC_Os11g47760	Os_chr11	28280643	[C/T]	REGULATORY
OsMed37_8	LOC_Os11g47760	Os_chr11	28280636	[A/G]	REGULATORY
OsMed37_8	LOC_Os11g47760	Os_chr11	28280632	[C/T]	REGULATORY
OsMed37_8	LOC_Os11g47760	Os_chr11	28280631	[A/G]	REGULATORY
OsMed37_8	LOC_Os11g47760	Os_chr11	28280627	[G/A]	REGULATORY
OsMed37_8	LOC_Os11g47760	Os_chr11	28280618	[G/A]	REGULATORY
OsMed37_8	LOC_Os11g47760	Os_chr11	28280502	[G/A]	REGULATORY
OsMed37_8	LOC_Os11g47760	Os_chr11	28280494	[C/G]	REGULATORY
OsMed37_8	LOC_Os11g47760	Os_chr11	28280490	[G/C]	REGULATORY
OsMed37_8	LOC_Os11g47760	Os_chr11	28280468	[A/G]	REGULATORY
OsMed37_8	LOC_Os11g47760	Os_chr11	28280462	[G/A]	REGULATORY
OsMed37_8	LOC_Os11g47750	Os_chr11	28280339	[G/A]	SYNONYMOUS-CODING
OsMed37_8	LOC_Os11g47750	Os_chr11	28280180	[T/G]	SYNONYMOUS-CODING
OsMed37_8	LOC_Os11g47760	Os_chr11	28281318	[C/T]	SYNONYMOUS-CODING
OsMed37_8	LOC_Os11g47760	Os_chr11	28281174	[T/G]	SYNONYMOUS-CODING
OsMed37_8	LOC_Os11g47760	Os_chr11	28281156	[C/A]	SYNONYMOUS-CODING
OsMed37_8	LOC_Os11g47760	Os_chr11	28281153	[G/T]	SYNONYMOUS-CODING
OsMed37_8	LOC_Os11g47760	Os_chr11	28281147	[T/G]	SYNONYMOUS-CODING
OsMed37_8	LOC_Os11g47760	Os_chr11	28283901	[T/C]	SYNONYMOUS-CODING
OsMed37_8	LOC_Os11g47760	Os_chr11	28283857	[C/T]	SYNONYMOUS-CODING
OsMed4_1	LOC_Os09g36890	Os_chr09	21274027	[T/C]	INTRON
OsMed4_1	LOC_Os09g36890	Os_chr09	21273819	[C/A]	INTRON
OsMed4_1	LOC_Os09g36890	Os_chr09	21274537	[T/A]	INTRON
OsMed4_1	LOC_Os09g36890	Os_chr09	21274332	[C/T]	INTRON
OsMed4_1	LOC_Os09g36890	Os_chr09	21274258	[G/A]	INTRON
OsMed4_1	LOC_Os09g36900	Os_chr09	21276780	[C/A]	INTRON
OsMed4_1	LOC_Os09g36900	Os_chr09	21276689	[C/A]	INTRON
OsMed4_1	LOC_Os09g36890	Os_chr09	21275709	[C/A]	NON-SYNONYMOUS-CODING
OsMed4_1	LOC_Os09g36890	Os_chr09	21274906	[C/T]	NON-SYNONYMOUS-CODING
OsMed4_1	LOC_Os09g36890	Os_chr09	21274695	[T/G]	NON-SYNONYMOUS-CODING
OsMed4_1	LOC_Os09g36900	Os_chr09	21276236	[C/A]	NON-SYNONYMOUS-CODING
OsMed4_1	LOC_Os09g36900	Os_chr09	21276233	[G/T]	NON-SYNONYMOUS-CODING
OsMed4_1	LOC_Os09g36890	Os_chr09	21273341	[G/A]	REGULATORY
OsMed4_1	LOC_Os09g36900	Os_chr09	21275952	[T/A]	REGULATORY
OsMed4_1	LOC_Os09g36900	Os_chr09	21275887	[A/T]	REGULATORY
OsMed4_1	LOC_Os09g36900	Os_chr09	21275884	[C/T]	REGULATORY
OsMed4_1	LOC_Os09g36890	Os_chr09	21275285	[C/T]	SYNONYMOUS-CODING

**Mediator genes**	**MSU locus ID**	**Chromosomes**	**Physical Position (bp)**	**SNPs**	**Structural Annotation**
OsMed4_1	LOC_Os09g36890	Os_chr09	21274748	[C/T]	SYNONYMOUS-CODING
OsMed4_1	LOC_Os09g36890	Os_chr09	21274634	[C/T]	SYNONYMOUS-CODING
OsMed4_2	LOC_Os11g05150	Os_chr11	2261046	[G/A]	INTRON
OsMed4_2	LOC_Os11g05150	Os_chr11	2261017	[G/T]	INTRON
OsMed4_2	LOC_Os11g05150	Os_chr11	2260945	[C/T]	INTRON
OsMed4_2	LOC_Os11g05150	Os_chr11	2260918	[C/T]	INTRON
OsMed4_2	LOC_Os11g05150	Os_chr11	2260909	[T/C]	INTRON
OsMed4_2	LOC_Os11g05150	Os_chr11	2260878	[T/A]	INTRON
OsMed4_2	LOC_Os11g05150	Os_chr11	2260861	[C/T]	INTRON
OsMed4_2	LOC_Os11g05150	Os_chr11	2261164	[G/T]	NON-SYNONYMOUS-CODING
OsMed4_2	LOC_Os11g05150	Os_chr11	2260726	[G/A]	NON-SYNONYMOUS-CODING
OsMed4_2	LOC_Os11g05150	Os_chr11	2260577	[C/T]	NON-SYNONYMOUS-CODING
OsMed4_2	LOC_Os11g05150	Os_chr11	2261376	[C/T]	REGULATORY
OsMed4_2	LOC_Os11g05150	Os_chr11	2261331	[G/T]	REGULATORY
OsMed4_2	LOC_Os11g05150	Os_chr11	2260698	[C/T]	SYNONYMOUS-CODING
OsMed4_2	LOC_Os11g05150	Os_chr11	2260686	[G/A]	SYNONYMOUS-CODING
OsMed4_2	LOC_Os11g05150	Os_chr11	2260656	[G/T]	SYNONYMOUS-CODING
OsMed4_2	LOC_Os11g05150	Os_chr11	2260607	[G/A]	SYNONYMOUS-CODING
OsMed4_2	LOC_Os11g05150	Os_chr11	2260572	[T/C]	SYNONYMOUS-CODING
OsMed5_1	LOC_Os05g24684	Os_chr05	14250894	[C/T]	INTRON
OsMed5_1	LOC_Os05g24684	Os_chr05	14250864	[C/T]	INTRON
OsMed5_1	LOC_Os05g24684	Os_chr05	14250861	[A/C]	INTRON
OsMed5_1	LOC_Os05g24684	Os_chr05	14250653	[G/C]	INTRON
OsMed5_1	LOC_Os05g24684	Os_chr05	14250298	[G/A]	INTRON
OsMed5_1	LOC_Os05g24684	Os_chr05	14250209	[G/A]	INTRON
OsMed5_1	LOC_Os05g24684	Os_chr05	14249946	[T/C]	INTRON
OsMed5_1	LOC_Os05g24684	Os_chr05	14249607	[G/A]	INTRON
OsMed5_1	LOC_Os05g24684	Os_chr05	14249604	[T/A]	INTRON
OsMed5_1	LOC_Os05g24684	Os_chr05	14249468	[A/G]	INTRON
OsMed5_1	LOC_Os05g24684	Os_chr05	14249326	[C/A]	INTRON
OsMed5_1	LOC_Os05g24684	Os_chr05	14249314	[G/A]	INTRON
OsMed5_1	LOC_Os05g24684	Os_chr05	14248423	[C/A]	INTRON
OsMed5_1	LOC_Os05g24684	Os_chr05	14247827	[G/A]	INTRON
OsMed5_1	LOC_Os05g24684	Os_chr05	14247785	[C/G]	INTRON
OsMed5_1	LOC_Os05g24684	Os_chr05	14247588	[C/T]	INTRON
OsMed5_1	LOC_Os05g24684	Os_chr05	14247416	[A/C]	INTRON
OsMed5_1	LOC_Os05g24684	Os_chr05	14246967	[A/G]	INTRON
OsMed5_1	LOC_Os05g24684	Os_chr05	14246861	[G/C]	INTRON
OsMed5_1	LOC_Os05g24684	Os_chr05	14246686	[A/C]	INTRON
OsMed5_1	LOC_Os05g24684	Os_chr05	14246483	[C/G]	INTRON
OsMed5_1	LOC_Os05g24684	Os_chr05	14246400	[C/T]	INTRON
OsMed5_1	LOC_Os05g24684	Os_chr05	14245851	[A/T]	INTRON
OsMed5_1	LOC_Os05g24684	Os_chr05	14245083	[T/C]	INTRON
OsMed5_1	LOC_Os05g24684	Os_chr05	14244964	[G/A]	INTRON
OsMed5_1	LOC_Os05g24684	Os_chr05	14243426	[G/C]	INTRON
OsMed5_1	LOC_Os05g24684	Os_chr05	14243359	[G/A]	INTRON
OsMed5_1	LOC_Os05g24684	Os_chr05	14243358	[C/T]	INTRON

Mediator genes	MSU locus ID	Chromosomes	Physical Position (bp)	SNPs	Structural Annotation
OsMed5_1	LOC_Os05g24684	Os_chr05	14243343	[G/A]	INTRON
OsMed5_1	LOC_Os05g24684	Os_chr05	14241609	[T/A]	INTRON
OsMed5_1	LOC_Os05g24684	Os_chr05	14241595	[G/A]	INTRON
OsMed5_1	LOC_Os05g24684	Os_chr05	14241579	[A/G]	INTRON
OsMed5_1	LOC_Os05g24684	Os_chr05	14240906	[G/A]	INTRON
OsMed5_1	LOC_Os05g24684	Os_chr05	14240776	[C/A]	INTRON
OsMed5_1	LOC_Os05g24684	Os_chr05	14240592	[C/A]	INTRON
OsMed5_1	LOC_Os05g24684	Os_chr05	14240079	[A/G]	INTRON
OsMed5_1	LOC_Os05g24684	Os_chr05	14239976	[C/T]	INTRON
OsMed5_1	LOC_Os05g24684	Os_chr05	14239944	[C/A]	INTRON
OsMed5_1	LOC_Os05g24684	Os_chr05	14239781	[C/A]	INTRON
OsMed5_1	LOC_Os05g24684	Os_chr05	14239302	[G/A]	INTRON
OsMed5_1	LOC_Os05g24684	Os_chr05	14239052	[C/A]	INTRON
OsMed5_1	LOC_Os05g24684	Os_chr05	14245932	[G/T]	NON-SYNONYMOUS-CODING
OsMed5_1	LOC_Os05g24684	Os_chr05	14244026	[C/T]	NON-SYNONYMOUS-CODING
OsMed5_1	LOC_Os05g24684	Os_chr05	14242951	[G/A]	NON-SYNONYMOUS-CODING
OsMed5_1	LOC_Os05g24684	Os_chr05	14240248	[G/A]	NON-SYNONYMOUS-CODING
OsMed5_1	LOC_Os05g24684	Os_chr05	14238287	[C/T]	NON-SYNONYMOUS-CODING
OsMed5_1	LOC_Os05g24684	Os_chr05	14251359	[C/T]	REGULATORY
OsMed5_1	LOC_Os05g24684	Os_chr05	14251315	[A/G]	REGULATORY
OsMed5_1	LOC_Os05g24684	Os_chr05	14237751	[A/G]	REGULATORY
OsMed5_1	LOC_Os05g24684	Os_chr05	14237491	[T/A]	REGULATORY
OsMed5_1	LOC_Os05g24684	Os_chr05	14252323	[G/A]	REGULATORY
OsMed5_1	LOC_Os05g24684	Os_chr05	14252318	[G/A]	REGULATORY
OsMed5_1	LOC_Os05g24684	Os_chr05	14252273	[T/C]	REGULATORY
OsMed5_1	LOC_Os05g24684	Os_chr05	14252248	[G/A]	REGULATORY
OsMed5_1	LOC_Os05g24684	Os_chr05	14252245	[G/A]	REGULATORY
OsMed5_1	LOC_Os05g24684	Os_chr05	14252237	[C/T]	REGULATORY
OsMed5_1	LOC_Os05g24684	Os_chr05	14252202	[G/A]	REGULATORY
OsMed5_1	LOC_Os05g24684	Os_chr05	14252096	[C/A]	REGULATORY
OsMed5_1	LOC_Os05g24684	Os_chr05	14252068	[C/T]	REGULATORY
OsMed5_1	LOC_Os05g24684	Os_chr05	14252034	[C/T]	REGULATORY
OsMed5_1	LOC_Os05g24684	Os_chr05	14251976	[G/A]	REGULATORY
OsMed5_1	LOC_Os05g24684	Os_chr05	14251949	[T/C]	REGULATORY
OsMed5_1	LOC_Os05g24684	Os_chr05	14251903	[G/C]	REGULATORY
OsMed5_1	LOC_Os05g24684	Os_chr05	14251873	[C/T]	REGULATORY
OsMed5_1	LOC_Os05g24684	Os_chr05	14251844	[A/G]	REGULATORY
OsMed5_1	LOC_Os05g24684	Os_chr05	14251739	[G/A]	REGULATORY
OsMed5_1	LOC_Os05g24684	Os_chr05	14251727	[A/G]	REGULATORY
OsMed5_1	LOC_Os05g24684	Os_chr05	14251690	[G/A]	REGULATORY
OsMed5_1	LOC_Os05g24684	Os_chr05	14251678	[G/A]	REGULATORY
OsMed5_1	LOC_Os05g24684	Os_chr05	14251659	[C/T]	REGULATORY
OsMed5_1	LOC_Os05g24684	Os_chr05	14251649	[G/A]	REGULATORY
OsMed5_1	LOC_Os05g24684	Os_chr05	14251648	[T/C]	REGULATORY
OsMed5_1	LOC_Os05g24684	Os_chr05	14251620	[G/A]	REGULATORY
OsMed5_1	LOC_Os05g24684	Os_chr05	14251618	[C/T]	REGULATORY
OsMed5_1	LOC_Os05g24684	Os_chr05	14251603	[C/T]	REGULATORY

**Mediator genes**	**MSU locus ID**	**Chromosomes**	**Physical Position (bp)**	**SNPs**	**Structural Annotation**
OsMed5_1	LOC_Os05g24684	Os_chr05	14251599	[T/C]	REGULATORY
OsMed5_1	LOC_Os05g24684	Os_chr05	14251594	[T/C]	REGULATORY
OsMed5_1	LOC_Os05g24684	Os_chr05	14251576	[C/T]	REGULATORY
OsMed5_1	LOC_Os05g24684	Os_chr05	14251575	[A/G]	REGULATORY
OsMed5_1	LOC_Os05g24684	Os_chr05	14251549	[A/G]	REGULATORY
OsMed5_1	LOC_Os05g24684	Os_chr05	14251491	[A/G]	REGULATORY
OsMed5_1	LOC_Os05g24684	Os_chr05	14251477	[C/A]	REGULATORY
OsMed5_1	LOC_Os05g24684	Os_chr05	14251457	[T/C]	REGULATORY
OsMed5_1	LOC_Os05g24684	Os_chr05	14251430	[T/C]	REGULATORY
OsMed5_1	LOC_Os05g24684	Os_chr05	14244636	[G/A]	SYNONYMOUS-CODING
OsMed5_1	LOC_Os05g24684	Os_chr05	14244462	[A/T]	SYNONYMOUS-CODING
OsMed5_1	LOC_Os05g24684	Os_chr05	14238485	[T/C]	SYNONYMOUS-CODING
OsMed5_1	LOC_Os05g24684	Os_chr05	14237966	[G/A]	SYNONYMOUS-CODING
OsMed5_1	LOC_Os05g24684	Os_chr05	14237942	[A/G]	SYNONYMOUS-CODING
OsMed5_2	LOC_Os07g11000	Os_chr07	6041924	[A/C]	INTRON
OsMed5_2	LOC_Os07g11000	Os_chr07	6041828	[A/T]	INTRON
OsMed5_2	LOC_Os07g11000	Os_chr07	6041596	[A/G]	INTRON
OsMed5_2	LOC_Os07g11000	Os_chr07	6041536	[T/G]	INTRON
OsMed5_2	LOC_Os07g11000	Os_chr07	6041532	[T/G]	INTRON
OsMed5_2	LOC_Os07g11000	Os_chr07	6040780	[T/C]	INTRON
OsMed5_2	LOC_Os07g11000	Os_chr07	6040311	[G/A]	INTRON
OsMed5_2	LOC_Os07g11000	Os_chr07	6040299	[C/T]	INTRON
OsMed5_2	LOC_Os07g11000	Os_chr07	6040248	[A/G]	INTRON
OsMed5_2	LOC_Os07g11000	Os_chr07	6040210	[G/A]	INTRON
OsMed5_2	LOC_Os07g11000	Os_chr07	6039926	[A/T]	INTRON
OsMed5_2	LOC_Os07g11000	Os_chr07	6039709	[T/C]	INTRON
OsMed5_2	LOC_Os07g11000	Os_chr07	6039675	[T/C]	INTRON
OsMed5_2	LOC_Os07g11000	Os_chr07	6039597	[T/C]	INTRON
OsMed5_2	LOC_Os07g11000	Os_chr07	6039540	[A/C]	INTRON
OsMed5_2	LOC_Os07g11000	Os_chr07	6039527	[T/C]	INTRON
OsMed5_2	LOC_Os07g11000	Os_chr07	6039332	[T/C]	INTRON
OsMed5_2	LOC_Os07g11000	Os_chr07	6038777	[T/C]	INTRON
OsMed5_2	LOC_Os07g11000	Os_chr07	6038128	[T/C]	INTRON
OsMed5_2	LOC_Os07g11000	Os_chr07	6037257	[T/C]	INTRON
OsMed5_2	LOC_Os07g11000	Os_chr07	6036258	[G/A]	INTRON
OsMed5_2	LOC_Os07g11000	Os_chr07	6035908	[G/A]	INTRON
OsMed5_2	LOC_Os07g11000	Os_chr07	6042579	[G/C]	NON-SYNONYMOUS-CODING
OsMed5_2	LOC_Os07g11000	Os_chr07	6041201	[G/T]	NON-SYNONYMOUS-CODING
OsMed5_2	LOC_Os07g11000	Os_chr07	6041057	[G/A]	NON-SYNONYMOUS-CODING
OsMed5_2	LOC_Os07g11000	Os_chr07	6041049	[G/T]	NON-SYNONYMOUS-CODING
OsMed5_2	LOC_Os07g11000	Os_chr07	6038627	[A/G]	NON-SYNONYMOUS-CODING
OsMed5_2	LOC_Os07g11000	Os_chr07	6038052	[C/T]	NON-SYNONYMOUS-CODING
OsMed5_2	LOC_Os07g11000	Os_chr07	6036086	[T/G]	NON-SYNONYMOUS-CODING
OsMed5_2	LOC_Os07g11000	Os_chr07	6043100	[G/A]	REGULATORY
OsMed5_2	LOC_Os07g11000	Os_chr07	6043537	[T/C]	REGULATORY
OsMed5_2	LOC_Os07g11000	Os_chr07	6043513	[G/C]	REGULATORY
OsMed5_2	LOC_Os07g11000	Os_chr07	6043480	[A/G]	REGULATORY

**Mediator genes**	**MSU locus ID**	**Chromosomes**	**Physical Position (bp)**	**SNPs**	**Structural Annotation**
OsMed5_2	LOC_Os07g11000	Os_chr07	6043449	[C/T]	REGULATORY
OsMed5_2	LOC_Os07g11000	Os_chr07	6043441	[G/A]	REGULATORY
OsMed5_2	LOC_Os07g11000	Os_chr07	6043426	[C/T]	REGULATORY
OsMed5_2	LOC_Os07g11000	Os_chr07	6043320	[T/G]	REGULATORY
OsMed5_2	LOC_Os07g11000	Os_chr07	6043243	[A/G]	REGULATORY
OsMed5_2	LOC_Os07g11000	Os_chr07	6043189	[T/A]	REGULATORY
OsMed5_2	LOC_Os07g11000	Os_chr07	6043136	[C/A]	REGULATORY
OsMed5_2	LOC_Os07g11000	Os_chr07	6034926	[C/A]	REGULATORY
OsMed5_2	LOC_Os07g11000	Os_chr07	6034903	[C/A]	REGULATORY
OsMed5_2	LOC_Os07g11000	Os_chr07	6034759	[C/T]	REGULATORY
OsMed5_2	LOC_Os07g11000	Os_chr07	6034741	[G/A]	REGULATORY
OsMed5_2	LOC_Os07g11000	Os_chr07	6034735	[C/T]	REGULATORY
OsMed5_2	LOC_Os07g11000	Os_chr07	6034699	[T/C]	REGULATORY
OsMed5_2	LOC_Os07g11000	Os_chr07	6034689	[G/T]	REGULATORY
OsMed5_2	LOC_Os07g11000	Os_chr07	6034599	[G/A]	REGULATORY
OsMed5_2	LOC_Os07g11000	Os_chr07	6034591	[G/A]	REGULATORY
OsMed5_2	LOC_Os07g11000	Os_chr07	6034510	[A/G]	REGULATORY
OsMed5_2	LOC_Os07g11000	Os_chr07	6034491	[A/C]	REGULATORY
OsMed5_2	LOC_Os07g11000	Os_chr07	6034368	[G/A]	REGULATORY
OsMed5_2	LOC_Os07g11000	Os_chr07	6034361	[C/A]	REGULATORY
OsMed5_2	LOC_Os07g11000	Os_chr07	6034316	[A/T]	REGULATORY
OsMed5_2	LOC_Os07g11000	Os_chr07	6034279	[T/C]	REGULATORY
OsMed5_2	LOC_Os07g11000	Os_chr07	6034276	[C/T]	REGULATORY
OsMed5_2	LOC_Os07g11000	Os_chr07	6034259	[C/T]	REGULATORY
OsMed5_2	LOC_Os07g11000	Os_chr07	6034257	[C/T]	REGULATORY
OsMed5_2	LOC_Os07g11000	Os_chr07	6034243	[A/T]	REGULATORY
OsMed5_2	LOC_Os07g11000	Os_chr07	6034226	[A/G]	REGULATORY
OsMed5_2	LOC_Os07g11000	Os_chr07	6034151	[C/A]	REGULATORY
OsMed5_2	LOC_Os07g11000	Os_chr07	6034141	[A/G]	REGULATORY
OsMed5_2	LOC_Os07g11000	Os_chr07	6034139	[C/A]	REGULATORY
OsMed5_2	LOC_Os07g11000	Os_chr07	6034100	[T/G]	REGULATORY
OsMed5_2	LOC_Os07g11000	Os_chr07	6043025	[C/T]	SYNONYMOUS-CODING
OsMed5_2	LOC_Os07g11000	Os_chr07	6041411	[C/T]	SYNONYMOUS-CODING
OsMed5_2	LOC_Os07g11000	Os_chr07	6040685	[C/T]	SYNONYMOUS-CODING
OsMed5_2	LOC_Os07g11000	Os_chr07	6038446	[C/T]	SYNONYMOUS-CODING
OsMed5_3	LOC_Os07g48350	Os_chr07	28898126	[T/G]	INTRON
OsMed5_3	LOC_Os07g48350	Os_chr07	28898094	[T/G]	INTRON
OsMed5_3	LOC_Os07g48350	Os_chr07	28898075	[C/G]	INTRON
OsMed5_3	LOC_Os07g48350	Os_chr07	28897310	[T/G]	INTRON
OsMed5_3	LOC_Os07g48350	Os_chr07	28897101	[A/G]	INTRON
OsMed5_3	LOC_Os07g48350	Os_chr07	28896513	[G/T]	INTRON
OsMed5_3	LOC_Os07g48350	Os_chr07	28896321	[A/G]	INTRON
OsMed5_3	LOC_Os07g48350	Os_chr07	28896040	[C/A]	INTRON
OsMed5_3	LOC_Os07g48350	Os_chr07	28895826	[T/A]	INTRON
OsMed5_3	LOC_Os07g48350	Os_chr07	28895476	[G/A]	INTRON
OsMed5_3	LOC_Os07g48350	Os_chr07	28895459	[T/A]	INTRON
OsMed5_3	LOC_Os07g48350	Os_chr07	28895396	[C/T]	INTRON

Mediator genes	MSU locus ID	Chromosomes	Physical Position (bp)	SNPs	Structural Annotation
OsMed5_3	LOC_Os07g48350	Os_chr07	28894749	[T/A]	INTRON
OsMed5_3	LOC_Os07g48350	Os_chr07	28894010	[A/T]	INTRON
OsMed5_3	LOC_Os07g48350	Os_chr07	28893359	[A/T]	INTRON
OsMed5_3	LOC_Os07g48350	Os_chr07	28892939	[G/T]	INTRON
OsMed5_3	LOC_Os07g48350	Os_chr07	28892518	[A/G]	INTRON
OsMed5_3	LOC_Os07g48350	Os_chr07	28899486	[G/A]	NON-SYNONYMOUS-CODING
OsMed5_3	LOC_Os07g48350	Os_chr07	28896172	[G/A]	NON-SYNONYMOUS-CODING
OsMed5_3	LOC_Os07g48350	Os_chr07	28893827	[G/A]	NON-SYNONYMOUS-CODING
OsMed5_3	LOC_Os07g48350	Os_chr07	28893653	[C/T]	NON-SYNONYMOUS-CODING
OsMed5_3	LOC_Os07g48350	Os_chr07	28893250	[G/A]	NON-SYNONYMOUS-CODING
OsMed5_3	LOC_Os07g48350	Os_chr07	28892144	[T/A]	REGULATORY
OsMed5_3	LOC_Os07g48350	Os_chr07	28892167	[C/T]	REGULATORY
OsMed5_3	LOC_Os07g48350	Os_chr07	28892108	[C/T]	REGULATORY
OsMed5_3	LOC_Os07g48350	Os_chr07	28892054	[G/T]	REGULATORY
OsMed5_3	LOC_Os07g48350	Os_chr07	28891781	[T/G]	REGULATORY
OsMed5_3	LOC_Os07g48350	Os_chr07	28891753	[A/C]	REGULATORY
OsMed5_3	LOC_Os07g48350	Os_chr07	28891698	[A/C]	REGULATORY
OsMed5_3	LOC_Os07g48350	Os_chr07	28891636	[A/G]	REGULATORY
OsMed5_3	LOC_Os07g48350	Os_chr07	28891604	[C/T]	REGULATORY
OsMed5_3	LOC_Os07g48350	Os_chr07	28891501	[C/T]	REGULATORY
OsMed5_3	LOC_Os07g48350	Os_chr07	28891491	[C/T]	REGULATORY
OsMed5_3	LOC_Os07g48350	Os_chr07	28891479	[A/G]	REGULATORY
OsMed5_3	LOC_Os07g48350	Os_chr07	28891469	[G/A]	REGULATORY
OsMed5_3	LOC_Os07g48350	Os_chr07	28891435	[G/A]	REGULATORY
OsMed5_3	LOC_Os07g48350	Os_chr07	28891428	[T/C]	REGULATORY
OsMed5_3	LOC_Os07g48350	Os_chr07	28891409	[G/A]	REGULATORY
OsMed5_3	LOC_Os07g48350	Os_chr07	28891385	[G/A]	REGULATORY
OsMed5_3	LOC_Os07g48350	Os_chr07	28891377	[C/T]	REGULATORY
OsMed5_3	LOC_Os07g48350	Os_chr07	28891354	[G/A]	REGULATORY
OsMed5_3	LOC_Os07g48350	Os_chr07	28891345	[G/A]	REGULATORY
OsMed5_3	LOC_Os07g48350	Os_chr07	28891229	[T/G]	REGULATORY
OsMed5_3	LOC_Os07g48350	Os_chr07	28891221	[C/T]	REGULATORY
OsMed5_3	LOC_Os07g48350	Os_chr07	28891196	[C/T]	REGULATORY
OsMed5_3	LOC_Os07g48350	Os_chr07	28891191	[C/T]	REGULATORY
OsMed5_3	LOC_Os07g48350	Os_chr07	28891109	[C/A]	REGULATORY
OsMed5_3	LOC_Os07g48350	Os_chr07	28899531	[C/T]	SYNONYMOUS-CODING
OsMed5_3	LOC_Os07g48350	Os_chr07	28898705	[C/T]	SYNONYMOUS-CODING
OsMed5_3	LOC_Os07g48350	Os_chr07	28897683	[C/T]	SYNONYMOUS-CODING
OsMed5_3	LOC_Os07g48350	Os_chr07	28897554	[T/A]	SYNONYMOUS-CODING
OsMed5_3	LOC_Os07g48350	Os_chr07	28893769	[A/C]	SYNONYMOUS-CODING
OsMed6_1	LOC_Os06g11370	Os_chr06	5978629	[C/T]	INTRON
OsMed6_1	LOC_Os06g11370	Os_chr06	5978556	[T/C]	INTRON
OsMed6_1	LOC_Os06g11370	Os_chr06	5978470	[A/G]	INTRON
OsMed6_1	LOC_Os06g11370	Os_chr06	5978446	[G/A]	INTRON
OsMed6_1	LOC_Os06g11370	Os_chr06	5978419	[C/A]	INTRON
OsMed6_1	LOC_Os06g11370	Os_chr06	5978393	[A/T]	INTRON
OsMed6_1	LOC_Os06g11370	Os_chr06	5978375	[A/G]	INTRON

Mediator genes	MSU locus ID	Chromosomes	Physical Position (bp)	SNPs	Structural Annotation
OsMed6_1	LOC_Os06g11370	Os_chr06	5977981	[T/A]	INTRON
OsMed6_1	LOC_Os06g11370	Os_chr06	5977952	[G/T]	INTRON
OsMed6_1	LOC_Os06g11370	Os_chr06	5977937	[G/A]	INTRON
OsMed6_1	LOC_Os06g11370	Os_chr06	5977838	[G/T]	INTRON
OsMed6_1	LOC_Os06g11370	Os_chr06	5977829	[A/G]	INTRON
OsMed6_1	LOC_Os06g11370	Os_chr06	5977819	[T/C]	INTRON
OsMed6_1	LOC_Os06g11370	Os_chr06	5977707	[C/T]	INTRON
OsMed6_1	LOC_Os06g11370	Os_chr06	5977647	[G/A]	INTRON
OsMed6_1	LOC_Os06g11370	Os_chr06	5977571	[A/G]	INTRON
OsMed6_1	LOC_Os06g11370	Os_chr06	5977512	[C/T]	INTRON
OsMed6_1	LOC_Os06g11370	Os_chr06	5976978	[T/A]	INTRON
OsMed6_1	LOC_Os06g11370	Os_chr06	5976867	[G/A]	INTRON
OsMed6_1	LOC_Os06g11370	Os_chr06	5976833	[T/C]	INTRON
OsMed6_1	LOC_Os06g11370	Os_chr06	5979720	[C/G]	REGULATORY
OsMed6_1	LOC_Os06g11370	Os_chr06	5979696	[A/G]	REGULATORY
OsMed6_1	LOC_Os06g11370	Os_chr06	5979617	[G/A]	REGULATORY
OsMed6_1	LOC_Os06g11370	Os_chr06	5976586	[T/C]	REGULATORY
OsMed6_1	LOC_Os06g11370	Os_chr06	5976441	[C/A]	REGULATORY
OsMed6_1	LOC_Os06g11370	Os_chr06	5980813	[A/T]	REGULATORY
OsMed6_1	LOC_Os06g11370	Os_chr06	5980793	[C/T]	REGULATORY
OsMed6_1	LOC_Os06g11370	Os_chr06	5980790	[C/T]	REGULATORY
OsMed6_1	LOC_Os06g11370	Os_chr06	5980783	[G/A]	REGULATORY
OsMed6_1	LOC_Os06g11370	Os_chr06	5980759	[C/T]	REGULATORY
OsMed6_1	LOC_Os06g11370	Os_chr06	5980737	[G/A]	REGULATORY
OsMed6_1	LOC_Os06g11370	Os_chr06	5980735	[T/C]	REGULATORY
OsMed6_1	LOC_Os06g11370	Os_chr06	5980720	[A/G]	REGULATORY
OsMed6_1	LOC_Os06g11370	Os_chr06	5980705	[G/A]	REGULATORY
OsMed6_1	LOC_Os06g11370	Os_chr06	5980658	[T/C]	REGULATORY
OsMed6_1	LOC_Os06g11370	Os_chr06	5980641	[C/T]	REGULATORY
OsMed6_1	LOC_Os06g11370	Os_chr06	5980637	[G/A]	REGULATORY
OsMed6_1	LOC_Os06g11370	Os_chr06	5980625	[A/G]	REGULATORY
OsMed6_1	LOC_Os06g11370	Os_chr06	5980619	[G/A]	REGULATORY
OsMed6_1	LOC_Os06g11370	Os_chr06	5980590	[C/T]	REGULATORY
OsMed6_1	LOC_Os06g11370	Os_chr06	5980589	[G/T]	REGULATORY
OsMed6_1	LOC_Os06g11370	Os_chr06	5980583	[C/T]	REGULATORY
OsMed6_1	LOC_Os06g11370	Os_chr06	5980577	[C/T]	REGULATORY
OsMed6_1	LOC_Os06g11370	Os_chr06	5980491	[G/A]	REGULATORY
OsMed6_1	LOC_Os06g11370	Os_chr06	5980479	[C/T]	REGULATORY
OsMed6_1	LOC_Os06g11370	Os_chr06	5980382	[A/G]	REGULATORY
OsMed6_1	LOC_Os06g11370	Os_chr06	5980281	[G/C]	REGULATORY
OsMed6_1	LOC_Os06g11370	Os_chr06	5980247	[C/T]	REGULATORY
OsMed6_1	LOC_Os06g11370	Os_chr06	5980244	[T/G]	REGULATORY
OsMed6_1	LOC_Os06g11370	Os_chr06	5980233	[G/T]	REGULATORY
OsMed6_1	LOC_Os06g11370	Os_chr06	5980228	[G/A]	REGULATORY
OsMed6_1	LOC_Os06g11370	Os_chr06	5980213	[G/T]	REGULATORY
OsMed6_1	LOC_Os06g11370	Os_chr06	5980200	[C/T]	REGULATORY
OsMed6_1	LOC_Os06g11370	Os_chr06	5980198	[A/G]	REGULATORY

**Mediator genes**	**MSU locus ID**	**Chromosomes**	**Physical Position (bp)**	**SNPs**	**Structural Annotation**
OsMed6_1	LOC_Os06g11370	Os_chr06	5980180	[G/A]	REGULATORY
OsMed6_1	LOC_Os06g11370	Os_chr06	5980148	[C/T]	REGULATORY
OsMed6_1	LOC_Os06g11370	Os_chr06	5980140	[A/G]	REGULATORY
OsMed6_1	LOC_Os06g11370	Os_chr06	5980135	[A/G]	REGULATORY
OsMed6_1	LOC_Os06g11370	Os_chr06	5980092	[C/T]	REGULATORY
OsMed6_1	LOC_Os06g11370	Os_chr06	5980068	[C/T]	REGULATORY
OsMed6_1	LOC_Os06g11370	Os_chr06	5980053	[G/T]	REGULATORY
OsMed6_1	LOC_Os06g11370	Os_chr06	5980046	[C/T]	REGULATORY
OsMed6_1	LOC_Os06g11370	Os_chr06	5980040	[C/T]	REGULATORY
OsMed6_1	LOC_Os06g11370	Os_chr06	5980038	[C/T]	REGULATORY
OsMed6_1	LOC_Os06g11370	Os_chr06	5980031	[C/T]	REGULATORY
OsMed6_1	LOC_Os06g11370	Os_chr06	5980030	[G/A]	REGULATORY
OsMed6_1	LOC_Os06g11370	Os_chr06	5980014	[A/G]	REGULATORY
OsMed6_1	LOC_Os06g11370	Os_chr06	5979977	[T/C]	REGULATORY
OsMed6_1	LOC_Os06g11370	Os_chr06	5979955	[T/C]	REGULATORY
OsMed6_1	LOC_Os06g11370	Os_chr06	5979938	[A/G]	REGULATORY
OsMed6_1	LOC_Os06g11370	Os_chr06	5979937	[C/T]	REGULATORY
OsMed6_1	LOC_Os06g11370	Os_chr06	5979046	[G/C]	SYNONYMOUS-CODING
OsMed6_1	LOC_Os06g11370	Os_chr06	5976732	[T/C]	SYNONYMOUS-CODING
OsMed7_1	LOC_Os04g56640	Os_chr04	33591401	[G/C]	INTRON
OsMed7_1	LOC_Os04g56640	Os_chr04	33591182	[C/T]	INTRON
OsMed7_1	LOC_Os04g56640	Os_chr04	33591163	[T/G]	INTRON
OsMed7_1	LOC_Os04g56640	Os_chr04	33591103	[A/T]	INTRON
OsMed7_1	LOC_Os04g56646	Os_chr04	33592719	[C/T]	REGULATORY
OsMed7_1	LOC_Os04g56646	Os_chr04	33592652	[A/T]	REGULATORY
OsMed7_1	LOC_Os04g56646	Os_chr04	33592476	[T/G]	REGULATORY
OsMed7_1	LOC_Os04g56646	Os_chr04	33592425	[G/T]	REGULATORY
OsMed7_1	LOC_Os04g56646	Os_chr04	33592414	[C/A]	REGULATORY
OsMed7_1	LOC_Os04g56640	Os_chr04	33589347	[T/C]	REGULATORY
OsMed7_1	LOC_Os04g56640	Os_chr04	33592298	[G/T]	REGULATORY
OsMed7_1	LOC_Os04g56640	Os_chr04	33590760	[C/T]	SYNONYMOUS-CODING
OsMed8_1	LOC_Os03g31010	Os_chr03	17672357	[G/A]	INTRON
OsMed8_1	LOC_Os03g31010	Os_chr03	17671969	[G/C]	INTRON
OsMed8_1	LOC_Os03g31010	Os_chr03	17671843	[A/G]	INTRON
OsMed8_1	LOC_Os03g31010	Os_chr03	17671788	[C/T]	INTRON
OsMed8_1	LOC_Os03g31010	Os_chr03	17671697	[T/C]	INTRON
OsMed8_1	LOC_Os03g31010	Os_chr03	17671062	[T/C]	INTRON
OsMed8_1	LOC_Os03g31010	Os_chr03	17671048	[A/G]	INTRON
OsMed8_1	LOC_Os03g31010	Os_chr03	17670809	[C/G]	INTRON
OsMed8_1	LOC_Os03g31010	Os_chr03	17670371	[C/A]	INTRON
OsMed8_1	LOC_Os03g31010	Os_chr03	17670315	[C/T]	INTRON
OsMed8_1	LOC_Os03g31010	Os_chr03	17670053	[T/C]	INTRON
OsMed8_1	LOC_Os03g31010	Os_chr03	17669204	[T/C]	INTRON
OsMed8_1	LOC_Os03g31010	Os_chr03	17669181	[G/T]	INTRON
OsMed8_1	LOC_Os03g31010	Os_chr03	17669157	[T/A]	INTRON
OsMed8_1	LOC_Os03g31010	Os_chr03	17669042	[G/T]	INTRON
OsMed8_1	LOC_Os03g31010	Os_chr03	17668965	[C/T]	INTRON

**Mediator genes**	**MSU locus ID**	**Chromosomes**	**Physical Position (bp)**	**SNPs**	**Structural Annotation**
OsMed8_1	LOC_Os03g31010	Os_chr03	17668787	[G/T]	INTRON
OsMed8_1	LOC_Os03g31010	Os_chr03	17668672	[G/A]	INTRON
OsMed8_1	LOC_Os03g31010	Os_chr03	17668640	[C/T]	INTRON
OsMed8_1	LOC_Os03g31010	Os_chr03	17668525	[A/G]	INTRON
OsMed8_1	LOC_Os03g31010	Os_chr03	17671340	[C/G]	NON-SYNONYMOUS-CODING
OsMed8_1	LOC_Os03g31010	Os_chr03	17671302	[G/A]	NON-SYNONYMOUS-CODING
OsMed8_1	LOC_Os03g31010	Os_chr03	17670760	[G/T]	NON-SYNONYMOUS-CODING
OsMed8_1	LOC_Os03g31010	Os_chr03	17672861	[C/T]	REGULATORY
OsMed8_1	LOC_Os03g31010	Os_chr03	17672854	[A/G]	REGULATORY
OsMed8_1	LOC_Os03g31010	Os_chr03	17672830	[C/T]	REGULATORY
OsMed8_1	LOC_Os03g31010	Os_chr03	17672787	[T/C]	REGULATORY
OsMed8_1	LOC_Os03g31010	Os_chr03	17672612	[C/T]	REGULATORY
OsMed8_1	LOC_Os03g31010	Os_chr03	17667895	[C/T]	REGULATORY
OsMed8_1	LOC_Os03g31010	Os_chr03	17667706	[T/C]	REGULATORY
OsMed8_1	LOC_Os03g31010	Os_chr03	17667701	[C/G]	REGULATORY
OsMed8_1	LOC_Os03g31010	Os_chr03	17667686	[A/G]	REGULATORY
OsMed8_1	LOC_Os03g31010	Os_chr03	17667660	[G/A]	REGULATORY
OsMed8_1	LOC_Os03g31010	Os_chr03	17667657	[C/T]	REGULATORY
OsMed8_1	LOC_Os03g31010	Os_chr03	17667652	[T/C]	REGULATORY
OsMed8_1	LOC_Os03g31010	Os_chr03	17667612	[G/A]	REGULATORY
OsMed8_1	LOC_Os03g31010	Os_chr03	17667606	[G/A]	REGULATORY
OsMed8_1	LOC_Os03g31010	Os_chr03	17667560	[A/G]	REGULATORY
OsMed8_1	LOC_Os03g31010	Os_chr03	17667544	[T/A]	REGULATORY
OsMed8_1	LOC_Os03g31010	Os_chr03	17667270	[C/T]	REGULATORY
OsMed8_1	LOC_Os03g31010	Os_chr03	17667235	[T/A]	REGULATORY
OsMed8_1	LOC_Os03g31010	Os_chr03	17667135	[A/T]	REGULATORY
OsMed8_1	LOC_Os03g31010	Os_chr03	17667081	[C/A]	REGULATORY
OsMed8_1	LOC_Os03g31010	Os_chr03	17672562	[A/G]	SYNONYMOUS-CODING
OsMed8_1	LOC_Os03g31010	Os_chr03	17672529	[C/T]	SYNONYMOUS-CODING
OsMed8_1	LOC_Os03g31010	Os_chr03	17671532	[A/G]	SYNONYMOUS-CODING
OsMed9_1	LOC_Os01g31629	Os_chr01	17324113	[T/A]	INTRON
OsMed9_1	LOC_Os01g31629	Os_chr01	17324002	[T/C]	INTRON
OsMed9_1	LOC_Os01g31629	Os_chr01	17323987	[A/T]	INTRON
OsMed9_1	LOC_Os01g31629	Os_chr01	17323828	[A/C]	INTRON
OsMed9_1	LOC_Os01g31629	Os_chr01	17323749	[C/T]	INTRON
OsMed9_1	LOC_Os01g31629	Os_chr01	17323687	[C/T]	INTRON
OsMed9_1	LOC_Os01g31629	Os_chr01	17323591	[G/A]	INTRON
OsMed9_1	LOC_Os01g31629	Os_chr01	17323572	[G/A]	INTRON
OsMed9_1	LOC_Os01g31629	Os_chr01	17323282	[C/G]	INTRON
OsMed9_1	LOC_Os01g31629	Os_chr01	17323176	[C/T]	INTRON
OsMed9_1	LOC_Os01g31629	Os_chr01	17323111	[G/T]	INTRON
OsMed9_1	LOC_Os01g31629	Os_chr01	17322951	[C/T]	INTRON
OsMed9_1	LOC_Os01g31629	Os_chr01	17322898	[C/T]	INTRON
OsMed9_1	LOC_Os01g31629	Os_chr01	17322893	[C/T]	INTRON
OsMed9_1	LOC_Os01g31629	Os_chr01	17322842	[T/G]	INTRON
OsMed9_1	LOC_Os01g31629	Os_chr01	17322429	[G/C]	INTRON
OsMed9_1	LOC_Os01g31629	Os_chr01	17322364	[G/C]	INTRON

**Mediator genes**	**MSU locus ID**	**Chromosomes**	**Physical Position (bp)**	**SNPs**	**Structural Annotation**
OsMed9_1	LOC_Os01g31629	Os_chr01	17322336	[A/C]	INTRON
OsMed9_1	LOC_Os01g31629	Os_chr01	17322144	[G/A]	INTRON
OsMed9_1	LOC_Os01g31629	Os_chr01	17322125	[A/G]	INTRON
OsMed9_1	LOC_Os01g31629	Os_chr01	17322055	[T/A]	INTRON
OsMed9_1	LOC_Os01g31629	Os_chr01	17321972	[T/C]	INTRON
OsMed9_1	LOC_Os01g31629	Os_chr01	17321965	[G/A]	INTRON
OsMed9_1	LOC_Os01g31629	Os_chr01	17321955	[C/A]	INTRON
OsMed9_1	LOC_Os01g31629	Os_chr01	17321496	[A/G]	INTRON
OsMed9_1	LOC_Os01g31629	Os_chr01	17321444	[G/A]	INTRON
OsMed9_1	LOC_Os01g31629	Os_chr01	17321365	[G/A]	INTRON
OsMed9_1	LOC_Os01g31629	Os_chr01	17321346	[G/T]	INTRON
OsMed9_1	LOC_Os01g31629	Os_chr01	17321344	[G/A]	INTRON
OsMed9_1	LOC_Os01g31629	Os_chr01	17321076	[C/T]	INTRON
OsMed9_1	LOC_Os01g31629	Os_chr01	17321072	[C/G]	INTRON
OsMed9_1	LOC_Os01g31629	Os_chr01	17321060	[T/G]	INTRON
OsMed9_1	LOC_Os01g31629	Os_chr01	17320816	[A/T]	INTRON
OsMed9_1	LOC_Os01g31629	Os_chr01	17320784	[G/A]	INTRON
OsMed9_1	LOC_Os01g31629	Os_chr01	17320769	[C/G]	INTRON
OsMed9_1	LOC_Os01g31629	Os_chr01	17320707	[G/A]	INTRON
OsMed9_1	LOC_Os01g31629	Os_chr01	17320620	[C/T]	INTRON
OsMed9_1	LOC_Os01g31629	Os_chr01	17320524	[G/A]	INTRON
OsMed9_1	LOC_Os01g31629	Os_chr01	17320351	[C/T]	INTRON
OsMed9_1	LOC_Os01g31629	Os_chr01	17320153	[T/A]	INTRON
OsMed9_1	LOC_Os01g31629	Os_chr01	17320101	[G/A]	INTRON
OsMed9_1	LOC_Os01g31629	Os_chr01	17319639	[A/G]	INTRON
OsMed9_1	LOC_Os01g31629	Os_chr01	17319566	[T/G]	INTRON
OsMed9_1	LOC_Os01g31629	Os_chr01	17319561	[T/C]	INTRON
OsMed9_1	LOC_Os01g31629	Os_chr01	17319543	[G/A]	INTRON
OsMed9_1	LOC_Os01g31629	Os_chr01	17319345	[G/A]	INTRON
OsMed9_1	LOC_Os01g31629	Os_chr01	17319341	[T/C]	INTRON
OsMed9_1	LOC_Os01g31629	Os_chr01	17319242	[G/A]	INTRON
OsMed9_1	LOC_Os01g31629	Os_chr01	17319003	[A/T]	INTRON
OsMed9_1	LOC_Os01g31629	Os_chr01	17318905	[T/C]	INTRON
OsMed9_1	LOC_Os01g31629	Os_chr01	17318881	[C/G]	INTRON
OsMed9_1	LOC_Os01g31629	Os_chr01	17318591	[C/T]	INTRON
OsMed9_1	LOC_Os01g31629	Os_chr01	17318581	[C/T]	INTRON
OsMed9_1	LOC_Os01g31629	Os_chr01	17318421	[C/T]	INTRON
OsMed9_1	LOC_Os01g31629	Os_chr01	17318332	[C/T]	INTRON
OsMed9_1	LOC_Os01g31629	Os_chr01	17318317	[G/A]	INTRON
OsMed9_1	LOC_Os01g31629	Os_chr01	17318310	[A/G]	INTRON
OsMed9_1	LOC_Os01g31629	Os_chr01	17318217	[C/T]	INTRON
OsMed9_1	LOC_Os01g31629	Os_chr01	17317839	[G/A]	INTRON
OsMed9_1	LOC_Os01g31629	Os_chr01	17317830	[C/T]	INTRON
OsMed9_1	LOC_Os01g31629	Os_chr01	17317824	[A/G]	INTRON
OsMed9_1	LOC_Os01g31629	Os_chr01	17317817	[T/G]	INTRON
OsMed9_1	LOC_Os01g31629	Os_chr01	17317796	[C/T]	INTRON
OsMed9_1	LOC_Os01g31629	Os_chr01	17317786	[G/T]	INTRON

Mediator genes	MSU locus ID	Chromosomes	Physical Position (bp)	SNPs	Structural Annotation
OsMed9_1	LOC_Os01g31629	Os_chr01	17317757	[G/A]	INTRON
OsMed9_1	LOC_Os01g31629	Os_chr01	17317747	[G/T]	INTRON
OsMed9_1	LOC_Os01g31629	Os_chr01	17317722	[A/G]	INTRON
OsMed9_1	LOC_Os01g31629	Os_chr01	17317605	[A/G]	INTRON
OsMed9_1	LOC_Os01g31629	Os_chr01	17317559	[G/T]	INTRON
OsMed9_1	LOC_Os01g31629	Os_chr01	17317554	[G/T]	INTRON
OsMed9_1	LOC_Os01g31629	Os_chr01	17317550	[G/A]	INTRON
OsMed9_1	LOC_Os01g31629	Os_chr01	17317547	[G/A]	INTRON
OsMed9_1	LOC_Os01g31629	Os_chr01	17317524	[G/A]	INTRON
OsMed9_1	LOC_Os01g31629	Os_chr01	17317463	[G/A]	INTRON
OsMed9_1	LOC_Os01g31629	Os_chr01	17317455	[G/A]	INTRON
OsMed9_1	LOC_Os01g31629	Os_chr01	17317437	[C/T]	INTRON
OsMed9_1	LOC_Os01g31629	Os_chr01	17317383	[G/A]	INTRON
OsMed9_1	LOC_Os01g31629	Os_chr01	17317376	[T/C]	INTRON
OsMed9_1	LOC_Os01g31629	Os_chr01	17317341	[T/C]	INTRON
OsMed9_1	LOC_Os01g31629	Os_chr01	17317243	[T/C]	INTRON
OsMed9_1	LOC_Os01g31629	Os_chr01	17317227	[G/T]	INTRON
OsMed9_1	LOC_Os01g31629	Os_chr01	17317144	[G/A]	INTRON
OsMed9_1	LOC_Os01g31629	Os_chr01	17317142	[C/T]	INTRON
OsMed9_1	LOC_Os01g31629	Os_chr01	17317139	[C/T]	INTRON
OsMed9_1	LOC_Os01g31629	Os_chr01	17317113	[A/G]	INTRON
OsMed9_1	LOC_Os01g31629	Os_chr01	17317108	[A/G]	INTRON
OsMed9_1	LOC_Os01g31629	Os_chr01	17317101	[C/A]	INTRON
OsMed9_1	LOC_Os01g31629	Os_chr01	17317080	[G/A]	INTRON
OsMed9_1	LOC_Os01g31629	Os_chr01	17317069	[G/A]	INTRON
OsMed9_1	LOC_Os01g31629	Os_chr01	17317053	[G/A]	INTRON
OsMed9_1	LOC_Os01g31629	Os_chr01	17317033	[G/A]	INTRON
OsMed9_1	LOC_Os01g31629	Os_chr01	17317031	[C/T]	INTRON
OsMed9_1	LOC_Os01g31629	Os_chr01	17317022	[C/T]	INTRON
OsMed9_1	LOC_Os01g31629	Os_chr01	17316950	[G/A]	INTRON
OsMed9_1	LOC_Os01g31629	Os_chr01	17316944	[C/T]	INTRON
OsMed9_1	LOC_Os01g31629	Os_chr01	17316804	[C/T]	INTRON
OsMed9_1	LOC_Os01g31629	Os_chr01	17316778	[G/C]	INTRON
OsMed9_1	LOC_Os01g31629	Os_chr01	17316742	[G/A]	INTRON
OsMed9_1	LOC_Os01g31629	Os_chr01	17316679	[G/A]	INTRON
OsMed9_1	LOC_Os01g31629	Os_chr01	17316638	[C/T]	INTRON
OsMed9_1	LOC_Os01g31629	Os_chr01	17316634	[G/A]	INTRON
OsMed9_1	LOC_Os01g31629	Os_chr01	17316626	[C/T]	INTRON
OsMed9_1	LOC_Os01g31629	Os_chr01	17316571	[G/A]	INTRON
OsMed9_1	LOC_Os01g31629	Os_chr01	17316464	[C/T]	INTRON
OsMed9_1	LOC_Os01g31629	Os_chr01	17316410	[G/A]	INTRON
OsMed9_1	LOC_Os01g31629	Os_chr01	17316397	[G/A]	INTRON
OsMed9_1	LOC_Os01g31629	Os_chr01	17316388	[C/A]	INTRON
OsMed9_1	LOC_Os01g31629	Os_chr01	17316338	[A/G]	INTRON
OsMed9_1	LOC_Os01g31629	Os_chr01	17316297	[A/G]	INTRON
OsMed9_1	LOC_Os01g31629	Os_chr01	17316267	[A/G]	INTRON
OsMed9_1	LOC_Os01g31629	Os_chr01	17316230	[C/T]	INTRON

**Mediator genes**	**MSU locus ID**	**Chromosomes**	**Physical Position (bp)**	**SNPs**	**Structural Annotation**
OsMed9_1	LOC_Os01g31629	Os_chr01	17316228	[T/C]	INTRON
OsMed9_1	LOC_Os01g31629	Os_chr01	17316198	[C/T]	INTRON
OsMed9_1	LOC_Os01g31629	Os_chr01	17316085	[A/C]	INTRON
OsMed9_1	LOC_Os01g31629	Os_chr01	17315940	[T/C]	INTRON
OsMed9_1	LOC_Os01g31629	Os_chr01	17315907	[G/A]	INTRON
OsMed9_1	LOC_Os01g31629	Os_chr01	17315879	[C/T]	INTRON
OsMed9_1	LOC_Os01g31629	Os_chr01	17315802	[G/A]	INTRON
OsMed9_1	LOC_Os01g31629	Os_chr01	17315778	[C/T]	INTRON
OsMed9_1	LOC_Os01g31629	Os_chr01	17315612	[G/A]	INTRON
OsMed9_1	LOC_Os01g31629	Os_chr01	17315443	[C/T]	INTRON
OsMed9_1	LOC_Os01g31629	Os_chr01	17315440	[C/T]	INTRON
OsMed9_1	LOC_Os01g31629	Os_chr01	17315429	[C/A]	INTRON
OsMed9_1	LOC_Os01g31629	Os_chr01	17315428	[T/A]	INTRON
OsMed9_1	LOC_Os01g31629	Os_chr01	17315422	[C/T]	INTRON
OsMed9_1	LOC_Os01g31629	Os_chr01	17315415	[G/A]	INTRON
OsMed9_1	LOC_Os01g31629	Os_chr01	17315278	[G/A]	INTRON
OsMed9_1	LOC_Os01g31629	Os_chr01	17315214	[G/A]	INTRON
OsMed9_1	LOC_Os01g31629	Os_chr01	17315172	[G/A]	INTRON
OsMed9_1	LOC_Os01g31629	Os_chr01	17315155	[G/A]	INTRON
OsMed9_1	LOC_Os01g31629	Os_chr01	17315127	[G/A]	INTRON
OsMed9_1	LOC_Os01g31629	Os_chr01	17315087	[C/T]	INTRON
OsMed9_1	LOC_Os01g31629	Os_chr01	17315056	[C/T]	INTRON
OsMed9_1	LOC_Os01g31629	Os_chr01	17315012	[G/A]	INTRON
OsMed9_1	LOC_Os01g31629	Os_chr01	17314994	[G/A]	INTRON
OsMed9_1	LOC_Os01g31629	Os_chr01	17314979	[G/A]	INTRON
OsMed9_1	LOC_Os01g31629	Os_chr01	17314934	[G/A]	INTRON
OsMed9_1	LOC_Os01g31629	Os_chr01	17314915	[A/C]	INTRON
OsMed9_1	LOC_Os01g31629	Os_chr01	17314863	[G/A]	INTRON
OsMed9_1	LOC_Os01g31629	Os_chr01	17314769	[G/A]	INTRON
OsMed9_1	LOC_Os01g31629	Os_chr01	17314750	[G/A]	INTRON
OsMed9_1	LOC_Os01g31629	Os_chr01	17314715	[T/C]	INTRON
OsMed9_1	LOC_Os01g31629	Os_chr01	17314704	[C/T]	INTRON
OsMed9_1	LOC_Os01g31629	Os_chr01	17314570	[G/A]	INTRON
OsMed9_1	LOC_Os01g31629	Os_chr01	17314560	[T/C]	INTRON
OsMed9_1	LOC_Os01g31629	Os_chr01	17314547	[G/A]	INTRON
OsMed9_1	LOC_Os01g31629	Os_chr01	17314529	[T/A]	INTRON
OsMed9_1	LOC_Os01g31629	Os_chr01	17314511	[G/A]	INTRON
OsMed9_1	LOC_Os01g31629	Os_chr01	17314478	[C/A]	INTRON
OsMed9_1	LOC_Os01g31629	Os_chr01	17314473	[C/T]	INTRON
OsMed9_1	LOC_Os01g31629	Os_chr01	17314444	[G/A]	INTRON
OsMed9_1	LOC_Os01g31629	Os_chr01	17314431	[C/T]	INTRON
OsMed9_1	LOC_Os01g31629	Os_chr01	17314416	[A/G]	INTRON
OsMed9_1	LOC_Os01g31629	Os_chr01	17314409	[C/T]	INTRON
OsMed9_1	LOC_Os01g31629	Os_chr01	17314381	[G/A]	INTRON
OsMed9_1	LOC_Os01g31629	Os_chr01	17314377	[C/T]	INTRON
OsMed9_1	LOC_Os01g31629	Os_chr01	17314297	[G/A]	INTRON
OsMed9_1	LOC_Os01g31629	Os_chr01	17314269	[G/A]	INTRON

**Mediator genes**	**MSU locus ID**	**Chromosomes**	**Physical Position (bp)**	**SNPs**	**Structural Annotation**
OsMed9_1	LOC_Os01g31629	Os_chr01	17314230	[G/A]	INTRON
OsMed9_1	LOC_Os01g31629	Os_chr01	17314228	[C/T]	INTRON
OsMed9_1	LOC_Os01g31629	Os_chr01	17314216	[C/T]	INTRON
OsMed9_1	LOC_Os01g31629	Os_chr01	17314206	[G/A]	INTRON
OsMed9_1	LOC_Os01g31629	Os_chr01	17314190	[T/A]	INTRON
OsMed9_1	LOC_Os01g31629	Os_chr01	17314150	[A/G]	INTRON
OsMed9_1	LOC_Os01g31629	Os_chr01	17314125	[C/T]	INTRON
OsMed9_1	LOC_Os01g31629	Os_chr01	17314031	[G/A]	INTRON
OsMed9_1	LOC_Os01g31629	Os_chr01	17314010	[G/A]	INTRON
OsMed9_1	LOC_Os01g31629	Os_chr01	17313973	[G/A]	INTRON
OsMed9_1	LOC_Os01g31629	Os_chr01	17313959	[G/A]	INTRON
OsMed9_1	LOC_Os01g31629	Os_chr01	17313952	[G/A]	INTRON
OsMed9_1	LOC_Os01g31629	Os_chr01	17313912	[G/A]	INTRON
OsMed9_1	LOC_Os01g31629	Os_chr01	17313900	[T/C]	INTRON
OsMed9_1	LOC_Os01g31629	Os_chr01	17313870	[T/C]	INTRON
OsMed9_1	LOC_Os01g31629	Os_chr01	17313863	[G/A]	INTRON
OsMed9_1	LOC_Os01g31629	Os_chr01	17313814	[G/A]	INTRON
OsMed9_1	LOC_Os01g31629	Os_chr01	17313796	[C/T]	INTRON
OsMed9_1	LOC_Os01g31629	Os_chr01	17313787	[G/A]	INTRON
OsMed9_1	LOC_Os01g31629	Os_chr01	17313757	[G/A]	INTRON
OsMed9_1	LOC_Os01g31629	Os_chr01	17313719	[C/T]	INTRON
OsMed9_1	LOC_Os01g31629	Os_chr01	17313703	[G/A]	INTRON
OsMed9_1	LOC_Os01g31629	Os_chr01	17313694	[G/A]	INTRON
OsMed9_1	LOC_Os01g31629	Os_chr01	17313620	[C/T]	INTRON
OsMed9_1	LOC_Os01g31629	Os_chr01	17313518	[C/T]	INTRON
OsMed9_1	LOC_Os01g31629	Os_chr01	17313415	[T/C]	INTRON
OsMed9_1	LOC_Os01g31629	Os_chr01	17313373	[C/A]	INTRON
OsMed9_1	LOC_Os01g31629	Os_chr01	17313365	[T/C]	INTRON
OsMed9_1	LOC_Os01g31629	Os_chr01	17313360	[G/T]	INTRON
OsMed9_1	LOC_Os01g31629	Os_chr01	17313347	[A/G]	INTRON
OsMed9_1	LOC_Os01g31629	Os_chr01	17313345	[C/T]	INTRON
OsMed9_1	LOC_Os01g31629	Os_chr01	17313337	[A/G]	INTRON
OsMed9_1	LOC_Os01g31629	Os_chr01	17313321	[T/C]	INTRON
OsMed9_1	LOC_Os01g31629	Os_chr01	17313316	[T/G]	INTRON
OsMed9_1	LOC_Os01g31629	Os_chr01	17313303	[G/A]	INTRON
OsMed9_1	LOC_Os01g31629	Os_chr01	17313288	[C/T]	INTRON
OsMed9_1	LOC_Os01g31629	Os_chr01	17313138	[G/A]	INTRON
OsMed9_1	LOC_Os01g31629	Os_chr01	17313021	[C/T]	INTRON
OsMed9_1	LOC_Os01g31629	Os_chr01	17313004	[G/A]	INTRON
OsMed9_1	LOC_Os01g31629	Os_chr01	17312999	[A/G]	INTRON
OsMed9_1	LOC_Os01g31629	Os_chr01	17312975	[G/A]	INTRON
OsMed9_1	LOC_Os01g31629	Os_chr01	17312934	[G/A]	INTRON
OsMed9_1	LOC_Os01g31629	Os_chr01	17312932	[A/G]	INTRON
OsMed9_1	LOC_Os01g31629	Os_chr01	17312919	[G/A]	INTRON
OsMed9_1	LOC_Os01g31629	Os_chr01	17312855	[A/T]	INTRON
OsMed9_1	LOC_Os01g31629	Os_chr01	17312732	[C/T]	INTRON
OsMed9_1	LOC_Os01g31629	Os_chr01	17312686	[G/A]	INTRON

**Mediator genes**	**MSU locus ID**	**Chromosomes**	**Physical Position (bp)**	**SNPs**	**Structural Annotation**
OsMed9_1	LOC_Os01g31629	Os_chr01	17312634	[C/T]	INTRON
OsMed9_1	LOC_Os01g31629	Os_chr01	17312625	[T/A]	INTRON
OsMed9_1	LOC_Os01g31629	Os_chr01	17312617	[C/T]	INTRON
OsMed9_1	LOC_Os01g31629	Os_chr01	17312591	[T/A]	INTRON
OsMed9_1	LOC_Os01g31629	Os_chr01	17312586	[G/A]	INTRON
OsMed9_1	LOC_Os01g31629	Os_chr01	17312531	[C/T]	INTRON
OsMed9_1	LOC_Os01g31629	Os_chr01	17312523	[C/T]	INTRON
OsMed9_1	LOC_Os01g31629	Os_chr01	17312513	[C/T]	INTRON
OsMed9_1	LOC_Os01g31629	Os_chr01	17312508	[A/G]	INTRON
OsMed9_1	LOC_Os01g31629	Os_chr01	17312460	[T/C]	INTRON
OsMed9_1	LOC_Os01g31629	Os_chr01	17312401	[G/C]	INTRON
OsMed9_1	LOC_Os01g31629	Os_chr01	17312380	[G/A]	INTRON
OsMed9_1	LOC_Os01g31629	Os_chr01	17312371	[G/A]	INTRON
OsMed9_1	LOC_Os01g31629	Os_chr01	17312366	[G/A]	INTRON
OsMed9_1	LOC_Os01g31629	Os_chr01	17312349	[C/A]	INTRON
OsMed9_1	LOC_Os01g31629	Os_chr01	17312287	[C/T]	INTRON
OsMed9_1	LOC_Os01g31629	Os_chr01	17312281	[A/C]	INTRON
OsMed9_1	LOC_Os01g31629	Os_chr01	17312245	[C/T]	INTRON
OsMed9_1	LOC_Os01g31629	Os_chr01	17312228	[C/T]	INTRON
OsMed9_1	LOC_Os01g31629	Os_chr01	17312211	[A/C]	INTRON
OsMed9_1	LOC_Os01g31629	Os_chr01	17312200	[G/A]	INTRON
OsMed9_1	LOC_Os01g31629	Os_chr01	17312147	[T/A]	INTRON
OsMed9_1	LOC_Os01g31629	Os_chr01	17312077	[T/G]	INTRON
OsMed9_1	LOC_Os01g31629	Os_chr01	17312066	[C/T]	INTRON
OsMed9_1	LOC_Os01g31629	Os_chr01	17312013	[G/A]	INTRON
OsMed9_1	LOC_Os01g31629	Os_chr01	17311994	[C/T]	INTRON
OsMed9_1	LOC_Os01g31629	Os_chr01	17311942	[T/C]	INTRON
OsMed9_1	LOC_Os01g31629	Os_chr01	17311927	[G/A]	INTRON
OsMed9_1	LOC_Os01g31629	Os_chr01	17311925	[G/A]	INTRON
OsMed9_1	LOC_Os01g31629	Os_chr01	17311835	[C/G]	INTRON
OsMed9_1	LOC_Os01g31629	Os_chr01	17311803	[G/A]	INTRON
OsMed9_1	LOC_Os01g31629	Os_chr01	17311776	[C/T]	INTRON
OsMed9_1	LOC_Os01g31629	Os_chr01	17311760	[G/T]	INTRON
OsMed9_1	LOC_Os01g31629	Os_chr01	17311717	[T/C]	INTRON
OsMed9_1	LOC_Os01g31629	Os_chr01	17311700	[C/A]	INTRON
OsMed9_1	LOC_Os01g31629	Os_chr01	17311686	[G/A]	INTRON
OsMed9_1	LOC_Os01g31629	Os_chr01	17311603	[T/A]	INTRON
OsMed9_1	LOC_Os01g31629	Os_chr01	17311596	[C/T]	INTRON
OsMed9_1	LOC_Os01g31629	Os_chr01	17311580	[C/T]	INTRON
OsMed9_1	LOC_Os01g31629	Os_chr01	17311530	[G/A]	INTRON
OsMed9_1	LOC_Os01g31629	Os_chr01	17311517	[C/T]	INTRON
OsMed9_1	LOC_Os01g31629	Os_chr01	17311510	[C/T]	INTRON
OsMed9_1	LOC_Os01g31629	Os_chr01	17311494	[G/C]	INTRON
OsMed9_1	LOC_Os01g31629	Os_chr01	17311458	[T/C]	INTRON
OsMed9_1	LOC_Os01g31629	Os_chr01	17311411	[A/C]	INTRON
OsMed9_1	LOC_Os01g31629	Os_chr01	17311383	[C/T]	INTRON
OsMed9_1	LOC_Os01g31629	Os_chr01	17311378	[A/G]	INTRON

Mediator genes	MSU locus ID	Chromosomes	Physical Position (bp)	SNPs	Structural Annotation
OsMed9_1	LOC_Os01g31629	Os_chr01	17311367	[A/C]	INTRON
OsMed9_1	LOC_Os01g31629	Os_chr01	17311273	[G/A]	INTRON
OsMed9_1	LOC_Os01g31629	Os_chr01	17311241	[A/G]	INTRON
OsMed9_1	LOC_Os01g31629	Os_chr01	17311176	[T/C]	INTRON
OsMed9_1	LOC_Os01g31629	Os_chr01	17311116	[C/T]	INTRON
OsMed9_1	LOC_Os01g31629	Os_chr01	17311112	[C/T]	INTRON
OsMed9_1	LOC_Os01g31629	Os_chr01	17311105	[C/A]	INTRON
OsMed9_1	LOC_Os01g31629	Os_chr01	17311091	[C/T]	INTRON
OsMed9_1	LOC_Os01g31629	Os_chr01	17310999	[C/T]	INTRON
OsMed9_1	LOC_Os01g31629	Os_chr01	17310959	[C/T]	INTRON
OsMed9_1	LOC_Os01g31629	Os_chr01	17310954	[C/A]	INTRON
OsMed9_1	LOC_Os01g31629	Os_chr01	17310945	[G/A]	INTRON
OsMed9_1	LOC_Os01g31629	Os_chr01	17310909	[G/T]	INTRON
OsMed9_1	LOC_Os01g31629	Os_chr01	17310898	[T/A]	INTRON
OsMed9_1	LOC_Os01g31629	Os_chr01	17310845	[C/T]	INTRON
OsMed9_1	LOC_Os01g31629	Os_chr01	17310841	[C/T]	INTRON
OsMed9_1	LOC_Os01g31629	Os_chr01	17310808	[G/C]	INTRON
OsMed9_1	LOC_Os01g31629	Os_chr01	17310778	[A/G]	INTRON
OsMed9_1	LOC_Os01g31629	Os_chr01	17310720	[G/A]	INTRON
OsMed9_1	LOC_Os01g31629	Os_chr01	17310705	[C/T]	INTRON
OsMed9_1	LOC_Os01g31629	Os_chr01	17310688	[G/C]	INTRON
OsMed9_1	LOC_Os01g31629	Os_chr01	17310650	[A/G]	INTRON
OsMed9_1	LOC_Os01g31629	Os_chr01	17310585	[C/T]	INTRON
OsMed9_1	LOC_Os01g31629	Os_chr01	17310456	[C/T]	INTRON
OsMed9_1	LOC_Os01g31629	Os_chr01	17310449	[C/T]	INTRON
OsMed9_1	LOC_Os01g31629	Os_chr01	17310422	[G/A]	INTRON
OsMed9_1	LOC_Os01g31629	Os_chr01	17310397	[T/C]	INTRON
OsMed9_1	LOC_Os01g31629	Os_chr01	17310373	[A/C]	INTRON
OsMed9_1	LOC_Os01g31629	Os_chr01	17310357	[G/A]	INTRON
OsMed9_1	LOC_Os01g31629	Os_chr01	17310349	[G/A]	INTRON
OsMed9_1	LOC_Os01g31629	Os_chr01	17310331	[G/A]	INTRON
OsMed9_1	LOC_Os01g31629	Os_chr01	17310251	[C/T]	INTRON
OsMed9_1	LOC_Os01g31629	Os_chr01	17310224	[T/G]	INTRON
OsMed9_1	LOC_Os01g31629	Os_chr01	17310216	[G/A]	INTRON
OsMed9_1	LOC_Os01g31629	Os_chr01	17310209	[G/T]	INTRON
OsMed9_1	LOC_Os01g31629	Os_chr01	17310180	[G/A]	INTRON
OsMed9_1	LOC_Os01g31629	Os_chr01	17310167	[T/C]	INTRON
OsMed9_1	LOC_Os01g31629	Os_chr01	17310144	[C/T]	INTRON
OsMed9_1	LOC_Os01g31629	Os_chr01	17310081	[C/T]	INTRON
OsMed9_1	LOC_Os01g31629	Os_chr01	17310065	[A/G]	INTRON
OsMed9_1	LOC_Os01g31629	Os_chr01	17310046	[G/T]	INTRON
OsMed9_1	LOC_Os01g31629	Os_chr01	17310043	[T/C]	INTRON
OsMed9_1	LOC_Os01g31629	Os_chr01	17309735	[C/A]	INTRON
OsMed9_1	LOC_Os01g31629	Os_chr01	17309678	[A/G]	INTRON
OsMed9_1	LOC_Os01g31629	Os_chr01	17309677	[C/T]	INTRON
OsMed9_1	LOC_Os01g31629	Os_chr01	17309670	[G/A]	INTRON
OsMed9_1	LOC_Os01g31629	Os_chr01	17309666	[A/G]	INTRON

**Mediator genes**	**MSU locus ID**	**Chromosomes**	**Physical Position (bp)**	**SNPs**	**Structural Annotation**
OsMed9_1	LOC_Os01g31629	Os_chr01	17309539	[G/C]	INTRON
OsMed9_1	LOC_Os01g31629	Os_chr01	17309513	[C/T]	INTRON
OsMed9_1	LOC_Os01g31629	Os_chr01	17309483	[T/C]	INTRON
OsMed9_1	LOC_Os01g31629	Os_chr01	17309401	[C/T]	INTRON
OsMed9_1	LOC_Os01g31629	Os_chr01	17309391	[C/A]	INTRON
OsMed9_1	LOC_Os01g31629	Os_chr01	17309220	[C/T]	INTRON
OsMed9_1	LOC_Os01g31629	Os_chr01	17309021	[C/T]	INTRON
OsMed9_1	LOC_Os01g31629	Os_chr01	17309013	[A/G]	INTRON
OsMed9_1	LOC_Os01g31629	Os_chr01	17308881	[C/T]	INTRON
OsMed9_1	LOC_Os01g31629	Os_chr01	17308875	[T/G]	INTRON
OsMed9_1	LOC_Os01g31629	Os_chr01	17324539	[C/A]	INTRON
OsMed9_1	LOC_Os01g31629	Os_chr01	17308255	[G/A]	REGULATORY
OsMed9_1	LOC_Os01g31629	Os_chr01	17308565	[G/T]	REGULATORY
OsMed9_1	LOC_Os01g31629	Os_chr01	17308500	[G/A]	REGULATORY
OsMed9_1	LOC_Os01g31629	Os_chr01	17325703	[G/A]	REGULATORY
OsMed9_1	LOC_Os01g31629	Os_chr01	17325642	[C/A]	REGULATORY
OsMed9_1	LOC_Os01g31629	Os_chr01	17325625	[C/T]	REGULATORY
OsMed9_1	LOC_Os01g31629	Os_chr01	17325616	[A/G]	REGULATORY
OsMed9_1	LOC_Os01g31629	Os_chr01	17325499	[T/G]	REGULATORY
OsMed9_1	LOC_Os01g31629	Os_chr01	17325399	[G/A]	REGULATORY
OsMed9_1	LOC_Os01g31629	Os_chr01	17325394	[T/C]	REGULATORY
OsMed9_1	LOC_Os01g31629	Os_chr01	17325322	[C/T]	REGULATORY
OsMed9_1	LOC_Os01g31629	Os_chr01	17325314	[T/G]	REGULATORY
OsMed9_1	LOC_Os01g31629	Os_chr01	17325248	[C/T]	REGULATORY
OsMed9_1	LOC_Os01g31629	Os_chr01	17325221	[G/A]	REGULATORY
OsMed9_1	LOC_Os01g31629	Os_chr01	17325147	[C/T]	REGULATORY
OsMed9_1	LOC_Os01g31629	Os_chr01	17325135	[A/T]	REGULATORY
OsMed9_1	LOC_Os01g31629	Os_chr01	17325056	[T/G]	REGULATORY
OsMed9_1	LOC_Os01g31629	Os_chr01	17325019	[G/A]	REGULATORY
OsMed9_1	LOC_Os01g31629	Os_chr01	17325017	[C/A]	REGULATORY
OsMed9_1	LOC_Os01g31629	Os_chr01	17325016	[T/A]	REGULATORY
OsMed9_1	LOC_Os01g31629	Os_chr01	17324979	[T/C]	REGULATORY
OsMed9_1	LOC_Os01g31629	Os_chr01	17324978	[G/A]	REGULATORY
OsMed9_1	LOC_Os01g31629	Os_chr01	17324967	[C/T]	REGULATORY
OsMed9_1	LOC_Os01g31629	Os_chr01	17324962	[C/G]	REGULATORY
OsMed9_1	LOC_Os01g31629	Os_chr01	17324958	[G/A]	REGULATORY
OsMed9_1	LOC_Os01g31629	Os_chr01	17324923	[C/T]	REGULATORY
OsMed9_1	LOC_Os01g31629	Os_chr01	17324916	[A/T]	REGULATORY
OsMed9_1	LOC_Os01g31629	Os_chr01	17324905	[C/T]	REGULATORY
OsMed9_1	LOC_Os01g31629	Os_chr01	17324896	[C/T]	REGULATORY
OsMed9_1	LOC_Os01g31629	Os_chr01	17324890	[C/T]	REGULATORY
OsMed9_1	LOC_Os01g31629	Os_chr01	17324887	[G/A]	REGULATORY
OsMed9_1	LOC_Os01g31629	Os_chr01	17324860	[G/A]	REGULATORY
OsMed9_1	LOC_Os01g31629	Os_chr01	17324841	[C/T]	REGULATORY
OsMed9_1	LOC_Os01g31629	Os_chr01	17324819	[G/C]	REGULATORY
OsMed9_1	LOC_Os01g31629	Os_chr01	17324809	[G/A]	REGULATORY
OsMed9_1	LOC_Os01g31629	Os_chr01	17324804	[T/C]	REGULATORY

Mediator genes	MSU locus ID	Chromosomes	Physical Position (bp)	SNPs	Structural Annotation
OsMed9_1	LOC_Os01g31629	Os_chr01	17324799	[G/T]	REGULATORY
OsMed9_1	LOC_Os01g31629	Os_chr01	17324793	[G/A]	REGULATORY
OsMed9_1	LOC_Os01g31629	Os_chr01	17324777	[G/A]	REGULATORY
OsMed9_1	LOC_Os01g31629	Os_chr01	17324771	[T/C]	REGULATORY
OsMed9_1	LOC_Os01g31629	Os_chr01	17324770	[A/G]	REGULATORY
OsMed9_1	LOC_Os01g31629	Os_chr01	17324740	[C/G]	REGULATORY
OsMed9_1	LOC_Os01g31629	Os_chr01	17322704	[C/T]	SYNONYMOUS-CODING
OsMed9_1	LOC_Os01g31629	Os_chr01	17322587	[T/G]	SYNONYMOUS-CODING
OsMed9_1	LOC_Os01g31629	Os_chr01	17308597	[A/T]	SYNONYMOUS-CODING
OsMed9_2	LOC_Os03g15560	Os_chr03	8570132	[T/C]	INTRON
OsMed9_2	LOC_Os03g15560	Os_chr03	8569812	[C/G]	INTRON
OsMed9_2	LOC_Os03g15560	Os_chr03	8569259	[C/T]	INTRON
OsMed9_2	LOC_Os03g15560	Os_chr03	8569080	[C/T]	INTRON
OsMed9_2	LOC_Os03g15560	Os_chr03	8569078	[C/G]	INTRON
OsMed9_2	LOC_Os03g15560	Os_chr03	8568677	[C/A]	INTRON
OsMed9_2	LOC_Os03g15560	Os_chr03	8568668	[A/T]	INTRON
OsMed9_2	LOC_Os03g15560	Os_chr03	8568650	[C/A]	INTRON
OsMed9_2	LOC_Os03g15560	Os_chr03	8568599	[G/A]	INTRON
OsMed9_2	LOC_Os03g15560	Os_chr03	8568369	[T/G]	INTRON
OsMed9_2	LOC_Os03g15560	Os_chr03	8568352	[G/T]	INTRON
OsMed9_2	LOC_Os03g15560	Os_chr03	8568324	[C/T]	INTRON
OsMed9_2	LOC_Os03g15560	Os_chr03	8568206	[A/T]	INTRON
OsMed9_2	LOC_Os03g15560	Os_chr03	8567469	[C/A]	REGULATORY
OsMed9_2	LOC_Os03g15560	Os_chr03	8570865	[G/T]	REGULATORY
OsMed9_2	LOC_Os03g15560	Os_chr03	8570647	[A/G]	REGULATORY
OsMed9_2	LOC_Os03g15560	Os_chr03	8570597	[G/A]	REGULATORY
OsMed9_2	LOC_Os03g15560	Os_chr03	8570466	[T/A]	REGULATORY
OsMed9_2	LOC_Os03g15560	Os_chr03	8570384	[A/G]	REGULATORY
OsMed9_2	LOC_Os03g15560	Os_chr03	8567357	[C/A]	REGULATORY
OsMed9_2	LOC_Os03g15560	Os_chr03	8567292	[C/T]	REGULATORY
OsMed9_2	LOC_Os03g15560	Os_chr03	8567274	[C/T]	REGULATORY
OsMed9_2	LOC_Os03g15560	Os_chr03	8567246	[T/C]	REGULATORY
OsMed9_2	LOC_Os03g15560	Os_chr03	8567237	[A/T]	REGULATORY
OsMed9_2	LOC_Os03g15560	Os_chr03	8567194	[G/A]	REGULATORY
OsMed9_2	LOC_Os03g15560	Os_chr03	8567141	[C/T]	REGULATORY
OsMed9_2	LOC_Os03g15560	Os_chr03	8567128	[G/T]	REGULATORY
OsMed9_2	LOC_Os03g15560	Os_chr03	8567100	[G/A]	REGULATORY
OsMed9_2	LOC_Os03g15560	Os_chr03	8567081	[C/T]	REGULATORY
OsMed9_2	LOC_Os03g15560	Os_chr03	8567080	[G/A]	REGULATORY
OsMed9_2	LOC_Os03g15560	Os_chr03	8567079	[C/T]	REGULATORY
OsMed9_2	LOC_Os03g15560	Os_chr03	8567046	[G/A]	REGULATORY
OsMed9_2	LOC_Os03g15560	Os_chr03	8567014	[A/G]	REGULATORY
OsMed9_2	LOC_Os03g15560	Os_chr03	8567004	[T/A]	REGULATORY
OsMed9_2	LOC_Os03g15560	Os_chr03	8566910	[G/A]	REGULATORY
OsMed9_2	LOC_Os03g15560	Os_chr03	8566892	[G/A]	REGULATORY
OsMed9_2	LOC_Os03g15560	Os_chr03	8566888	[G/A]	REGULATORY
OsMed9_2	LOC_Os03g15560	Os_chr03	8566881	[G/A]	REGULATORY

**Mediator genes**	**MSU locus ID**	**Chromosomes**	**Physical Position (bp)**	**SNPs**	**Structural Annotation**
OsMed9_2	LOC_Os03g15560	Os_chr03	8566874	[C/T]	REGULATORY
OsMed9_2	LOC_Os03g15560	Os_chr03	8566869	[A/G]	REGULATORY
OsMed9_2	LOC_Os03g15560	Os_chr03	8566858	[C/T]	REGULATORY
OsMed9_2	LOC_Os03g15560	Os_chr03	8566850	[C/T]	REGULATORY
OsMed9_2	LOC_Os03g15560	Os_chr03	8566846	[G/A]	REGULATORY
OsMed9_2	LOC_Os03g15560	Os_chr03	8566839	[T/A]	REGULATORY
OsMed9_2	LOC_Os03g15560	Os_chr03	8566838	[G/A]	REGULATORY
OsMed9_2	LOC_Os03g15560	Os_chr03	8566835	[G/C]	REGULATORY
OsMed9_2	LOC_Os03g15560	Os_chr03	8566831	[C/T]	REGULATORY
OsMed9_2	LOC_Os03g15560	Os_chr03	8566783	[G/A]	REGULATORY
OsMed9_2	LOC_Os03g15560	Os_chr03	8566759	[G/A]	REGULATORY
OsMed9_2	LOC_Os03g15560	Os_chr03	8566754	[G/A]	REGULATORY
OsMed9_2	LOC_Os03g15560	Os_chr03	8566736	[G/A]	REGULATORY
OsMed9_2	LOC_Os03g15560	Os_chr03	8566730	[G/A]	REGULATORY
OsMed9_2	LOC_Os03g15560	Os_chr03	8566721	[G/C]	REGULATORY
OsMed9_2	LOC_Os03g15560	Os_chr03	8566718	[A/G]	REGULATORY
OsMed9_2	LOC_Os03g15560	Os_chr03	8566715	[T/C]	REGULATORY
OsMed9_2	LOC_Os03g15560	Os_chr03	8566714	[T/A]	REGULATORY
OsMed9_2	LOC_Os03g15560	Os_chr03	8566705	[G/A]	REGULATORY
OsMed9_2	LOC_Os03g15560	Os_chr03	8566686	[C/T]	REGULATORY
OsMed9_2	LOC_Os03g15560	Os_chr03	8566524	[A/T]	REGULATORY
OsMed9_2	LOC_Os03g15560	Os_chr03	8570308	[G/A]	SYNONYMOUS-CODING
OsMed9_2	LOC_Os03g15560	Os_chr03	8569593	[G/C]	SYNONYMOUS-CODING
OsMed9_2	LOC_Os03g15560	Os_chr03	8567574	[C/T]	SYNONYMOUS-CODING

Table S2. Statistical measures for grain size trait evaluated in an association panel

**Grain size Traits**	**384 rice accessions**			
	**Mean ± S.D.**	**Range**	**Coefficient of variation (CV %)**	**Broad-sense Heritability (H^2 %)**
Grain length (mm)	8.5 ± 0.71	6.6 - 11.2	8	75
Grain width (mm)	2.8 ± 0.31	1.9 - 3.5	11	73
1000-grain weight (g)	26.5 ± 4.8	15.4 - 39.2	18	82

Table S3. ANOVA-based summary effect of grain size/weight trait measured in 384 rice accessions belonging to an association panel

**Traits**	**Environments**		**Source of variation**			
	**Years**	**Geographical locations**	Replication	Genotype (G)/Accessions	Block (Rep)	Error (e)
1000-grain weight (g) (GWg)	2012	New Delhi	41.8 (1)**	139.7 (383)*	18.7*	4.7 (383)**
	2012	Tamil Nadu	63.7 (1)*	187.6 (383)**	25.5*	7.5 (380)*
	2013	New Delhi	52.5 (1)*	153.7 (383)**	21.4*	4.2 (383)*
	2013	Tamil Nadu	71.3 (1)*	198.1 (383)**	29.8**	8.1 (380)*

Degree of freedom (*df*) are mentioned within Parentheses
*significant at 0.01 and **significant at 0.001

Table S4: **-ΔCt (Ct Ubiquitin- Ct Gene) values along with standard deviation used for heat map generation**

OsMED9_1

	Avg. S1	Avg. S2	Avg. S3	Avg. S4	Avg. S5	Avg. Lf		Stdev. S1	Stdev. S2	Stdev. S3	Stdev. S4	Stdev. S5	Stdev. Lf
**LGR**	-1.33744	-1.56478	-2.76861	-0.23278	-0.9955	-1.70456	**LGR**	0.24222	0.169536	0.264592	0.219032	0.097817	0.385333
**PB1121**	-1.61811	-2.38833	-2.29111	0.067889	-0.69517	-1.52967	**PB1121**	0.216836	0.333282	0.45031	0.42846	0.141186	0.02687
**IR64**	-1.90822	-2.78567	-2.03544	0.505	0.626444	-1.108	**IR64**	0.101802	0.057831	0.34862	0.284728	0.392833	0.269141
**NIPPONBARE**	-1.48989	-2.49489	-1.80611	0.753167	-0.88278	-1.05083	**NIPPONBARE**	0.262705	0.091836	0.10328	0.120444	0.204995	0.305725
**SONASAL**	-1.22933	-1.94744	-1.55833	-0.05417	-0.88333	-0.73922	**SONASAL**	0.231118	0.259428	0.196363	0.132229	0.198986	0.148937
**BINDLI**	-1.12044	-1.767	-3.16822	-1.05322	-0.37683	-0.97228	**BINDLI**	0.105579	0.253885	0.088366	0.137662	0.16287	0.226905

OsMED5_3

	Avg. S1	Avg. S2	Avg. S3	Avg. S4	Avg. S5	Avg. Lf		Stdev. S1	Stdev. S2	Stdev. S3	Stdev. S4	Stdev. S5	Stdev. Lf
**LGR**	-3.11911	-2.45633	-4.33467	-3.90122	-2.52178	-1.0745	**LGR**	0.100087	0.340518	0.275625	0.16417	0.346255	0.20294
**PB1121**	-2.01478	-2.68856	-3.26433	-2.954	-0.08567	-0.92967	**PB1121**	0.198001	0.506498	0.176306	0.249673	0.18479	0.151339
**IR64**	-3.06244	-3.85633	-4.78183	-1.95517	-0.36289	-1.519	**IR64**	0.187153	0.305825	0.292978	0.079904	0.152103	0.017442
**NIPPONBARE**	-2.333	-2.027	-2.40167	-1.60278	-0.61267	-0.41244	**NIPPONBARE**	0.269796	0.146117	0.167365	0.211217	0.123847	0.233936
**SONASAL**	-1.6915	-0.63367	-1.53333	-1.3145	0.322	-0.42589	**SONASAL**	0.033706	0.102766	0.373872	0.329748	0.084853	0.127329
**BINDLI**	-1.25244	-1.58211	-2.48144	-3.15556	-0.48633	-1.15567	**BINDLI**	0.298134	0.468044	0.140501	0.214785	0.276588	0.301511

OsMED11_1

	Avg. S1	Avg. S2	Avg. S3	Avg. S4	Avg. S5	Avg. Lf		Stdev. S1	Stdev. S2	Stdev. S3	Stdev. S4	Stdev. S5	Stdev. Lf
**LGR**	-0.51578	-1.08678	-0.84422	-0.56556	-0.79433	0.223667	**LGR**	0.119071	0.366895	0.114086	0.256991	0.05624	0.34686
**PB1121**	-1.19322	-1.44433	-0.29778	-0.76822	-0.01967	-0.44822	**PB1121**	0.214226	0.020743	0.190977	0.183669	0.12855	0.147938
**IR64**	-0.91456	-1.54767	-0.73744	-1.03083	-0.43444	-0.358	**IR64**	0.158386	0.144634	0.161579	0.267051	0.152155	0.04973
**NIPPONBARE**	-1.246	-1.18467	-0.22811	-0.51056	-0.41667	0.257278	**NIPPONBARE**	0.09938	0.212281	0.086923	0.050859	0.035554	0.227535
**SONASAL**	-1.38089	-1.009	-0.47689	0.101111	-0.18878	-0.61389	**SONASAL**	0.220292	0.174707	0.042505	0.094536	0.189919	0.071511
**BINDLI**	-0.63967	-1.10033	-0.31433	-0.89567	-0.23033	-0.32833	**BINDLI**	0.309241	0.387824	0.018856	0.225569	0.033942	0.324663

OsMED14_1

	Avg. S1	Avg. S2	Avg. S3	Avg. S4	Avg. S5	Avg. Lf		Stdev. S1	Stdev. S2	Stdev. S3	Stdev. S4	Stdev. S5	Stdev. Lf
**LGR**	-0.63533	-0.44433	-2.05856	-0.64956	0.182111	2.116666	**LGR**	0.228687	0.284727	0.438177	0.302283	0.46786	0.380443
**PB1121**	-0.10889	-0.814	-1.07983	-0.08817	0.707667	2.3605	**PB1121**	0.227551	0.042897	0.000708	0.281349	0.164992	0.254559
**IR64**	0.551667	-2.23744	-2.62433	-0.725	0.603667	1.896833	**IR64**	0.112148	0.226204	0.179134	0.141893	0.088962	0.002593
**NIPPONBARE**	-1.12883	-0.63017	-0.26244	0.519445	1.289889	1.925111	**NIPPONBARE**	0.15297	0.041248	0.153339	0.116871	0.267983	0.404036
**SONASAL**	-0.54683	0.381888	0.023333	0.379889	1.006889	2.876	**SONASAL**	0.276008	0.100211	0.011314	0.348105	0.184418	0.438124
**BINDLI**	-0.49783	-0.35994	-0.98444	-0.37842	1.515556	1.412333	**BINDLI**	0.062933	0.172879	0.246791	0.160631	0.055732	0.558143

	Avg.							Stdev.					
OsMED37_3													
	**S1**	**S2**	**S3**	**S4**	**S5**	**Lf**		**S1**	**S2**	**S3**	**S4**	**S5**	**Lf**
**LGR**	1.339	0.909166	0.392	1.593556	0.901334	3.117222	**LGR**	0.227461	0.199169	0.071183	0.202553	0.026623	0.394973
**PB1121**	-1.47456	-1.32283	-0.58717	-0.13467	-0.25044	0.274667	**PB1121**	0.26682	0.236881	0.015321	0.357795	0.172081	0.072125
**IR64**	3.865111	3.511778	2.151166	2.956166	1.2885	2.130111	**IR64**	0.145598	0.121788	0.554844	0.287321	0.028049	0.623033
**NIPPONBARE**	-0.66567	-1.23483	-0.81889	-0.43878	2.688	-1.37722	**NIPPONBARE**	0.300281	0.000236	0.095112	0.247353	0.118794	0.177166
**SONASAL**	0.468444	0.535222	0.287555	2.392889	0.106444	1.182333	**SONASAL**	0.246708	0.119127	0.229057	0.067479	0.071999	0.048555
**BINDLI**	2.442666	2.812	1.849444	-0.93933	-0.37333	-1.55467	**BINDLI**	0.178234	0.190202	0.346168	0.104639	0.193402	0.22598
OsMED25_1													
	**S1**	**S2**	**S3**	**S4**	**S5**	**Lf**		**S1**	**S2**	**S3**	**S4**	**S5**	**Lf**
**LGR**	-0.56133	0.151667	-0.96978	-0.19733	0.622111	0.339	**LGR**	0.212717	0.424301	0.247404	0.220031	0.086632	0.345012
**PB1121**	-1.72756	-0.47517	-0.47244	-0.617	-0.27511	0.289667	**PB1121**	0.133547	0.279778	0.460999	0.178779	0.276914	0.443097
**IR64**	-1.16817	-1.09989	-1.349	0.055667	-0.299	1.272833	**IR64**	0.07759	0.255913	0.167349	0.216648	0.157117	0.154856
**NIPPONBARE**	-1.10811	-0.06733	0.096111	0.599	0.792555	0.957556	**NIPPONBARE**	0.261922	0.175361	0.131477	0.160327	0.19842	0.14983
**SONASAL**	-0.52067	0.832444	0.285166	0.183111	0.434166	1.780444	**SONASAL**	0.467174	0.1053	0.039833	0.383278	0.064818	0.309674
**BINDLI**	-0.85133	-0.21222	-0.33183	-0.07033	1.241666	0.8525	**BINDLI**	0.140949	0.06979	0.317641	0.066467	0.269105	0.089317
OsMED15_1													
	**S1**	**S2**	**S3**	**S4**	**S5**	**Lf**		**S1**	**S2**	**S3**	**S4**	**S5**	**Lf**
**LGR**	-7.40678	-5.80433	-2.41944	-2.06867	-2.97944	-8.52056	**LGR**	0.48252	0.344374	0.263434	0.43646	0.365106	0.764332
**PB1121**	-6.44289	-3.55117	-1.95183	-4.64744	-1.44267	-9.08383	**PB1121**	0.385341	0.269408	0.268936	0.509749	0.293214	0.049262
**IR64**	-14.3108	-8.27506	-8.01267	-2.30144	-5.56089	-11.8482	**IR64**	1.032139	0.346981	0.9452	0.191996	0.213395	0.469272
**NIPPONBARE**	-7.129	-2.57533	-1.41511	-1.7655	-2.39633	-8.40222	**NIPPONBARE**	0.314848	0.300401	0.097766	0.152028	0.265102	0.692009
**SONASAL**	-2.818	-1.381	-1.20333	-1.61183	-1.67617	-6.09144	**SONASAL**	0.575388	0.007424	0.115023	0.325172	0.268465	0.5198
**BINDLI**	-5.38667	-4.10333	-2.15156	-2.39933	-1.84689	-8.95767	**BINDLI**	0.330535	0.382267	0.401713	0.010371	0.258958	0.708994
OsMED20_1													
	**S1**	**S2**	**S3**	**S4**	**S5**	**Lf**		**S1**	**S2**	**S3**	**S4**	**S5**	**Lf**
**LGR**	-3.24033	-3.09178	-4.88611	-4.28189	-3.58744	-1.82622	**LGR**	0.148021	0.024745	0.258555	0.027718	0.007582	0.252744
**PB1121**	-1.87183	-2.23922	-3.0545	-2.12567	-0.4215	-1.53767	**PB1121**	0.103944	0.264011	0.118086	0.381883	0.001179	0.428812
**IR64**	-3.498	-4.10856	-5.51711	-2.59828	-1.917	-2.69278	**IR64**	0.294614	0.158432	0.381875	0.10441	0.060339	0.516966
**NIPPONBARE**	-1.9795	-1.82467	-2.255	-1.82833	-1.821	-0.14956	**NIPPONBARE**	0.180784	0.153386	0.12901	0.356255	0.355115	0.378662
**SONASAL**	-0.791	-0.13467	-0.9055	-0.81717	-0.74467	-0.52033	**SONASAL**	0.150378	0.133879	0.096402	0.393387	0.183376	0.298681
**BINDLI**	-0.98011	-1.47633	-2.41933	-2.24922	-0.786	-1.14144	**BINDLI**	0.190053	0.049498	0.312925	0.032662	0.246662	0.520213

OsMED12_2

	Avg.							Stdev.					
	**S1**	**S2**	**S3**	**S4**	**S5**	**Lf**		**S1**	**S2**	**S3**	**S4**	**S5**	**Lf**
**LGR**	-3.16478	-4.23167	-0.96117	1.864111	2.229112	-2.71417	**LGR**	0.076085	0.151791	0.017678	0.239697	0.155544	0.210482
**PB1121**	-3.51889	-4.28167	-0.56833	-0.30622	-1.36344	-3.17589	**PB1121**	0.32371	0.425206	0.279061	0.595721	0.24688	0.262652
**IR64**	-3.54778	-4.95633	-0.80733	0.5895	-1.13367	-2.84422	**IR64**	0.761631	0.276171	0.26936	0.38726	0.309371	0.211748
**NIPPONBARE**	-2.98611	-2.63367	0.519556	1.076222	1.694667	-2.37367	**NIPPONBARE**	0.20919	0.338202	0.254578	0.110695	0.150727	0.098524
**SONASAL**	-2.02417	-2.52089	-0.45417	0.231889	-0.85156	-3.23389	**SONASAL**	0.092631	0.119333	0.154385	0.357824	0.042458	0.2688
**BINDLI**	-2.54411	-3.94111	0.700666	1.297778	1.222111	-3.80744	**BINDLI**	0.06837	0.064188	0.302045	0.097189	0.160959	0.274468

OsMED4_2

	**S1**	**S2**	**S3**	**S4**	**S5**	**Lf**		**S1**	**S2**	**S3**	**S4**	**S5**	**Lf**
**LGR**	-13.6527	-12.0522	-12.2466	-12.6138	-9.67017	-10.8797	**LGR**	0.361902	0.1525	0.37639	0.396632	0.086891	0.507329
**PB1121**	-15.0387	-12.2937	-11.999	-3.396	-1.36717	-10.8843	**PB1121**	0.127279	1.186173	0.225332	0.096167	0.051618	0.10703
**IR64**	-14.107	-17.6698	-13.6473	-5.51311	-2.137	-11.9053	**IR64**	0.068824	1.612611	1.080404	0.278418	0.016499	0.337915
**NIPPONBARE**	-11.3294	-11.8575	-8.89389	-11.3445	-8.89178	-12.3734	**NIPPONBARE**	0.251122	0.04549	0.375861	1.146769	0.405702	0.359215
**SONASAL**	-3.86817	-3.66422	-4.03878	-3.47333	-1.1635	-11.7537	**SONASAL**	0.170413	0.21754	0.331454	0.461977	0.048791	0.283498
**BINDLI**	-12.0583	-12.6122	-11.3115	-11.3725	-8.948	-10.5996	**BINDLI**	0.156978	0.69452	0.266579	1.616479	0.436802	0.183806

Avg.= Average -ΔCt values calculated for three biological replicates
Stdev.= Standard deviation calculated for -ΔCt from three biological replicates

Table S5. Primers used for differential expression analysis of grain size/weight-associated *MED* genes in rice

**Gene accession IDs**	**Gene identities**	**Forward Primer sequence (5'-3')**	**Reverse Primer sequences (5'-3')**	**Amplified fragment size (bp)**	**Annealing temperature (^{0}C)**
LOC_Os11g05150	*OsMED4_2*	GCAAAAGGATGGTGGAACCA	AGGCCTGGAAATTCTGAAGATCT	67	60
LOC_Os07g48350	*OsMED5_3*	GGAAAATGGTCCCGATGAATG	TTCGATGGAATGAAGAGAAGCA	65	60
LOC_Os02g09600	*OsMED11_1*	CACAAGTGTGACTGAGCGGTTT	GCATTTGCAAGCTGGCTACA	63	60
LOC_Os10g40260	*OsMED12_2*	TCAAGTGGCTATGCCGATTCT	GAGGACAGTAGAGGAGGACCACAT	72	60
LOC_Os08g24400	*OsMED14_1*	CAGCAGGTTCCTTTGGTTCATT	GTCTCATGGCTAGAGAGGGTTGA	62	60
LOC_Os04g03860	*OsMED15_1*	CACATCGCAATCGCAGCTTA	ACAAGCACGGCATGAATCAA	61	60
LOC_Os09g27140	*OsMED20_1*	TGACTTTTGCCTGAGGATTGG	CATCATGATTCCCCTCAGTGTTT	67	60
LOC_Os09g13610	*OsMED25_1*	TTCATTGTCGGTGGTATCTCCTAA	TTCCTCTTTGCCGCATTGTAT	69	60
LOC_Os03g16860	*OsMED37_3*	TGCTGATCCCCAGGAACAC	CGAACTTGCCGAGCAGGTT	144	60
LOC_Os01g22490	*UBQ5*	ACCACTTCGACCGCCACTACT	ACGCCTAAGCCTGCTGGTT	69	60
